# Sulfonamine-containing aurone derivatives act as inhibitors of intercellular movement of potato virus Y

**DOI:** 10.1016/j.isci.2025.113914

**Published:** 2025-10-30

**Authors:** Dan Chen, Qingqing Ma, Xin Li, Tao Yang, Shang Wu, Yanju Wang, Deyu Hu

**Affiliations:** 1 State Key Laboratory of Green Pesticide, Center for R&D of Fine Chemicals of Guizhou University, Guiyang 550025, P.R. China

**Keywords:** biological sciences, plant biology

## Abstract

Intercellular movement plays an important role in plant virus infection and transmission, which provides an opportunity for antiviral agents that interfere with the intercellular motion. However, there are still few compounds that directly interfere with the movement of viruses between cells. Herein, a series of aurone derivatives containing sulfonamide active groups were designed and synthesized. Among them, compound **D13** effectively inhibits virus infection by forming a stable hydrogen bond with Pro154 (P154) of *potato virus Y* (PVY) coat protein (CP). Further studies have shown that **D13** can effectively interfere with the process of P154-mediated viral intercellular trafficking in plasmodesmata (PD). This study emphasizes the core position of virus intercellular movement in plant virus diseases and its inhibition strategy and confirms that the aurone derivative **D13** is a candidate molecule for antiviral pesticides or intercellular movement inhibitors, providing a new direction for plant virus prevention and control.

## Highlights

• One aurone derivative showing good antiviral activity against PVY was screened
• An inhibitor of intercellular movement based on PVY CP was found
• Based on the new site of PVY CP, its key role in pathogenicity was verified

## Graphical abstract

**Figure:**
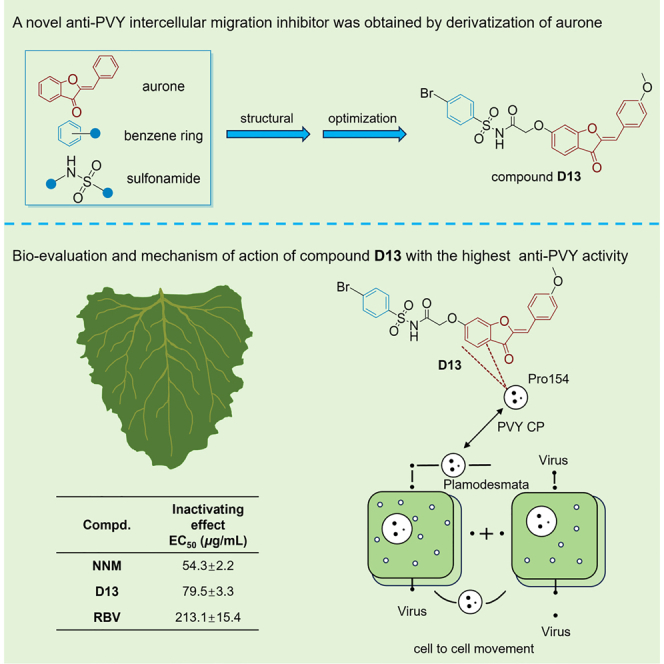

## Introduction

**Figure 1:**
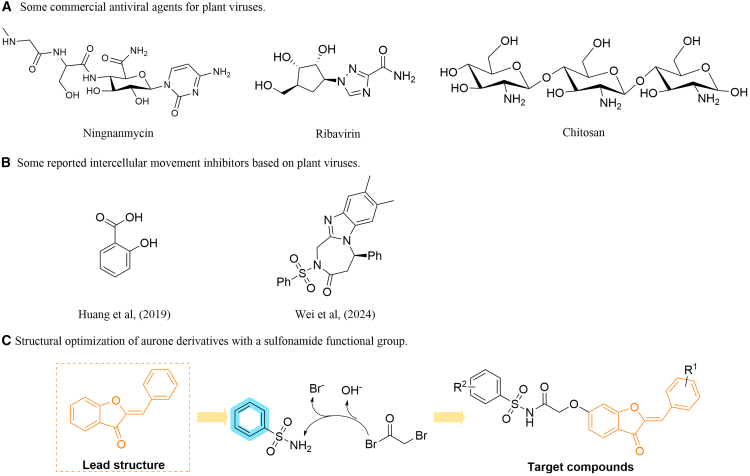
Research design of aurones-based plant antiviral agents (A) Some commercial antiviral agents for plant viruses. (B) Some reported intercellular movement inhibitors based on plant viruses. (C) Structural optimization of aurone derivatives with a sulfonamide functional group.

Potato (*Solanum tuberosum* L.) is considered the fourth most important staple crop worldwide, following maize, wheat, and rice, and is widely grown in more than 160 countries, with significant yield increases in emerging economies like China and India. As a crucial food source, potato is rich in energy, carbohydrates, and dietary fiber, and plays a key role in global food security and nutritional supply.^1^^,^^2^^,^^3^^,^^4^^,^^5^^,^^6^ However, plant viruses are major pathogens that affect crop yield and quality, and their outbreaks can cause severe direct and indirect economic losses, threatening global food supply and agricultural sustainability.^7^ The genus *Potyvirus* represents the most extensive category of plant viruses currently identified, comprising nearly 30% of all known plant viral species, and can infect a variety of dicot and monocot crops. *Potato virus Y* (PVY) is considered among the most significant representative members due to its significant economic impact.^8^^,^^9^^,^^10^ PVY was first reported in China in 1950, and by the 1980s, it had spread widely across most tobacco-growing areas.^11^^,^^12^ The results showed that PVY infection was mainly manifested as variety degradation, yield decline, and poor quality, which seriously affected agricultural production and caused significant economic losses. Infected potato plants can reduce yield and quality, with yield losses ranging from 10% to 80%.^13^^,^^14^^,^^15^^,^^16^^,^^17^^,^^18^^,^^19^^,^^20^^,^^21^ Furthermore, PVY exhibits a high degree of parasitic specificity, and plants lack a complete immune metabolic system, making field control of this virus particularly challenging.^22^ Even more concerning is that PVY not only infects plants independently but can also co-infect with other pathogens, such as *potato virus X* (PVX), *potato leafroll virus* (PLRV), *cucumber mosaic virus* (CMV), and *tomato mild mottle virus* (TMMOV), exacerbating the severity of the disease and increasing control difficulties.^23^^,^^24^^,^^25^^,^^26^ Currently, the most commonly used commercial antiviral agents are ningnanmycin (NNM), ribavirin (RBV), and chitosan (Figure 1A). However, effective and broad-spectrum antiviral agents for plant viral diseases are rare, and comprehensive control measures are required, which limits their application in sustainable agriculture. Therefore, developing highly effective inhibitors targeting key molecules provides a precise, safe, and sustainable solution for controlling plant viral diseases, playing an essential role in the advancement of sustainable agriculture.

The coat protein (CP) of PVY plays an essential role in the plant virus infection process, participating in key steps such as virus particle assembly, cell-to-cell movement, and systemic spread over long distances.^27^ Plant virus infection depends on efficient replication within cells and effective intercellular transmission. Virus movement through Plasmodesmata (PD) is a critical step for establishing systemic infection.^28^^,^^29^ Therefore, inhibiting virus movement between cells can effectively limit its infection range and reduce its pathogenicity. Despite the importance of intercellular movement in the viral infection process, research on specific inhibitors targeting this process remains limited. Although several potential key amino acid residues in the CP have been identified and studied, the specific role of these residues in virus movement between cells and their necessity for the proper execution of this function have not been fully elucidated. Our research team has confirmed that the key amino acid residue Ser125 (S125) and Arg157 (R157) on CP are both involved in the association among viral RNA (vRNA) and CP, which is essential for virus particle assembly and facilitates the virus’s ability to establish a systemic infection.^30^^,^^31^ Further investigation of the mechanisms behind these key amino acid residues will provide new theoretical insights for the control of viral movement between cells and lay the foundation for the development of novel antiviral strategies.

PD are specialized cytoplasmic connections in plant cells that facilitate the transcellular transport of water, nutrients, small signaling molecules, proteins, and RNA across the cell wall. The intercellular movement of plant viruses also relies on PD. Currently, research on specific and universal inhibitors targeting PD or intercellular movement remains limited. However, studies have identified potential mechanisms and factors that may influence PD function and intercellular material transport (Figure 1B).^32^^,^^33^^,^^34^^,^^35^^,^^36^ In 2007, Hofius et al. first discovered that the *Nicotiana tabacum* capsid protein interacting protein (NtCPIP) plays a critical role during PVY infection in tobacco, and confirmed that this protein is critical for the assembly of virus particles and their movement between cells via PD.^27^ Studies have shown that the Arg191 (R191) residue in the PVY CP interacts with NtCPIP, mediating the virus’s cell-to-cell movement.^33^ These findings provide experimental validation for previously reported discoveries. Furthermore, Asp6 (D6) and Gly9 (G9) residues have been identified as key sites closely related to PVY infection symptoms, potentially mediating interactions between the virus and host cells.^37^ Given the limited research on specific inhibitors targeting viral spread between adjacent cells, identifying more critical sites that significantly impact viral intercellular movement and further elucidating the mechanisms of different small molecules at these sites are crucial for the development of antiviral drugs. This study explores the potential applications of small molecules in interfering with viral cell-to-cell movement through structural optimization and target screening, providing theoretical support for the development of novel plant antiviral agents.

In this study, a series of structurally optimized aurone derivatives containing sulfonamide active groups were designed and synthesized based on a molecular hybridization strategy. Bioactivity tests revealed that some of the compounds exhibited significant curative, protective, and inactivating effects against PVY. Within these, compound **D13** demonstrated excellent antiviral activity by targeting the PVY CP (Figure 1C). Its inactivating activity, with a 50% effective concentration (EC_50_) of 79.5 *μ*g/mL, was significantly superior to the commercial antiviral agent RBV, whose EC_50_ is 213.1 *μ*g/mL. Molecular docking and microscale thermophoresis (MST) further confirmed that compound **D13** forms stable hydrogen bonds with Pro154 (P154), a key residue in CP. Further studies have shown that P154 plays a crucial role in the movement of the virus between cells, while **D13** can interfere with the function of this site and block the spread of the virus within the host, thereby reducing the pathogenicity of the virus. This study provides a new research idea for designing more targeted antiviral molecules.

## Results and discussion

### Chemistry

**Figure 2:**
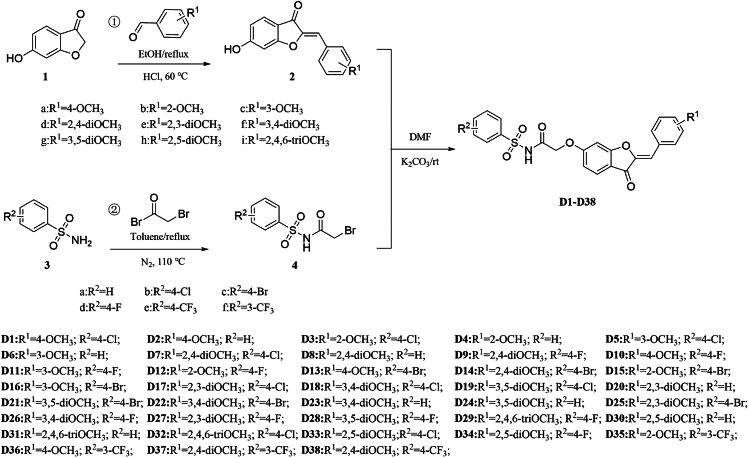
Synthesis route of the target compounds **D1−D38**

First, various substituted benzaldehydes and compound **1** were refluxed in ethanol for 6 h. The mixture underwent an aldehyde-ketone condensation reaction in an acidic solution at 60°C to yield intermediate **2**. Next, different substituted benzenesulfonamides underwent a nucleophilic substitution reaction with halogenated acyl esters under nitrogen flow to yield intermediate **4**. Finally, using K_2_CO_3_ as a base, the target compounds **D1−D38** were prepared through a bromination reaction involving the key intermediates **2** and **4** (Figure 2). The molecular structures of the target compounds **D1−D38** were elucidated using NMR and HRMS techniques. See Data S2.

### Antiviral activity assay

**Figure 3:**
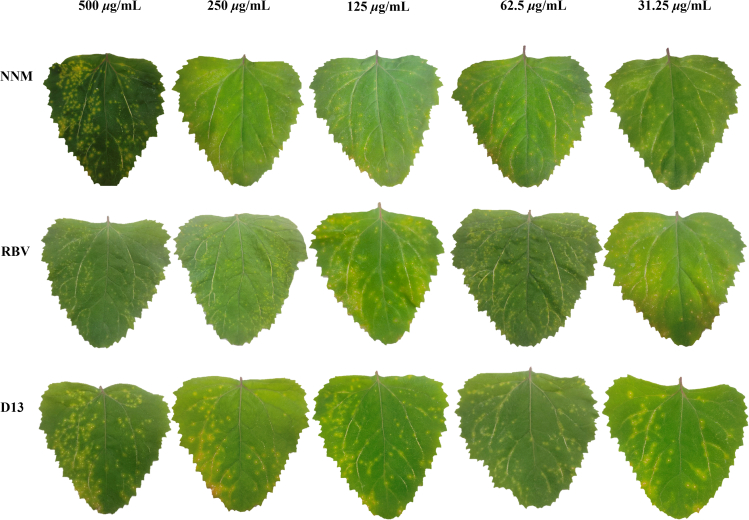
The effect of compound **D13** on PVY inactivation activity at different concentrations, with NNM and RBV as controls The concentrations are marked above the leaves. 25 mm equivalent focal length, object distance ≈10 cm, field of view ≈6 cm × 4 cm.

The half-leaf wilting method was used to systematically evaluate the antiviral effect of the target compounds **D1−D38** against PVY at a concentration of 500 *μ*g/mL,^38^ using *Chenopodium amaranticolor* as the dead-spot host and RBV and NNM as positive controls, as illustrated in Table 1. The majority of the target compounds demonstrated antiviral activity ranging from moderate to excellent, with eight compounds (**D2**, **D3**, **D4**, **D5**, **D13**, **D17**, **D24**, and **D27**) showing 60%–80% inactivation activity, significantly higher than the commercial drug RBV (59.3%). Compound **D13** demonstrated comparable or better curative and protective effects than NNM. Therefore, to further assess the inhibition activity of the compounds against PVY, we determined the EC_50_ values of some target compounds. As shown in Table 2, the inactivation EC_50_ values of **D1**, **D17**, **D24**, and **D13** were 307.2, 141.7, 147.3, and 79.5 *μ*g/mL, respectively. In comparison, compound **D13** exhibited a superior EC_50_ value compared to RBV (EC_50_ = 213.1 *μ*g/mL), but lower than NNM (EC_50_ = 54.3 *μ*g/mL) (Figure 3). The dose-response curve and the raw data can be seen in the supporting information Figure S1. Given that compound **D13** showed significant antiviral activity, we next investigated its preliminary mechanism of action.

**Table 1:** Antiviral activity of the title compounds against PVY at 500 *μ*g/mL

Compd.		Inactivating effect (%)^a^	Curative effect (%)^a^	Protective effect (%)^a^
**D1**	R^1^ = 4-OCH_3_; R^2^ = 4-Cl	50.0 ± 3.5	38.7 ± 3.7	44.8 ± 3.8
**D2**	R^1^ = 4-OCH_3_; R^2^ = H	65.1 ± 5.0	41.4 ± 2.9	36.9 ± 2.7
**D3**	R^1^ = 2-OCH_3_; R^2^ = 4-Cl	63.0 ± 4.7	40.1 ± 3.8	36.9 ± 2.9
**D4**	R^1^ = 2-OCH_3_; R^2^ = H	63.1 ± 2.2	43.1 ± 2.9	33.8 ± 1.0
**D5**	R^1^ = 3-OCH_3_; R^2^ = 4-Cl	60.7 ± 3.7	41.7 ± 3.5	37.6 ± 4.8
**D6**	R^1^ = 3-OCH_3_; R^2^ = H	58.8 ± 4.5	36.5 ± 2.9	40.5 ± 0.8
**D7**	R^1^ = 2,4-diOCH_3_; R^2^ = 4-Cl	42.9 ± 4.3	26.7 ± 2.9	40.9 ± 3.0
**D8**	R^1^ = 2,4-diOCH_3_; R^2^ = H	55.1 ± 4.8	38.0 ± 4.1	34.0 ± 2.7
**D9**	R^1^ = 2,4-diOCH_3_; R^2^ = 4-F	58.1 ± 5.4	45.7 ± 3.3	35.6 ± 2.5
**D10**	R^1^ = 4-OCH_3_; R^2^ = 4-F	43.7 ± 5.0	24.4 ± 1.9	33.9 ± 4.0
**D11**	R^1^ = 3-OCH_3_; R^2^ = 4-F	52.9 ± 2.8	40.6 ± 2.0	42.7 ± 2.8
**D12**	R^2^ = 2-OCH_3_; R^2^ = 4-F	59.4 ± 3.6	34.1 ± 3.5	44.5 ± 1.5
**D13**	R^1^ = 4-OCH_3_; R^2^ = 4-Br	75.3 ± 3.7	53.2 ± 1.6	47.7 ± 1.4
**D14**	R^1^ = 2,4-diOCH_3_; R^2^ = 4-Br	49.2 ± 4.2	29.8 ± 4.8	37.9 ± 4.4
**D15**	R^1^ = 2-OCH_3_; R^2^ = 4-Br	53.4 ± 4.2	37.1 ± 0.9	39.3 ± 2.9
**D16**	R^1^ = 3-OCH_3_; R^2^ = 4-Br	42.9 ± 4.3	29.0 ± 1.6	36.9 ± 2.4
**D17**	R^1^ = 2,3-diOCH_3_; R^2^ = 4-Cl	69.3 ± 2.2	46.2 ± 1.4	40.7 ± 2.3
**D18**	R^1^ = 3,4-diOCH_3_; R^2^ = 4-Cl	56.8 ± 2.8	25.5 ± 4.0	38.2 ± 3.0
**D19**	R^1^ = 3,5-diOCH_3_; R^2^ = 4-Cl	46.0 ± 3.0	36.4 ± 5.6	35.7 ± 3.8
**D20**	R^1^ = 2,3-diOCH_3_; R^2^ = H	64.7 ± 3.4	34.9 ± 4.9	31.3 ± 3.8
**D21**	R^1^ = 3,5-diOCH_3_; R^2^ = 4-Br	50.5 ± 1.6	33.0 ± 3.9	22.7 ± 1.2
**D22**	R^2^ = 3,4-diOCH_3_; R^2^ = 4-Br	52.0 ± 3.0	36.2 ± 1.5	28.1 ± 2.3
**D23**	R^1^ = 3,4-diOCH_3_; R^2^ = H	45.4 ± 1.1	35.3 ± 1.5	33.4 ± 3.9
**D24**	R^1^ = 3,5-diOCH_3_; R^2^ = H	68.5 ± 2.7	50.1 ± 1.8	31.0 ± 3.0
**D25**	R^1^ = 2,3-diOCH_3_; R^2^ = 4-Br	55.4 ± 2.0	35.9 ± 3.9	41.9 ± 2.6
**D26**	R^1^ = 3,4-diOCH_3_; R^2^ = 4-F	55.3 ± 3.0	32.4 ± 1.7	40.2 ± 5.0
**D27**	R^1^ = 2,3-diOCH_3_; R^2^ = 4-F	64.1 ± 4.2	45.1 ± 3.7	33.9 ± 2.3
**D28**	R^1^ = 3,5-diOCH_3_; R^2^ = 4-F	56.4 ± 4.7	39.9 ± 2.9	32.5 ± 4.4
**D29**	R^1^ = 2,4,6-triOCH_3_; R^2^ = 4-F	57.1 ± 4.3	36.7 ± 4.0	37.9 ± 2.7
**D30**	R^1^ = 2,5-diOCH_3_; R^2^ = H	49.4 ± 3.4	33.3 ± 1.4	34.2 ± 3.3
**D31**	R^1^ = 2,4,6-triOCH_3_; R^2^ = H	57.4 ± 4.3	42.9 ± 2.4	26.3 ± 2.6
**D32**	R^1^ = 2,4,6-triOCH_3_; R^2^ = 4-Cl	53.8 ± 3.0	43.4 ± 2.3	33.3 ± 2.6
**D33**	R^2^ = 2,5-diOCH_3_;R^2^ = 4-Cl	51.1 ± 3.9	41.2 ± 2.4	39.7 ± 2.7
**D34**	R^1^ = 2,5-diOCH_3_; R^2^ = 4-F	46.9 ± 2.4	39.9 ± 3.7	36.0 ± 1.6
**D35**	R^1^ = 2-OCH_3_; R^2^ = 3-CF_3_	46.6 ± 2.0	37.6 ± 1.8	39.9 ± 2.8
**D36**	R^1^ = 4-OCH_3_; R^2^ = 3-CF_3_	55.1 ± 2.8	36.8 ± 2.2	33.9 ± 4.1
**D37**	R^1^ = 2,4-diOCH_3_; R^2^ = 3-CF_3_	43.8 ± 3.2	38.2 ± 2.2	29.8 ± 2.7
**D38**	R^1^ = 2,4-diOCH_3_; R^2^ = 4-CF_3_	33.3 ± 3.1	36.7 ± 3.5	28.0 ± 4.0
NNM^b^	/	83.3 ± 1.2	49.0 ± 3.9	49.1 ± 1.8
RBV^c^	/	59.3 ± 3.8	41.9 ± 2.7	44.2 ± 3.1

a Average of three replicates.

b NNM was used as control.

c RBV was used as control.

**Table 2:** EC_50_ Values of some Products anti-PVY inactivation activity *in vivo*

Compd.		Inactivating effect EC_50_ (*μ*g/mL)^a^
**D1**	R^1^ = 4-OCH_3_; R^2^ = 4-Cl	307.2 ± 13.7
**D13**	R^1^ = 4-OCH_3_; R^2^ = 4-Br	79.5 ± 3.3
**D17**	R^1^ = 2-OCH_3_; R^2^ = 4-Cl	141.7 ± 15.2
**D24**	R^1^ = 2,3-diOCH_3_; R^2^ = 4-Cl	147.3 ± 11.0
NNM^b^	/	54.3 ± 2.2
RBV^c^	/	213.1 ± 15.4

a Average of three replicates.

b NNM was used as control.

c RBV was used as control.

### Molecular docking

**Figure 4:**
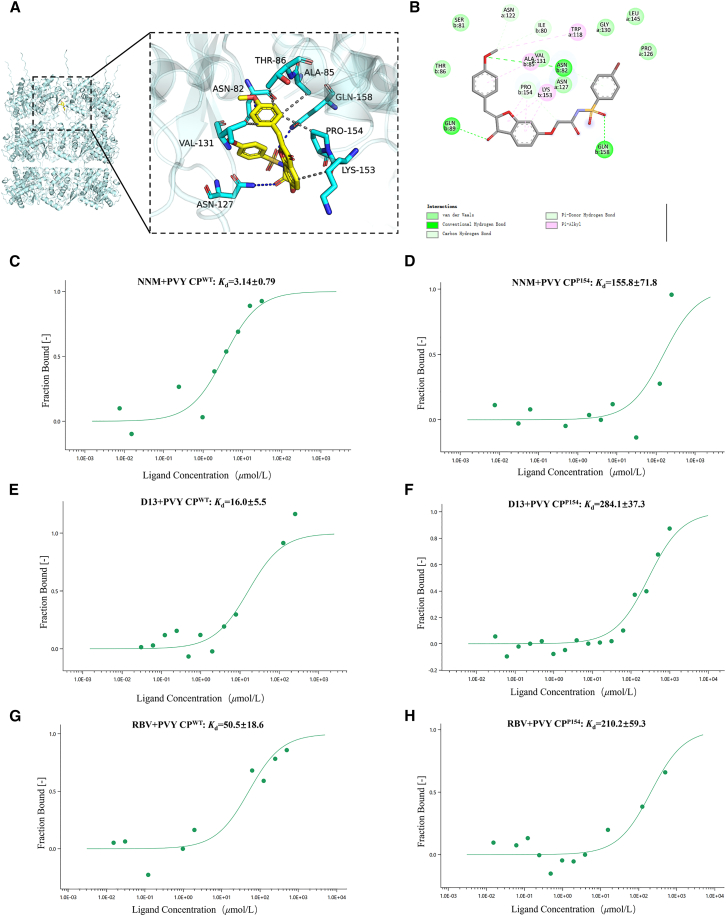
P154 in PVY CP is a key binding site for PVY CP (A) Three-dimensional view of molecular docking of **D13**. (B) Planar view of molecular docking of **D13**. (C–H) MST results of PVY CP and mutant protein PVY CP^P154A^ with compound **D13**, RBV and NNM.

Molecular docking simulation, as an efficient and convenient computational tool, can accurately reveal small molecule-target protein interactions, thereby providing strong support for drug design and biomolecular research. In this study, we used Vina 1.2.3 software to dock compound **D13** with PVY CP to investigate whether there is an interaction between compound **D13** and the CP protein. The docking results showed that the small molecule **D13** fits deeply into the active pocket formed by amino acid residues such as proline at position 154 of PVY CP. The establishment of hydrogen bonds guarantees a strong interaction between the protein and the small molecule. Additionally, hydrophobic interactions between **D13** and residues ASN-82, ASN-127, and GLN-158 provide strong van der Waals forces. The docking software calculated a binding affinity score of −8.473 kcal/mol for the **D13**-PVY CP complex. A negative binding affinity indicates the potential for binding, with lower values typically suggesting stronger binding potential. This suggests that **D13** binds well with PVY CP. The molecular docking experiment preliminarily identified P154 as a potential target site for the interaction of compound **D13** with PVY CP (Figures 4A and 4B).

### MST was used to verify the binding of small molecule drugs to proteins

CP is essential for controlling viral gene expression and the formation of viral particles, exhibiting virus-specific interactions that can influence the assembly and accumulation of viral particles.^39^^,^^40^ We constructed the pET-PVY CP and pET-PVY CP^P154A^ plasmids using pET-32a (+) as the vector by mutating the 154th Pro residue to alanine (Ala) in PVY CP. Subsequently, we obtained the wild-type His-PVY CP and mutant His-PVY CP^P154A^ proteins through prokaryotic expression and purification. 12% SDS-PAGE electrophoresis results showed that a distinct band around 50 kDa was observed; the band position of the target protein is consistent with the position predicted by its theoretical molecular weight. After digestion with enterokinase and removal of the His-tag, a specific band at approximately 33 kDa was observed, indicating successful protein expression and purification. The purification results of wild-type PVY CP^wt^ and mutant PVY CP^P154A^ proteins are shown in Figure S3.

Affinity, an important parameter describing the binding capacity of a drug molecule to its target, is typically characterized by the dissociation constant (*K*_d_). This constant quantifies the strength of the interaction between the drug molecule and its target. It has a long history of nearly a century of application and is widely used to predict the pharmacological responses of drugs.^41^ MST, as a powerful biomolecular interaction analysis technique, quantifies binding events by detecting the movement of molecules in a microscopic temperature gradient, demonstrating significant advantages. Compared to traditional methods, MST offers several advantages, such as ease of operation, no need for sample immobilization, measurement in free solution, low sample consumption, and efficient data acquisition. These characteristics make it a promising technique for high-throughput screening and rapid quantitative analysis.^42^ The experimental results indicate that mutation of proline at position 154 in PVY CP to Ala significantly reduces the binding affinity of the mutant PVY CP^P154A^ protein compared to the wild-type PVY CP. The *K*_d_ value of compound **D13** for PVY CP was determined to be 16.0 *μ*M, whereas the *K*_d_ for the P154A mutant rose to 284.1 *μ*M. Comparable shifts in *K*_d_ were observed for NNM and RBV, confirming that **D13** binds PVY CP with a markedly higher affinity than its P154A variant. Relative to the reference compounds, **D13** exhibits stronger binding than RBV (*K*_d_ = 50.5 *μ*M) but slightly weaker than NNM (*K*_d_ = 3.14 *μ*M) (Figures 4C–4H), a ranking that mirrors their respective anti-PVY activities. This finding further confirms that P154 of the PVY CP constitutes the critical binding site for aurone derivatives bearing the sulfonamide pharmacophore.

### The inhibitory effect of compound D13 on the systemic infection of PVY

**Figure 5:**
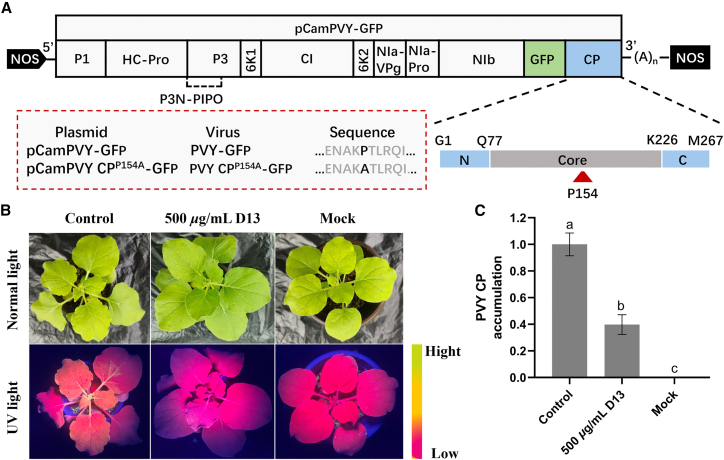
Effect of compound **D13** on PVY infection (A) Schematic diagram of the pCamPVY-GFP genomic structure. P154 is indicated by red arrowheads, located in the core region of PVY CP. The site-directed mutagenized and wild-type plasmids, viruses, and sequences are shown in the red-lined box. (B) Green fluorescence expression map of *N. benthamiana* leaf blades treated with PVY-GFP infectious **D13** and DMSO under UV illumination. 25 mm equivalent focal length, camera height ≈25 cm, field of view ≈6.5 cm × 8.7 cm. (C) Quantitative evaluation of PVY CP accumulation: the accumulation levels of PVY CP were determined by RT-qPCR in systemically infected leaves of *N. benthamiana* plants. After inoculation with *Agrobacterium*-carrying pCamPVY-GFP, leaves were sprayed with 1% Tween 80 solution containing **D13** (500 *μ*g/mL) and DMSO (control), respectively. Actin was used as an internal reference for data normalization. The results are shown as the mean ± standard deviation (SD) for each treatment group, and three biological replicates were included. Different letters indicate statistically significant differences (*p* < 0.05, one-way ANOVA).

To investigate the effect of compound **D13** on PVY system infection, we inoculated *Nicotiana benthamiana* (*N. benthamiana*) leaves with *Agrobacterium* carrying the wild-type PVY-GFP. After 24 h, the infiltrated leaves were treated with dimethyl sulfoxide (DMSO) as the negative control group and 500 *μ*g/mL **D13** as the treatment group. As shown in Figure 5B, it was found that on the seven days post-inoculation (dpi), the DMSO control group showed strong green fluorescence in stems and young top leaves, while in the 500 *μ*g/mL **D13** treatment group, green fluorescence was almost unobservable. The results of reverse transcription quantitative real-time PCR (RT-qPCR) showed that the accumulation level of PVY RNA in systemic leaves of *N. benthamiana* treated with 500 *μ*g/mL **D13** was significantly reduced to 39.8% compared with the DMSO control group defined as 100% (Figure 5C). To further confirm reproducibility, an additional independent biological replicate (Figure S4) was uploaded, and the observed trend is consistent with the primary dataset. These findings demonstrate that compound **D13** markedly reduces PVY accumulation and effectively suppresses systemic infection of PVY-GFP in *N. benthamiana*, positioning it as a promising antiviral agrochemical candidate.

### Effects of mutation at position P154 of CP on PVY accumulation levels

**Figure 6:**
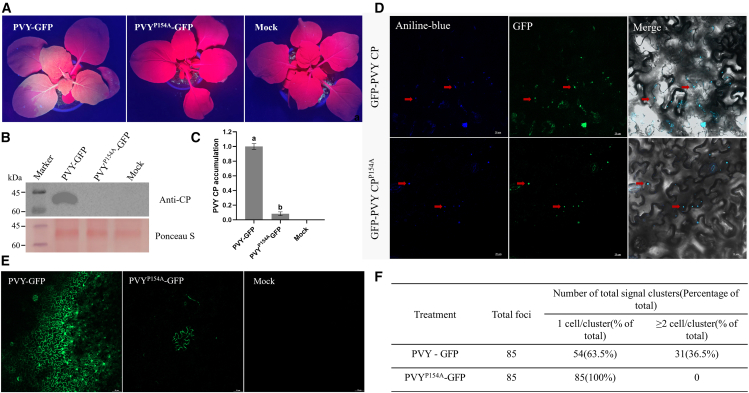
The effect of P154 mutation on PVY infection (A) Symptoms under UV illumination in *N. benthamiana* plants inoculated with wild-type or mutant PVY. 25 mm equivalent focal length, camera height ≈25 cm, field of view ≈6.5 cm × 8.7 cm. (B and C) Accumulation level of PVY CP in systemically infected leaves: in *N. benthamiana* plants, the accumulation level of PVY CP in systemically infected leaves infected with wild-type and mutant PVY was analyzed by WB and RT-qPCR techniques at seven dpi. Among them, RT-qPCR was normalized with actin as an internal reference, and WB used Ponceau S staining as a reference for sample loading, the 50 kDa band corresponds to PVY CP, molecular weight markers are shown on the left. The experimental data are presented as the mean ± SD of each treatment group, including three biological replicates. Different letters indicate statistically significant differences (*p* < 0.05, one-way ANOVA). (D) On the five dpi, images were taken using a confocal microscope to observe the effect of P154 mutation on the subcellular localization of PVY CP. Scale bars represent 20 *μ*m. (E) The role of P154 in PVY cell-to-cell movement. Scale bars represent 50 *μ*m. (F) The cell-to-cell movement efficiency of wild-type and mutated PVY-infected on day five.

To further validate the potential of P154 in CP as a binding site for the small molecule **D13** *in vivo*, we introduced a site-directed mutation at codon 154 of CP based on the infectious clone pCamPVY-GFP, generating the pCamPVY^P154A^-GFP plasmid (Figure 5A). *Agrobacterium* containing either pCamPVY-GFP or pCamPVY^P154A^-GFP was introduced into fully expanded leaves of four to six-week-old *N. benthamiana* plants. Seven dpi, intense green fluorescence was detected in the systemic leaves of plants infiltrated with pCamPVY-GFP under ultraviolet light. Nevertheless, no green fluorescence was detected in the pCamPVY^P154A^-GFP infiltrated plants (Figure 6A). To rule out the possibility that the mutant was perceived as completely non-infectious simply because of an early failure, we re-imaged whole *N. benthamiana* plants inoculated with the wild-type and the mutant at 13 dpi (Figure S5). Western blotting (WB) and RT-qPCR were employed to assess the impact of the P154 residue on viral accumulation in systemic leaves at both the protein and transcript levels. WB revealed a pronounced and readily detectable PVY CP-specific band in wild-type PVY-GFP-infected plants, whereas this band was markedly attenuated and almost undetectable in PVY^P154A^-GFP-infected plants (Figure 6B). Consistently, RT-qPCR analysis demonstrated that PVY CP accumulation in systemic leaves was significantly higher in the wild-type PVY-GFP group than in the PVY^P154A^-GFP group (Figure 6C). These results indicate that mutation of P154 in CP significantly affects viral accumulation in *N. benthamiana*, and the P154 mutation in PVY^P154A^-GFP is crucial for systemic infection of the virus in plants.

### The effect of P154 on subcellular localization of PVY CP

CP is essential for the movement and systemic infection of PVY in plants, as PVY spread through PD between plant cells.^43^ Previous studies have established that the PD targeting of CP is intimately linked to its role in mediating long-distance virus movement. Kozieł et al. employed immunogold double labeling in cucumber leaves to demonstrate that both the CP and movement protein of *Prune dwarf virus* (PDV) co-localize within PD-associated tubules, whereas CP is also found dispersed in the cytoplasm, indicating that CP serves dual functions in virion assembly and PD targeting.^44^ Building on this, we investigated whether residue P154 influences the subcellular localization of PVY CP-particularly its ability to accumulate at PD-by introducing *Agrobacterium* strains carrying pCamGFP-PVY CP or pCamGFP-PVY CP^P154A^ into *N. benthamiana* leaves for transient expression. On day five, the infiltrated leaves were stained with aniline blue for 10−15 min (to label the callose and show PD localization) and analyzed using confocal microscopy.^45^^,^^46^ The findings indicated that both GFP-PVY and GFP-PVY CP^P154A^ exhibited fluorescence patterns at the cell periphery and co-localized with the aniline blue-stained callose. This observation indicates that, despite the P154 substitution, both GFP-PVY CP and GFP-PVY CP^P154A^ retain the ability to localize efficiently at the cell periphery (Figure 6D). These findings provide evidence that GFP-PVY and GFP-PVY CP^P154A^ retain functional ability for intercellular movement.

### The role of P154 in the intercellular movement of PVY

To evaluate the effect of P154 on PVY intercellular movement, *Agrobacterium* strains carrying PVY-GFP or PVY^P154A^-GFP were infiltrated into *N*. *benthamiana* leaves. At five dpi, confocal laser-scanning microscopy revealed that the wild-type PVY-GFP had spread beyond the initially infected cells, with GFP fluorescence detected in adjacent cells (Figure 6E). Quantitative analysis showed that the cell-to-cell movement efficiency of wild-type PVY-GFP reached 63.5% (Figure 6F), indicating efficient intercellular spread under normal conditions. In contrast, no cell-to-cell movement was observed in leaves expressing PVY^P154A^-GFP; GFP fluorescence remained confined to single cells. These results demonstrate that the P154A mutation markedly impairs PVY intercellular movement, underscoring the critical role of P154 in this process.

Early GFP fluorescence intensities were further quantified at three dpi. No significant difference was observed between the wild type and the P154A mutant within single cells (Figure S2), indicating that the impaired intercellular movement of the P154A mutant during early infection is not attributable to reduced viral replication. These data corroborate that the P154 residue governs PVY systemic spread by modulating intercellular movement rather than replication.

In conclusion, this study synthesized a series of plant virus inhibitors based on aurones that exhibited strong inhibition of PVY infection. Among the target compounds, compound **D13** demonstrated better antiviral activity compared to the commercial control drug RBV. Molecular docking results indicated that P154 is a potential key binding site for the interaction of the small molecule with PVY CP, which was further confirmed by MST experiments. Additionally, experiments such as WB and RT-qPCR showed that compound **D13** effectively suppressed PVY-GFP systemic infection in *N. benthamiana*, with P154 playing an essential role in the virus’s movement from cell to cell. By inhibiting the cell-to-cell spread of PVY, compound **D13** significantly reduced the virus’s pathogenicity. Therefore, the compound **D13** may act as a potential inhibitor of intercellular motility. This work lays the foundation for the development of novel antiviral aurone derivatives and further expands the potential for developing intercellular movement inhibitors targeting virus CPs. This strategy provides new ideas and directions for research into antiviral drugs for plant virus.

### Limitations of the study

In this study, we identified a novel compound **D13** with significant antiviral activity through rational design, synthesis, and bioactivity evaluation. Using a suite of techniques including molecular docking, MST, WB, RT-qPCR, *Agrobacterium*-mediated infection, and confocal laser microscopy, we innovatively revealed that **D13** likely targets the P154 residue of the CP to affect viral intercellular movement. Although **D13** exhibited good inactivation activity against PVY, its antiviral efficacy has only been tested against PVY, and no assessments have been conducted on other viruses. The specific regulatory mechanisms of **D13** remain unclear. In particular, how **D13** blocks viral intercellular movement, a key pathogenic behavior, and whether this process involves interacting proteins, are still unknown.

## Resource availability

### Lead contact

Further information and requests for resources and reagents should be directed to and will be fulfilled by the lead contact, Yanju Wang (18985900557@163.com).

### Materials availability

pET32a (+) -PVY CP^wt^, pET32a (+) -PVY CP^P154A^ clones, and all necessary resources will be promptly provided to ensure accessibility and support for your endeavors.

### Data and code availability

• All requested data are shared promptly upon receipt of a request.
• This paper does not report original code.
• Additional details necessary for re-examination of the data presented in this article can be sourced from the lead contact upon submission of a request.

## Acknowledgments

The authors are grateful to the Scientific Research Innovation Team of Guizhou University (No. 202403).

## Author contributions

D.C.: conceptualization, writing the original draft and editing, investigation, data curation, and formal analysis. Q.M.: formal analysis and conceptualization. X.L.: software application and conceptualization. T.Y.: investigation and formal analysis. S.W.: investigation and formal analysis. Y.W. and D.H.: writing review & editing, supervision, project administration, funding acquisition. All authors have read and reviewed the manuscript.

## Declaration of interests

The authors declare no competing interests.

## STAR★Methods

### Key resources table

**Table:** 

REAGENT or RESOURCE	SOURCE	IDENTIFIER
**Antibodies**		

anti-GFP	Proteintech	Cat# 66002-1-Ig; RRID: AB_11182611
anti- PVY CP	Youke	Custom synthesis, http://www.youke-ab.cn/
Horseradish peroxidase-conjugated goat anti-rabbit IgG	Proteintech	Cat# SA00001-2; RRID: AB_2722564

**Bacterial and virus strains**		

*Escherichia coli* DH5*α* Competent Cells	Sangon Biotech	Cat# B528413-0010
*Escherichia coli* BL21 (DE3) Competent Cells	Sangon Biotech	Cat# B528414-0010
*Agrobacterium* GV3101 Competent Cells	Sangon Biotech	Cat# B528430-0010
Potato virus Y	The laboratory preservation	N/A

**Biological samples**		

*Nicotiana benthamiana* seeds	The laboratory preservation	N/A
*Nicotiana tabacum* K326 seeds	The laboratory preservation	N/A
*Chenopodium amaranticolor* seeds	The laboratory preservation	N/A

**Chemicals, peptides, and recombinant proteins**		

6-Hydroxybenzofuran-3(2*H*)-one	Energy Chemical	Cas. 6272-26-0
Bromoacetyl bromide	Energy Chemical	Cas: 598-21-0
Kanamycin Monosulfate	Sangon Biotech	Cas: A506636-0025
Ampicillin sodium	Sangon Biotech	Cas: A610028-0025
Acetosyringone	Sangon Biotech	Cas: A601111-0001
MES monohydrate	Sangon Biotech	Cas: A100169-0025
*N*, *N*-Dimethylformamide	Energy Chemical	Cas: 68-12-2
DMSO-*d*_6_	Energy Chemical	Cas: 2206-27-1
Ningnanmycin	Bide Pharmatech	Cas: 156410-09-2
Ribavirin	Aladdin	Cas: 36791-04-5
PVY CP^P154A^	Manuscript	N/A

**Critical commercial assays**		

TureColor Tricolor predyed protein Marker	Sangon Biotech	Cat# C610010-0001
TIANprep Mini Plasmid Kit	TIANGEN	Cat# DP103-02
TGX Stain-Free™ FastCast™ Acrylamide Kit, 12%	Bio-rad	Cat# 161-0185
Trizol reagent	Invitrogen	Cat# 15596026
HiScript III 1st Strand cDNA Synthesis Kit (+gDNA wiper)	Vazyme	Cat# R312-01
TB Green Ⅱ	TaKaRa	Cat# R820A
Monolith RED-NHS second generation protein labeling kit	NanoTemper	Cat# MO-L011

**Deposited data**		

*Potato Virus Y* coat protein structure	Protein Data Bank	PDB: 6HXZ

**Oligonucleotides**		

primer sequences	Manuscript	See supplemental information (Table S1)

**Recombinant DNA**		

pET-32a(+) PVY CP^wt^ prokaryotic expression plasmid	The laboratory preservation	N/A
pET-32a(+) PVY CP^P154A^ prokaryotic expression plasmid	Sangon Biotech	Custom synthesis, https://www.sangon.com

**Software and algorithms**		

ÄKTA go™ protein purification system	Cytiva	https://www.cytivalifesciences.com/en/us
GraphPad Prism 8	Dotmatics	https://www.graphpad.com/
ChemDraw 20.0	Revvity Signals	https://revvitysignals.com/
NanoTemper Monolith Instrument (NT.115) Control and Analysis Software Package	NanoTemper	https://nanotempertech.com/microscale-thermophoresis/
MestReNova	Mestrelab Research	https://www.mestrelabcn.com/

### Method details

#### Materials and instruments

6-Hydroxybenzofuran-3(2*H*)-one (purity 98%; CAS: 6272-26-0), bromoacetyl bromide, substituted sulfonamides, and other reagents were purchased from Aladdin (Shanghai, China) or Energy Chemical (Shanghai, China). The solvents used were reagent-grade and applied without additional purification steps. *Nicotiana tabacum* K326, *N. benthamiana*, and *Chenopodium amaranticolor* were all grown in a greenhouse with stable light/dark cycles and controlled temperature conditions. This controlled environment minimized the influence of external factors on plant growth.

The ^1^H, ^13^C, and ^19^F NMR spectra of the target compounds were recorded in DMSO-*d*_6_ using Bruker AG 400 MHz and JEOL ECX 500 MHz NMR instruments. High-resolution mass spectrometry (HRMS) analysis was conducted using a Thermo Scientific Q Exactive mass spectrometer (Massachusetts, USA) to determine the molecular weight of the compounds. The melting points of the compounds were measured with an SGWX-4B micro melting point apparatus. The AKTA go system (Cytiva, Massachusetts) was used to purify the wild-type and mutant proteins. Fluorescence-labeled images were observed using a confocal microscope (Carl Zeiss, Germany).

#### General procedure for the target compounds D1−D38

Intermediates **2a−2i** were synthesized with slight modifications based on previously reported methods.^47^ Different substituted benzaldehydes (3.00 mmol) were introduced into a solution of 6-hydroxybenzofuran-3(2*H*)-one (2.00 mmol) in ethanol (30 mL), followed by the addition of 12 M HCl (3 mL). A distinct color change was observed upon mixing. Heated and stirred at 60**−**70°C for 6 hours. After the reaction is completed, the mixed system is poured into water, and after the solid is precipitated, the precipitate is filtered and vacuum dried to separate, and subsequently purified to afford intermediates **2a−2i**.

Intermediates **4a−4f** were synthesized with slight modifications based on previously reported methods.^48^ Under a nitrogen atmosphere, a three-neck flask was charged with substituted benzenesulfonamides (2.00 mmol) and anhydrous toluene (5 mL). The reaction mixture was then stirred and heated to 110°C until complete dissolution was observed. Once the temperature reached 110°C, it was maintained for 10 minutes before slowly adding bromoacetyl bromide (7.21 mmol) dropwise via a syringe. A color change was observed upon addition, and the reaction was continuously heated at 110°C in toluene for an additional 5 hours. Upon completion, after cooling to room temperature and then kept at -20°C overnight to minimize disturbance to product crystallization. The obtained solid was separated by vacuum filtration and washed multiple times with cold toluene until no further color change was observed. Ultimately, the purified product was dried at 60°C for 24 hours, affording intermediates **4a−4f**.

The target compounds **D1−D38** were synthesized based on a previously reported method with slight modifications.^49^ Taking compound **D13** as an example, (*Z*)-6-hydroxy-2-(4-methoxybenzylidene) benzofuran-3(2*H*)-one (3.35 mmol), 2-bromo-*N*-((4-bromophenyl) sulfonyl) acetamide (6.70 mmol), potassium carbonate (0.27 mmol), and DMF (5 mL) were stirred at room temperature overnight for 18−24 hours. After the reaction was complete, the reaction mixture was poured into water (100 mL). The organic layer was extracted with ethyl acetate (4 × 100 mL). The organic layer was dried with anhydrous Na_2_SO_4_ (s) and then concentrated. Compound **D13** was purified by column chromatography using a mixture of petroleum ether and ethyl acetate (petroleum ether: ethyl acetate = 2:1) as the eluent, yielding the final product as a yellow solid. (*Z*)-*N*-((4-bromophenyl)sulfonyl)-2-((2-(4-methoxybenzylidene)-3-oxo-2,3-dihydrobenzofuran-6-yl)oxy)acetamide **(D13)**: Yield: 82.34%; yellow solid; m. p. 223.8–224.7°C; ^1^H NMR (400 MHz, DMSO-*d*_6_): *δ* 7.99 – 7.91 (m, 2H, Ar-H), 7.89 – 7.79 (m, 4H, Ar-H), 7.67 (d, *J* = 8.6 Hz, 1H, ꞊CH-), 7.13 – 7.07 (m, 2H, Ar-H), 6.98 (d, *J* = 2.1 Hz, 1H, Ar-H), 6.86 (s, 1H, Ar-H), 6.80 (dd, *J* = 8.6, 2.2 Hz, 1H, Ar-H), 4.86 (s, 2H, -CH_2_-), 3.84 (s, 3H, -CH_3_). ^13^C NMR (101 MHz, DMSO-*d*_*6*_): *δ* 181.89, 167.77, 167.70, 165.74, 161.13, 146.44, 139.52, 133.63, 132.56, 130.00, 127.88, 125.85, 124.95, 115.15, 115.10, 113.21, 112.15, 98.18, 67.16, 55.87. HRMS (ESI): m/z for C_24_H_17_BrNO_7_S [M-H] ^-^ calcd 541.99036 found 541.99121.

#### Antiviral bioassays

Owing to its non-host resistance to PVY, *Chenopodium amaranticolor* develops a hypersensitive response (HR) within 48–72 hours post-inoculation, producing sharply delimited chlorotic or necrotic local lesions on the inoculated leaves. The number of lesions correlates linearly and positively with virus titre, thereby serving as a quantitative indicator of viral virulence and of the inactivating efficacy of test compounds. The PVY viral extract was rubbed onto the surface of *Nicotiana tabacum* K326 leaves. After the characteristic symptoms of leaf wrinkling and inward curling appeared, the leaves were harvested and stored at -80°C, then purified according to previously reported methods.^50^^,^^51^^,^^52^ Host leaves were ground to a fine powder in liquid nitrogen, followed by homogenization on ice in 0.5 M phosphate buffer. An equal volume of chloroform:n-butanol (1:1, v/v) corresponding to 10% of the total buffer volume was added, and the mixture was further homogenized on ice. After centrifugation at 10,000g for 20 minutes (4°C), the supernatant was collected as the crude PVY extract. Using 4–5-leaf-stage *Chenopodium amaranticolor* maintained under optimal growth conditions, a bioassay was conducted with two plants per treatment to systematically evaluate the curative, protective, and inactivating efficacy of the target compound against PVY.^53^ On this basis, the inactivation activity of selected compounds with better inactivation efficacy against PVY was further evaluated at concentrations of 31.25, 62.5, 125, 250, and 500 *μ*g/mL, and their EC_50_ values were calculated. Each compound was evaluated in three replicates, and the average value was calculated.

##### Anti-PVY curative activity

*Chenopodium amaranticolor* was selected as the dead spot host. Excess leaves were first trimmed off, and a uniform layer of carborundum was applied to the leaf surface. The leaves were then inoculated by rubbing with a 1200-fold diluted virus solution. After more than an hour, the carborundum on the leaf surface was washed off with running water, and a 500 *μ*g/mL drug solution was prepared. Once the leaves had dried under natural conditions, the drug solution was applied to the right side of the leaves using a brush, while the left side was treated with a 1% Tween 80 solution as a control. The treated plants were cultivated in a greenhouse for observation, and the number of dead spots on the leaves was counted after approximately 5 days.

##### Anti-PVY protective activity

In contrast to the antiviral therapeutic activity, the leaves were first treated with the drug solution. A 500 *μ*g/mL drug solution was applied to the right side of the leaves, with the left side serving as a control and treated with a 1% Tween 80 solution. After the leaves had dried under natural conditions, they were placed in a greenhouse for 24 hours before inoculation by rubbing with carborundum, following the same procedures as those for curative activity. After treatment, the plants were maintained in a greenhouse, and the number of local lesions on the inoculated leaves was recorded approximately 5 days.

##### Anti-PVY inactivating activity

Two different dilutions of the virus solution (600-fold and 1200-fold) were prepared, along with a 1000 *μ*g/mL drug solution. The drug solution was mixed with the 600-fold diluted virus solution on ice and incubated for 30 minutes. During the incubation period, the left side of the leaves was treated with a 1200-fold diluted virus solution. After the incubation was completed, the mixed system of drug and virus is inoculated on the right side of the leaf. After waiting for the infection to be completed, the carborundum on the leaf surface was washed off with running water, and the plants were then cultivated in a greenhouse. The number of dead spots on the leaves was counted after approximately 5 days.

#### Molecular docking

To further investigate the interaction mode of the target compound with PVY CP, molecular docking of compound **D13** was performed using AutoDock Vina 1.2.3 in this study. The PVY CP protein structure (PDB: 6HXZ) and the **D13** small molecule were obtained from the PDB and PUBCHEM databases, respectively, and were optimized using the MMFF94 force field. Prior to docking, PyMol was used to remove water molecules, salt ions, and small molecules, and a docking box was set to cover the entire protein structure. After converting the protein and small molecules to the PDBQT format, the exhaustiveness was set to 32 with default parameters for other settings. The results were visualized using PyMol, and the top-scoring binding conformations were selected for analysis.

#### Plasmid construction and site-directed mutagenesis

The full-length gene synthesis method was employed to clone the PVY CP encoding sequence into the expression vector pET-32a (+). The resulting plasmid was named pET-PVY CP.^54^ Based on pET-PVY CP, the codon at position 154 was altered to Ala. The resulting plasmid was named pET-PVY CP^P154A^. Using the infectious clone pCamPVY-GFP as a template, site-directed mutagenesis was performed to mutate proline at position 154 of the CP to Ala. The mutated plasmid was constructed using pCam0390 as the vector, resulting in the pCamPVY^P154A^-GFP plasmid.^55^ All constructed plasmids were sequenced to confirm their integrity. The primer information used in this study is detailed in Table S1.

#### *Agrobacterium*-mediated infiltration of PVY-GFP, agent treatment, and photographing

Wild-type or mutant viral plasmids were introduced into *agrobacterium* tumefaciens strain GV3101. Bacterial cultures were grown overnight at 28°C with shaking at 220 rpm, harvested by centrifugation at 8 minutes when OD_600_ reached 0.6–0.8, and the supernatant was discarded. The pellets were resuspended in infiltration buffer to the turbidity required by the specific experiment, incubated in darkness for 3 hours, and then infiltrated into appropriately aged *N*. *benthamiana* leaves. After 24 hours, the infiltrated leaves were treated with 500 *μ*g/mL compound **D13**, or DMSO (control). At 7 dpi, GFP fluorescence in leaves was visualized and photographed under 365 nm excitation using a LUYOR handheld UV lamp (LUYOR-3415RG, Luyor Instrument, Shanghai, China). After *Agrobacterium* infection, samples were taken from the same position of three sets of parallel samples and mixed. The samples were quickly frozen in liquid nitrogen and then kept at -80°C to maintain stability in preparation for subsequent experiments and analysis.

#### RNA extraction, cDNA synthesis and RT-qPCR

The samples of *N. benthamiana* infected with *Agrobacterium* were taken out from the -80°C freezer, small steel beads were added, and they were ground into fine powder with liquid nitrogen to ensure that the cells were completely broken and the nucleic acid components in the cells were released. Then, total RNA was isolated from *N. benthamiana* leaves using Trizol reagent (TaKaRa, China). RNA was reverse transcribed into cDNA using a reverse transcription kit (Vazyme, China) and specific primers, and then RT-qPCR analysis was performed using TB Green (TaKaRa, China). The primers used is recorded in Supporting Information.

#### The expression and purification of PVY CP and its mutants

The PVY CP coding sequence was successfully inserted into the expression vector pET-32a (+) to construct the recombinant plasmid pET-PVY CP. The plasmid was then introduced into *Escherichia coli* (*E. coli*) BL21 (DE_3_) pLysS competent cells and allowed to proliferate, protein expression was initiated by the inducer isopropyl *β*-D-1-thiogalactopyranoside (IPTG) to promote efficient protein expression under appropriate conditions. Cells were lysed by ultrasonic disruption to release intracellular proteins. Cell debris and insoluble substances were removed by high-speed centrifugation of the lysate, and the supernatant was collected for subsequent soluble protein extraction. The Ni-NTA column was used to specifically bind to PVY CP with a 6 × His tag and separate the target protein. Subsequently, the protein was digested with enterokinase to remove the affinity tag. Finally, the protein was identified by 12% SDS-PAGE electrophoresis. Building upon pET-PVY CP, the codon at position 154 was substituted with Ala. The plasmid was called pET-PVY CP^P154A^, and the expression and purification process of its mutant PVY CP^P154A^ protein was the same as that of the wild-type protein.

#### MST combined with fluorescence labeling bioassay method

The experiment was performed using a Monolith NT.115 instrument (NanoTemper, Germany), with specific procedures referenced from the literature.^56^^,^^57^ To improve the accuracy of small molecule drug concentrations, an initial concentration of 40.0 mM was prepared, which was then gradually diluted to a final concentration of 2.0 mM using a buffered solvent. After protein desalting, the protein was fluorescently labeled using the Monolith™ RED-NHS second-generation labeling kit. MST was used to measure the fluorescence intensity of the small molecule-protein complex, followed by interaction testing using Start MST Measurement. The binding strength was evaluated using the fluorescence quenching curve of the protein, and the experimental results were denoised, fitted, and normalized using specialized software to obtain accurate binding parameters.

#### Western blot assay

According to the description in previous literature,^58^ the total protein of *N*. *benthamiana* leaves infected with *Agrobacterium* was extracted and quantitatively analyzed. Based on consistent sample loading, density analysis of the bands in the colorimetric results was performed to assess the protein expression levels. Membranes were probed with either anti-GFP (Proteintech, China; 1:5,000 dilution) or anti-PVY CP (Youke, China; 1:4,000 dilution) primary antibodies in 5% skim-milk-TBST, incubated on a shaker at room temperature for 4–6 hours. After washing, horseradish peroxidase-conjugated goat anti-rabbit IgG (Proteintech, China) was applied as secondary antibody at 1:8,000 for 2 hours at room temperature to specifically detect the target proteins. After the antibody incubation, the target protein signal was developed using chemiluminescent developer, and the signal was imaged and analyzed using the Chemi Doc MP Imaging System (Bio-Rad, USA).

#### Confocal microscopy observation and image processing

In this study, we employed a confocal microscope (Carl Zeiss, Germany) to observe the intercellular movement of PVY-GFP and PVY^P154A^-GFP in *N. benthamiana* leaves at the subcellular level. Specifically, leaves infected with *Agrobacterium*-mediated PVY-GFP and PVY^P154A^-GFP were sectioned and mounted on slides. Fluorescence images were captured by adjusting the parameters of the laser confocal microscope. GFP fluorescence imaging parameters were set as follows: excitation, 488 nm; emission collection, 520–540 nm; frame resolution, 1024 × 1024 pixels; pixel size, 0.62 *μ*m; pixel dwell time, 8 *μ*s; line averaging, 2 ×. All fluorescence images were subsequently analyzed and processed using ZEN 2.1 software.

### Quantification and statistical analysis

For each experiment, n = 3 biologically independent replicates (each replicate corresponds to a treatment), expressed as mean ± SD. The central trend measure is the average, and the dispersion measure is SD. Statistical tests were performed using SPSS 26.0 (IBM, USA) software for one-way analysis of variance. The significant difference was represented by a specific letter at the top of the column, where p < 0.05 indicated statistical significance, and different letters meant statistically significant differences.

## Supplemental information

Document S1. Figures S1–S5, Table S1, and Data S2

Data S1. Unprocessed raw experimental images
