# Supplementary material for: Sulfonamine-containing aurone derivatives act as inhibitors of intercellular movement of potato virus Y
Source: iScience. 2025 Oct 30;28(12):113914. doi: 10.1016/j.isci.2025.113914 (PMC12663734; doi:10.1016/j.isci.2025.113914)
Supplement: Document S1. Figures S1–S5, Table S1, and Data S2 [file mmc1.pdf]

iScience, Volume *28*

Supplemental information

Sulfonamine-containing aurone derivatives act as inhibitors of intercellular movement of potato virus Y

Dan Chen, Qingqing Ma, Xin Li, Tao Yang, Shang Wu, Yanju Wang, and Deyu Hu

Supplemental figures

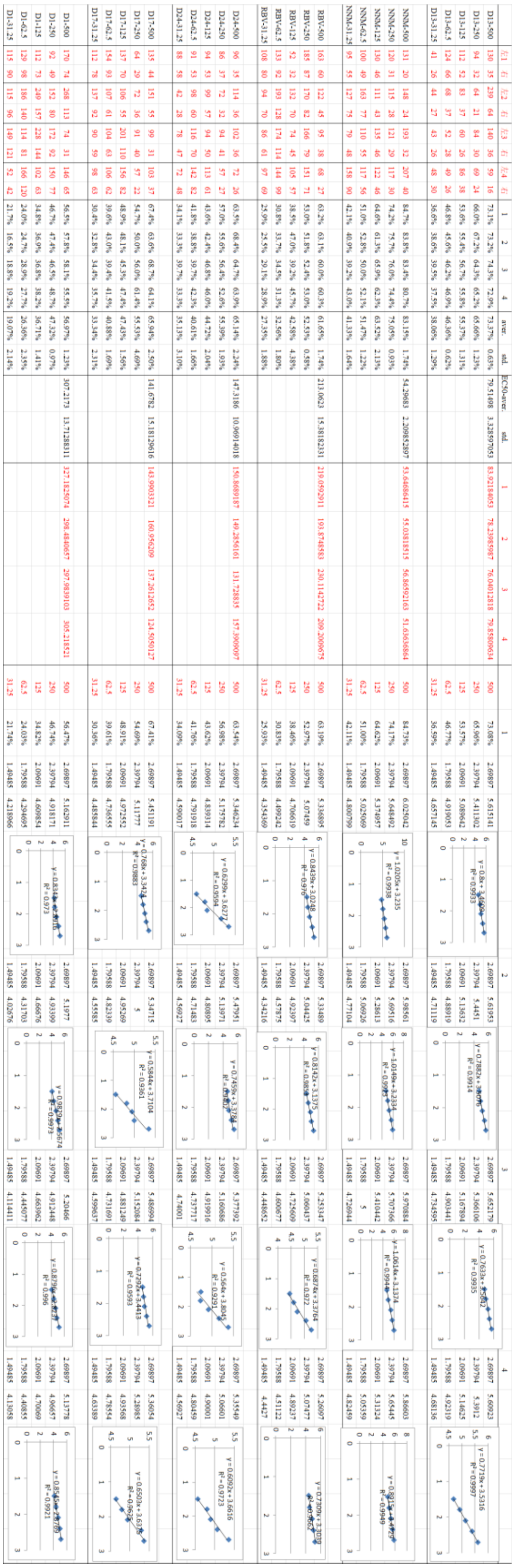

Figure S1. Raw data and corresponding dose–response curves for the EC_{50} values of the anti-PVY inactivation activities of compounds D13, NNM, and RBV.

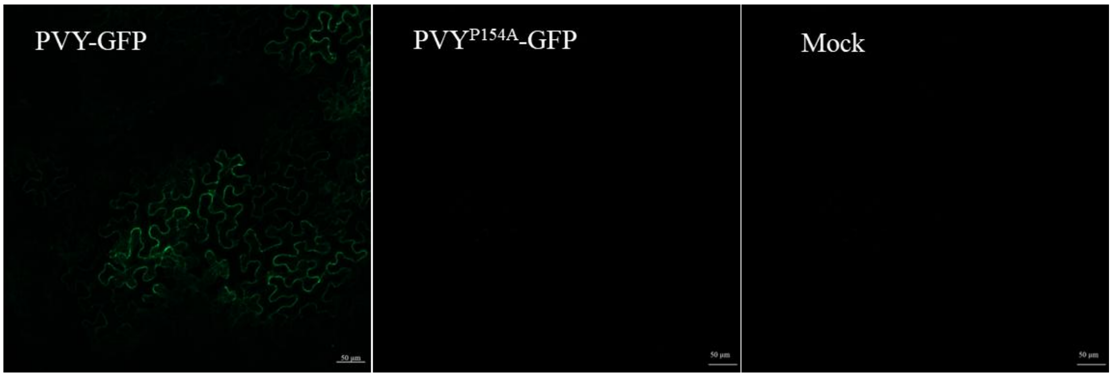

Figure S2. The intercellular movement of wild-type PVY-GFP and mutant PVY CP^{P154A}-GFP infected *N. benthamiana* plants on the third day.

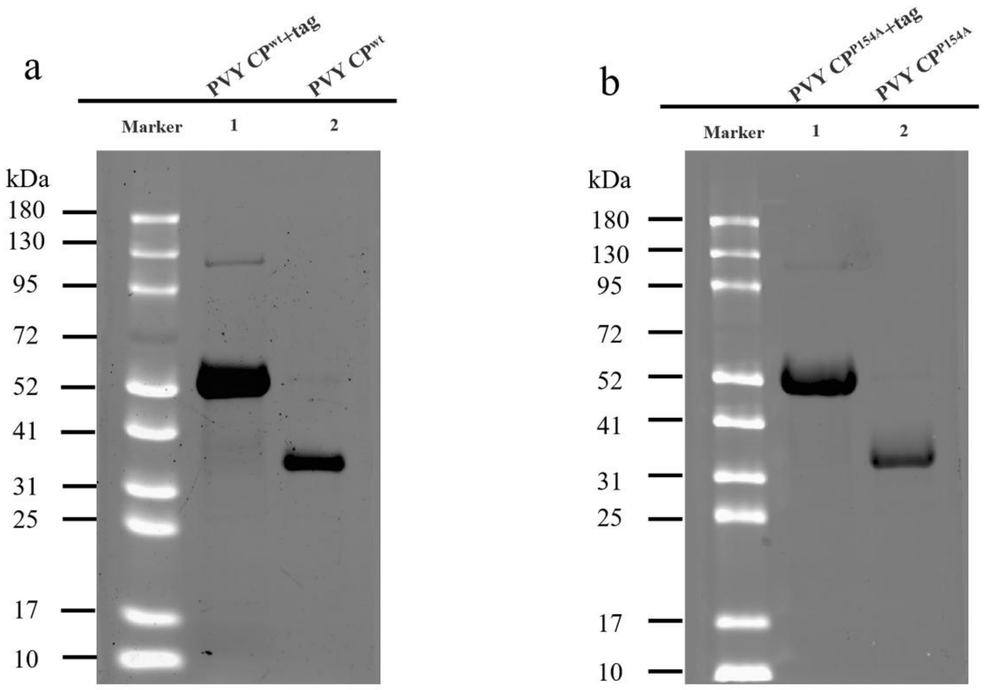

Figure S3. Purification results of wild-type PVY CP^{wt} and mutant PVY CP^{P154A} protein, respectively. PVY CP^{wt} and PVY CP^{P154A} with His-S-tag were observed in lane 1, PVY CP^{wt} and PVY CP^{P154A} after digestion and desalination were observed in lane 2.

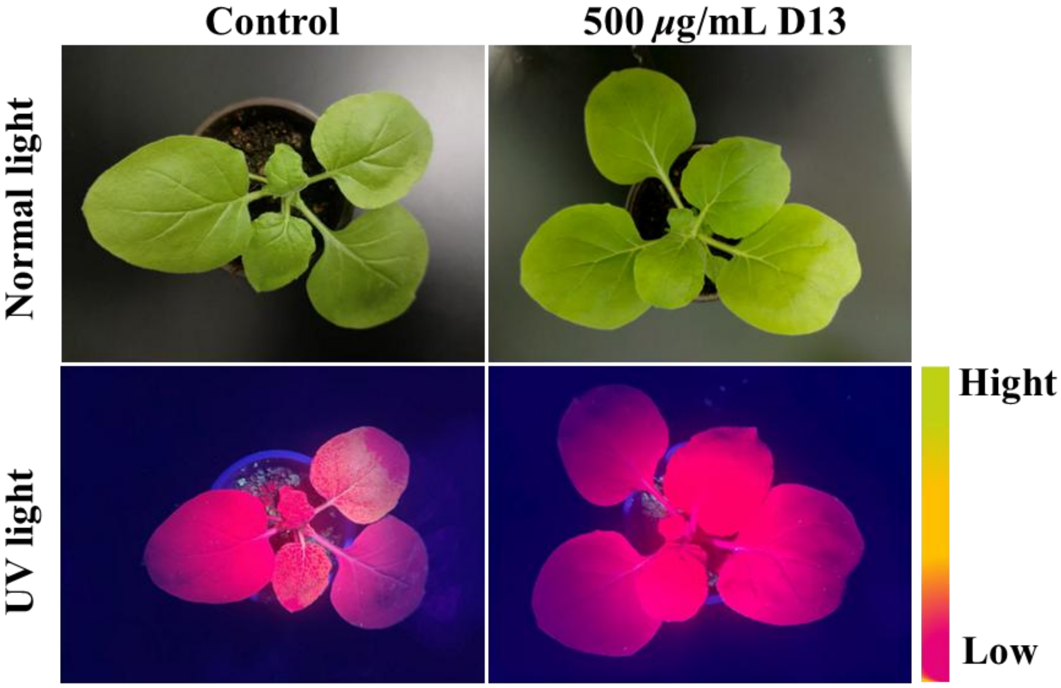

Figure S4. In another biological replicate, the green fluorescence distribution map of *N. benthamiana* leaves infected with PVY-GFP treated with D13 and DMSO under UV excitation.

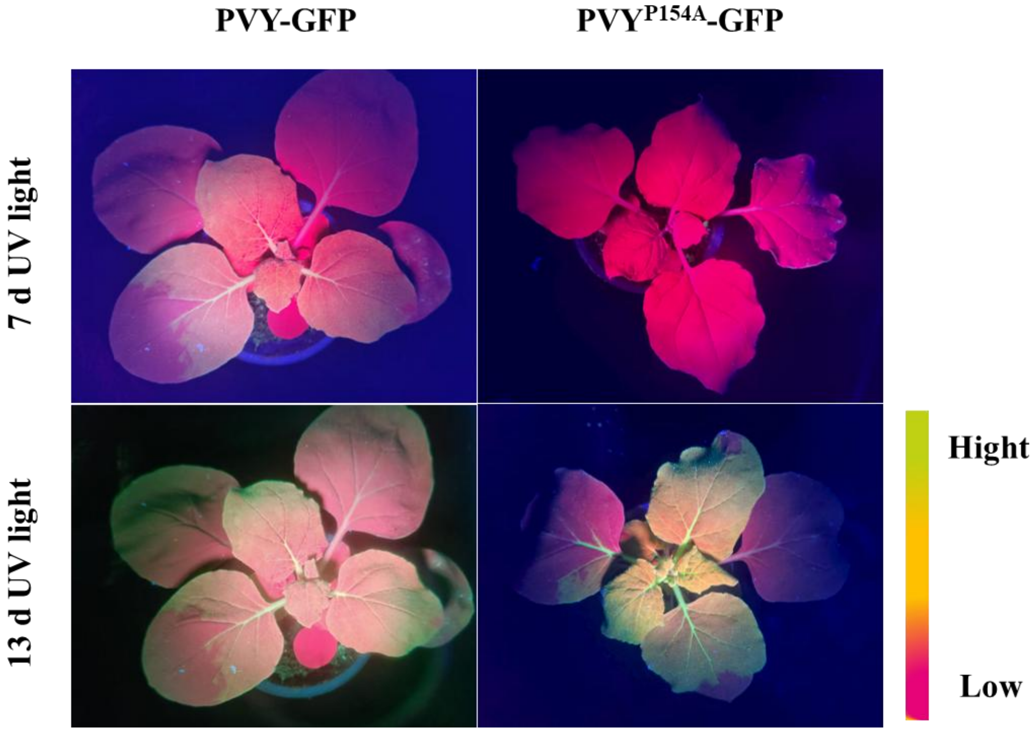

Figure S5. Symptoms of *N. benthamiana* plants inoculated with wild-type or mutant PVY under UV light on days 7 and 13.

Supplemental tables

Table S1. Primer sequences

Primer name	Sequence **5'→3'**
PVY- P154A-F	CAAAAGCAACCCTTAGGCAAATCATGGCACA
PVY- P154A-R	AAGGGTTGCTTTTGCATTCTCAACGATTGGTTTC
qPCR-PVY CP-F	TGGCGAGGTTCCATTTTCA
qPCR-PVY CP-R	CATAGGAGAAACTGAGATGCCAACT
qPCR-Actin-F	CACACTGGAGTGATGGTTGG
qPCR-Actin-R	GGTGTGGTGCCAAATCTTCT

Data S2. Characterization of target compounds D1–D38, related to Figure 2 and STAR Methods.

A) Spectral and physicochemical data ofthe product.

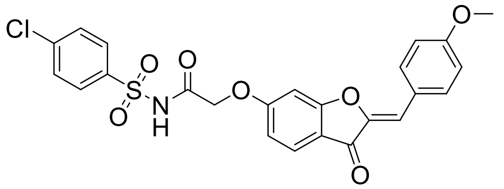

(*Z*)-*N*-((4-chlorophenyl)sulfonyl)-2-((2-(4-methoxybenzylidene)-3-oxo-2,3-dihydrobenzofuran-6-yl)oxy)acetamide (D1): Yield: 83.28%; buff solid; m. p. 221.9–223.4 °C; ^{1}H NMR (400 MHz, DMSO-d_6): δ 7.94 (dd, J = 8.8, 2.2 Hz, 4H, Ar-H), 7.71 – 7.65 (m, 3H, Ar-H), 7.09 (d, J = 8.8 Hz, 2H, Ar-H), 6.98 (d, J = 2.1 Hz, 1H, Ar-H), 6.86 (s, 1H, Ar-H), 6.79 (dd, J = 8.6, 2.1 Hz, 1H, =CH-), 4.87 (s, 2H, -CH_2-), 3.84 (s, 3H, -CH_3). ^{13}C NMR (101 MHz, DMSO-d_6): δ 181.89, 167.66, 167.44, 165.59, 161.12, 146.42, 139.17, 138.53, 133.61, 130.01, 129.73, 125.84, 124.91, 115.18, 115.10, 114.39, 112.18, 98.17, 66.94, 55.84. HRMS (ESI): m/z for $C_{24}H_{19}ClNO_7S$ $[M+H]^+$ calcd 500. 05653, found 500. 05579.

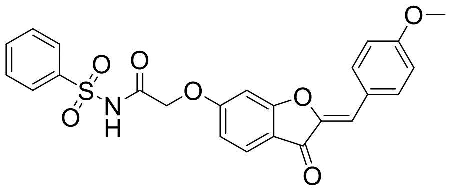

(*Z*)-2-((2-(4-methoxybenzylidene)-3-oxo-2,3-dihydrobenzofuran-6-yl)oxy)-*N*-(phenylsulfonyl)acetamide (D2): Yield: 79.11%; buff solid; m. p. 239.0–241.0 °C; ^{1}H NMR (400 MHz, DMSO-d_6): δ 12.72 (s, 1H, -NH-), 7.99 – 7.90 (m, 4H, Ar-H), 7.70 (d, J = 7.5 Hz, 1H, Ar-H), 7.70 – 7.63 (m, 2H, Ar-H), 7.62 (d, J = 1.7 Hz, 1H, Ar-H), 7.09 (d, J = 8.9 Hz, 2H, Ar-H), 6.96 (d, J = 2.1 Hz, 1H, Ar-H), 6.85 (s, 1H, Ar-H), 6.78 (dd, J = 8.6, 2.1 Hz, 1H, =CH-), 4.91 (s, 2H, -CH_2-), 3.83 (s, 3H, -CH_3). ^{13}C NMR (101 MHz, DMSO-d_6): δ 181.90, 167.66, 167.17, 165.59, 161.17, 146.40, 139.59, 134.29, 133.64, 129.64, 127.96, 125.87, 124.91, 115.18, 115.15, 113.17, 112.17, 98.20, 66.83, 55.88. HRMS (ESI): m/z for $C_{24}H_{20}NO_7S$ $[M+H]^+$ calcd 466.09550, found 466.09470.

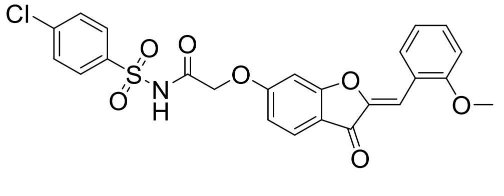

(*Z*)-*N*-((4-chlorophenyl)sulfonyl)-2-((2-(2-methoxybenzylidene)-3-oxo-2,3-dihydrobenzofuran-6-yl)oxy)acetamide (D3): Yield: 83.57%; buff solid; m. p. 225.9–226.8 °C; ^{1}H NMR (400 MHz, DMSO-d_6): δ 8.18 (d, J = 6.4 Hz, 1H, Ar-H), 7.73 (d, J = 8.5 Hz, 2H, Ar-H), 7.63 (d, J = 8.5 Hz, 1H, Ar-H), 7.48 – 7.43 (m, 1H, Ar-H), 7.40 (d, J = 8.5 Hz, 2H, Ar-H), 7.14 (d, J = 8.0 Hz, 2H, Ar-H), 7.09 (s, 1H, Ar-H), 6.77 (d, J = 2.1 Hz, 1H, Ar-H), 6.73 (d, J = 10.8 Hz, 1H, =CH-), 4.47 (s, 2H, -CH_2-), 3.91 (s, 3H, -CH_3). ^{13}C NMR (101 MHz, DMSO-d_6): δ 182.04, 167.93, 167.41, 165.81, 158.64, 147.57, 139.15, 138.56, 132.30, 131.46, 130.01, 129.78, 129.73, 126.00,

121.35, 120.55, 114.93, 113.30, 104.99, 98.31, 66.95, 56.29. HRMS (ESI): m/z for $C_{24}H_{19}ClNO_7S$ [M+H]$^+$ calcd 500.05653, found 500.05560.

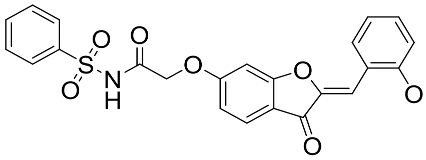

(*Z*)-2-((2-(2-methoxybenzylidene)-3-oxo-2,3-dihydrobenzofuran-6-yl)oxy)-*N*-(phenylsulfonyl)acetamide (D4): Yield: 87.19%; buff solid; m. p. 199.5–201.0 °C; ^{1}H NMR (500 MHz, DMSO-d_6): δ 12.64 (s, 1H, -NH-), 8.15 (dd, J = 7.6, 1.7 Hz, 1H, Ar-H), 7.98 – 7.91 (m, 2H, Ar-H), 7.71 – 7.63 (m, 2H, Ar-H), 7.62 (t, J = 7.7 Hz, 2H, Ar-H), 7.48 – 7.43 (m, 1H, Ar-H), 7.16 – 7.11 (m, 2H, Ar-H), 7.11 (s, 1H, Ar-H), 6.98 (d, J = 2.1 Hz, 1H, Ar-H), 6.79 (dd, J = 8.6, 2.1 Hz, 1H, =CH-), 4.89 (s, 2H, -CH_2-), 3.90 (s, 3H, -CH_3). ^{13}C NMR (101 MHz, DMSO-d_6): δ 182.05, 167.92, 167.13, 165.82, 158.67, 147.56, 139.63, 134.27, 132.38, 131.44, 129.64, 127.96, 126.03, 121.36, 120.52, 114.92, 113.33, 112.08, 104.95, 98.32, 66.86, 56.33. HRMS (ESI): m/z for $C_{24}H_{18}NO_7S$ [M-H]$^-$ calcd 464.07985, found 464.08011.

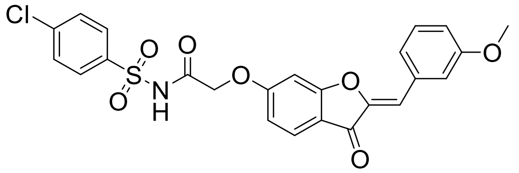

(*Z*)-*N*-((4-chlorophenyl)sulfonyl)-2-((2-(3-methoxybenzylidene)-3-oxo-2,3-dihydrobenzofuran-6-yl)oxy)acetamide (D5): Yield: 75.32%; buff solid; m. p. 206.4–208.1 °C; ^{1}H NMR (400 MHz, DMSO-d_6): δ 7.94 – 7.91 (m, 2H, Ar-H), 7.70 – 7.66 (m, 3H, Ar-H), 7.56 (d, J = 7.8 Hz, 1H, Ar-H), 7.53 (t, J = 2.1 Hz, 1H, Ar-H), 7.43 (t, J = 8.0 Hz, 1H, Ar-H), 7.03 (d, J = 2.2 Hz, 2H, Ar-H), 6.84 (s, 1H, Ar-H), 6.80 (dd, J = 8.6, 2.2 Hz, 1H, =CH-), 4.88 (s, 2H, -CH_2-), 3.82 (s, 3H, -CH_3). ^{13}C NMR (101 MHz, DMSO-d_6): δ 182.15, 168.06, 167.40, 165.93, 159.90, 147.71, 139.17, 138.55, 133.62, 130.51, 130.00, 129.73, 126.04, 124.05, 117.08, 115.89, 114.84, 113.36, 111.63, 98.37, 66.98, 55.65. HRMS (ESI): m/z for $C_{24}H_{20}NO_7S$ [M+H]$^+$ calcd 466.09550 found 466.09451.

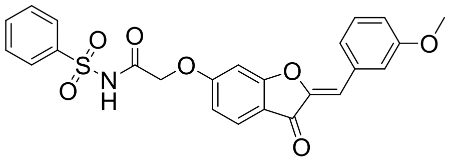

(*Z*)-2-((2-(3-methoxybenzylidene)-3-oxo-2,3-dihydrobenzofuran-6-yl)oxy)-*N*-(phenylsulfonyl)acetamide (D6): Yield: 80.21%; buff solid; m. p. 214.7–215.6 °C; ^{1}H NMR (400 MHz, DMSO-d_6): δ 12.63 (s, 1H, -NH-), 7.96 – 7.93 (m, 2H, Ar-H), 7.71 – 7.65 (m, 2H, Ar-H), 7.62 (dd, J = 8.4, 6.7 Hz, 2H, Ar-H), 7.58 – 7.52 (m, 2H, Ar-H), 7.44 (t, J = 7.9 Hz, 1H, Ar-H), 7.05 (dd, J = 8.2, 2.7 Hz, 1H, =CH-), 7.01 (d, J = 2.1 Hz, 1H, Ar-H), 6.84 (s, 1H, Ar-H), 6.80 (dd, J = 8.6, 2.1 Hz, 1H, Ar-H), 4.89 (s, 2H, -CH_2-), 3.82 (s, 3H, -CH_3). ^{13}C NMR (101 MHz, DMSO-d_6): δ 182.16, 168.05, 167.15, 165.92, 159.91, 147.69, 139.65, 134.27, 133.61, 130.52, 129.63, 127.95, 126.03, 124.06,

117.09, 115.90, 114.83, 113.39, 111.61, 98.35, 66.90, 55.67. HRMS (ESI): m/z for $C_{24}H_{19}ClNO_7S$ [M+H]$^+$ calcd 500.05653, found 500.05563.

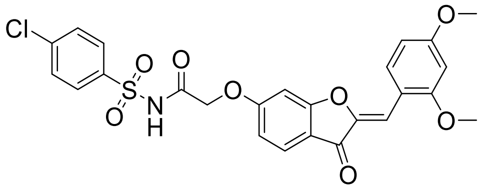

(*Z*)-*N*-((4-chlorophenyl)sulfonyl)-2-((2-(2,4-dimethoxybenzylidene)-3-oxo-2,3-dihydrobenzofuran-6-yl)oxy)acetamide (D7): Yield: 87.63%; yellow solid; m. p. 192.5–194.2 °C; ^{1}H NMR (400 MHz, DMSO-d_6): δ 8.00 – 7.94 (m, 2H, Ar-H), 7.71 (t, J = 1.8 Hz, 1H, Ar-H), 7.67 (d, J = 8.6 Hz, 1H, Ar-H), 7.45 – 7.37 (m, 2H, Ar-H), 7.07 (d, J = 1.8 Hz, 2H, Ar-H), 7.06 (s, 1H, Ar-H), 7.03 (d, J = 2.1 Hz, 1H, Ar-H), 6.79 (dd, J = 8.6, 2.2 Hz, 1H, =CH-), 4.82 (s, 2H, -CH$_2$-), 3.85 (s, 3H, -CH$_3$), 3.80 (s, 3H, -CH$_3$). ^{13}C NMR (101 MHz, DMSO-d_6): δ 181.73, 169.61, 167.57, 166.19, 163.01, 160.35, 146.38, 137.07, 132.78, 129.56, 128.99, 128.90, 125.60, 114.78, 113.43, 113.27, 107.21, 105.42, 98.60, 98.01, 68.41, 56.41, 56.01. HRMS (ESI): m/z for $C_{25}H_{21}O_8ClN_2S$ [M+H]$^+$ calcd 530.06709 found 530.06641.

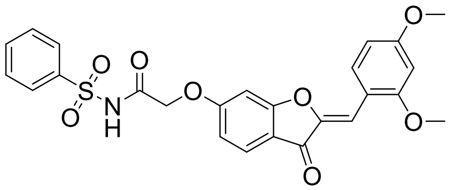

(*Z*)-*N*-((4-chlorophenyl)sulfonyl)-2-((2-(2,4-dimethoxybenzylidene)-3-oxo-2,3-dihydrobenzofuran-6-yl)oxy)acetamide (D8): Yield: 74.57%; yellow solid; m. p. 180.0–182.2 °C; ^{1}H NMR (400 MHz, DMSO-d_6): δ 8.13 (d, J = 8.8 Hz, 1H, Ar-H), 7.85 (dt, J = 6.8, 1.6 Hz, 2H, Ar-H), 7.62 (d, J = 8.6 Hz, 1H, Ar-H), 7.53 (s, 1H, Ar-H), 7.50 (dt, J = 8.7, 6.8 Hz, 3H, Ar-H), 7.06 (s, 1H, Ar-H), 6.85 (d, J = 2.1 Hz, 1H, Ar-H), 6.75 (d, J = 8.7 Hz, 1H, =CH-), 6.68 (d, J = 2.4 Hz, 1H, Ar-H), 4.68 (s, 2H, -CH$_2$-), 3.91 (s, 3H, -CH$_3$), 3.86 (s, 3H, -CH$_3$). ^{13}C NMR (101 MHz, DMSO-d_6): δ 181.74, 167.46, 167.23, 165.46, 163.11, 160.43, 146.29, 139.69, 134.23, 132.76, 129.61, 127.95, 125.79, 115.25, 113.34, 113.11, 107.23, 105.60, 98.61, 98.17, 66.88, 56.45, 56.04. HRMS (ESI): m/z for $C_{25}H_{20}NO_8S$ [M-H]$^-$ calcd 494.09041, found 494.09100.

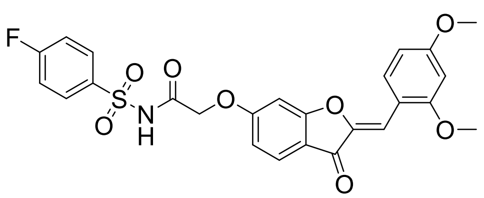

(*Z*)-2-((2-(2,4-dimethoxybenzylidene)-3-oxo-2,3-dihydrobenzofuran-6-yl)oxy)-*N*-((4-fluorophenyl)sulfonyl)acetamide (D9): Yield: 74.69%; yellow solid; m. p. 208.1–209.2 °C; ^{1}H NMR (400 MHz, DMSO-d_6): δ 8.11 (d, J = 8.7 Hz, 1H, Ar-H), 8.05 – 7.97 (m, 2H, Ar-H), 7.66 (d, J = 8.6 Hz, 1H, Ar-H), 7.46 (t, J = 8.8 Hz, 2H, Ar-H), 7.07 (s, 1H, Ar-H), 6.95 (d, J = 2.1 Hz, 1H, Ar-H), 6.78 (dd, J = 8.6, 2.2 Hz, 1H, Ar-H), 6.73 (dd, J = 8.8, 2.5 Hz, 1H, =CH-), 6.68 (d, J = 2.4 Hz, 1H, Ar-H), 4.88 (s, 2H, -CH$_2$-), 3.91 (s, 3H, -CH$_3$), 3.86 (s, 3H, -CH$_3$). ^{13}C NMR (101 MHz, DMSO-d_6): δ 181.74,

167.46, 167.31, 165.44, 163.09, 160.42, 146.29, 132.76, 131.36, 131.27, 125.80, 116.94, 116.71, 115.26, 113.34, 113.10, 107.24, 105.63, 98.58, 98.17, 66.87, 56.44, 56.02). ^{19}F NMR (376 MHz, DMSO-d_6): δ -104.47. HRMS (ESI): m/z for $C_{25}H_{21}FNO_8S$ $[M+H]^+$ calcd 514.09664 found 514.09631.

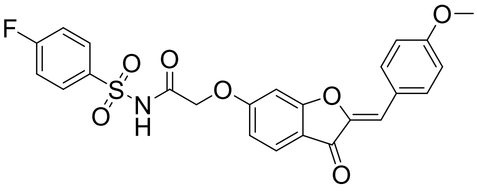

(*Z*)-*N*-((4-fluorophenyl)sulfonyl)-2-((2-(4-methoxybenzylidene)-3-oxo-2,3-dihydrobenzofuran-6-yl)oxy)acetamide (D10): Yield: 84.28%; buff solid; m. p. 222.8–224.5 °C; ^{1}H NMR (400 MHz, DMSO-d_6): δ 8.05 – 7.97 (m, 2H, Ar-H), 7.97 – 7.90 (m, 2H, Ar-H), 7.67 (d, *J* = 8.6 Hz, 1H, Ar-H), 7.46 (t, *J* = 8.8 Hz, 2H, Ar-H), 7.13 – 7.05 (m, 2H, Ar-H), 6.96 (d, *J* = 2.1 Hz, 1H, Ar-H), 6.85 (s, 1H, Ar-H), 6.79 (dd, *J* = 8.6, 2.2 Hz, 1H, =CH-), 4.88 (s, 2H, -CH_2-), 3.83 (s, 3H, -CH_3). ^{13}C NMR (101 MHz, DMSO-d_6): δ 181.90, 167.65, 167.35, 165.59, 161.13, 146.40, 133.61, 131.36, 131.26, 125.84, 124.89, 116.92, 116.69, 115.17, 115.10, 113.18, 112.17, 98.16, 66.90, 55.83. ^{19}F NMR (376 MHz, DMSO-d_6): δ -104.61. HRMS (ESI): m/z for $C_{24}H_{18}FNO_7SNa$ $[M+Na]^+$ calcd 506.06802 found 506.06735.

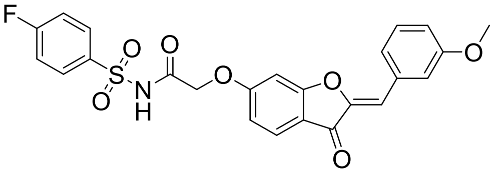

(*Z*)-*N*-((4-fluorophenyl)sulfonyl)-2-((2-(3-methoxybenzylidene)-3-oxo-2,3-dihydrobenzofuran-6-yl)oxy)acetamide (D11): Yield: 75.69%; buff solid; m. p. 214.4–215.8 °C; ^{1}H NMR (400 MHz, DMSO-d_6) δ 12.52 (s, 1H, -NH-), 8.05 – 7.97 (m, 2H, Ar-H), 7.68 (d, *J* = 8.6 Hz, 1H, Ar-H), 7.59 – 7.50 (m, 2H, Ar-H), 7.45 (t, *J* = 8.8 Hz, 3H, Ar-H), 7.08 – 6.99 (m, 2H, Ar-H), 6.83 (s, 1H, Ar-H), 6.80 (dd, *J* = 8.6, 2.2 Hz, 1H, =CH-), 4.88 (s, 2H, -CH_2-), 3.82 (s, 3H, -CH_3). ^{13}C NMR (101 MHz, DMSO-d_6): δ 182.16, 168.06, 167.31, 165.96, 164.07, 159.91, 147.70, 133.61, 131.34, 131.25, 130.53, 126.05, 124.05, 117.10, 116.92, 116.69, 115.92, 114.83, 111.62, 98.39, 66.97, 55.67. ^{19}F NMR (376 MHz, DMSO-d_6): δ -104.67. HRMS (ESI): m/z for $C_{24}H_{19}FNO_7S$ $[M+H]^+$ calcd 484.08608 found 484.08542.

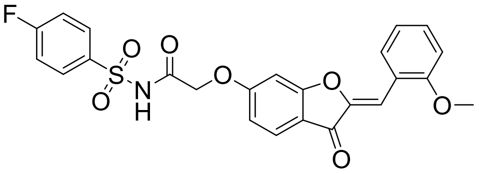

(*Z*)-*N*-((4-fluorophenyl)sulfonyl)-2-((2-(2-methoxybenzylidene)-3-oxo-2,3-dihydrobenzofuran-6-yl)oxy)acetamide (D12): Yield: 76.25%; buff solid; m. p. 217.8–219.2 °C; ^{1}H NMR (500 MHz, DMSO-d_6) δ 8.11 (d, *J* = 7.8 Hz, 1H, Ar-H), 8.05 – 7.98 (m, 2H, Ar-H), 7.65 (d, *J* = 8.5 Hz, 1 H, Ar-H), 7.44 (d, *J* = 9.2 Hz, 3H, Ar-H), 7.08 (s, 3H, Ar-H), 6.93 (s, 1H, Ar-H), 6.78 (d, *J* = 8.7 Hz, 1H, =CH-), 4.86 (s, 2H, -

CH_2-), 3.87 (s, 3H, -CH_3). ^{13}C NMR (101 MHz, DMSO-*d6*): δ 182.04, 167.91, 167.26, 165.78, 158.63, 147.54, 132.30, 131.43, 131.37, 131.28, 125.99, 121.32, 120.52, 116.94, 116.71, 114.94, 113.28, 111.98, 104.97, 98.28, 66.87, 56.26. ^{19}F NMR (376 MHz, DMSO-*d6*): δ -73.64. HRMS (ESI): m/z for $C_{24}H_{19}FNO_7S$ $[M+H]^+$ calcd 484.08608 found 484.08551.

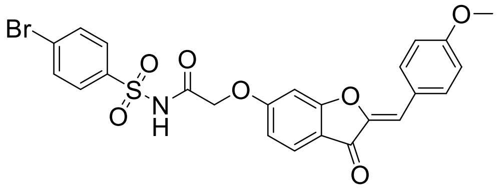

(*Z*)-*N*-((4-bromophenyl)sulfonyl)-2-((2-(4-methoxybenzylidene)-3-oxo-2,3-dihydrobenzofuran-6-yl)oxy)acetamide (D13): Yield: 82.34%; yellow solid; m. p. 223.8–224.7 °C; ^1H NMR (400 MHz, DMSO-*d*6): δ 7.99 – 7.91 (m, 2H, Ar-H), 7.89 – 7.79 (m, 4H, Ar-H), 7.67 (d, *J* = 8.6 Hz, 1H, Ar-H), 7.13 – 7.07 (m, 2H, Ar-H), 6.98 (d, *J* = 2.1 Hz, 1H, Ar-H), 6.86 (s, 1H, Ar-H), 6.80 (dd, *J* = 8.6, 2.2 Hz, 1H, =CH-), 4.86 (s, 2H, -CH_2-), 3.84 (s, 3H, -CH_3). ^{13}C NMR (101 MHz, DMSO-*d6*): δ 181.89, 167.77, 167.70, 165.74, 161.13, 146.44, 139.52, 133.63, 132.56, 130.00, 127.88, 125.85, 124.95, 115.15, 115.10, 113.21, 112.15, 98.18, 67.16, 55.87. HRMS (ESI): m/z for $C_{24}H_{17}BrNO_7S$ $[M-H]^-$ calcd 541.99036 found 541.99121.

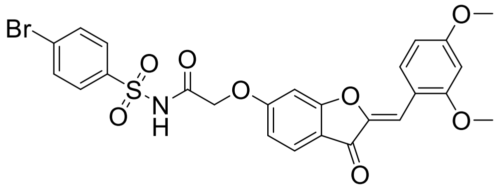

(*Z*)-*N*-((4-bromophenyl)sulfonyl)-2-((2-(2,4-dimethoxybenzylidene)-3-oxo-2,3-dihydrobenzofuran-6-yl)oxy)acetamide (D14): Yield: 80.12%; yellow solid; m. p. 249.1–250.6 °C; ^1H NMR (500 MHz, DMSO-*d*6) δ 8.12 (d, *J* = 8.8 Hz, 1H, Ar-H), 7.80 (d, *J* = 15.2 Hz, 3H, Ar-H), 7.78 (s, 1H, Ar-H), 7.64 (d, *J* = 8.6 Hz, 1H, Ar-H), 7.06 (s, 1H, Ar-H), 6.93 (d, *J* = 2.1 Hz, 1H, Ar-H), 6.77 (dd, *J* = 8.7, 2.0 Hz, 1H, Ar-H), 6.74 (dd, *J* = 8.7, 2.4 Hz, 1H, =CH-), 6.68 (d, *J* = 2.4 Hz, 1H), 4.80 (s, 2H), 3.91 (s, 3H, -CH_3), 3.85 (d, *J* = 7.3 Hz, 3H, -CH_3). ^{13}C NMR (126 MHz, DMSO-*d*6): δ 181.87, 168.00, 167.70, 165.88, 163.57, 160.15, 147.21, 139.91, 132.76, 132.57, 130.01, 128.94, 127.75, 125.87, 115.33, 112.63, 105.22V, 102.53, 98.09, 91.67, 67.40, 56.50, 56.10. HRMS (ESI): m/z for $C_{25}H_{21}O_8BrNS$ $[M+H]^+$ calcd 574.01658 found 574.01575.

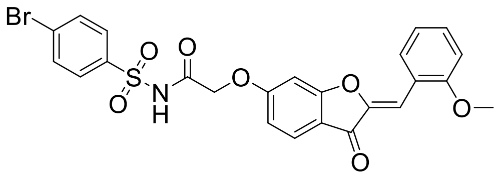

(*Z*)-*N*-((4-bromophenyl)sulfonyl)-2-((2-(2-methoxybenzylidene)-3-oxo-2,3-dihydrobenzofuran-6-yl)oxy)acetamide (D15): Yield: 76.25%; yellow solid; m. p. 204.8–205.9 °C; ^1H NMR (500 MHz, DMSO-*d*6): δ 7.92 (d, *J* = 7.9 Hz, 2H, Ar-H), 7.87 (d, *J* = 8.4 Hz, 2H, Ar-H), 7.62 (d, *J* = 7.2 Hz, 1H, Ar-H), 7.58 (dd, *J* = 8.1, 6.1 Hz, 2H, Ar-H), 7.03 (d, *J* = 8.4 Hz, 2H, Ar-H), 6.89 (d, *J* = 2.3 Hz, 1H, Ar-H), 6.78 (s,

1H, Ar-H), 6.72 (dd, J = 8.6, 2.2 Hz, 1H, =CH-), 4.92 (s, 2H, Ar-H) , -CH_2-, 3.78 (s, 3H, -CH_3). ^{13}C NMR (126 MHz, DMSO-d_6): δ 181.94, 167.72, 167.45, 161.19, 146.46, 139.90, 139.20, 138.86, 134.19, 133.65, 129.97, 129.63, 127.98, 125.87, 124.94, 115.21, 115.15, 113.20, 112.19, 98.19, 67.01, 55.90. HRMS (ESI): m/z for $C_{24}H_{19}O_7BrNS$ [M+H]$^+$ calcd 544.00601 found 544.00507.

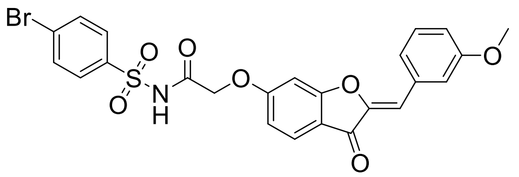

(*Z*)-*N*-((4-bromophenyl)sulfonyl)-2-((2-(3-methoxybenzylidene)-3-oxo-2,3-dihydrobenzofuran-6-yl)oxy)acetamide (D16): Yield: 72.65%; buff solid; m. p. 189.7–191.2 °C; ^{1}H NMR (400 MHz, DMSO-d_6): δ 7.86 – 7.74 (m, 4H, Ar-H), 7.67 (d, J = 8.6 Hz, 1H, Ar-H), 7.57 (d, J = 7.9 Hz, 1H, Ar-H), 7.53 (t, J = 2.0 Hz, 1H, Ar-H), 7.43 (t, J = 8.0 Hz, 1H, Ar-H), 7.04 (dd, J = 8.2, 2.6 Hz, 1H, Ar-H), 7.01 (d, J = 2.1 Hz, 1H, Ar-H), 6.83 (s, 1H, Ar-H), 6.79 (dd, J = 8.6, 2.2 Hz, 1H, =CH-), 4.82 (s, 2H, -CH_2-), 3.82 (s, 3H, -CH_3). ^{13}C NMR (101 MHz, DMSO-d_6): δ 182.15, 168.11, 168.07, 166.20, 159.92, 147.74, 140.08, 133.65, 132.42, 130.55, 129.92, 127.51, 126.00, 124.06, 117.07, 115.92, 114.67, 113.43, 111.57, 98.35, 67.44, 55.68. HRMS (ESI): m/z for $C_{24}H_{17}BrNO_7S$ [M-H]$^-$ calcd 541.99036 found 541.99072.

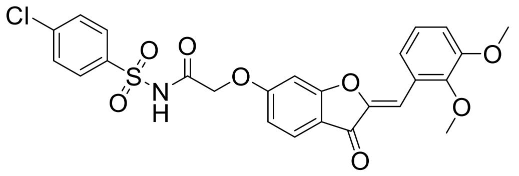

(*Z*)-*N*-((4-chlorophenyl)sulfonyl)-2-((2-(2,3-dimethoxybenzylidene)-3-oxo-2,3-dihydrobenzofuran-6-yl)oxy)acetamide (D17): Yield: 77.28%; buff solid; m. p. 214.2–215.9 °C; ^{1}H NMR (400 MHz, DMSO-d_6): δ 7.97 – 7.90 (m, 2H, Ar-H), 7.78 (dd, J = 7.9, 1.5 Hz, 1H, Ar-H), 7.69 (t, J = 2.4 Hz, 2H, Ar-H), 7.68 (d, J = 2.9 Hz, 1H, Ar-H), 7.24 (t, J = 8.0 Hz, 1H, Ar-H), 7.21 – 7.15 (m, 1H, Ar-H), 7.03 – 6.98 (m, 2H, Ar-H), 6.80 (dd, J = 8.6, 2.2 Hz, 1H, =CH-), 4.88 (s, 2H, -CH_2-), 3.85 (s, 3H, -CH_3), 3.82 (s, 3H, -CH_3). ^{13}C NMR (101 MHz, DMSO-d_6): δ 182.15, 168.02, 167.30, 165.93, 153.00, 148.74, 148.16, 139.24, 138.39, 130.03, 129.78, 126.11, 125.90, 125.00, 122.79, 115.21, 114.83, 113.36, 104.95, 98.33, 66.88, 61.57, 56.28. HRMS (ESI): m/z for $C_{25}H_{21}ClNO_8S$ [M+H]$^+$ calcd 530.06709, found 530.06653.

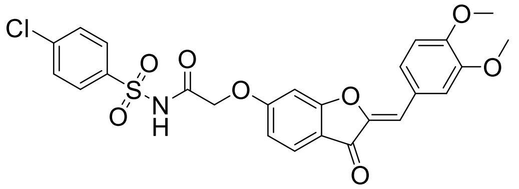

(*Z*)-*N*-((4-chlorophenyl)sulfonyl)-2-((2-(3,4-dimethoxybenzylidene)-3-oxo-2,3-dihydrobenzofuran-6-yl) oxy) acetamide (D18): Yield: 78.56%; yellow solid; m. p. 209.7–211.3 °C; ^{1}H NMR (400 MHz, DMSO-d_6): δ 7.98 – 7.90 (m, 2H, Ar-H), 7.77 (dd, J = 7.9, 1.6 Hz, 1H, Ar-H), 7.72 – 7.65 (m, 3H, Ar-H), 7.23 (t, J = 8.0 Hz, 1H, Ar-

H), 7.17 (dd, J = 8.4, 1.6 Hz, 1H, Ar-H), 7.00 (d, J = 3.8 Hz, 2H, Ar-H), 6.80 (dd, J = 8.6, 2.1 Hz, 1H, =CH-), 4.89 (s, 2H, -CH_2-), 3.84 (s, 3H, -CH_3), 3.82 (s, 3H, -CH_3). ^{13}C NMR (101 MHz, DMSO-d_6): δ 181.84, 167.63, 165.61, 151.06, 149.20, 146.47, 139.04, 138.77, 129.98, 129.91, 129.69, 125.83, 125.80, 125.05, 115.19, 114.79, 113.10, 112.56, 112.41, 98.39, 67.11, 56.07, 56.03. HRMS (ESI): m/z for $C_{25}H_{21}ClNO_8S$ $[M+H]^+$ calcd 530.06709, found 530.06635.

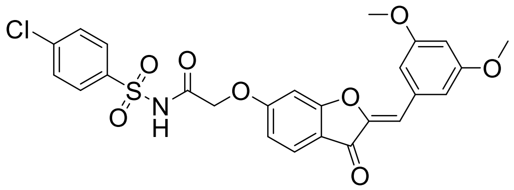

(*Z*)-*N*-((4-chlorophenyl)sulfonyl)-2-((2-(3,5-dimethoxybenzylidene)-3-oxo-2,3-dihydrobenzofuran-6-yl)oxy)acetamide (D19): Yield: 76.54%; yellow solid; m. p. 214.6–216.8 °C; ^1H NMR (400 MHz, DMSO-d_6): δ 7.91 (s, 2H, Ar-H), 7.69 – 7.60 (m, 3H, Ar-H), 7.15 (d, J = 2.3 Hz, 2H, Ar-H), 7.02 (d, J = 2.2 Hz, 1H, =CH-), 6.79 (d, J = 7.7 Hz, 2H, Ar-H), 6.62 (t, J = 2.3 Hz, 1H, Ar-H), 4.81 (s, 2H, -CH_2-), 3.80 (s, 6H, -CH_3). ^{13}C NMR (101 MHz, DMSO-d_6): δ 182.12, 168.07, 168.00, 166.14, 161.03, 147.77, 139.50, 138.59, 133.96, 129.86, 129.49, 125.94, 114.65, 113.38, 111.60, 109.72, 102.13, 98.38, 67.40, 55.77. HRMS (ESI): m/z for $C_{25}H_{20}NO_8SNa$ $[M+Na]^+$ calcd 552.04904, found 552.04852.

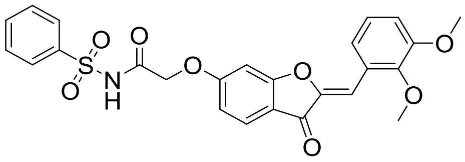

(*Z*)-2-((2-(2,3-dimethoxybenzylidene)-3-oxo-2,3-dihydrobenzofuran-6-yl)oxy)-*N*-(phenylsulfonyl)acetamide (D20): Yield: 80.71%; buff solid; m. p. 213.1–214.9 °C; ^1H NMR (400 MHz, DMSO-d_6): δ 12.63 (s, 1H, -NH-), 7.99 – 7.91 (m, 2H, Ar-H), 7.78 (dd, J = 7.8, 1.5 Hz, 1H, Ar-H), 7.72 – 7.58 (m, 4H, Ar-H), 7.25 (t, J = 8.1 Hz, 1H, Ar-H), 7.18 (dd, J = 8.3, 1.6 Hz, 1H, Ar-H), 7.02 (s, 1H, Ar-H), 6.98 (d, J = 2.1 Hz, 1H, Ar-H), 6.80 (dd, J = 8.6, 2.2 Hz, 1H, =CH-), 4.90 (s, 2H, -CH_2-), 3.86 (s, 3H, -CH_3), 3.83 (s, 3H, -CH_3). ^{13}C NMR (101 MHz, DMSO-d_6): δ 182.14, 167.99, 167.08, 165.90, 152.99, 148.73, 148.13, 139.57, 134.30, 129.64, 127.98, 126.07, 125.88, 124.94, 122.77, 115.18, 114.83, 113.33, 104.92, 98.31, 66.85, 61.55, 56.25. HRMS (ESI): m/z for $C_{25}H_{20}NO_8S$ $[M-H]^-$ calcd 494.09041, found 494.09097.

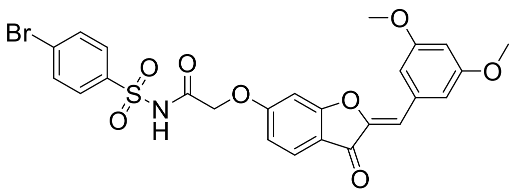

(*Z*)-*N*-((4-bromophenyl)sulfonyl)-2-((2-(3,5-dimethoxybenzylidene)-3-oxo-2,3-dihydrobenzofuran-6-yl)oxy)acetamide (D21): Yield: 87.28%; brownish solid; m. p. 195.2–197.3 °C; ^1H NMR (400 MHz, DMSO-d_6): δ 7.85 – 7.82 (m, 4H, Ar-H), 7.68 (d, J = 8.6 Hz, 1H, Ar-H), 7.16 (d, J = 2.2 Hz, 2H, Ar-H), 7.07 (d, J = 2.2 Hz, 1H, =CH-), 6.81 (d, J = 2.1 Hz, 1H, Ar-H), 6.79 (s, 1H, Ar-H), 6.63 (t, J = 2.3 Hz, 1H, Ar-H), 4.87

(s, 2H, -CH_2-), 3.81 (s, 6H, -CH_3). ^{13}C NMR (101 MHz, DMSO-d_6): δ 182.14, 168.05, 167.37, 165.94, 161.05, 147.76, 138.95, 133.96, 132.69, 130.02, 128.27, 126.03, 114.79, 113.35, 111.68, 109.76, 102.16, 98.45, 66.97, 55.80. HRMS (ESI): m/z for $C_{25}H_{20}BrNO_8SNa$ [M+Na]$^+$ calcd 595.99852 found 595.99701.

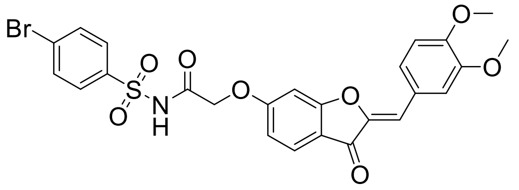

(*Z*)-*N*-((4-bromophenyl)sulfonyl)-2-((2-(3,4-dimethoxybenzylidene)-3-oxo-2,3-dihydrobenzofuran-6-yl)oxy)acetamide (D22): Yield: 80.29%; yellow solid; m. p. 166.1–168.4 °C; ^{1}H NMR (400 MHz, DMSO-d_6): δ 7.84 (s, 2H, Ar-H), 7.83 (s, 2H, Ar-H), 7.66 (d, J = 8.5 Hz, 1H, Ar-H), 7.10 (d, J = 8.4 Hz, 3H, Ar-H), 7.00 (d, J = 2.1 Hz, 1H, Ar-H), 6.84 (s, 1H, Ar-H), 6.79 (dd, J = 8.6, 2.1 Hz, 1H, =CH-), 4.86 (s, 2H, -CH_2-), 3.83 (d, J = 1.3 Hz, 6H, -CH_3). ^{13}C NMR (101 MHz, DMSO-d_6): δ 181.85, 167.62, 167.55, 151.03, 149.18, 148.52, 147.60, 146.46, 139.15, 132.70, 132.65, 130.02, 128.13, 125.04, 115.17, 114.74, 113.11, 112.59, 112.38, 98.37, 67.07, 56.06, 56.00. HRMS (ESI): m/z for $C_{25}H_{20}BrNO_8SNa$ [M+Na]$^+$ calcd 595.99852, found 595.99805.

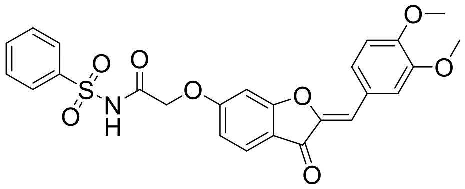

(*Z*)-2-((2-(3,4-dimethoxybenzylidene)-3-oxo-2,3-dihydrobenzofuran-6-yl)oxy)-*N*-(phenylsulfonyl)acetamide (D23): Yield: 76.57%; yellow solid; m. p. 160.7–162.3 °C; ^{1}H NMR (500 MHz, DMSO-d_6): δ 12.63 (s, 1H, -NH-), 7.97 – 7.95 (m, 1H, Ar-H), 7.94 (d, J = 1.5 Hz, 1H, Ar-H), 7.72 – 7.62 (m, 3H, Ar-H), 7.61 – 7.59 (m, 1H, Ar-H), 7.58 (d, J = 2.0 Hz, 1H, Ar-H), 7.11 (d, J = 8.3 Hz, 1H, Ar-H), 7.00 (d, J = 2.2 Hz, 1H, Ar-H), 6.85 (s, 1H, Ar-H), 6.80 (dd, J = 8.6, 2.3 Hz, 1H, =CH-), 4.89 (s, 2H, -CH_2-), 3.84 (s, 6H, -CH_3. ^{13}C NMR (101 MHz, DMSO-d_6): δ 181.86, 167.61, 167.16, 165.55, 151.07, 149.20, 146.44, 139.62, 134.30, 129.64, 127.96, 125.86, 125.81, 125.02, 115.21, 114.78, 113.11, 112.59, 112.40, 98.40, 66.92, 56.08, 56.02. HRMS (ESI): m/z for $C_{25}H_{22}NO_8S$ [M+H]$^+$ calcd 496.10606, found 496.10516.

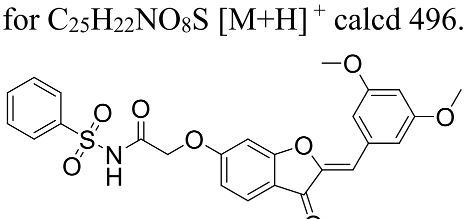

(*Z*)-2-((2-(3,5-dimethoxybenzylidene)-3-oxo-2,3-dihydrobenzofuran-6-yl)oxy)-*N*-(phenylsulfonyl)acetamide (D24): Yield: 76.31%; buff solid; m. p. 204.6–206.8 °C; ^{1}H NMR (500 MHz, DMSO-d_6): δ 12.62 (s, 1H, -NH-), 7.98 – 7.92 (m, 2H, Ar-H), 7.68 – 7.65 (m, 2H, Ar-H), 7.64 (d, J = 1.6 Hz, 1H, Ar-H), 7.62 (d, J = 1.6 Hz, 1H, Ar-H), 7.15 (d, J = 2.2 Hz, 2H, Ar-H), 7.02 (d, J = 2.1 Hz, 1H, Ar-H), 6.80 (dd, J = 8.6, 2.1 Hz, 1H, =CH-), 6.78 (s, 1H, Ar-H), 6.62 (t, J = 2.2 Hz, 1H, Ar-H), 4.90 (s, 2H, -CH_2-),

3.80 (s, 6H, -CH_3). ^{13}C NMR (101 MHz, DMSO-d_6): δ 182.15, 168.04, 167.10, 165.92, 161.07, 147.74, 139.56, 134.33, 133.94, 129.65, 127.95, 126.03, 114.80, 113.40, 111.67, 109.77, 102.15, 98.40, 66.88, 55.81. HRMS (ESI): m/z for $C_{25}H_{22}NO_8S$ [M+H]$^+$ calcd 496.10606, found 496.10532.

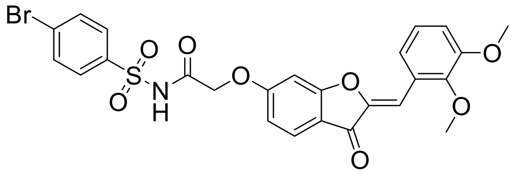

(*Z*)-*N*-((4-bromophenyl)sulfonyl)-2-((2-(2,3-dimethoxybenzylidene)-3-oxo-2,3-dihydrobenzofuran-6-yl)oxy)acetamide (D25): Yield: 79.66%; buff solid; m. p. 206.3–208.9 °C; ^1H NMR (500 MHz, DMSO-d_6): δ 8.13 (d, J = 8.7 Hz, 1H, Ar-H), 7.86 – 7.78 (m, 4H, Ar-H), 7.65 (d, J = 8.5 Hz, 1H, Ar-H), 7.07 (s, 1H, Ar-H), 6.94 (d, J = 2.4 Hz, 1H, =CH-), 6.80 – 6.76 (m, 1H, Ar-H), 6.76 – 6.72 (m, 1H, Ar-H), 6.68 (d, J = 2.5 Hz, 1H, Ar-H), 4.82 (s, 2H, -CH_2-), 3.91 (s, 3H, -CH_3), 3.86 (s, 3H, -CH_3). ^{13}C NMR (101 MHz, DMSO-d_6): δ 182.14, 168.05, 167.94, 166.15, 152.99, 148.73, 148.18, 132.53, 132.47, 129.95, 129.92, 126.05, 125.93, 125.01, 122.80, 115.19, 114.69, 113.40, 104.90, 98.28, 67.34, 61.57, 56.27. HRMS (ESI): m/z for $C_{25}H_{19}BrNO_8S$ [M-H]$^-$ calcd 572.00093, found 572.00195.

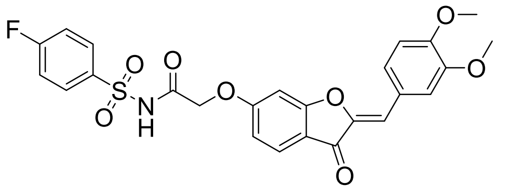

(*Z*)-2-((2-(3,4-dimethoxybenzylidene)-3-oxo-2,3-dihydrobenzofuran-6-yl)oxy)-*N*-((4-fluorophenyl)sulfonyl)acetamide (D26): Yield: 77.52%; yellow solid; m. p. 182.3–184.6 °C; ^1H NMR (400 MHz, DMSO-d_6): δ 8.03 – 7.97 (m, 2H, Ar-H), 7.66 (d, J = 8.6 Hz, 1H, Ar-H), 7.59 (dd, J = 8.3, 2.0 Hz, 1H, Ar-H), 7.57 (d, J = 2.0 Hz, 1H, Ar-H), 7.48 – 7.41 (m, 2H, Ar-H), 7.10 (d, J = 8.4 Hz, 1H, Ar-H), 6.99 (d, J = 2.2 Hz, 1H, Ar-H), 6.84 (s, 1H, Ar-H), 6.79 (dd, J = 8.6, 2.1 Hz, 1H, =CH-), 4.86 (s, 2H, -CH_2-), 3.83 (s, 6H, -CH_3). ^{13}C NMR (101 MHz, DMSO-d_6): δ 181.86, 167.61, 167.40, 165.57, 151.04, 149.17, 146.45, 131.33, 131.23, 125.83, 125.78, 125.01, 116.89, 116.67, 115.18, 114.74, 113.09, 112.58, 112.36, 98.37, 66.80, 56.05, 55.99. ^{19}F NMR (471 MHz, DMSO-d_6): δ -104.62. HRMS (ESI): m/z for $C_{25}H_{19}O_8FNS$ [M-H]$^-$ calcd 512.08099 found 512.08179.

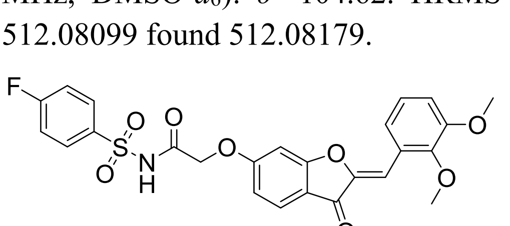

(*Z*)-2-((2-(2,3-dimethoxybenzylidene)-3-oxo-2,3-dihydrobenzofuran-6-yl)oxy)-*N*-((4-fluorophenyl)sulfonyl)acetamide (D27): Yield: 82.48%; buff solid; m. p. 157.4–159.2 °C; ^1H NMR (500 MHz, DMSO-d_6): δ 8.00 (dd, J = 8.9, 5.2 Hz, 2H, Ar-H), 7.77 (d, J = 7.7 Hz, 1H, Ar-H), 7.69 (d, J = 8.6 Hz, 1H, Ar-H), 7.45 (t, J = 8.7 Hz, 2H, Ar-

H), 7.24 (t, J = 8.0 Hz, 1H, =CH-), 7.01 (s, 2H, Ar-H), 6.98 (s, 1H, Ar-H), 6.82 – 6.77 (m, 1H, Ar-H), 4.88 (s, 2H, -CH_2-), 3.85 (s, 3H, -CH_3), 3.82 (d, J = 3.4 Hz, 3H, -CH_3). ^{13}C NMR (101 MHz, DMSO-d_6): δ 182.13, 168.07, 168.04, 166.22, 152.97, 148.71, 148.17, 131.08, 130.98, 126.00, 125.90, 124.98, 122.77, 116.55, 116.32, 115.16, 114.63, 113.42, 104.84, 98.24, 67.49, 61.55, 56.25. ^{19}F NMR (471 MHz, DMSO-d_6): δ -104.61. HRMS (ESI): m/z for $C_{25}H_{21}FNO_8S$ [M+H] $^+$ calcd 514.09664 found 514.09576.

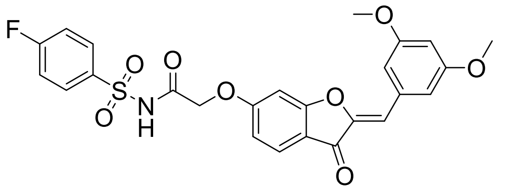

(*Z*)-2-((2-(3,5-dimethoxybenzylidene)-3-oxo-2,3-dihydrobenzofuran-6-yl)oxy)-*N*-((4-fluorophenyl)sulfonyl)acetamide (D28): Yield: 79.03%; brownish solid; m. p. 204.1–206.6 °C; ^{1}H NMR (500 MHz, DMSO-*d6*) δ 8.01 (dd, J = 8.7, 5.0 Hz, 2H, Ar-H), 7.68 (d, J = 8.5 Hz, 1H, Ar-H), 7.46 (t, J = 8.7 Hz, 2H, Ar-H), 7.15 (d, J = 2.2 Hz, 2H, Ar-H), 7.06 (d, J = 2.2 Hz, 1H, Ar-H), 6.80 (dd, J = 8.6, 2.3 Hz, 1H, =CH-), 6.79 (s, 1H, Ar-H), 6.62 (d, J = 2.3 Hz, 1H, Ar-H), 4.89 (s, 2H, -CH_2-), 3.81 (s, 6H, -CH_3). ^{13}C NMR (126 MHz, DMSO-d_6): δ 182.24, 168.12, 167.31, 166.02, 161.14, 147.82, 134.02, 131.43, 131.35, 126.12, 117.00, 116.82, 114.87, 113.43, 111.75, 109.84, 102.23, 98.54, 66.98, 55.87. ^{19}F NMR (471 MHz, DMSO-d_6): δ -104.44. HRMS (ESI): m/z for $C_{25}H_{21}FNO_8S$ [M+H]$^+$ calcd 514.09664, found 514.09595.

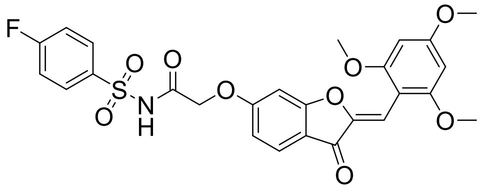

(*Z*)-*N*-((4-fluorophenyl)sulfonyl)-2-((3-oxo-2-(2,4,6-trimethoxybenzylidene)-2,3-dihydrobenzofuran-6-yl)oxy)acetamide (D29): Yield: 80.26%; yellow solid; m. p. 230.6–232.8 °C; ^{1}H NMR (400 MHz, DMSO-d_6): δ 7.99 (dd, J = 9.0, 5.1 Hz, 1H, Ar-H), 7.63 (d, J = 8.5 Hz, 1H, Ar-H), 7.44 (s, 1H, Ar-H), 6.83 (d, J = 2.1 Hz, 1H, Ar-H), 6.75 (s, 1H, Ar-H), 6.73 (dd, J = 8.6, 2.2 Hz, 1H, =CH-), 6.32 (s, 2H, Ar-H), 4.84 (s, 2H, -CH_2-), 3.84 (s, 3H, -CH_3), 3.83 (s, 6H, -CH_3). ^{13}C NMR (126 MHz, DMSO-d_6): δ 181.90, 167.67, 167.45, 165.71, 163.59, 160.15, 147.19, 136.18, 131.36, 131.29, 116.93, 116.75, 115.43, 112.59, 105.31, 102.49, 98.10, 91.64, 67.05, 56.47, 56.07. ^{19}F NMR (376 MHz, DMSO-d_6): δ -104.68. HRMS (ESI): m/z for $C_{26}H_{23}FNO_9S$ [M+H]$^+$ calcd 544.10721, found 544.10645.

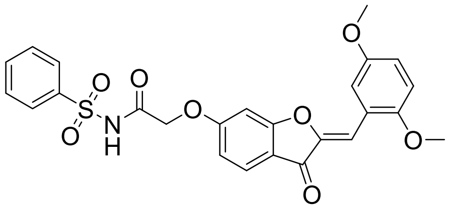

(*Z*)-2-((2-(2,5-dimethoxybenzylidene)-3-oxo-2,3-dihydrobenzofuran-6-yl)oxy)-*N*-(phenylsulfonyl)acetamide (D30): Yield: 83.41%; orange solid; m. p. 144.7–146.8 °C;

^{1}H NMR (400 MHz, DMSO-d_6): δ 8.47 (td, J = 4.4, 3.9, 2.1 Hz, 3H, Ar-H), 8.35 (d, J = 8.6 Hz, 1H, Ar-H), 8.11 – 8.04 (m, 3H, Ar-H), 7.81 (d, J = 2.2 Hz, 2H, Ar-H), 7.79 (s, 1H, Ar-H), 7.58 (d, J = 2.1 Hz, 1H, Ar-H), 7.47 (dd, J = 8.6, 2.1 Hz, 1H, =CH-), 5.21 (s, 2H, -CH$_2$-), 4.60 (s, 3H, -CH$_3$), 4.55 (s, 3H, -CH$_3$). ^{13}C NMR (101 MHz, DMSO-d_6): δ 181.96, 171.42, 168.13, 167.39, 153.54, 153.08, 147.80, 146.27, 130.40, 128.06, 126.98, 125.61, 121.22, 117.01, 116.73, 113.86, 113.77, 112.92, 104.38, 98.17, 69.95, 56.67, 56.03. HRMS (ESI): m/z for $C_{25}H_{22}NO_8S$ [M+H]$^+$ calcd 496.10606, found 496.10541.

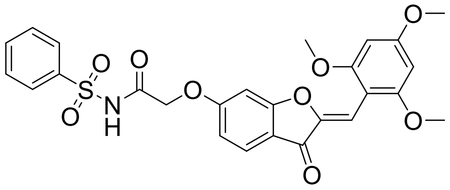

(*Z*)-2-((3-oxo-2-(2,4,6-trimethoxybenzylidene)-2,3-dihydrobenzofuran-6-yl)oxy)-*N*-(phenylsulfonyl)acetamide (D31): Yield: 78.21%; orange solid; m. p. 163.4–165.1 °C; ^{1}H NMR (400 MHz, DMSO-d_6): δ 7.96 – 7.91 (m, 2H, Ar-H), 7.71 – 7.65 (m, 1H, Ar-H), 7.63 (d, J = 4.2 Hz, 1H, Ar-H), 7.61 – 7.57 (m, 2H, Ar-H), 6.82 (d, J = 2.1 Hz, 1H, Ar-H), 6.75 (s, 1H, Ar-H), 6.73 (dd, J = 8.6, 2.2 Hz, 1H, =CH-), 6.33 (s, 2H, Ar-H), 4.86 (s, 2H, -CH$_2$-), 3.84 (s, 3H, -CH$_3$), 3.83 (s, 6H, -CH$_3$). ^{13}C NMR (126 MHz, DMSO-d_6): δ 181.90, 167.67, 167.20, 165.68, 163.59, 160.15, 147.20, 139.68, 134.35, 129.71, 128.00, 125.93, 115.45, 112.59, 105.32, 102.50, 98.11, 91.66, 66.95, 56.51, 56.10. HRMS (ESI): m/z for $C_{26}H_{24}NO_9S$ [M+H]$^+$ calcd 526.11663 found 526.11609.

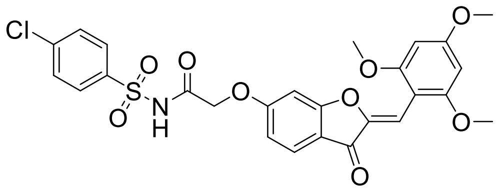

(*Z*)-*N*-((4-chlorophenyl)sulfonyl)-2-((3-oxo-2-(2,4,6-trimethoxybenzylidene)-2,3-dihydrobenzofuran-6-yl)oxy)acetamide (D32): Yield: 81.54%; orange solid; m. p. 206.4–208.5 °C; ^{1}H NMR (500 MHz, DMSO-d_6): δ 7.68 (d, J = 8.5 Hz, 2H, Ar-H), 7.53 (d, J = 8.5 Hz, 1H, Ar-H), 7.33 (d, J = 8.1 Hz, 2H, Ar-H), 6.69 (s, 1H, Ar-H), 6.62 (d, J = 9.0 Hz, 1H, =CH-), 6.60 (s, 1H, Ar-H), 6.28 (s, 2H, Ar-H), 4.39 (d, J = 5.6 Hz, 2H, -CH$_2$-), 3.80 (d, J = 4.5 Hz, 9H, -CH$_3$). ^{13}C NMR (126 MHz, DMSO-d_6): δ 181.90, 167.89, 167.11, 163.50, 160.13, 147.34, 145.08, 135.24, 129.16, 128.29, 128.24, 125.56, 114.52, 112.94, 104.89, 102.58, 97.82, 91.65, 66.88, 56.48, 56.06. HRMS (ESI): m/z for $C_{26}H_{23}ClNO_9S$ [M+H]$^+$ calcd 560.07766, found 560.07703.

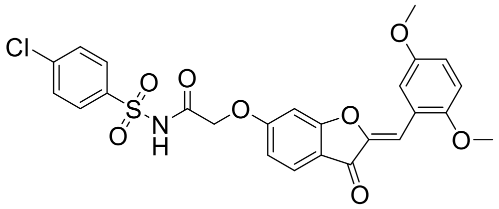

(*Z*)-*N*-((4-chlorophenyl)sulfonyl)-2-((2-(2,5-dimethoxybenzylidene)-3-oxo-2,3-dihydrobenzofuran-6-yl)oxy)acetamide (D33): Yield: 76.43%; yellow solid; m. p.

140.9–142.4 °C; ^{1}H NMR (400 MHz, DMSO-d_6): δ 7.91 (d, J = 8.6 Hz, 2H, Ar-H), 7.71 (d, J = 1.8 Hz, 1H, Ar-H), 7.66 (t, J = 9.0 Hz, 3H, Ar-H), 7.07 (d, J = 1.7 Hz, 2H, Ar-H), 7.06 (s, 1H, Ar-H), 7.04 (d, J = 2.2 Hz, 1H, Ar-H), 6.79 (dd, J = 8.6, 2.1 Hz, 1H, =CH-) , 4.83 (s, 2H, -CH_2-), 3.85 (s, 3H, -CH_3), 3.80 (s, 3H, -CH_3). ^{13}C NMR (101 MHz, DMSO-d_6): δ 182.03, 167.93, 167.80, 165.99, 153.47, 153.12, 147.66, 139.17, 138.78, 129.90, 129.57, 125.97, 121.13, 116.95, 116.89, 114.76, 113.39, 112.86, 104.80, 98.39, 67.24, 56.64, 55.97. HRMS (ESI): m/z for $C_{25}H_{21}O_8ClNS$ [M+H]$^+$ calcd 530.06709 found 530.06628.

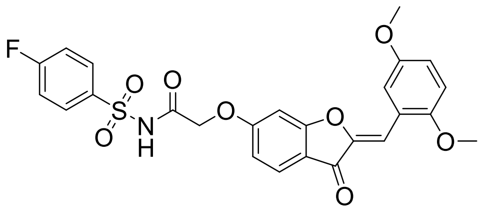

(*Z*)-2-((2-(2,5-dimethoxybenzylidene)-3-oxo-2,3-dihydrobenzofuran-6-yl)oxy)-*N*-((4-fluorophenyl)sulfonyl)acetamide (D34): Yield: 75.93%; yellow solid; m. p. 234.5–236.1 °C; ^{1}H NMR (400 MHz, DMSO-d_6): δ 8.01 – 7.93 (m, 2H, Ar-H), 7.71 – 7.69 (m, 1H, Ar-H), 7.67 (d, J = 8.6 Hz, 1H, Ar-H), 7.45 – 7.37 (m, 2H, Ar-H), 7.07 (s, 2H, Ar-H), 7.05 (s, 1H, Ar-H), 7.03 (d, J = 2.2 Hz, 1H, Ar-H), 6.79 (dd, J = 8.6, 2.1 Hz, 1H, =CH-), 4.83 (s, 2H, -CH_2-), 3.85 (s, 3H, -CH_3), 3.79 (s, 3H, -CH_3). ^{13}C NMR (126 MHz, DMSO-d_6): δ 182.10, 168.00, 166.13, 153.55, 153.20, 147.72, 131.16, 131.08, 125.99, 121.22, 117.02, 116.94, 116.65, 116.46, 114.80, 113.44, 112.91, 104.87, 98.43, 67.49, 56.69, 56.01. ^{19}F NMR (376 MHz, DMSO-d_6): δ -105.44. HRMS (ESI): m/z for $C_{25}H_{21}FNO_8S$ [M+Na]$^+$ calcd 514.09664, found 514.09613.

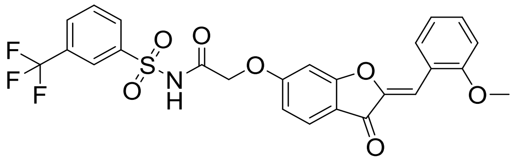

(*Z*)-2-((2-(2-methoxybenzylidene)-3-oxo-2,3-dihydrobenzofuran-6-yl)oxy)-*N*-((3-(trifluoromethyl)phenyl)sulfonyl)acetamide (D35): Yield: 79.01%; buff solid; m. p. 227.4–229.1 °C; ^{1}H NMR (500 MHz, DMSO-d_6): δ 8.24 (d, J = 8.0 Hz, 1H, Ar-H), 8.17 (s, 1H, Ar-H), 8.15 (d, J = 7.8 Hz, 1H, Ar-H), 8.09 (d, J = 7.9 Hz, 1H, Ar-H), 7.89 (t, J = 8.0 Hz, 1H, Ar-H), 7.67 (d, J = 8.6 Hz, 1H, Ar-H), 7.46 (t, J = 7.9 Hz, 1H, Ar-H), 7.14 (d, J = 8.6 Hz, 1H, Ar-H), 7.11 (s, 1H, Ar-H), 7.02 (d, J = 2.0 Hz, 1H, Ar-H), 6.81 (dd, J = 8.6, 2.0 Hz, 1H, =CH-), 4.90 (s, 2H, -CH_2-), 3.90 (s, 3H, -CH_3). ^{13}C NMR (126 MHz, DMSO-d_6): δ 182.17, 168.03, 167.94, 165.93, 158.74, 147.63, 141.24, 132.43, 132.09, 131.53, 131.34, 130.86, 130.00, 126.05, 124.61, 122.74, 121.39, 120.58, 114.98, 113.41, 112.11, 105.09, 98.33, 67.18, 56.37. ^{19}F NMR (376 MHz, DMSO-d_6): δ -61.43. HRMS (ESI): m/z for $C_{25}H_{19}F_3NO_7S$ [M+H]$^+$ calcd 524.08288 found 534.08228.

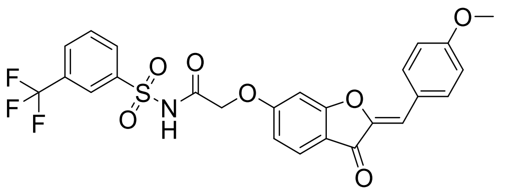

(*Z*)-2-((2-(4-methoxybenzylidene)-3-oxo-2,3-dihydrobenzofuran-6-yl)oxy)-*N*-((3-(trifluoromethyl)phenyl)sulfonyl)acetamide (D36): Yield: 83.44%; buff solid; m. p. 196.0–198.3 °C; ^{1}H NMR (400 MHz, DMSO-d_6): δ 8.23 (d, J = 7.9 Hz, 1H, Ar-H), 8.16 (d, J = 2.0 Hz, 1H, Ar-H), 8.08 (d, J = 7.7 Hz, 1H, Ar-H), 7.96 – 7.91 (m, 2H, Ar-H), 7.87 (t, J = 7.9 Hz, 1H, Ar-H), 7.65 (d, J = 8.6 Hz, 1H, Ar-H), 7.11 – 7.05 (m, 2H, Ar-H), 6.99 (d, J = 2.1 Hz, 1H, Ar-H), 6.85 (s, 1H, Ar-H), 6.79 (dd, J = 8.6, 2.2 Hz, 1H, =CH-), 4.88 (s, 2H, -CH$_2$-), 3.83 (s, 3H, -CH$_3$). ^{13}C NMR (151 MHz, DMSO-*d6*): δ 182.06, 168.28, 167.78, 165.80, 161.24, 146.48, 141.63, 133.71, 131.98, 131.22, 130.57, 130.17, 129.95, 125.89, 124.90, 124.55, 115.18, 115.15, 113.32, 112.29, 98.14, 67.36, 55.90. ^{19}F NMR (376 MHz, DMSO-d_6): δ -61.42. HRMS (ESI): m/z for $C_{25}H_{19}F_3NO_7S$ [M+H]$^+$ calcd 534.08288 found 534.08221.

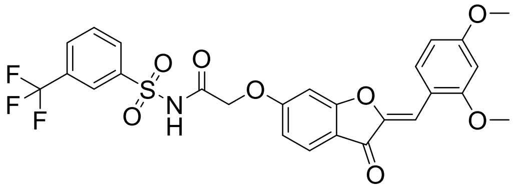

(*Z*)-2-((2-(2,4-dimethoxybenzylidene)-3-oxo-2,3-dihydrobenzofuran-6-yl)oxy)-*N*-((3-(trifluoromethyl)phenyl)sulfonyl)acetamide (D37): Yield: 78.30%; yellow solid; m. p. 211.5–213.0 °C; ^{1}H NMR (500 MHz, DMSO-d_6): δ 8.25 (d, J = 8.0 Hz, 1H, Ar-H), 8.18 (s, 1H, Ar-H), 8.08 (dd, J = 8.7, 3.3 Hz, 2H, Ar-H), 7.88 (t, J = 8.0 Hz, 1H, Ar-H), 7.63 (d, J = 8.5 Hz, 1H, Ar-H), 7.05 (s, 1H, Ar-H), 6.95 (d, J = 2.0 Hz, 1H, Ar-H), 6.78 (d, J = 8.5 Hz, 1H, =CH-), 6.71 – 6.67 (m, 1H, Ar-H), 6.65 (d, J = 2.4 Hz, 1H, Ar-H), 4.88 (s, 2H, -CH$_2$-), 3.89 (s, 3H, -CH$_3$), 3.84 (s, 3H, -CH$_3$). ^{13}C NMR (126 MHz, DMSO-d_6): δ 181.85, 167.79, 167.56, 165.49, 163.18, 160.52, 146.37, 140.94, 132.85, 132.14, 131.42, 131.04, 130.06, 125.84, 124.66, 122.71, 115.36, 113.43, 113.15, 107.26, 105.73, 98.64, 98.24, 67.04, 56.50, 56.07. ^{19}F NMR (471 MHz, DMSO-d_6): δ -61.43, -61.53, -61.66. HRMS (ESI): m/z for $C_{26}H_{21}F_3NO_8S$ [M+H]$^+$ calcd 564.09345 found 564.09302.

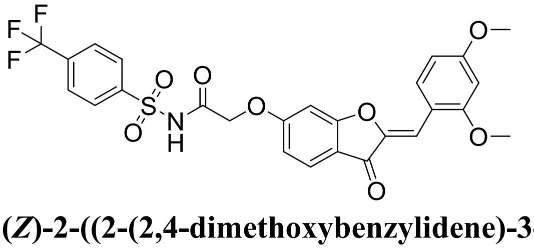

(*Z*)-2-((2-(2,4-dimethoxybenzylidene)-3-oxo-2,3-dihydrobenzofuran-6-yl)oxy)-*N*-((4-(trifluoromethyl)phenyl)sulfonyl)acetamide (D38): Yield: 78.51%; yellow solid; m. p. 230.3–232.4 °C; ^{1}H NMR (500 MHz, DMSO-d_6) δ 8.09 (d, J = 8.7 Hz, 1H, Ar-H), 8.02 (s, 1H, Ar-H), 7.88 (s, 1H, Ar-H), 7.84 (s, 1H, Ar-H), 7.73 (d, J = 8.2 Hz, 1H, Ar-H), 7.58 (d, J = 8.6 Hz, 1H, Ar-H), 7.02 (s, 1H, Ar-H), 6.84 (s, 1H, Ar-H), 6.71 (d, J = 2.3 Hz, 1H, Ar-H), 6.69 (t, J = 3.9 Hz, 1H, =CH-), 6.64 (d, J = 2.4 Hz, 1H, Ar-H),

4.66 (s, 2H, $-CH_2-$), 3.87 (s, 3H, $-CH_3$), 3.81 (s, 3H, $-CH_3$). ^{13}C NMR (100 MHz, DMSO-d_6): δ 205.9, 178.9, 158.9, 146.9, 133.8, 132.8, 130.0, 129.5, 129.2, 122.7, 114.2, 110.0, 72.8, 55.6, 50.6, 46.2, 30.9. ^{19}F NMR (376 MHz, DMSO-d_6): δ -61.31, -61.52, -61.72. HRMS (ESI): m/z for $C_{26}H_{19F}NO_8S$ $[M-H]^-$ calcd 562.07780 found 562.07963.

B) NMR and HRMS spectra of products.

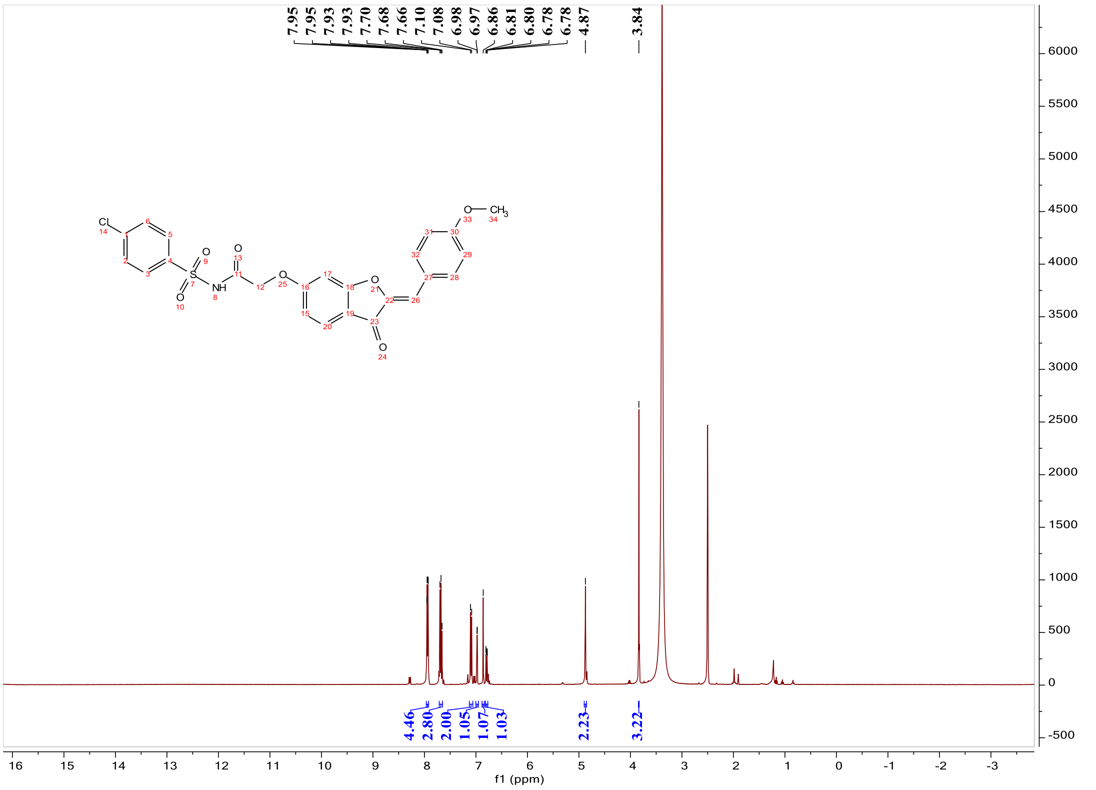

^{1}H NMR (400 MHz, DMSO-d_6) spectrum of compound **D1**

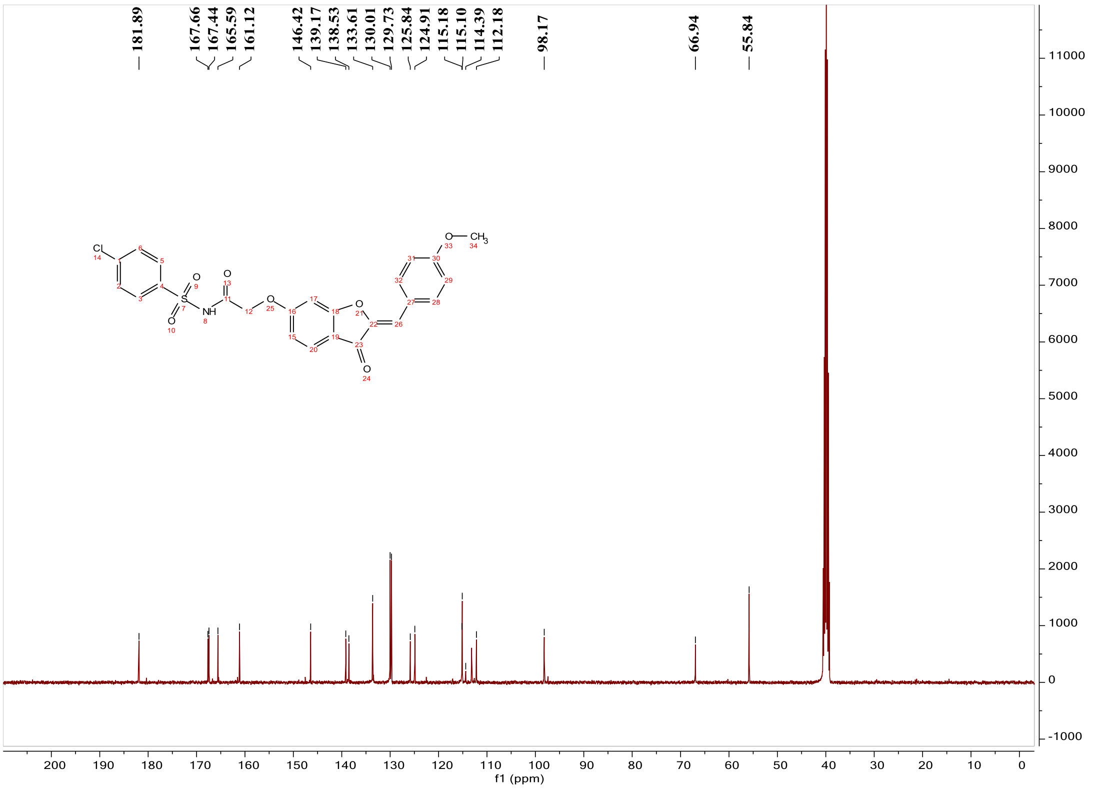

^{13}C NMR (101 MHz, DMSO-d_6) spectrum of compound **D1**

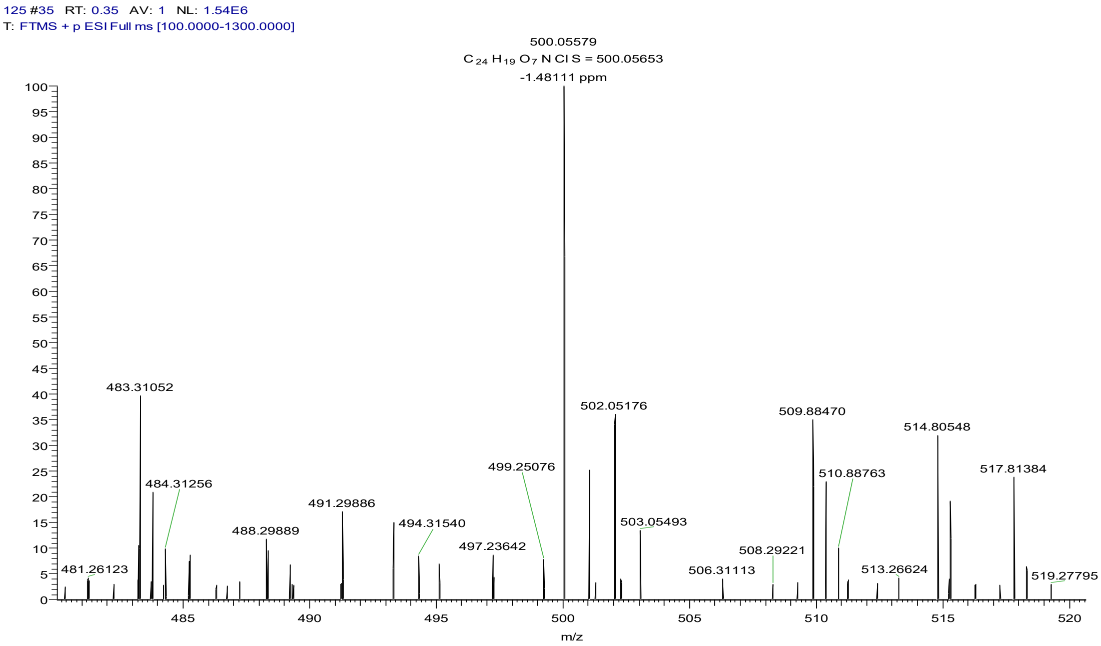

HRMS spectrum of compound **D1**

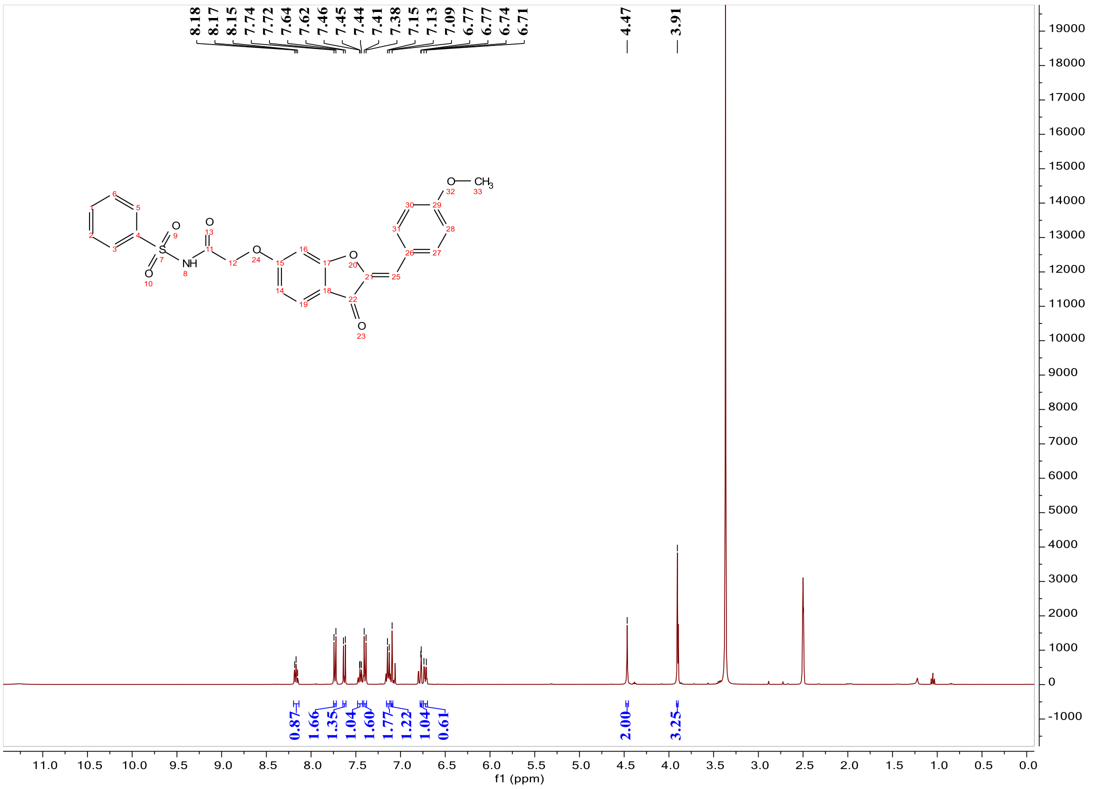

^{1}H NMR (400 MHz, DMSO-d_6) spectrum of compound **D2**

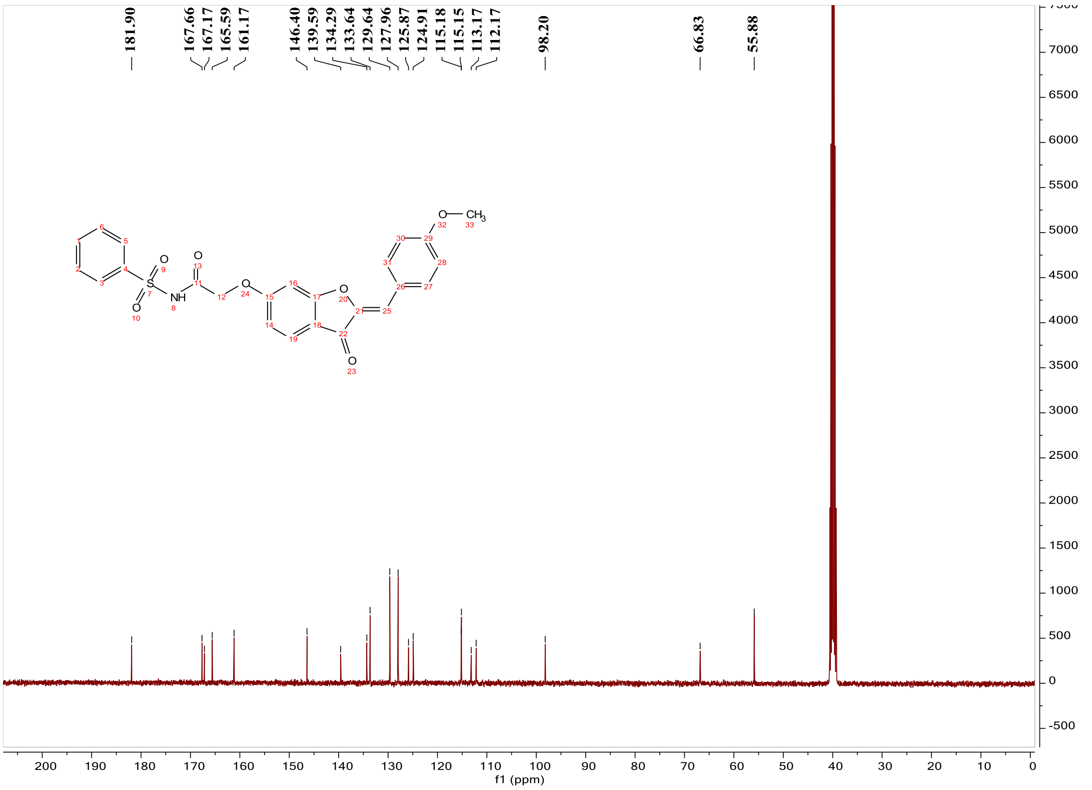

^{13}C NMR (101 MHz, DMSO-d_6) spectrum of compound **D2**

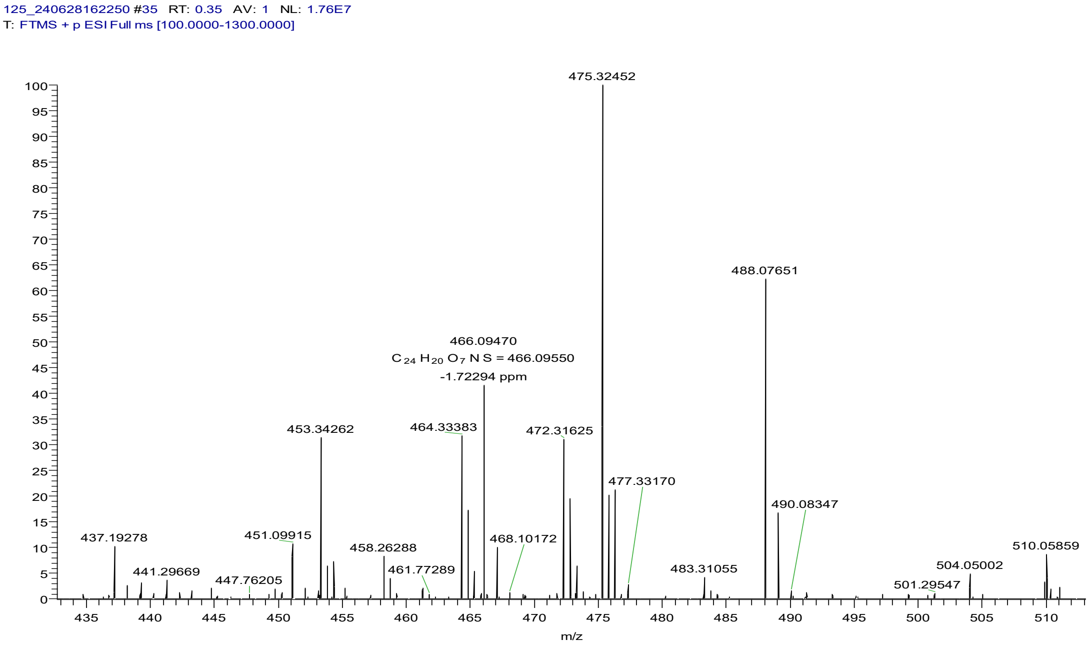

HRMS spectrum of compound **D2**

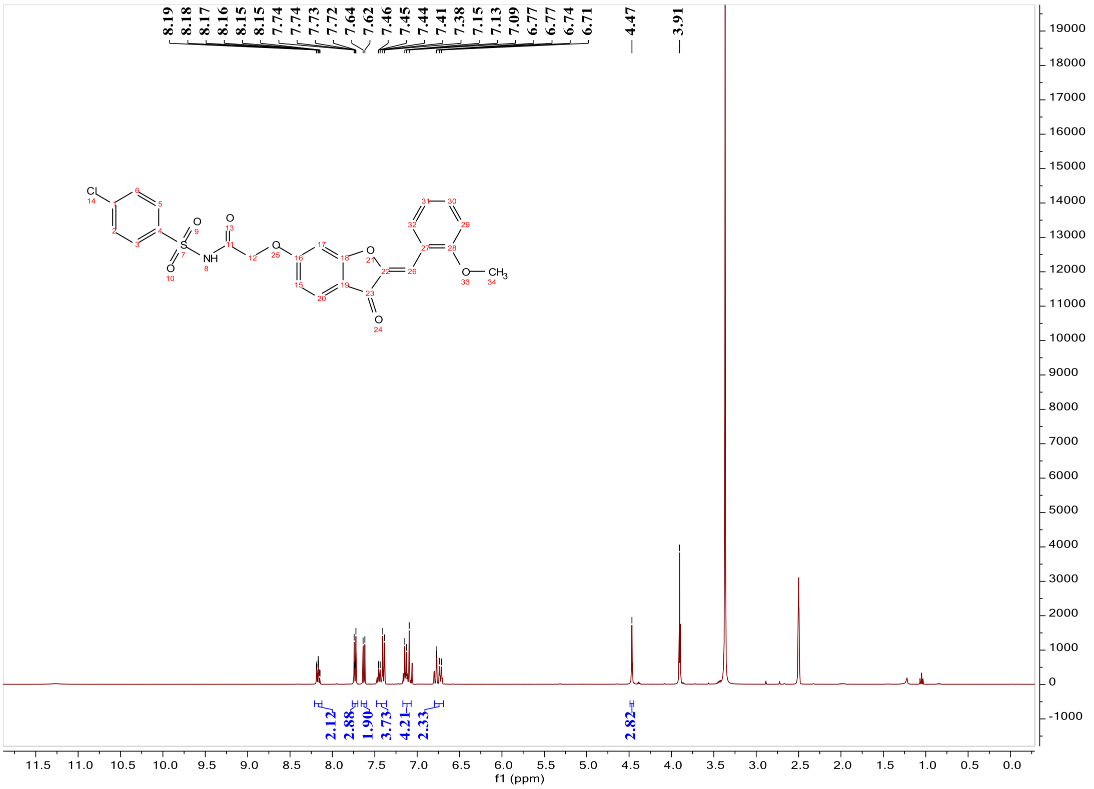

^{1}H NMR (400 MHz, DMSO-d_6) spectrum of compound **D3**

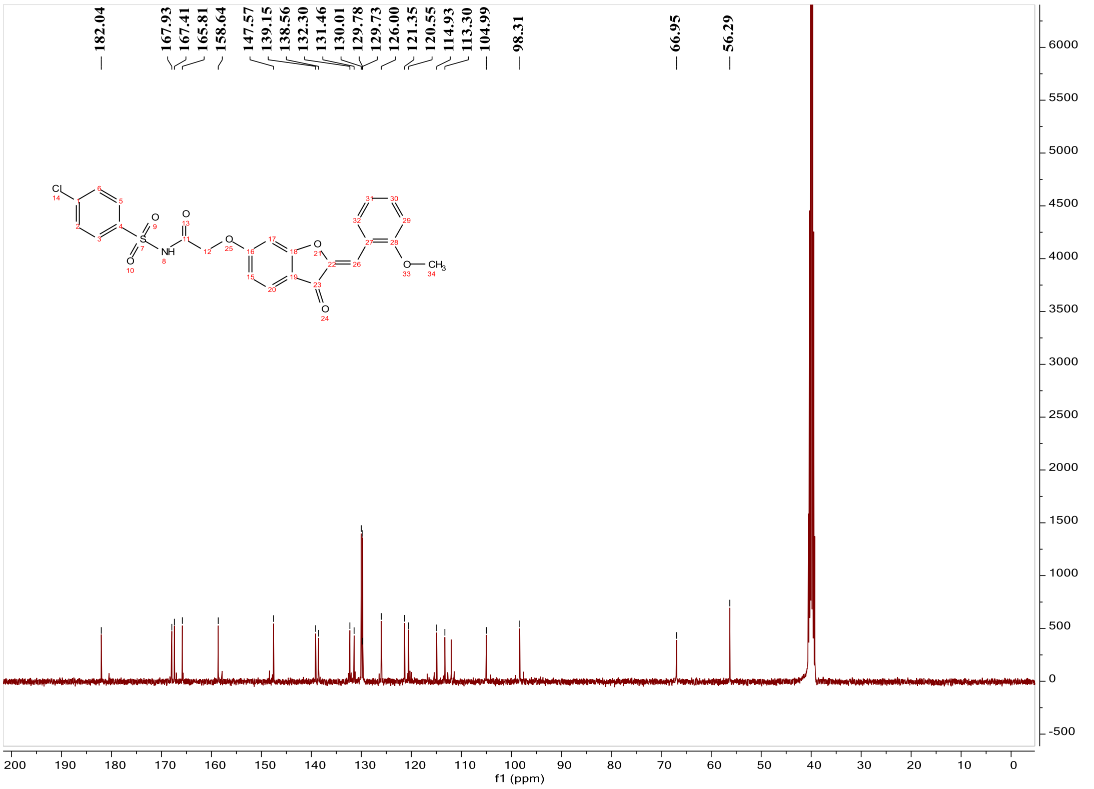

^{13}C NMR (101 MHz, DMSO-d_6) spectrum of compound **D3**

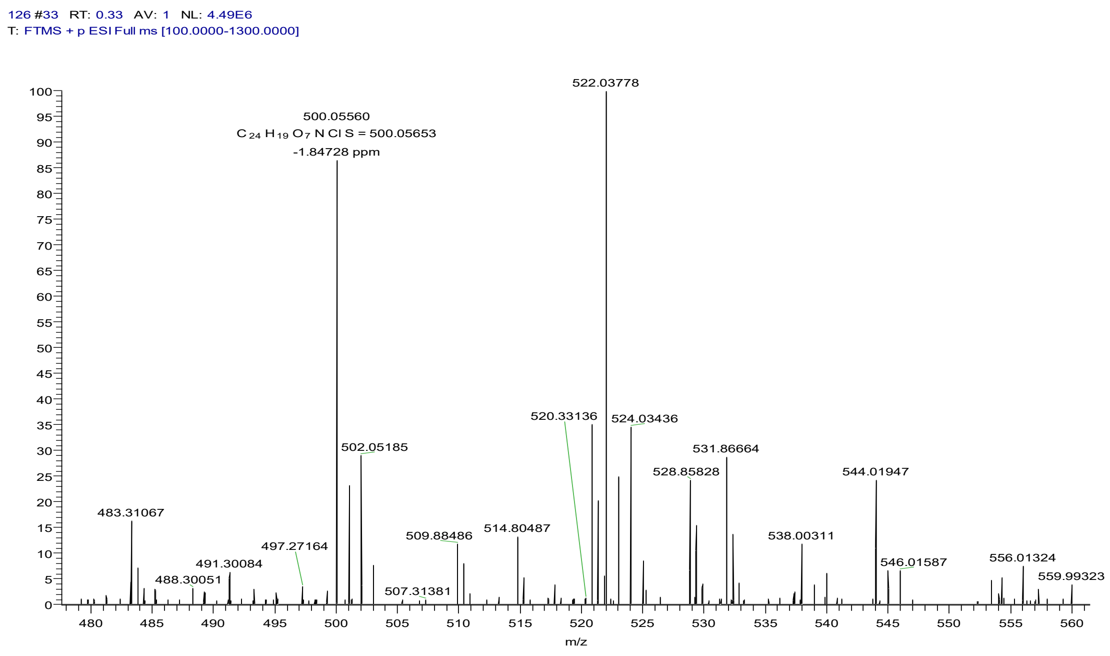

HRMS spectrum of compound **D3**

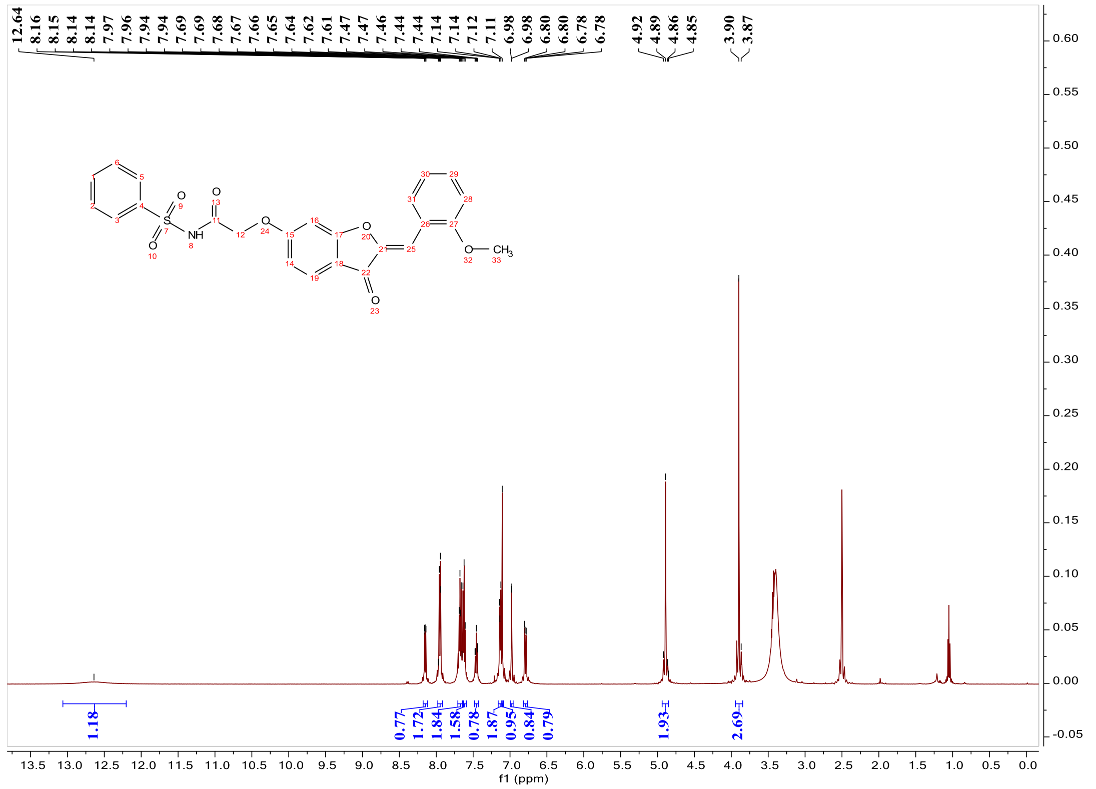

^{1}H NMR (500 MHz, DMSO-d_6) spectrum of compound **D4**

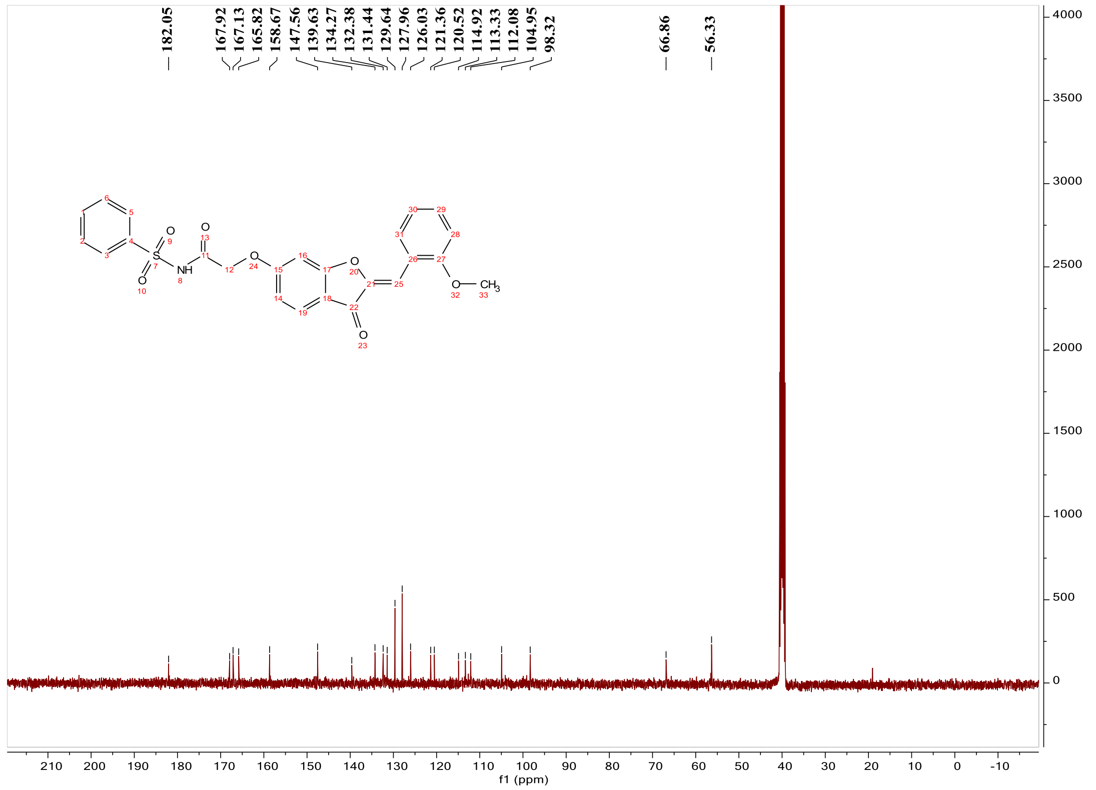

^{13}C NMR (101 MHz, DMSO-d_6) spectrum of compound **D4**

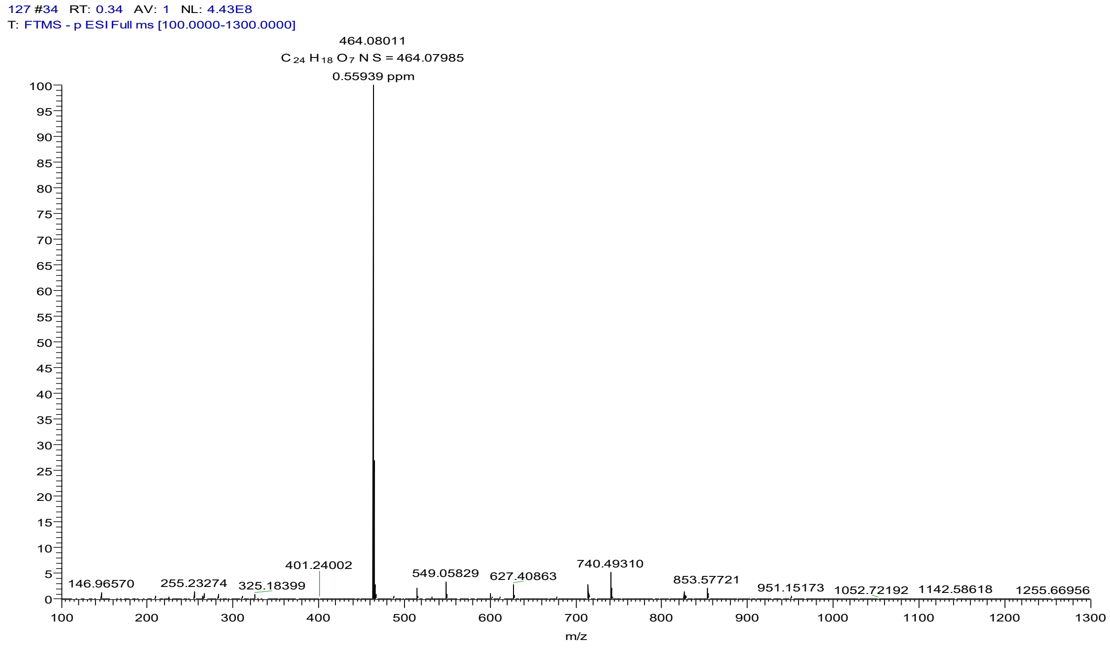

HRMS spectrum of compound **D4**

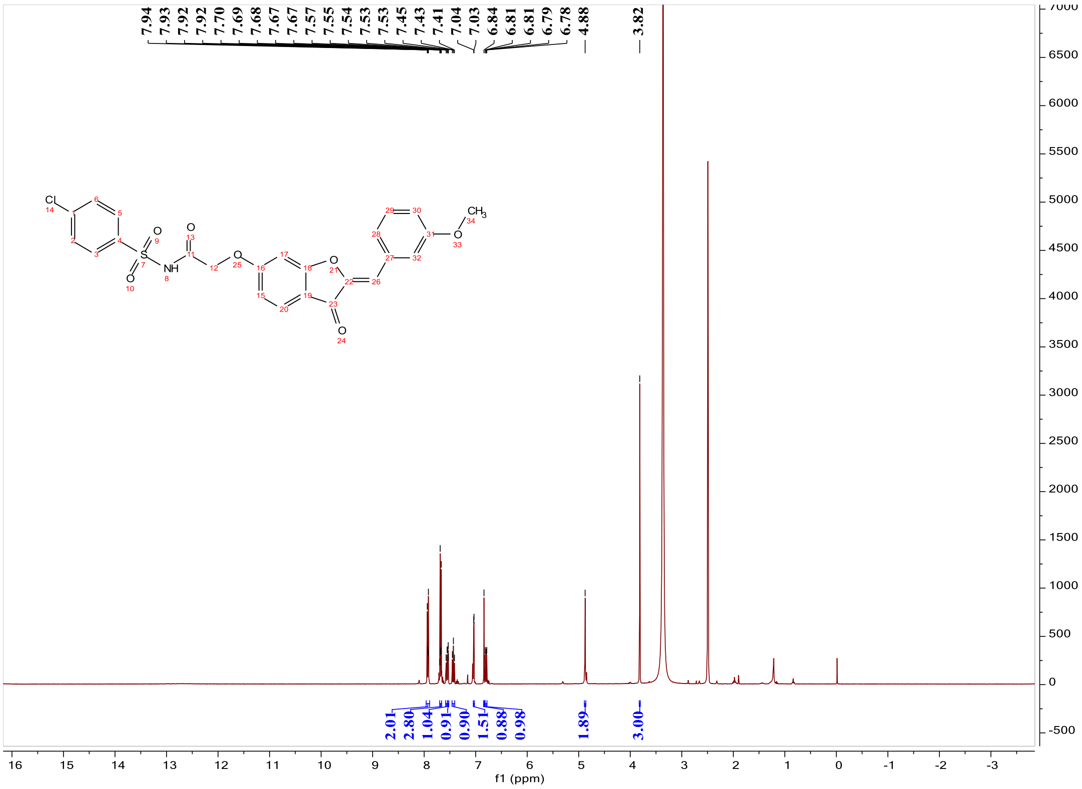

^{1}H NMR (400 MHz, DMSO-d_6) spectrum of compound **D5**

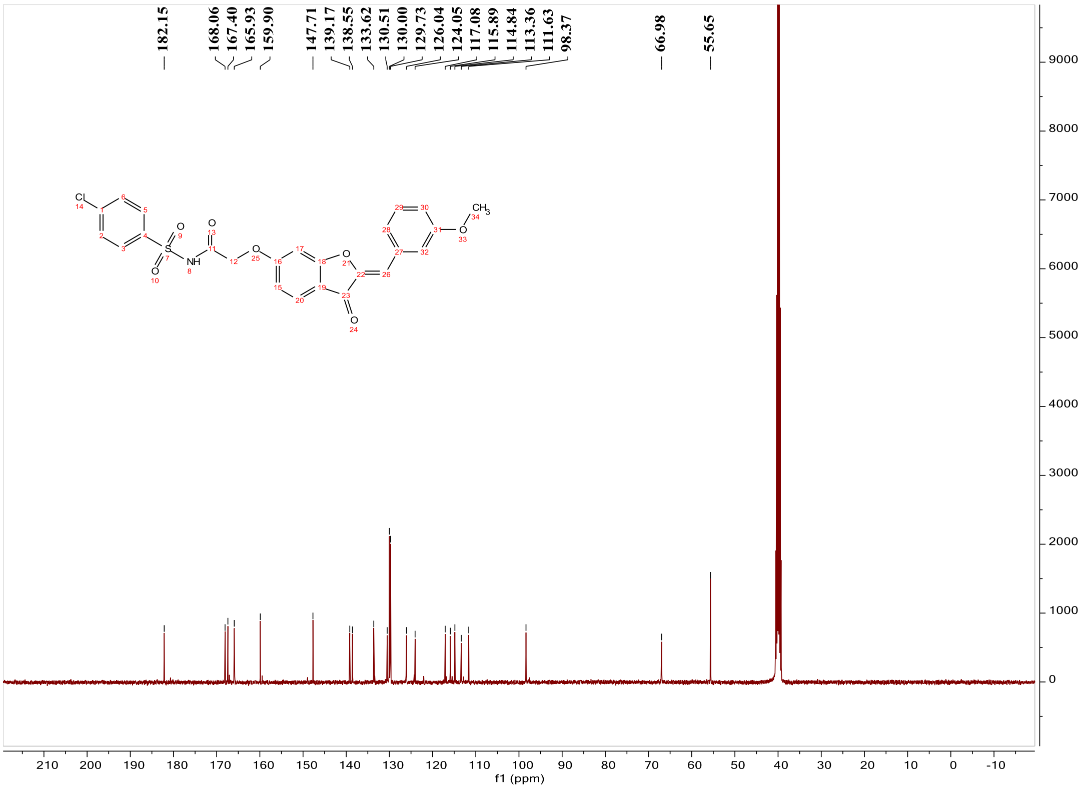

^{13}C NMR (101 MHz, DMSO-d_6) spectrum of compound **D5**

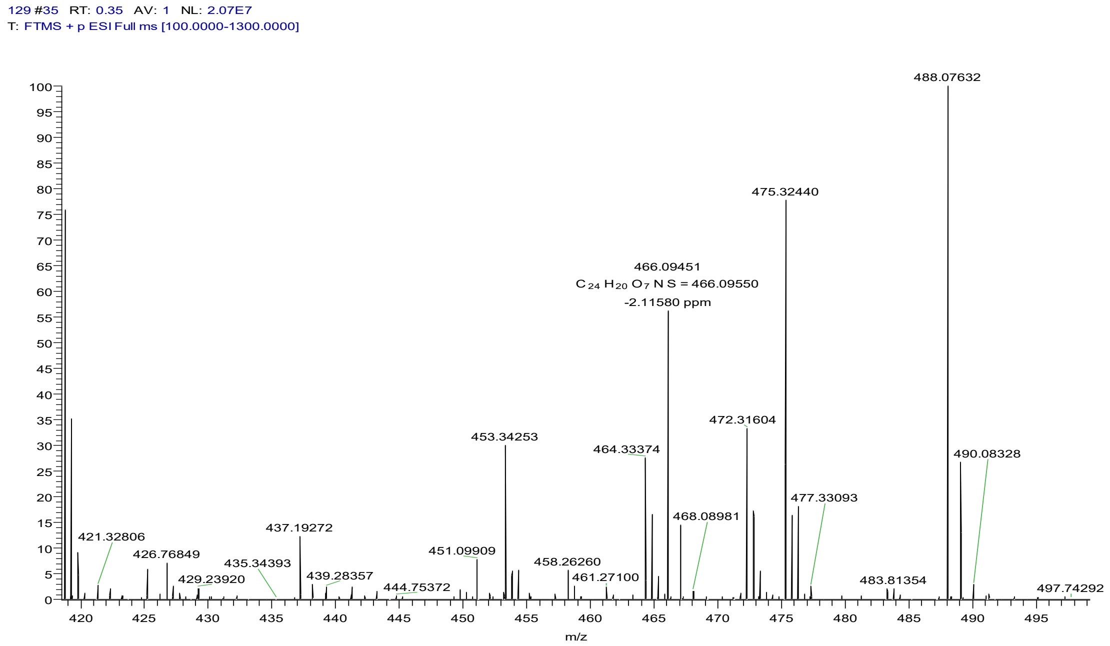

HRMS spectrum of compound **D5**

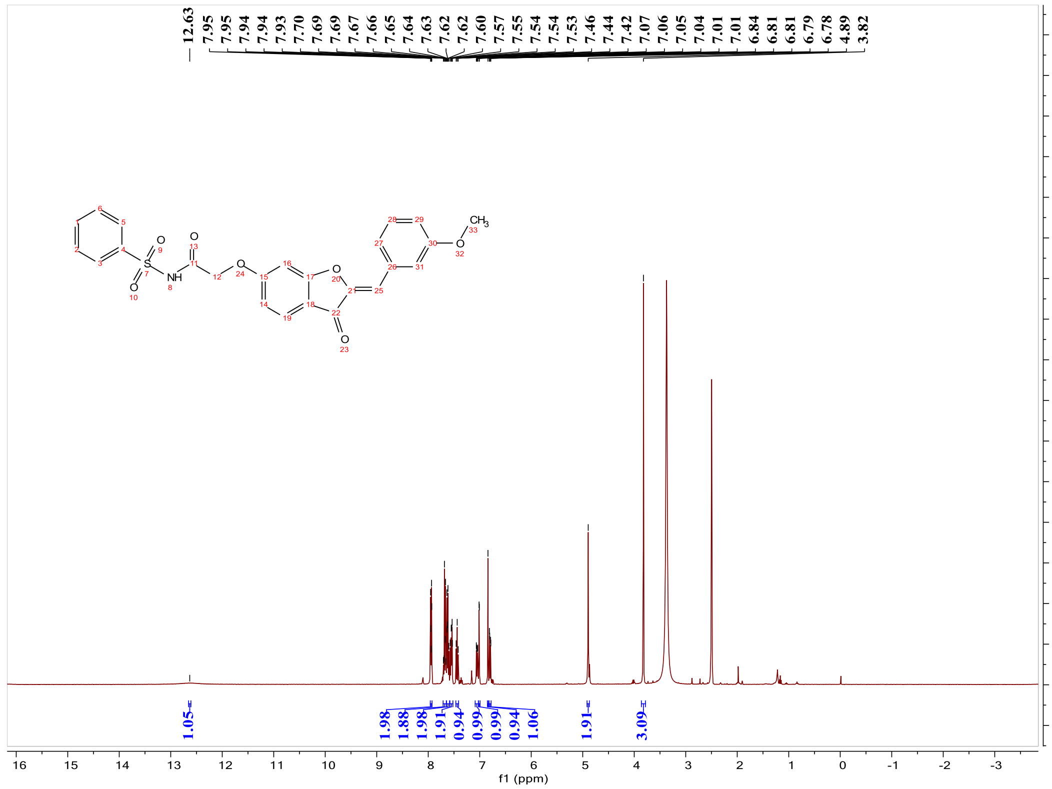

^{1}H NMR (400 MHz, DMSO-d_6) spectrum of compound **D6**

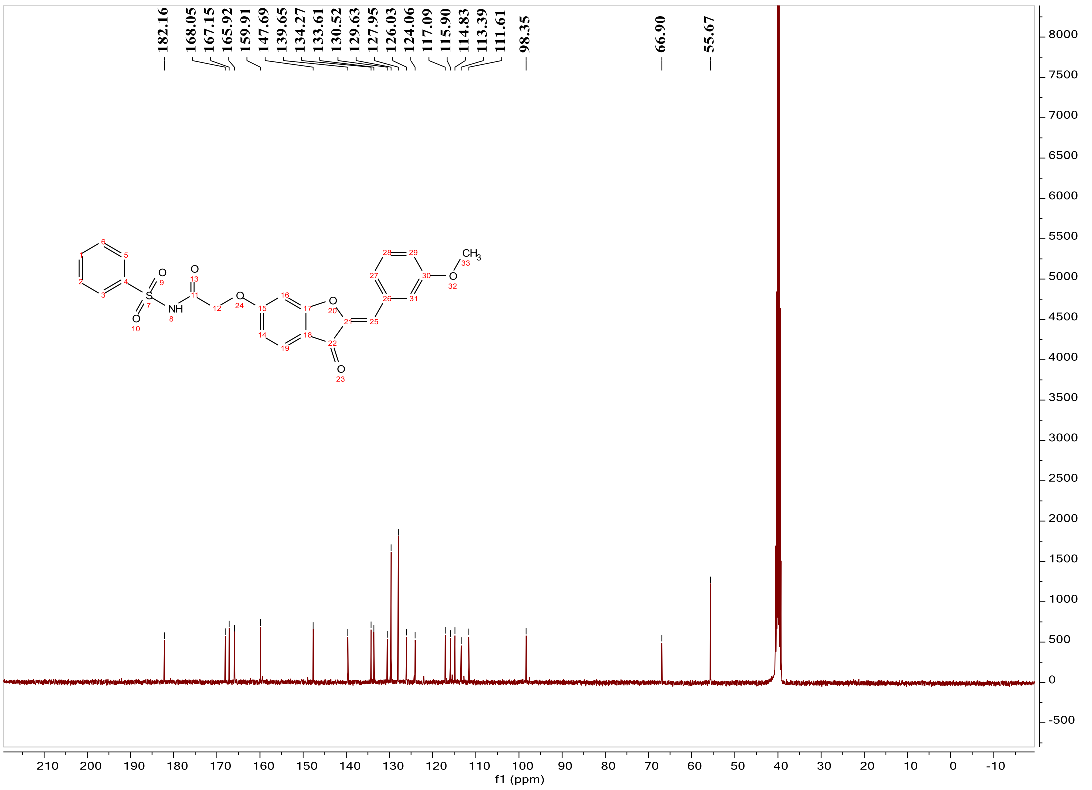

^{13}C NMR (101 MHz, DMSO-d_6) spectrum of compound **D6**

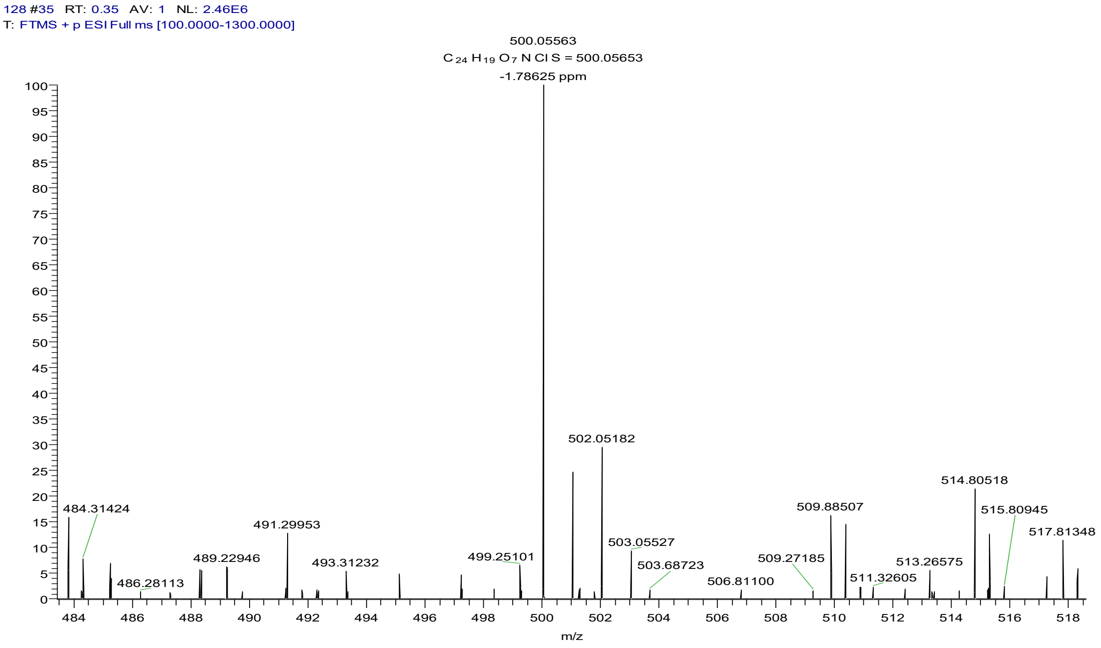

HRMS spectrum of compound **D6**

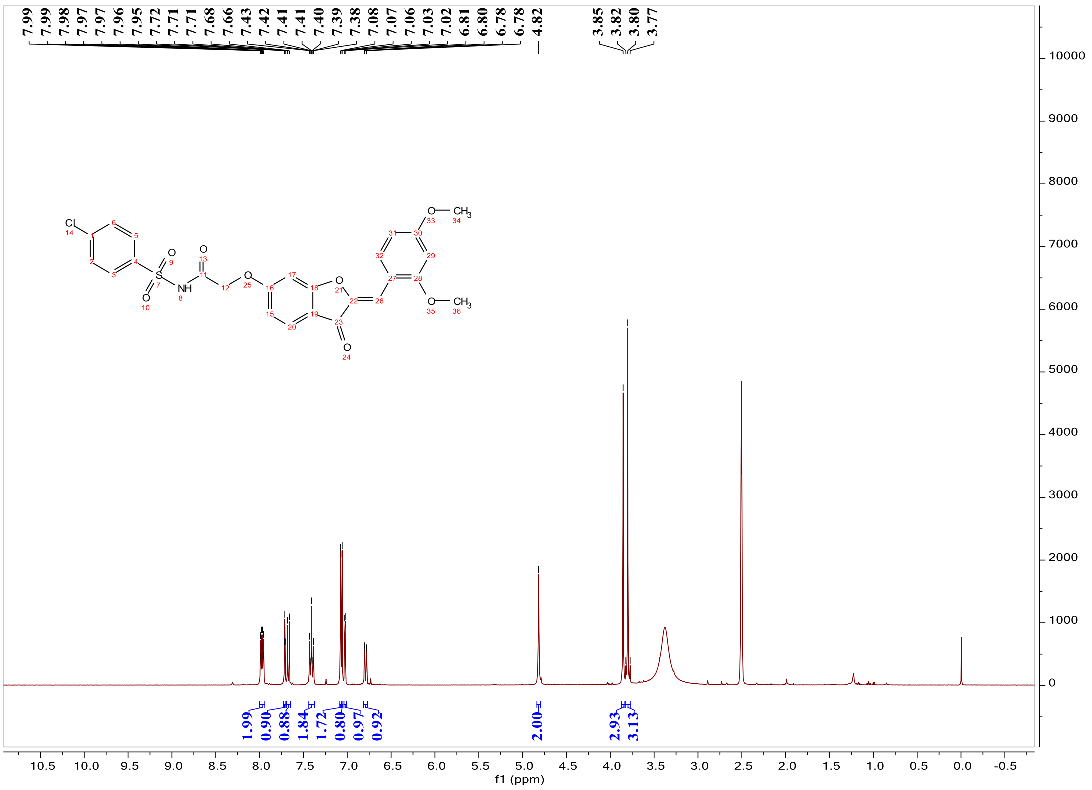

^{1}H NMR (400 MHz, DMSO-d_6) spectrum of compound **D7**

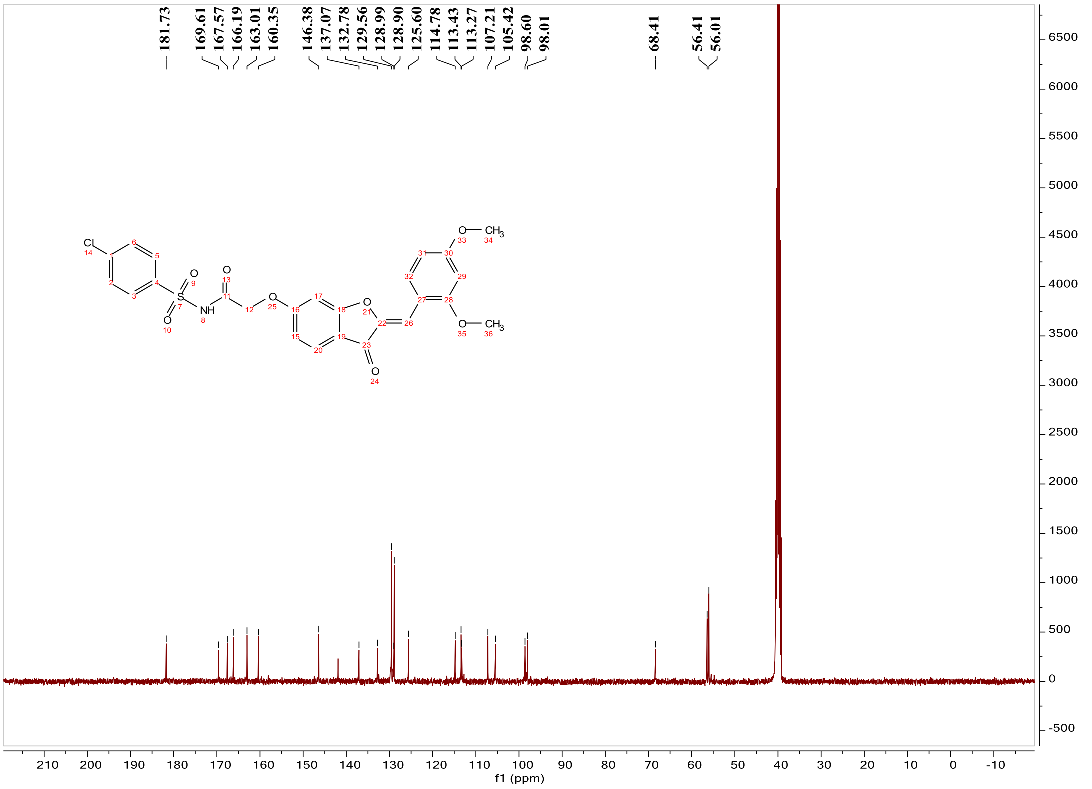

^{13}C NMR (101 MHz, DMSO-d_6) spectrum of compound **D7**

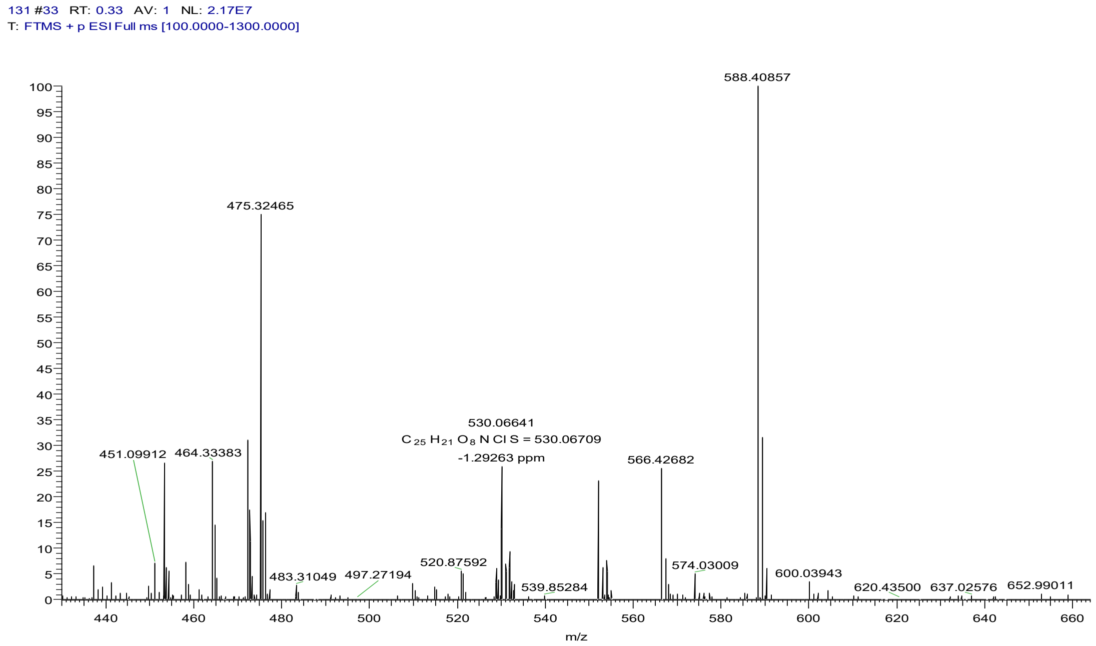

HRMS spectrum of compound **D7**

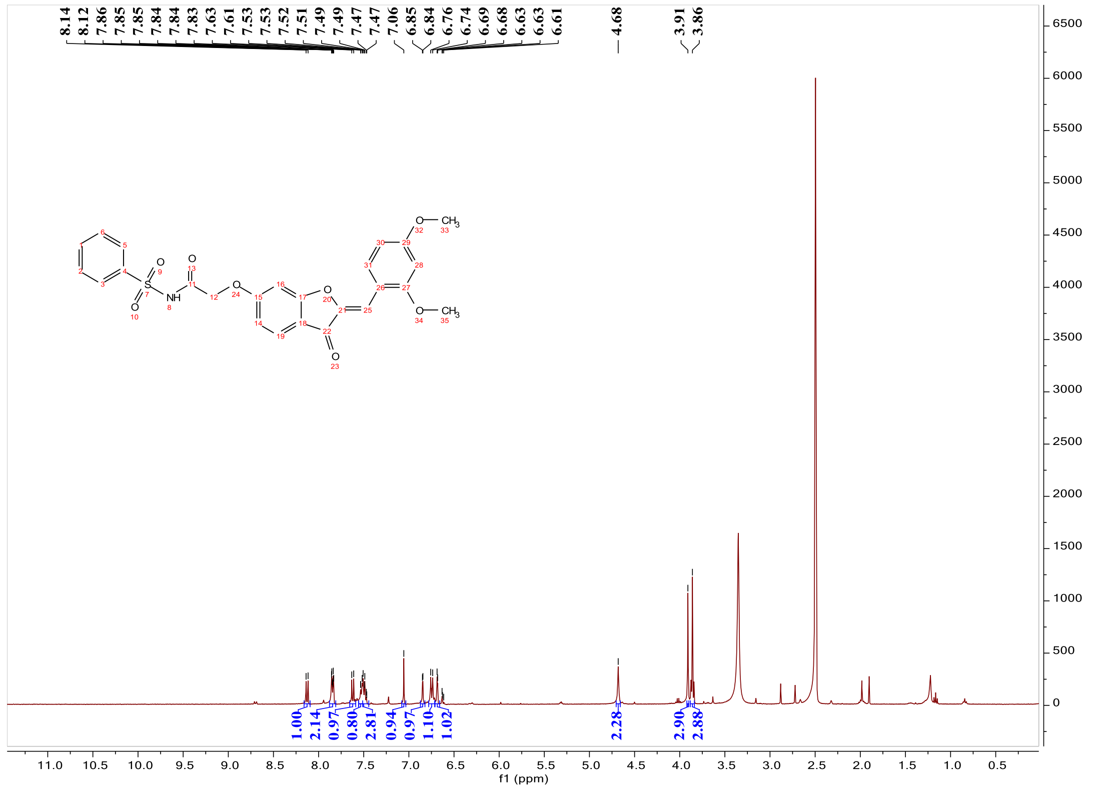

^{1}H NMR (400 MHz, DMSO-d_6) spectrum of compound **D8**

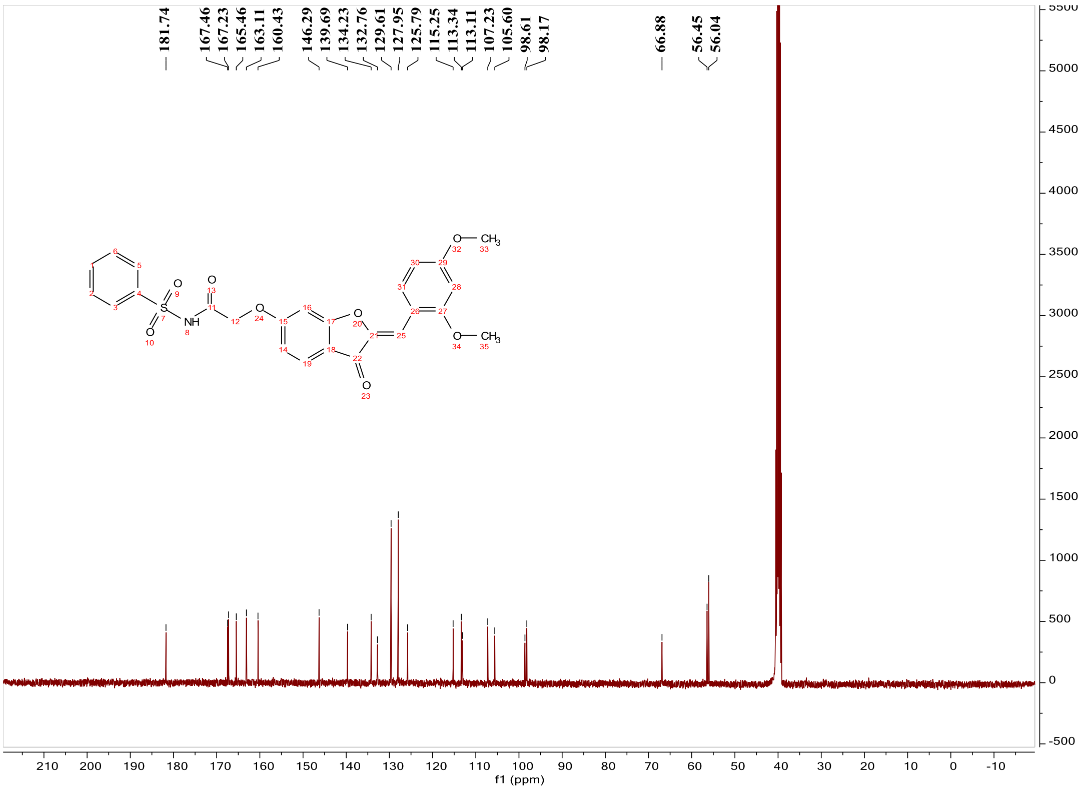

^{13}C NMR (101 MHz, DMSO-d_6) spectrum of compound **D8**

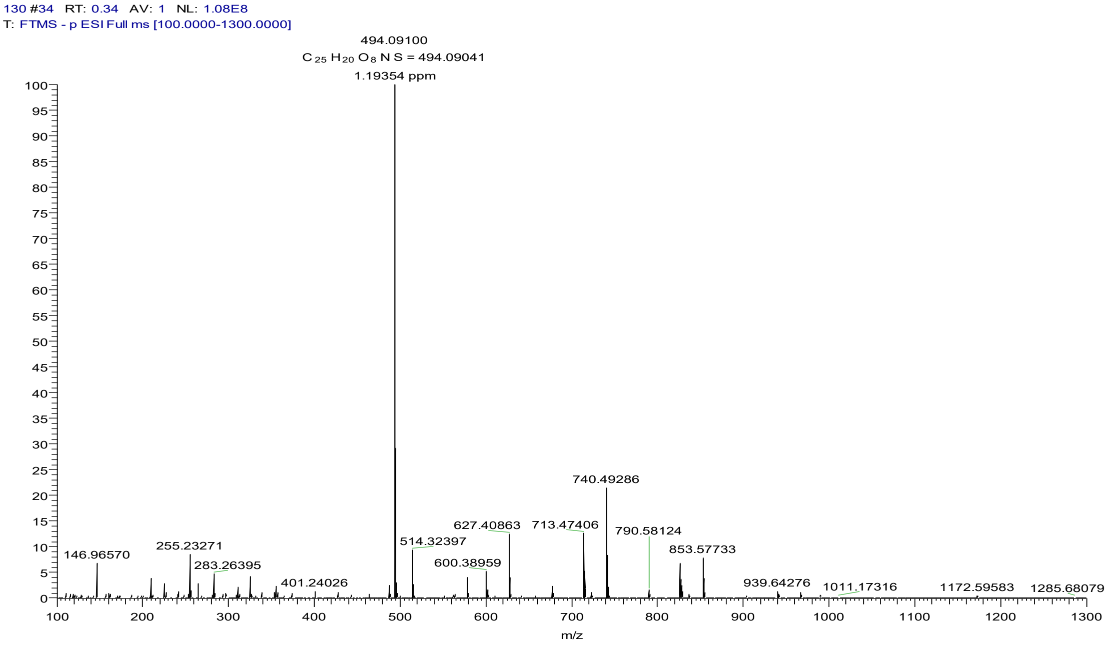

HRMS spectrum of compound **D8**

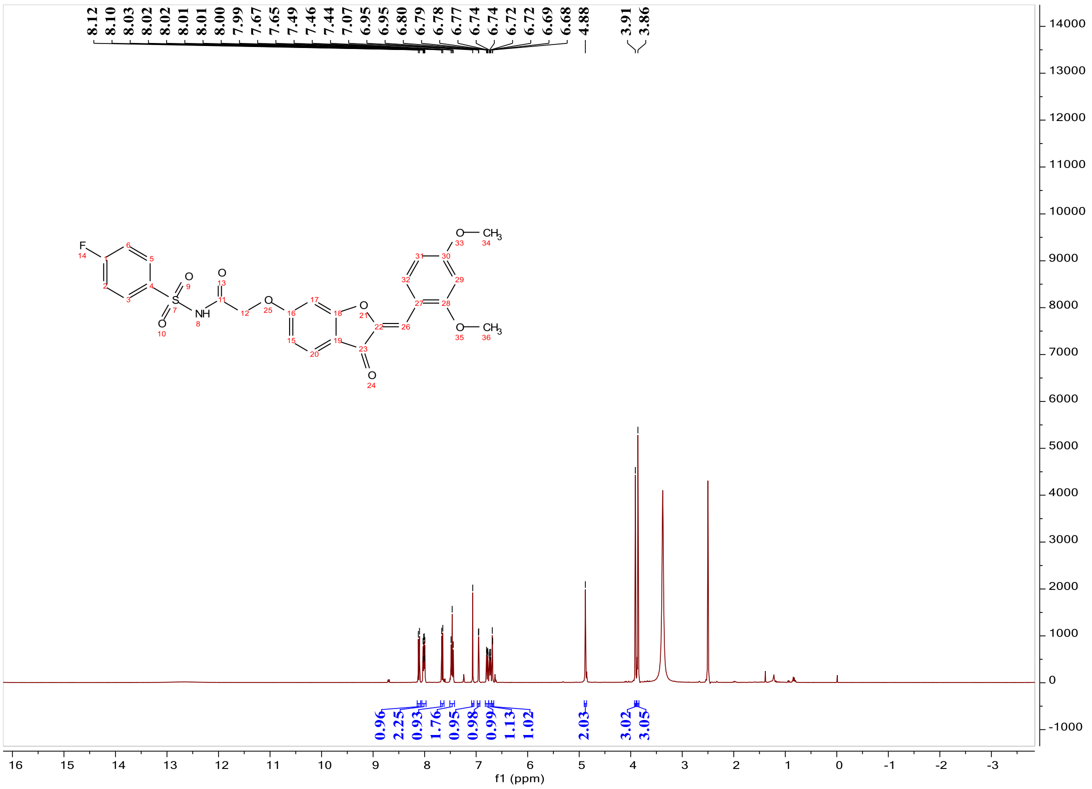

^{1}H NMR (400 MHz, DMSO-d_6) spectrum of compound **D9**

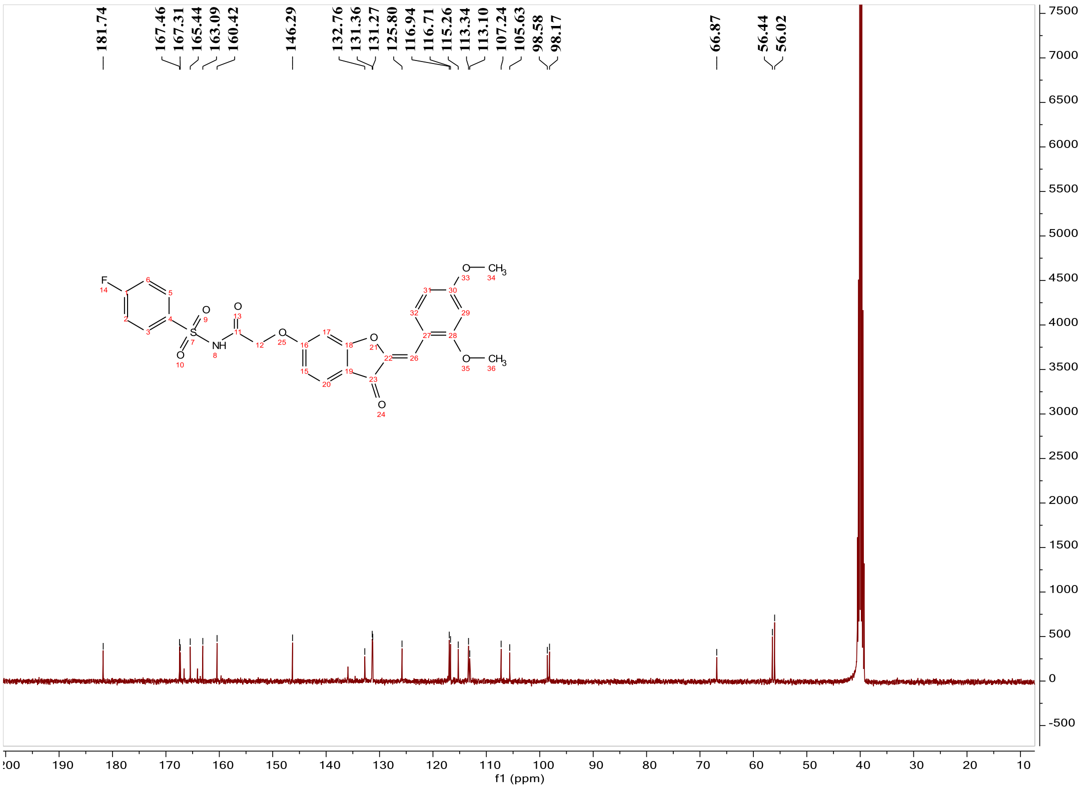

^{13}C NMR (101 MHz, DMSO-d_6) spectrum of compound **D9**

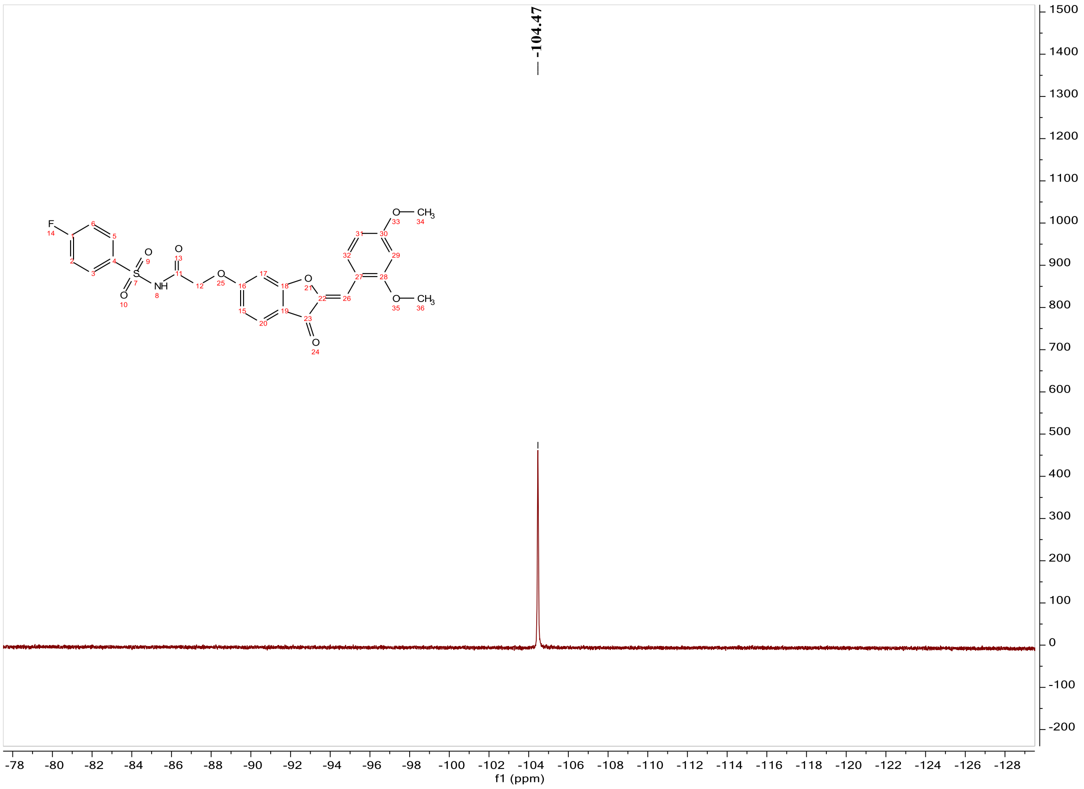

^{19}F NMR (376 MHz, DMSO-d_6) spectrum of compound **D9**

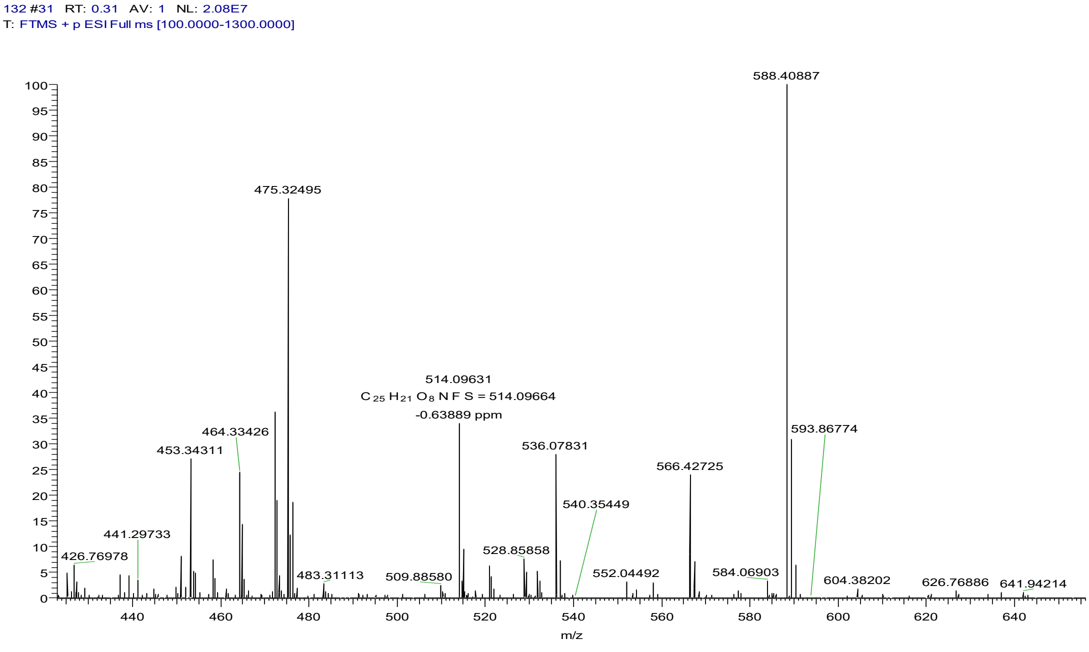

HRMS spectrum of compound **D9**

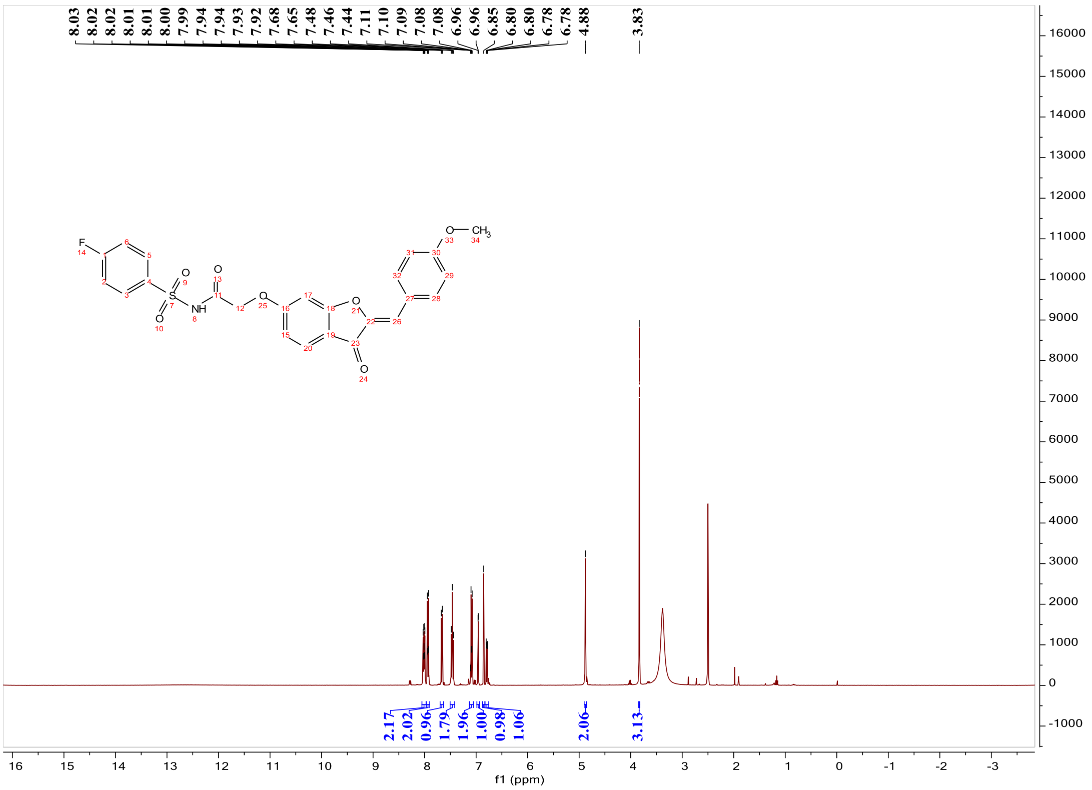

^{1}H NMR (400 MHz, DMSO-d_6) spectrum of compound **D10**

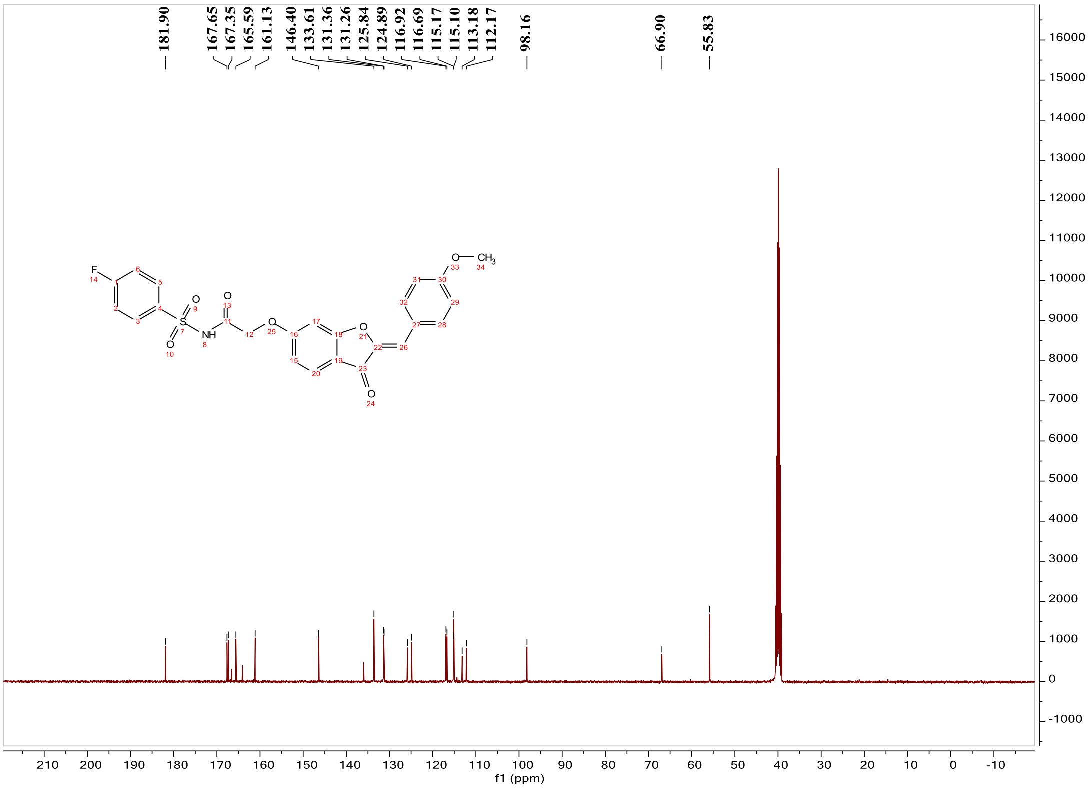

^{13}C NMR (101 MHz, DMSO-d_6) spectrum of compound **D10**

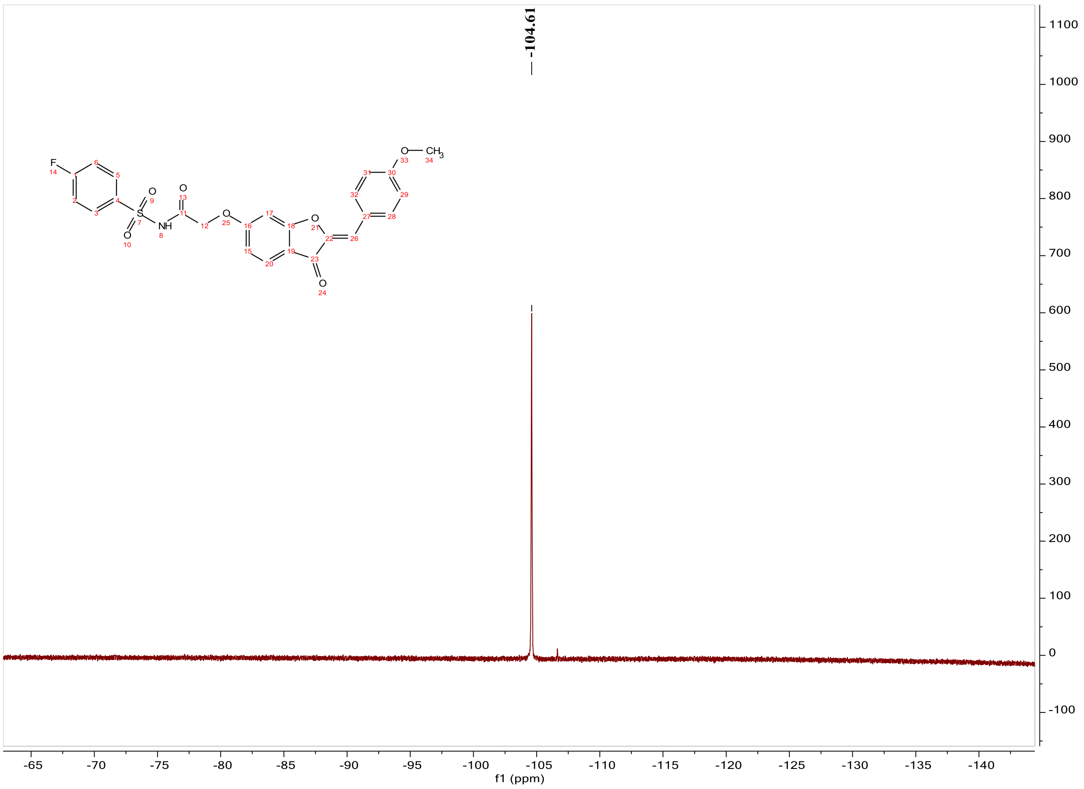

^{19}F NMR (376 MHz, DMSO-d_6) spectrum of compound **D10**

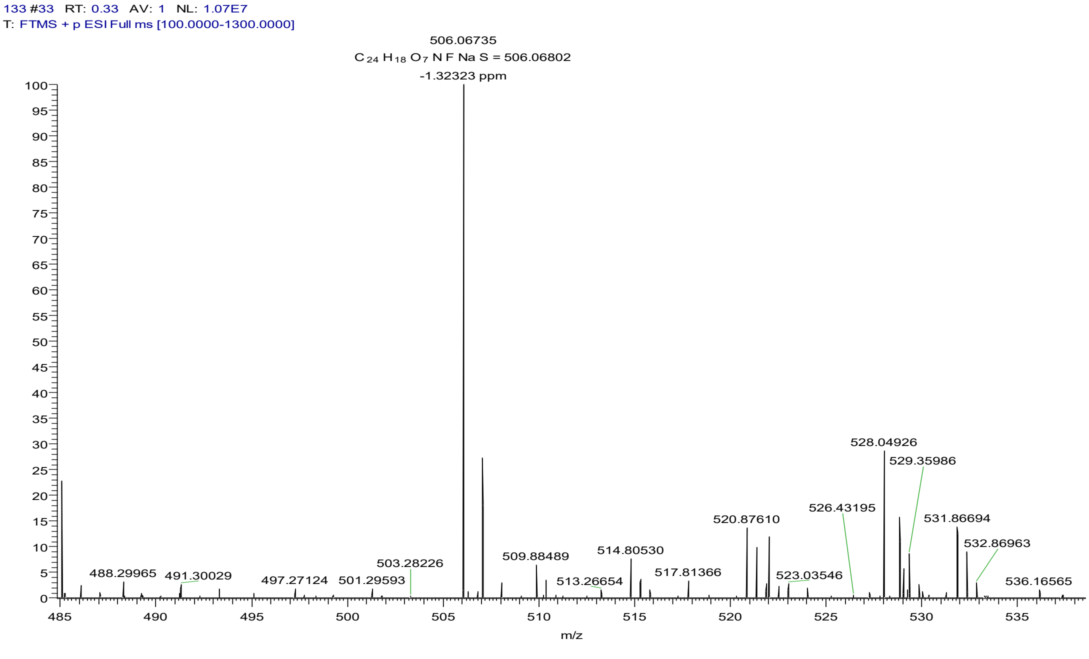

HRMS spectrum of compound **D10**

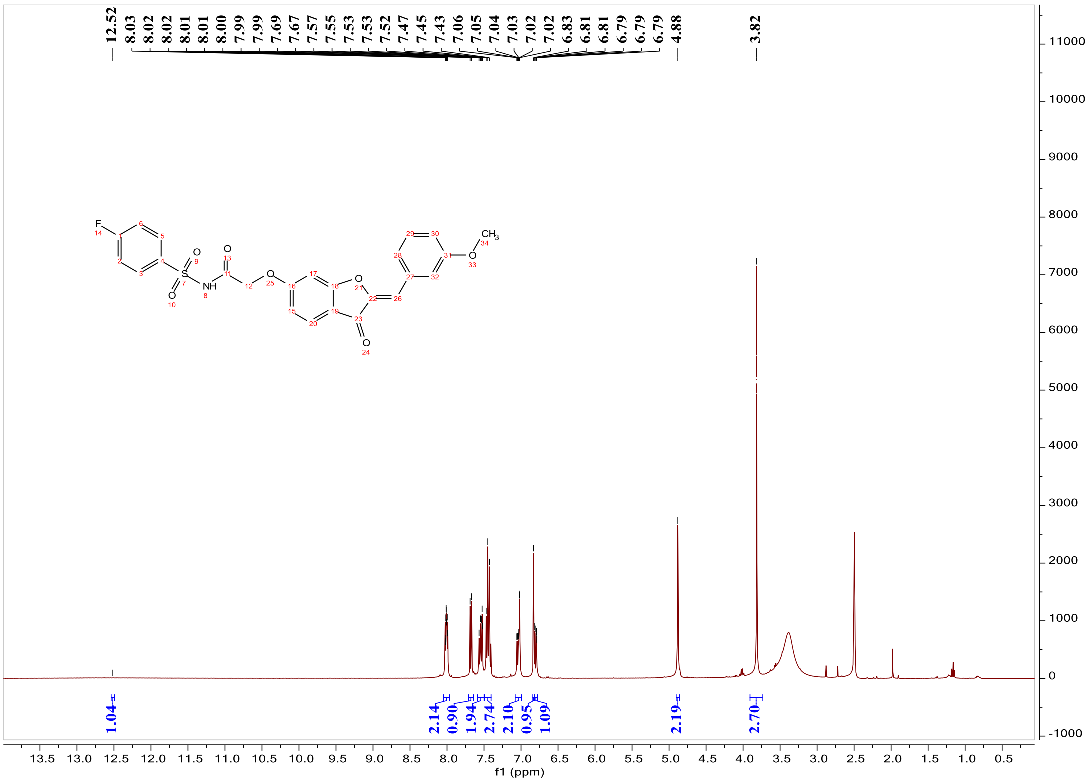

^{1}H NMR (400 MHz, DMSO-d_6) spectrum of compound **D11**

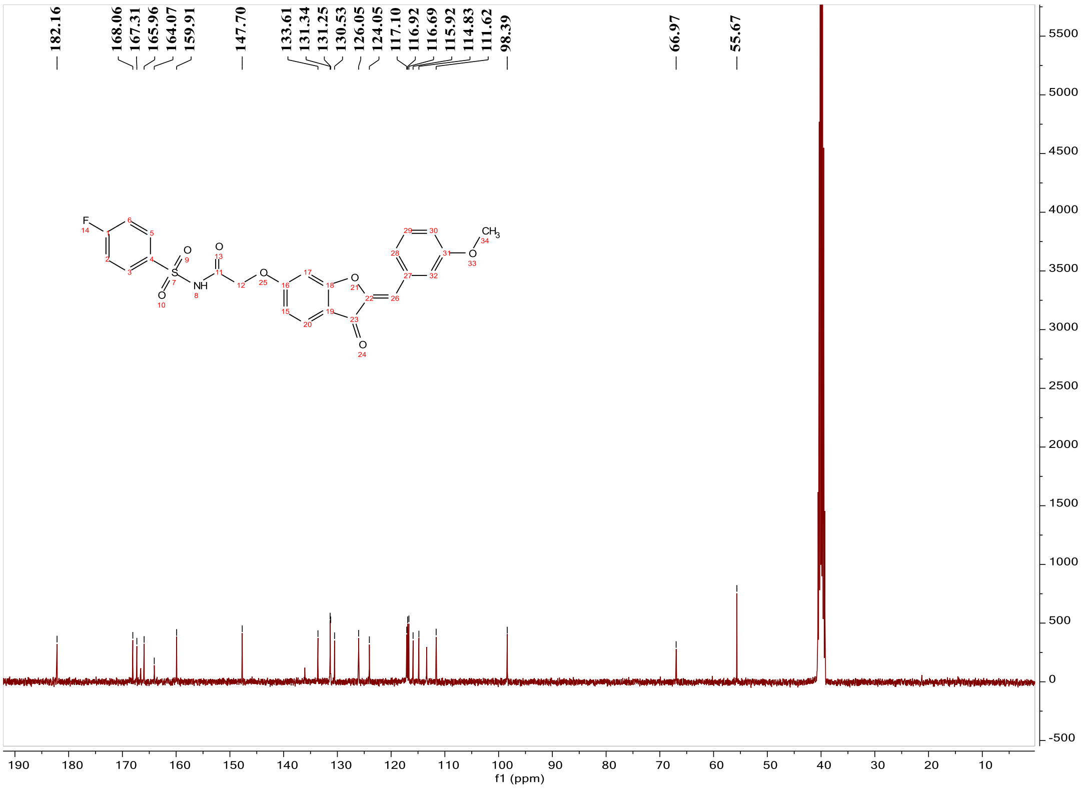

^{13}C NMR (101 MHz, DMSO-d_6) spectrum of compound **D11**

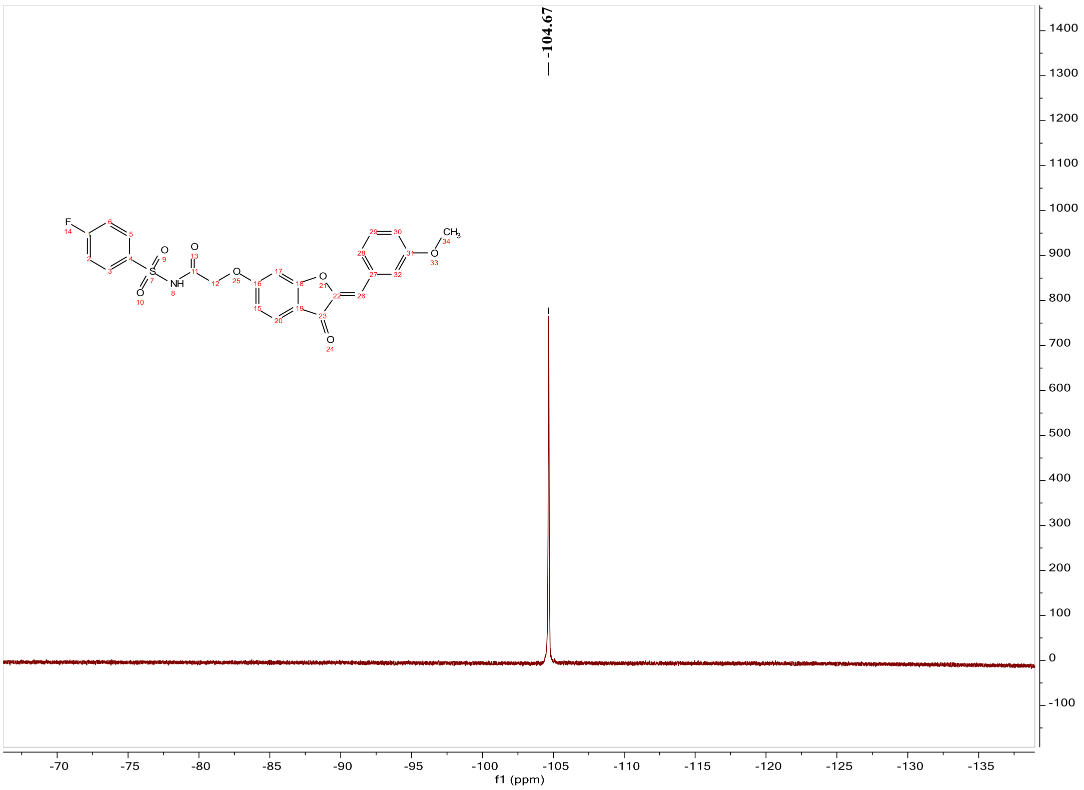

^{19}F NMR (376 MHz, DMSO-d_6) spectrum of compound **D11**

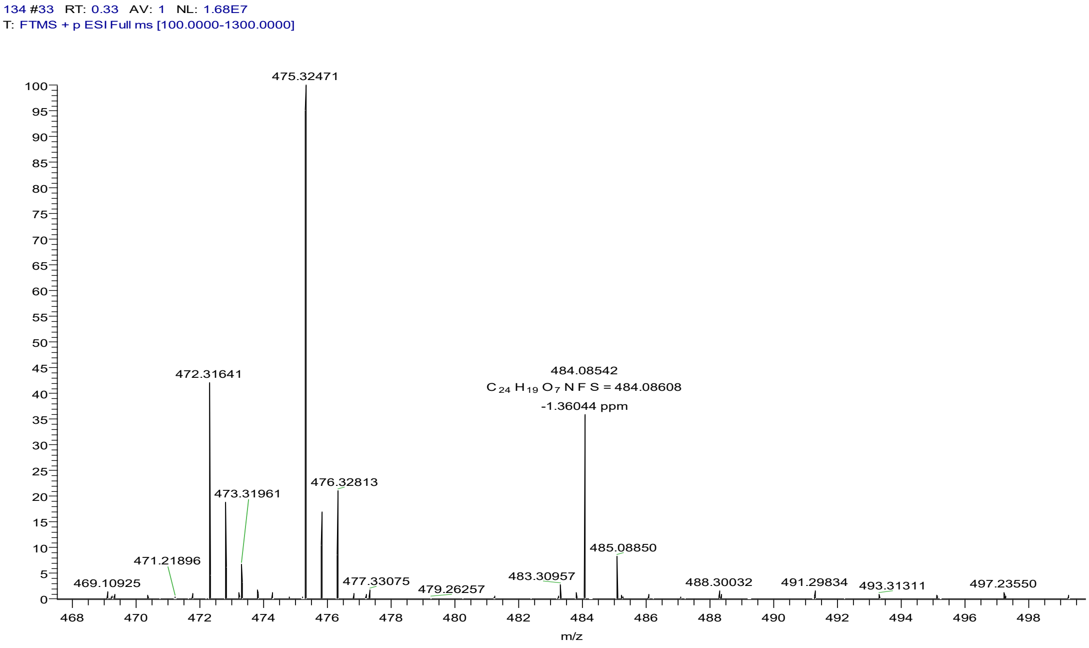

HRMS spectrum of compound **D11**

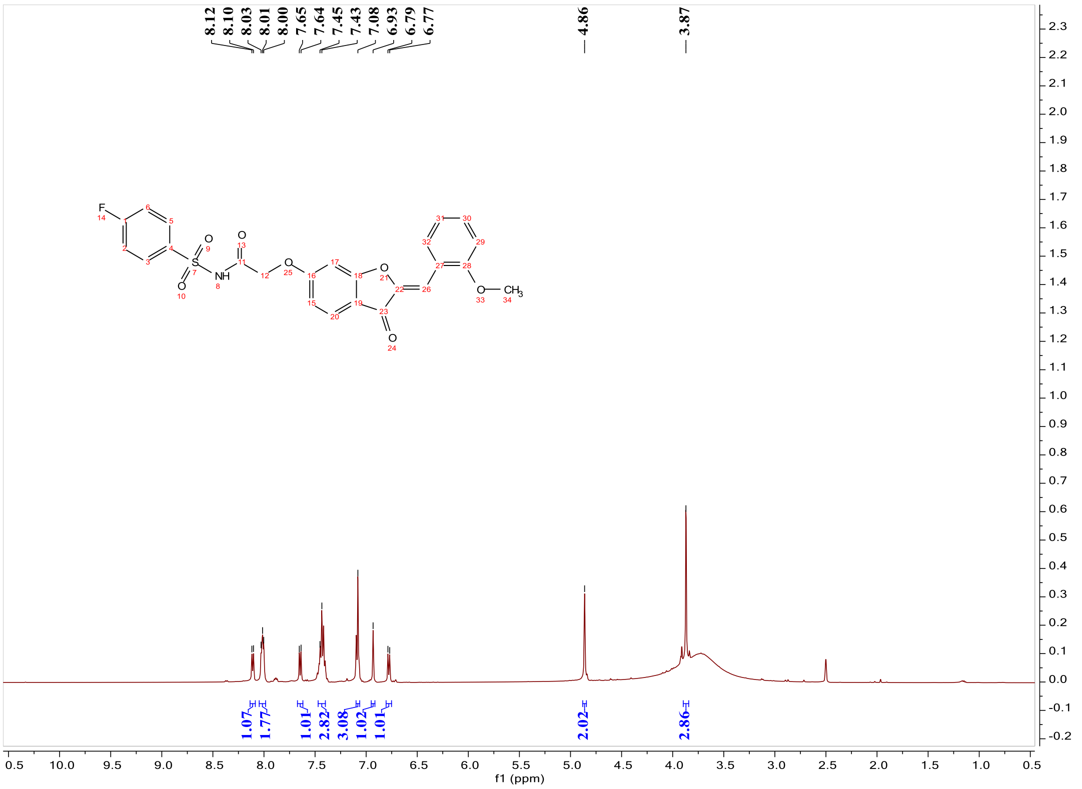

^{1}H NMR (500 MHz, DMSO-d_6) spectrum of compound **D12**

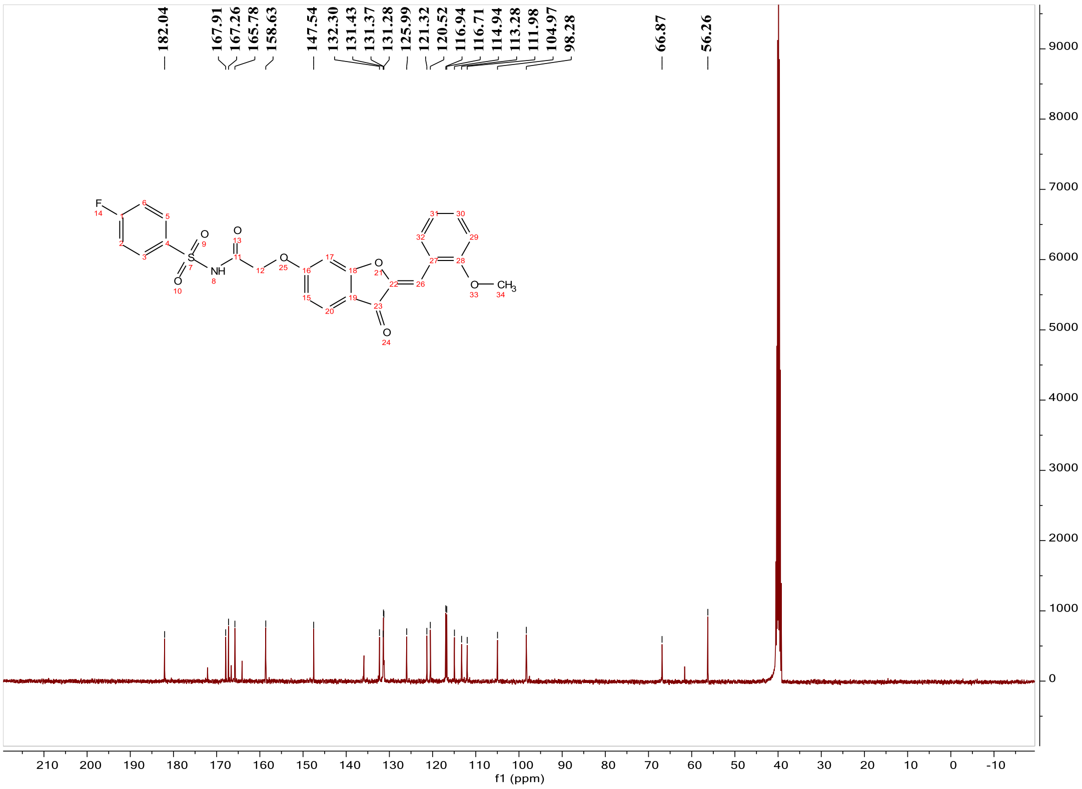

^{13}C NMR (101 MHz, DMSO-d_6) spectrum of compound **D12**

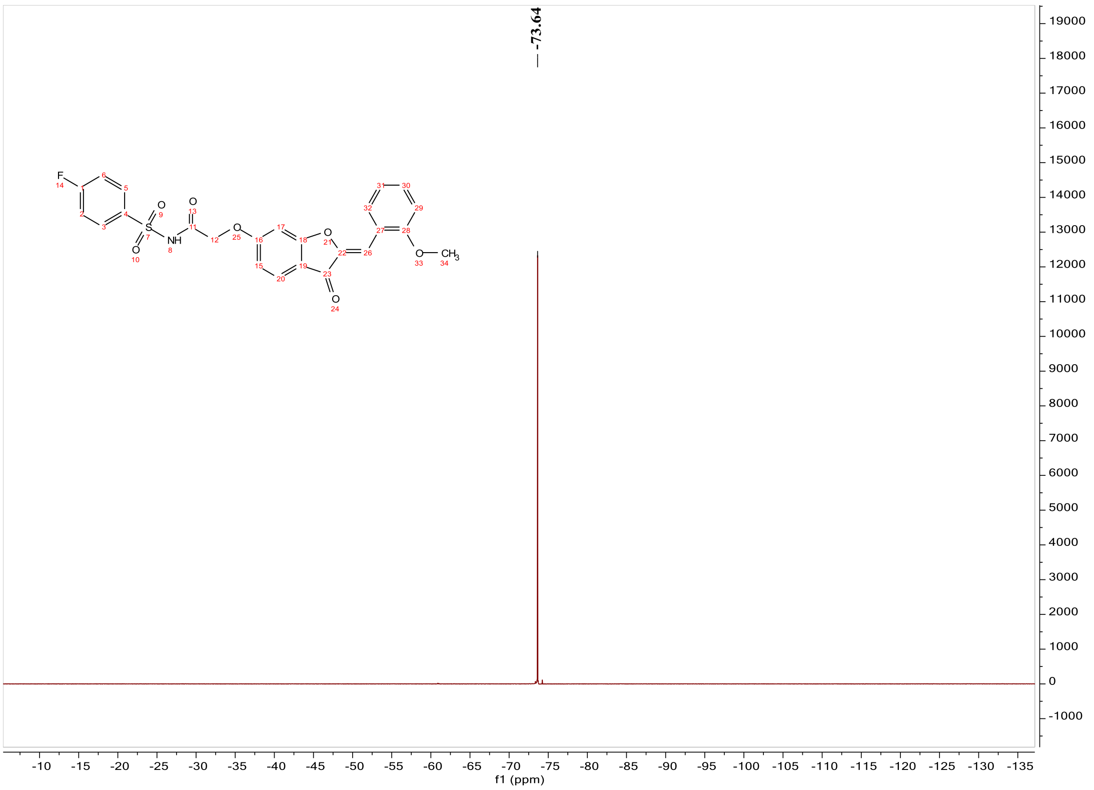

^{19}F NMR (376 MHz, DMSO-d_6) spectrum of compound **D12**

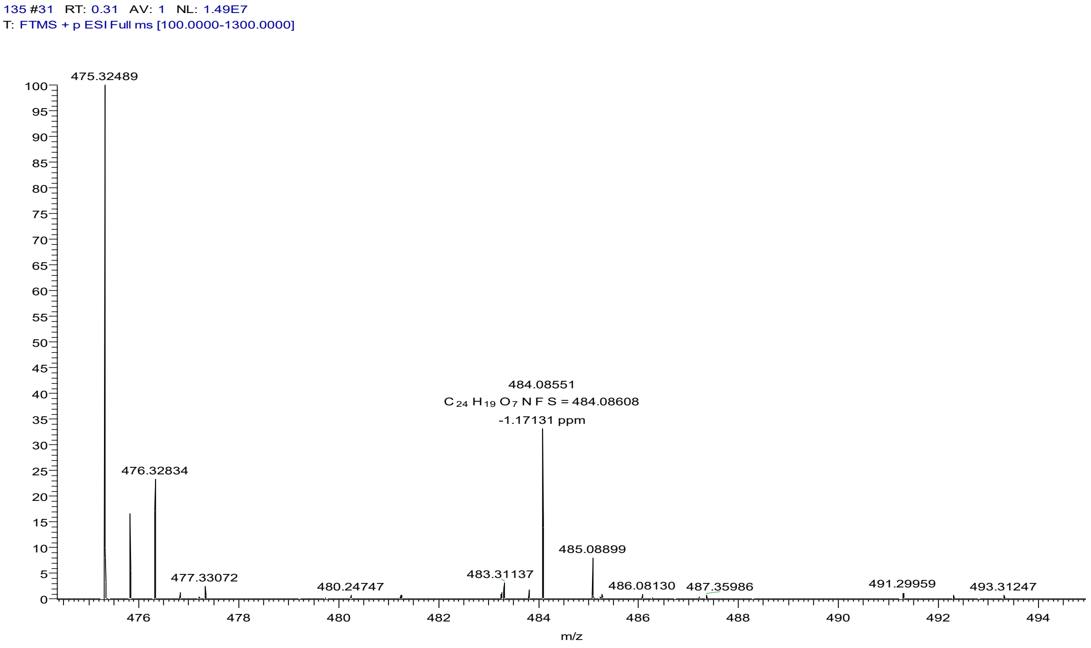

HRMS spectrum of compound **D12**

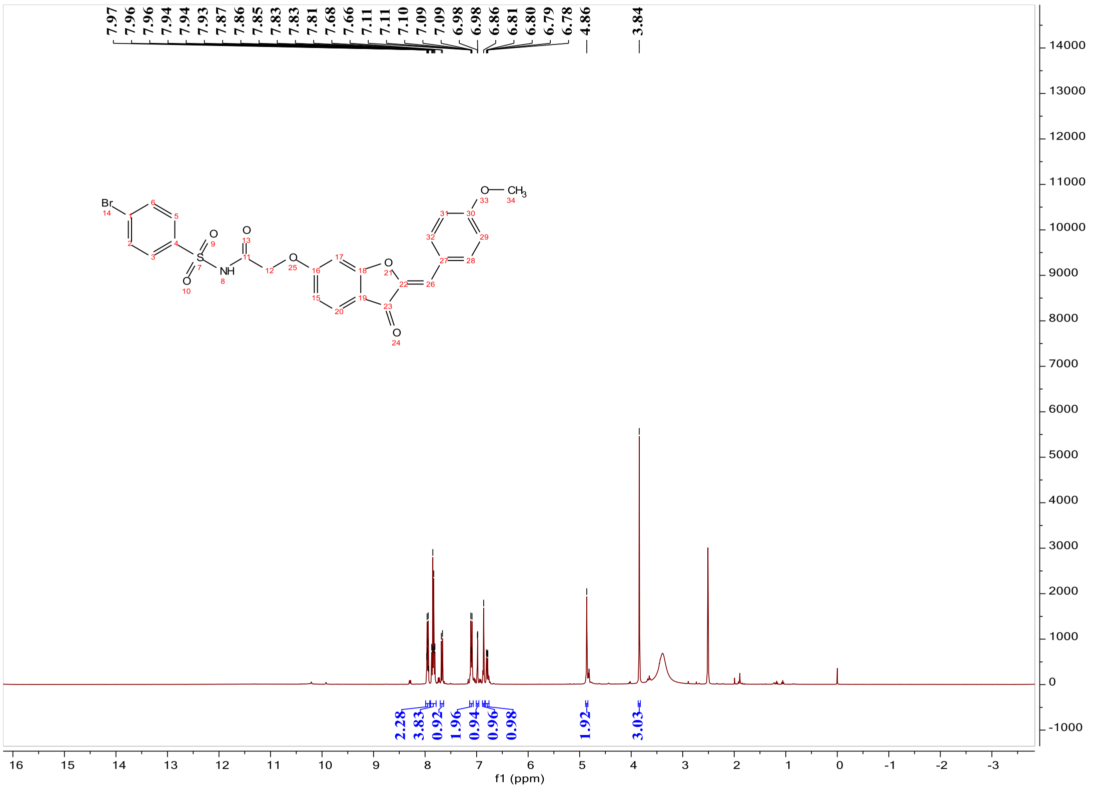

^{1}H NMR (400 MHz, DMSO-d_6) spectrum of compound **D13**

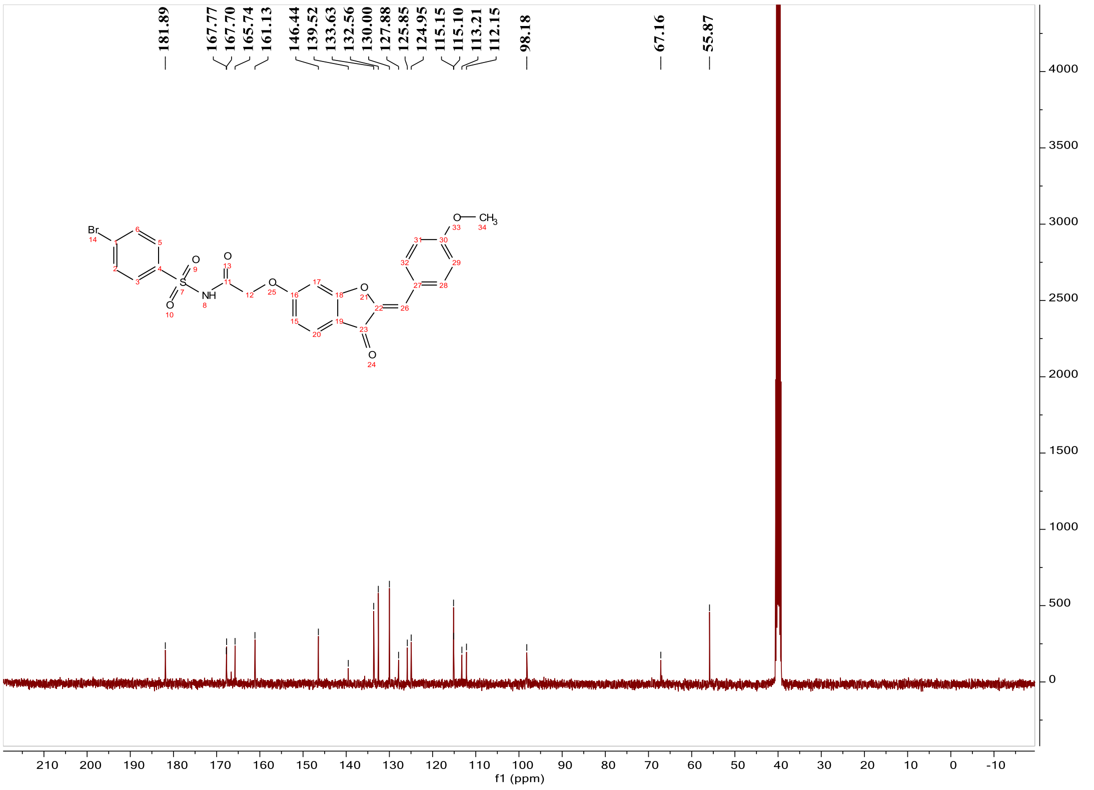

^{13}C NMR (101 MHz, DMSO-d_6) spectrum of compound **D13**

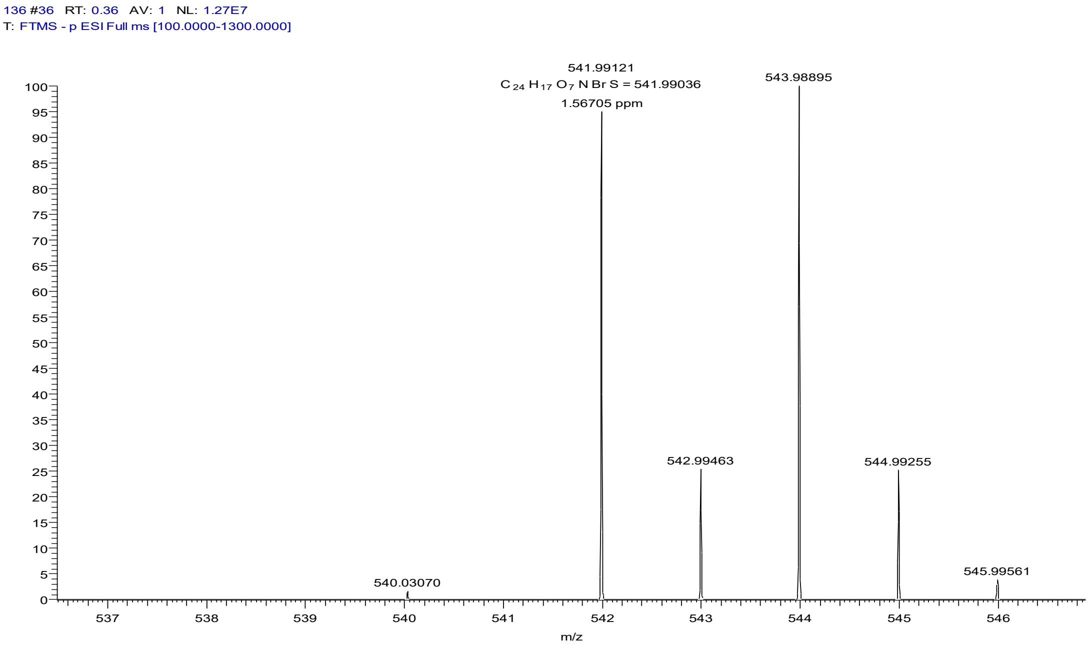

HRMS spectrum of compound **D13**

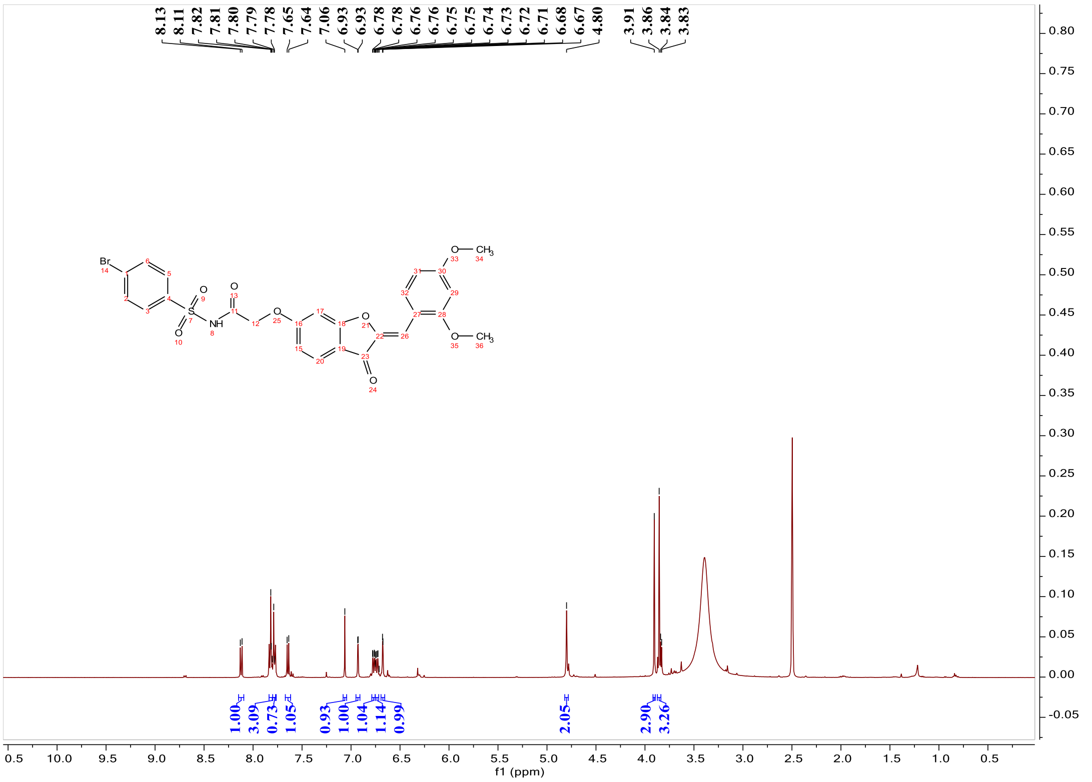

^{1}H NMR (500 MHz, DMSO-d_6) spectrum of compound **D14**

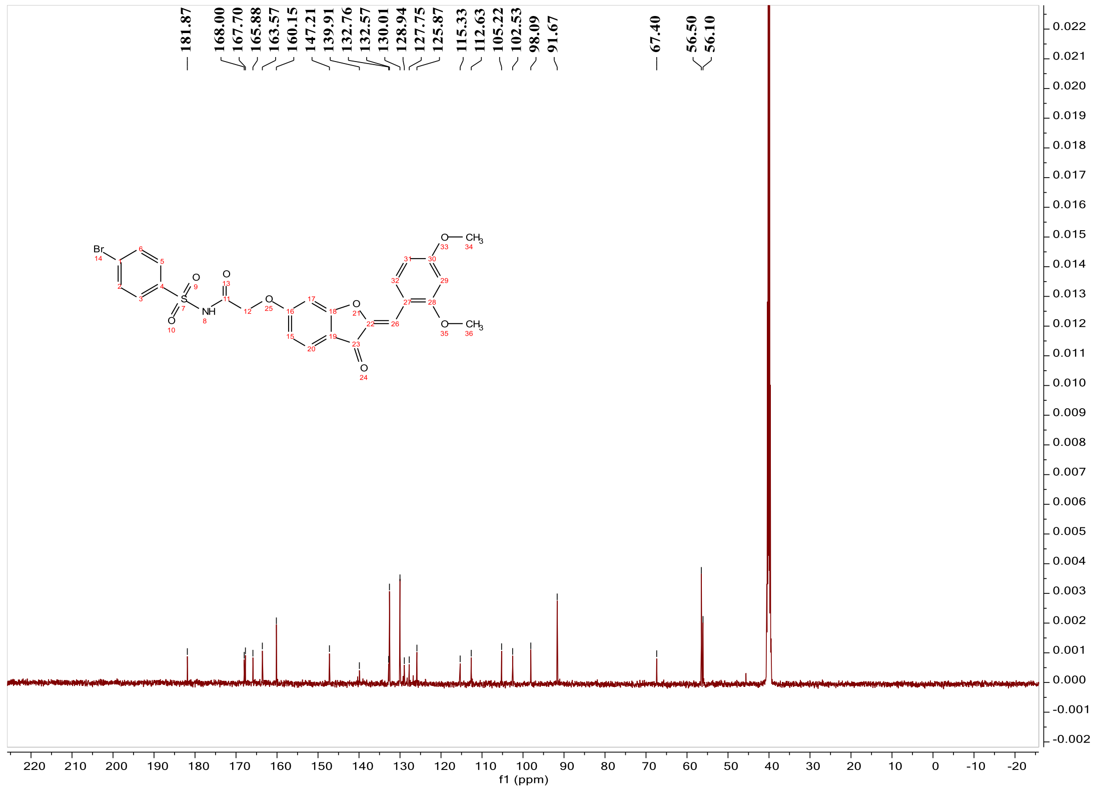

^{13}C NMR (126 MHz, DMSO-d_6) spectrum of compound **D14**

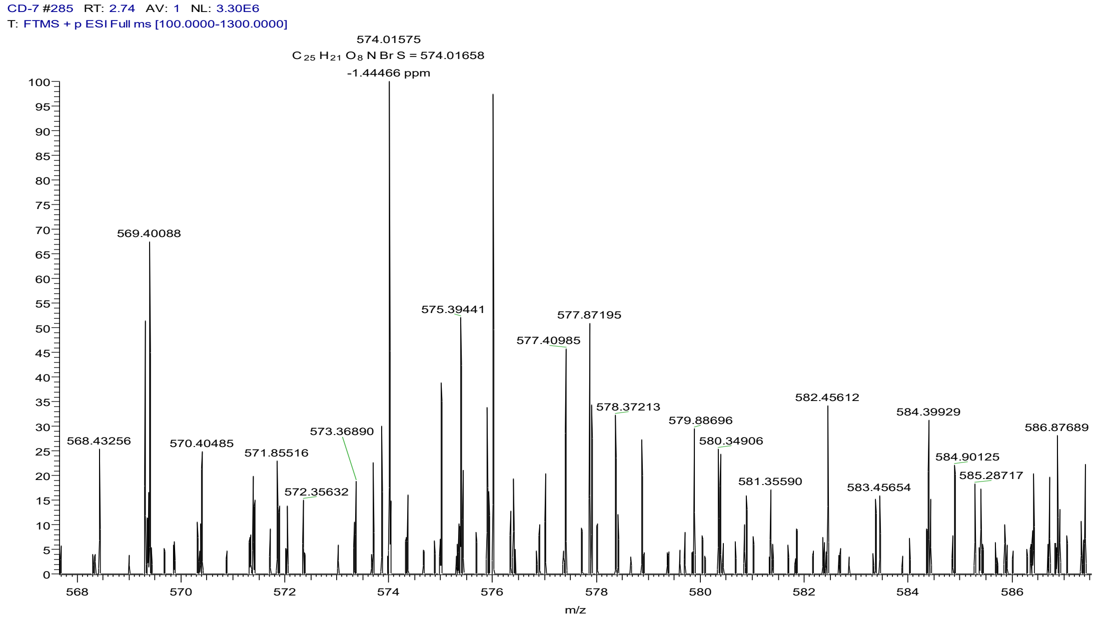

HRMS spectrum of compound **D14**

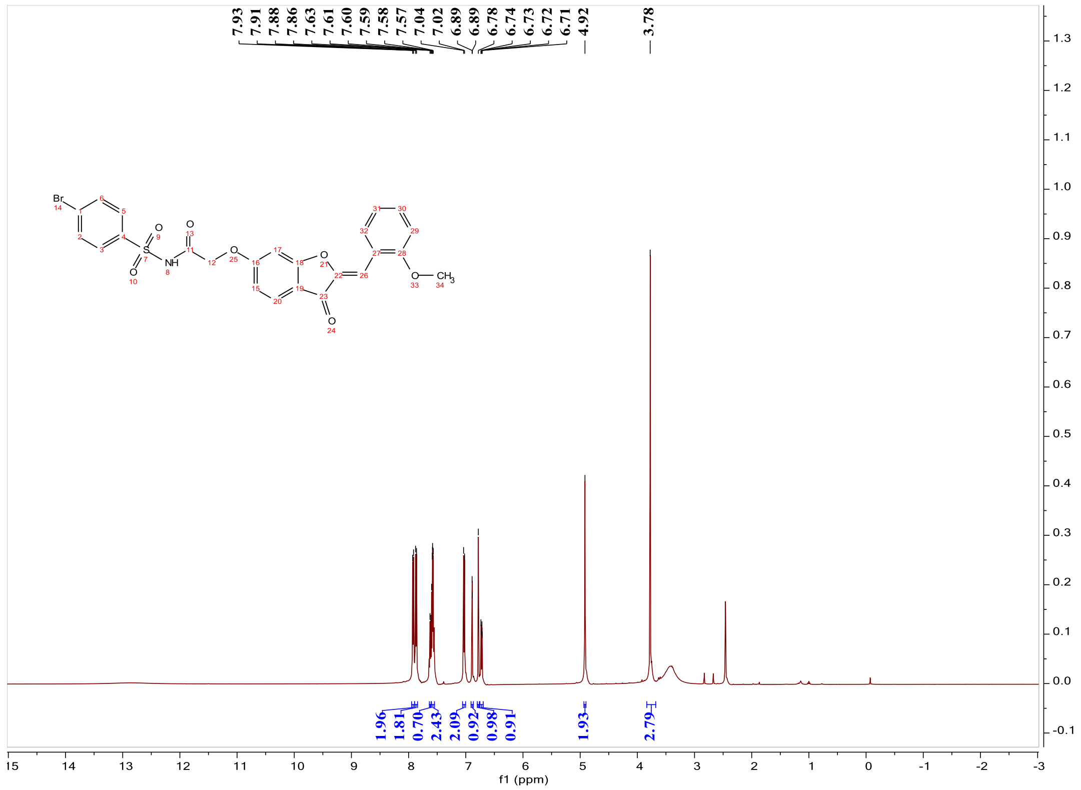

^1H NMR (500 MHz, DMSO-d_6) spectrum of compound **D15**

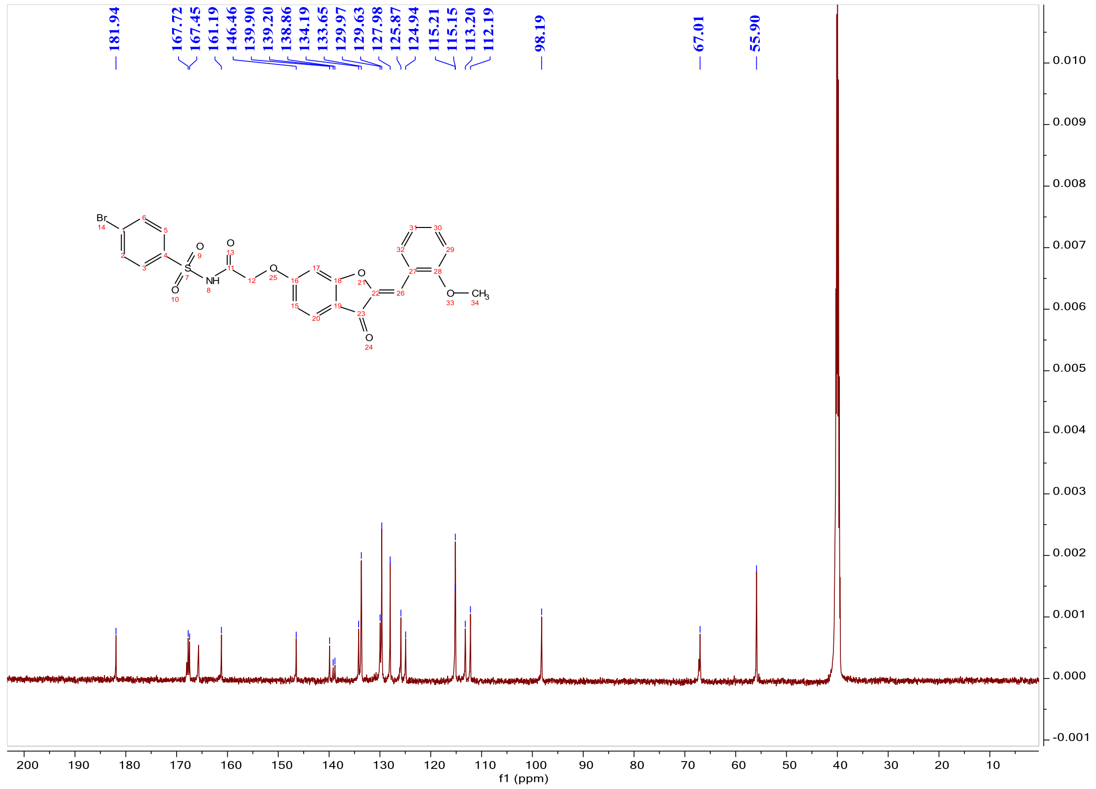

^{13}C NMR (126 MHz, DMSO-d_6) spectrum of compound **D15**

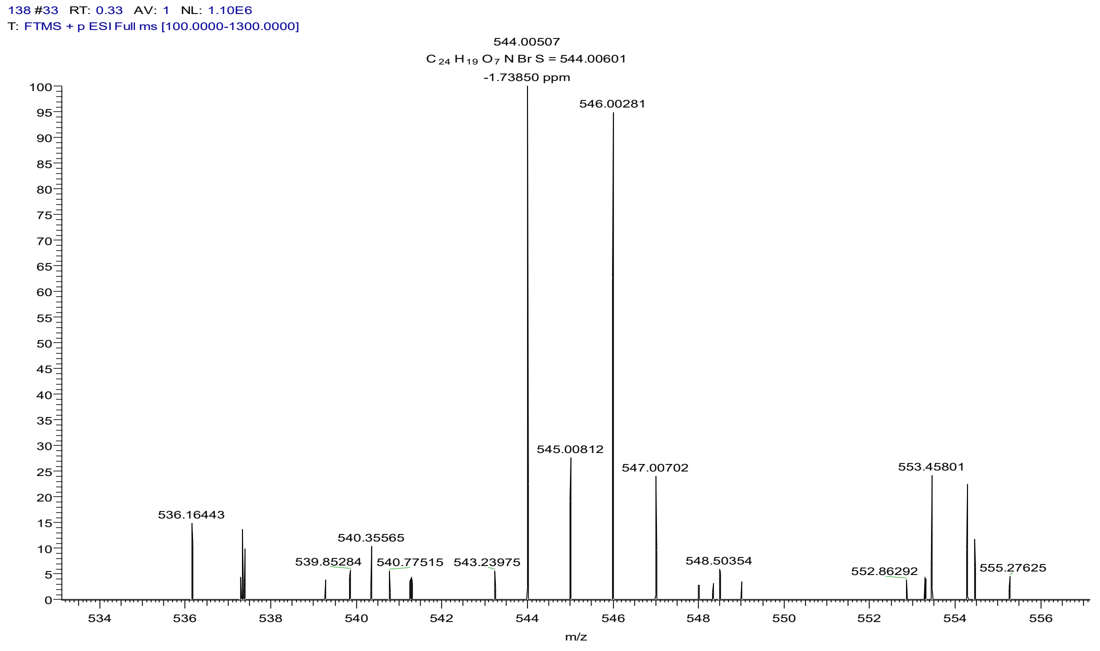

HRMS spectrum of compound **D15**

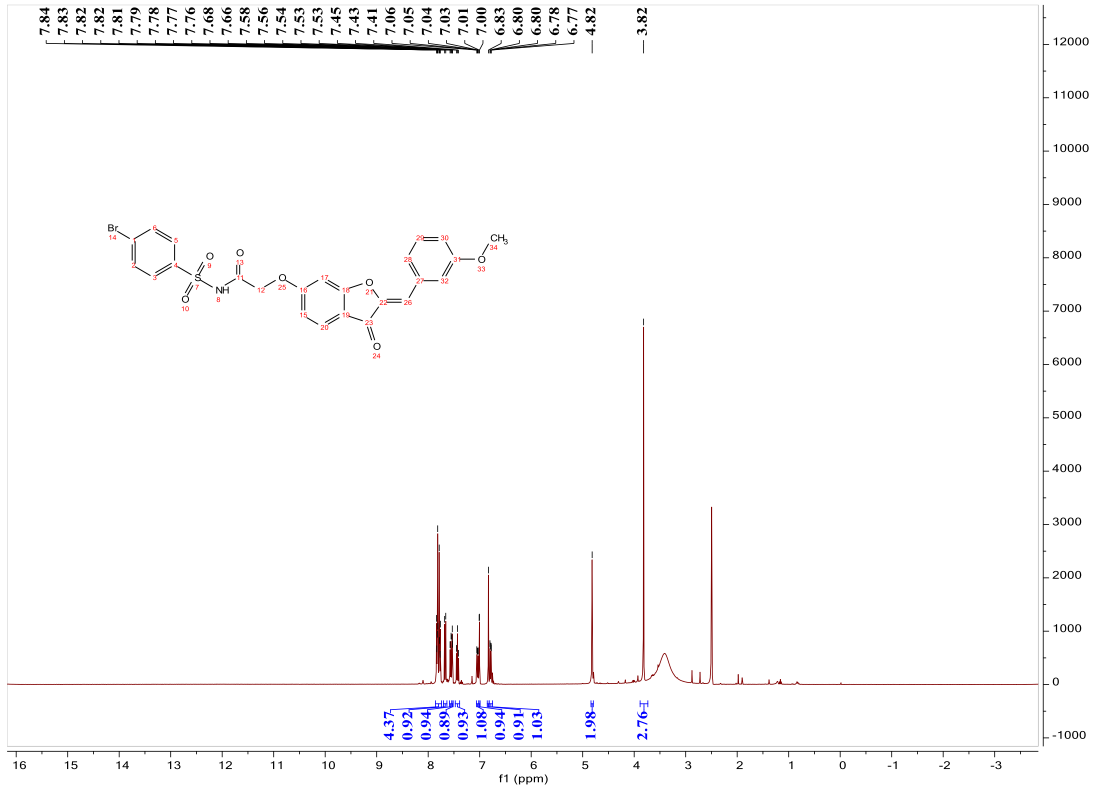

^{1}H NMR (400 MHz, DMSO-d_6) spectrum of compound **D16**

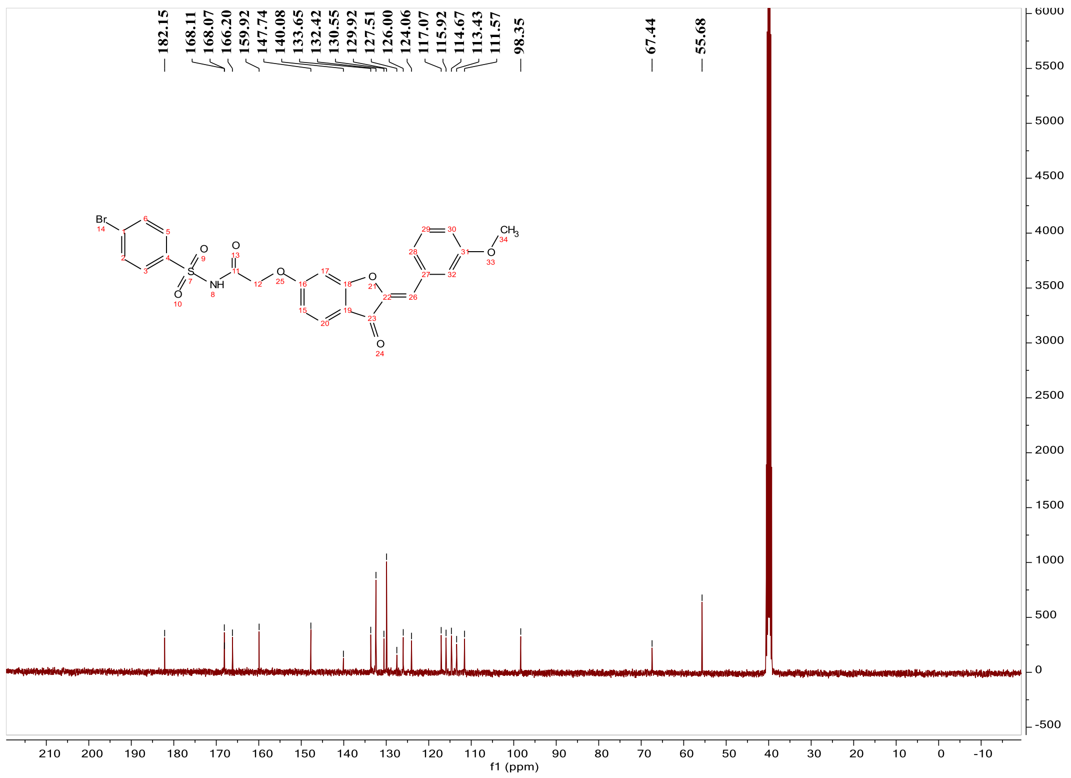

^{13}C NMR (101 MHz, DMSO-d_6) spectrum of compound **D16**

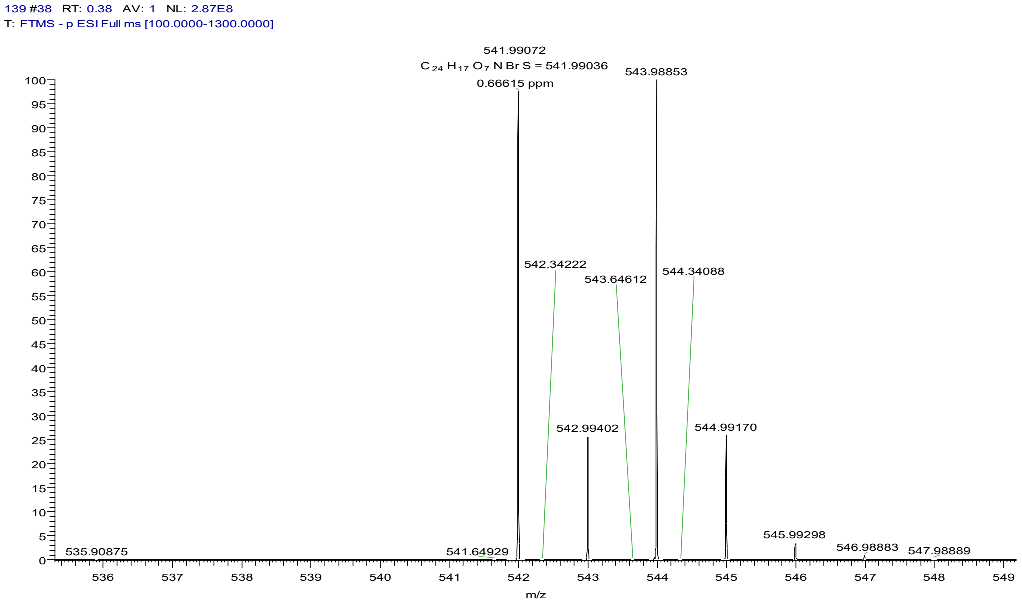

HRMS spectrum of compound **D16**

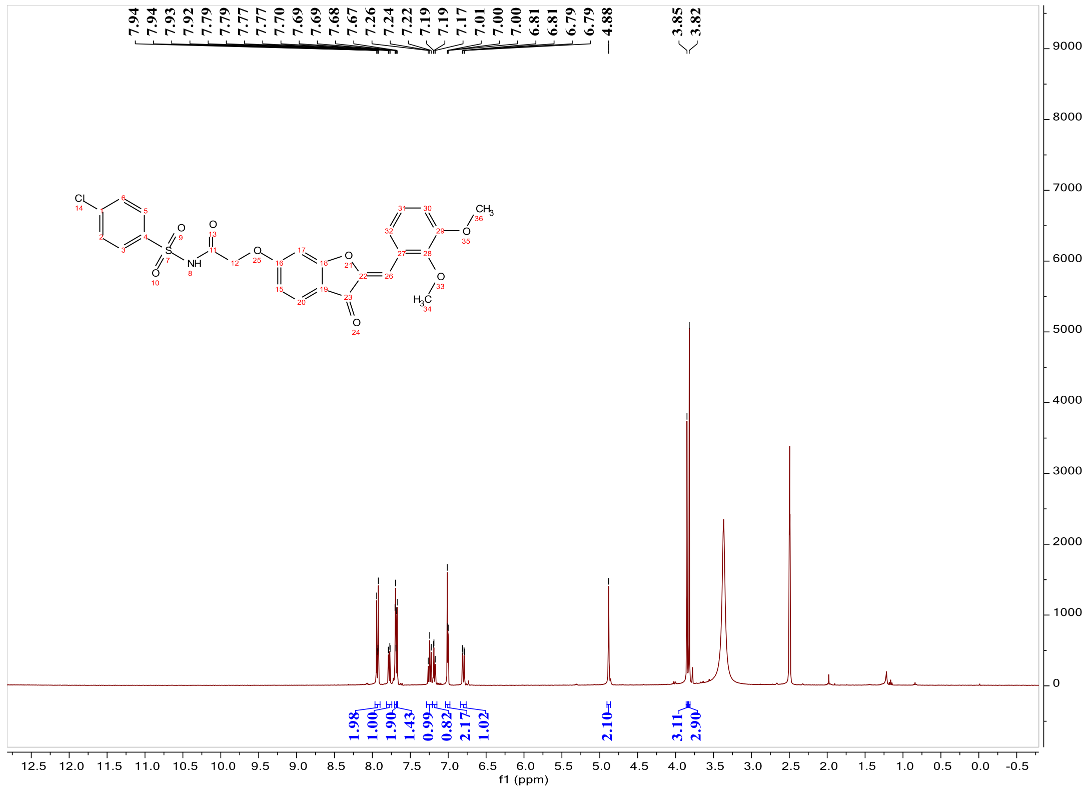

^{1}H NMR (400 MHz, DMSO-d_6) spectrum of compound **D17**

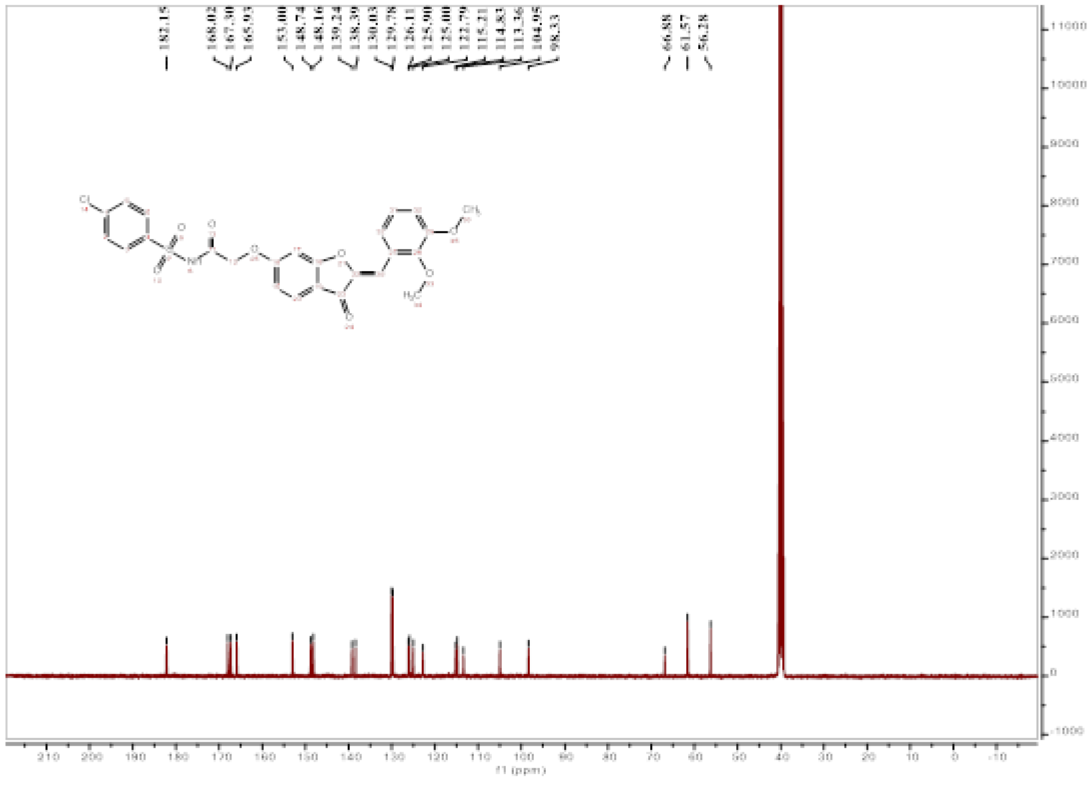

^{13}C NMR (101 MHz, DMSO-d_6) spectrum of compound **D17**

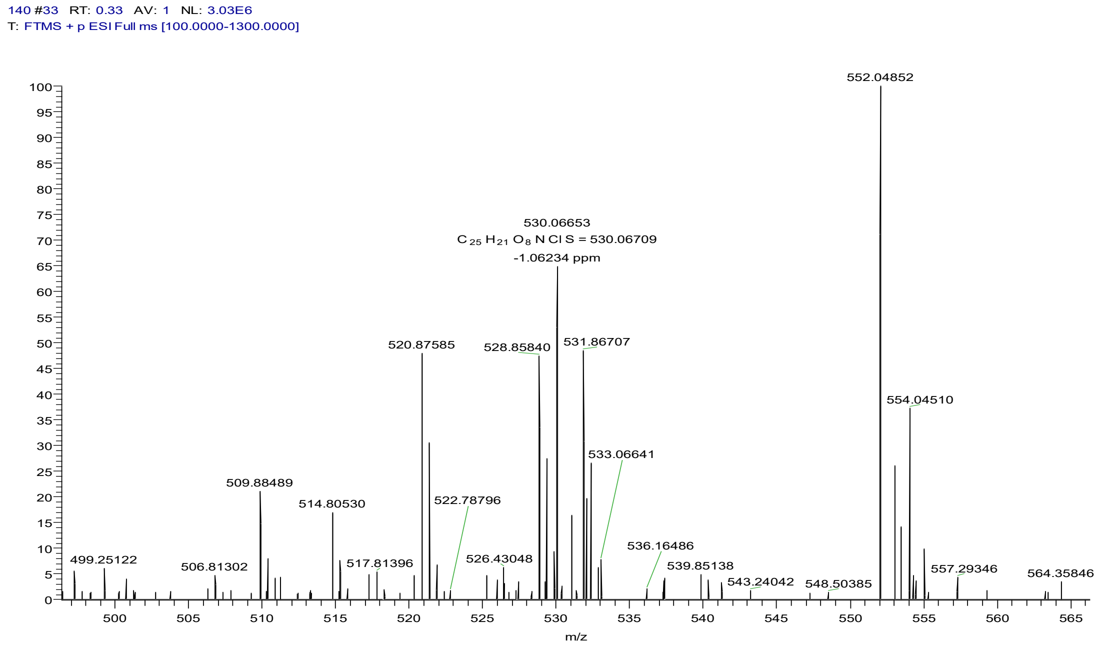

HRMS spectrum of compound **D17**

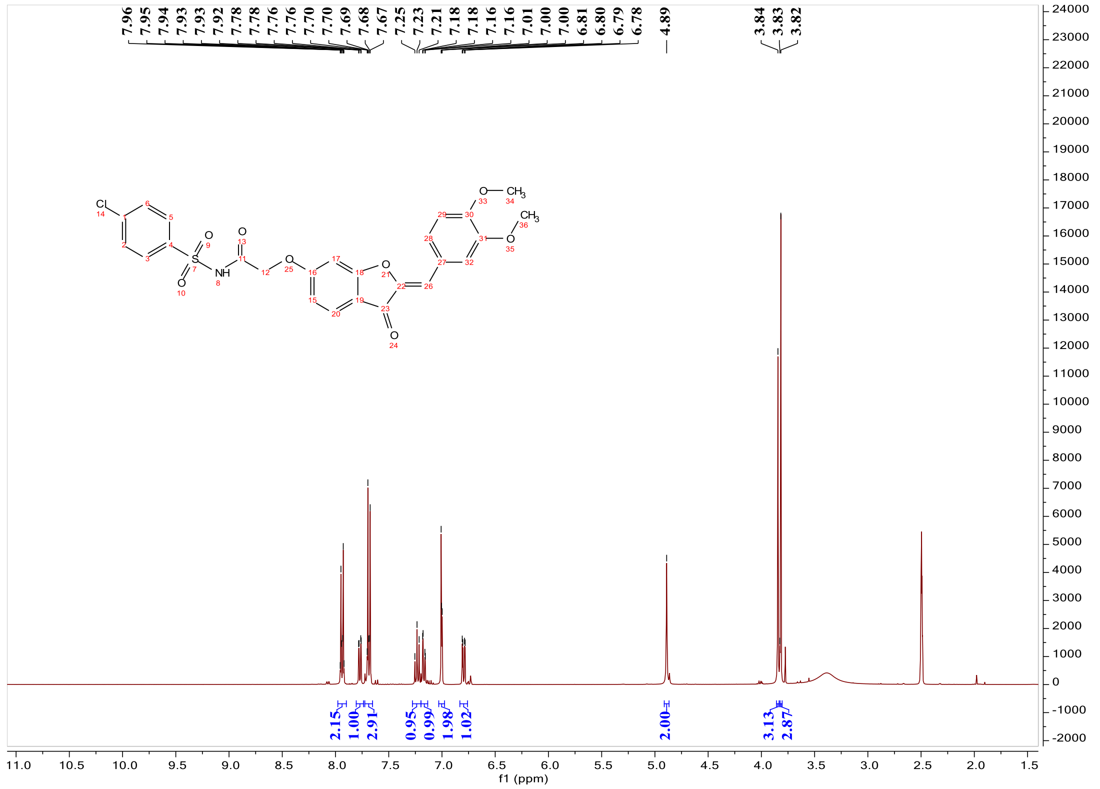

^{1}H NMR (400 MHz, DMSO-d_6) spectrum of compound **D18**

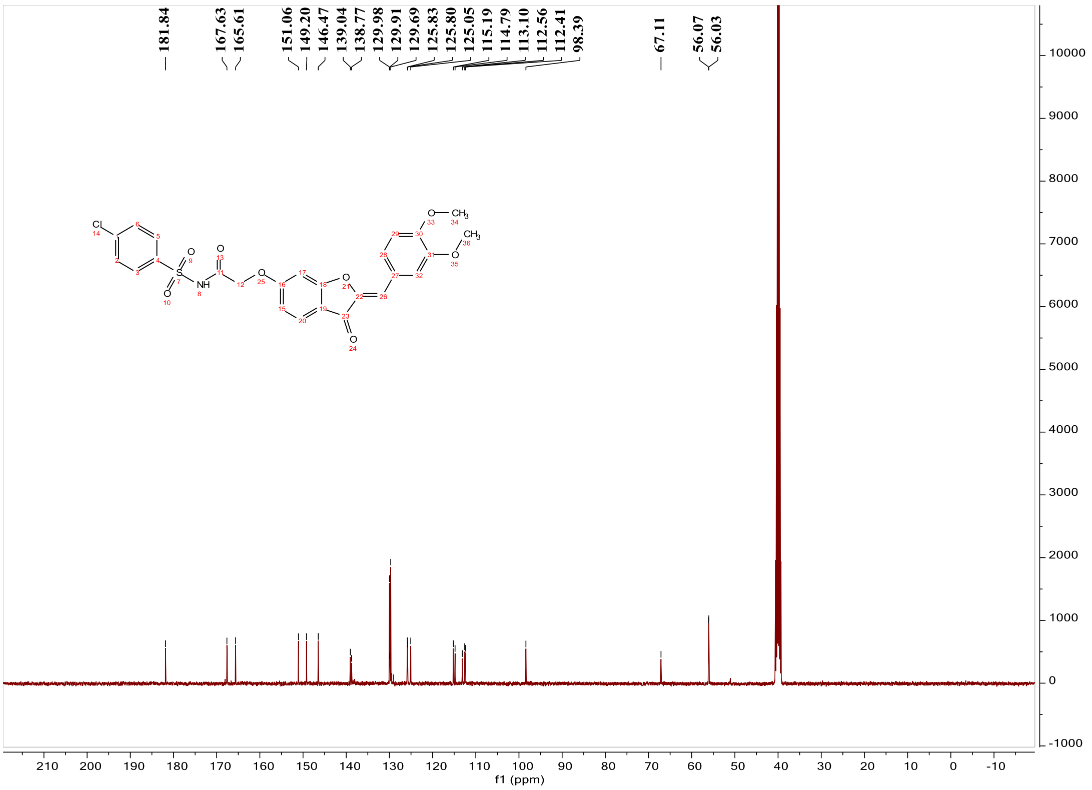

^{13}C NMR (101 MHz, DMSO-d_6) spectrum of compound **D18**

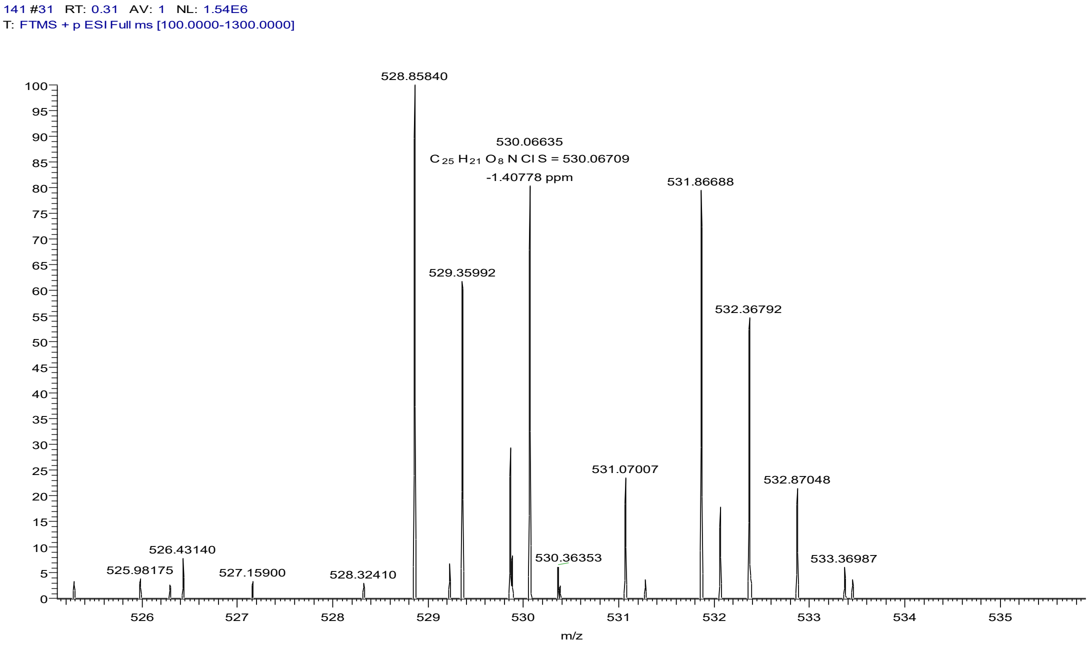

HRMS spectrum of compound **D18**

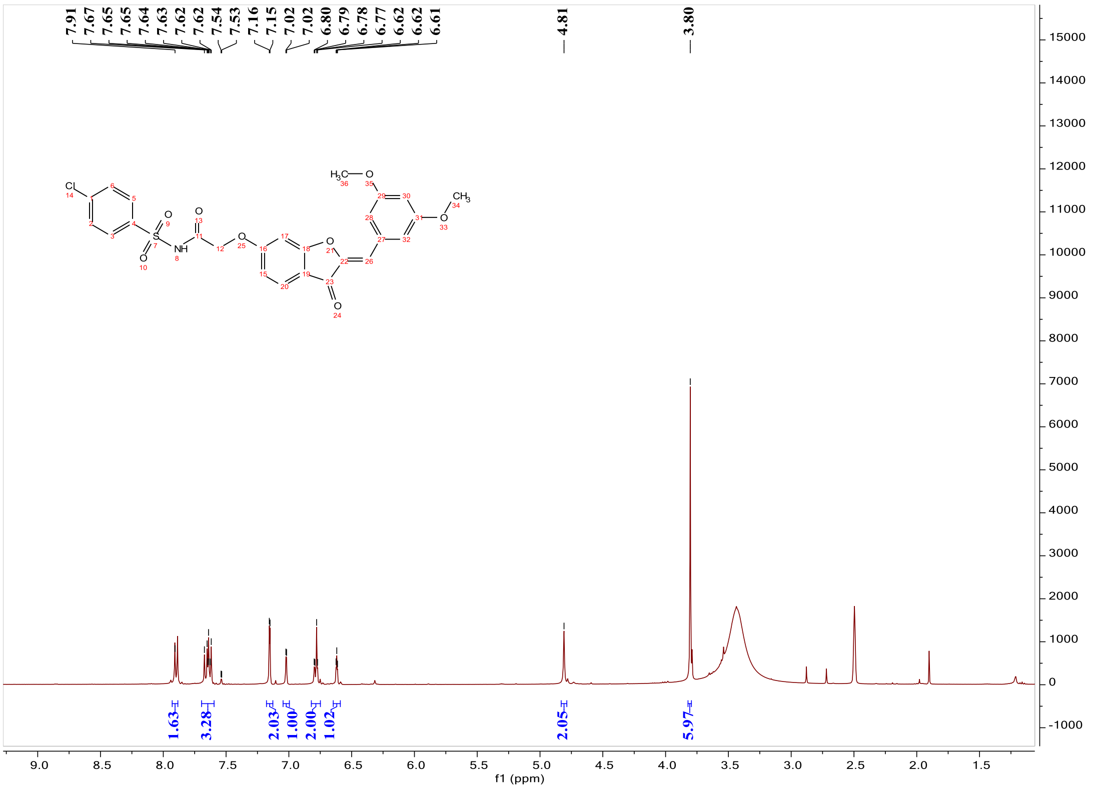

^{1}H NMR (400 MHz, DMSO-d_6) spectrum of compound **D19**

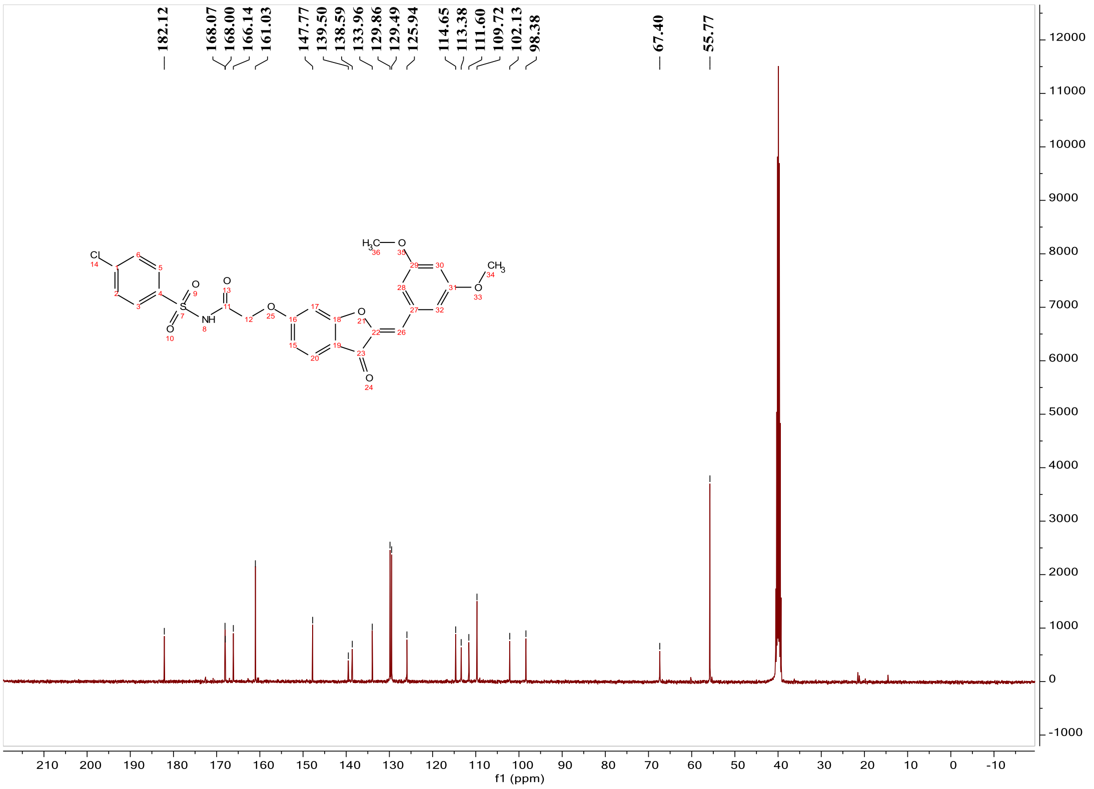

^{13}C NMR (101 MHz, DMSO-d_6) spectrum of compound **D19**

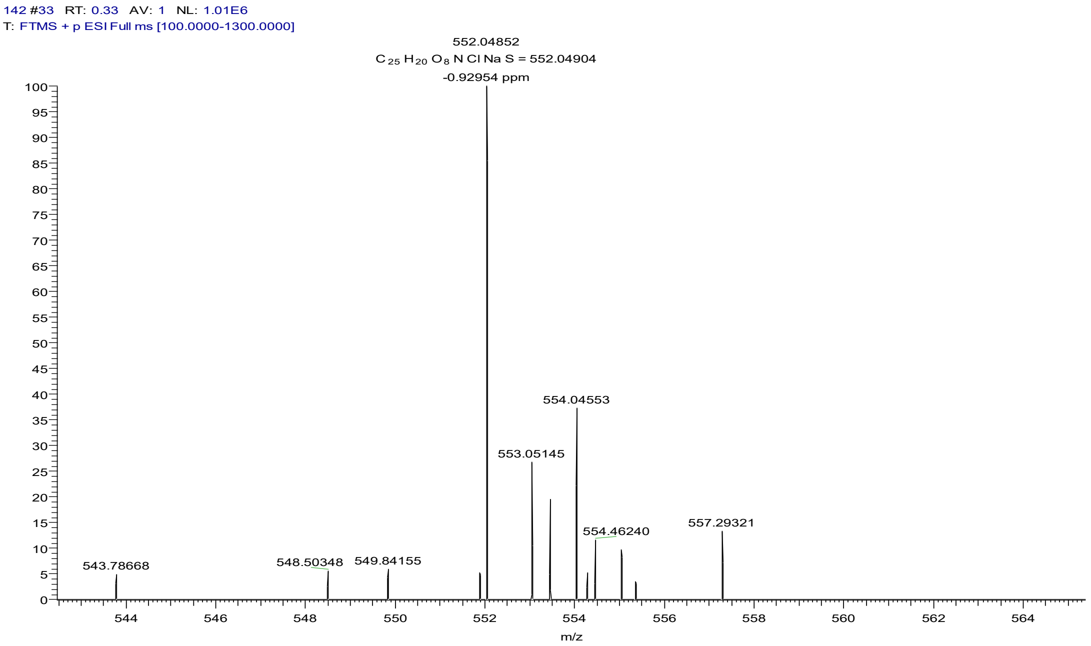

HRMS spectrum of compound **D19**

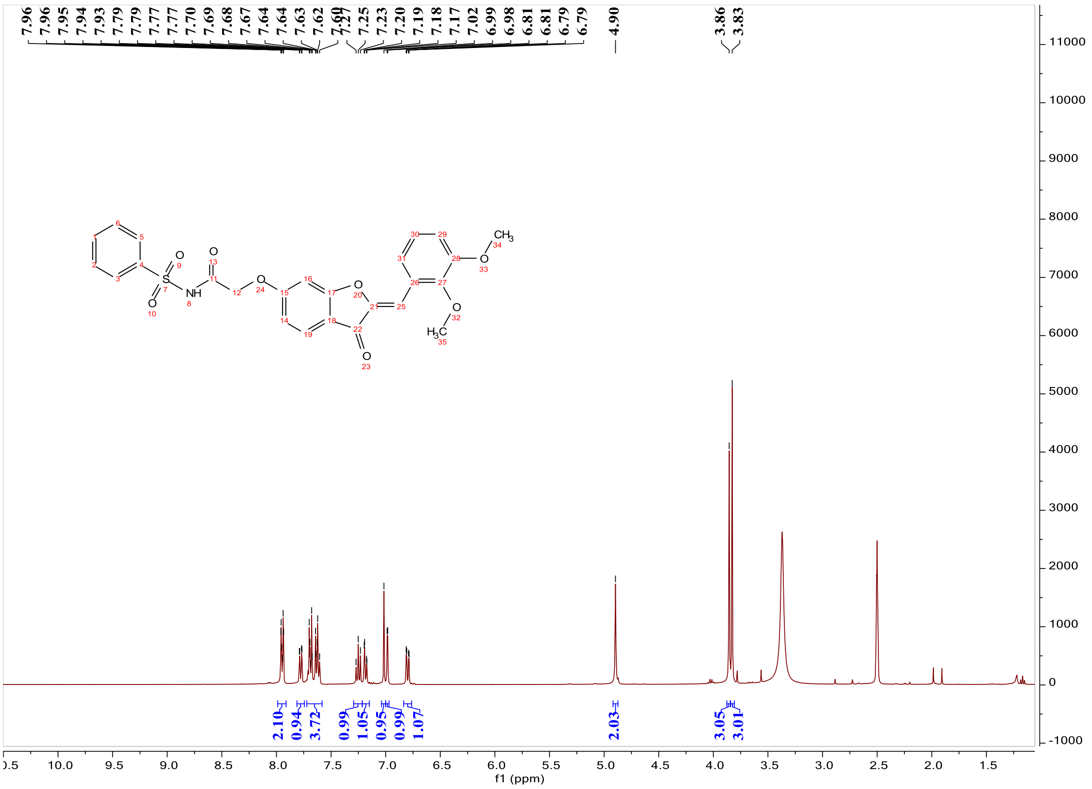

^{1}H NMR (400 MHz, DMSO-d_6) spectrum of compound **D20**

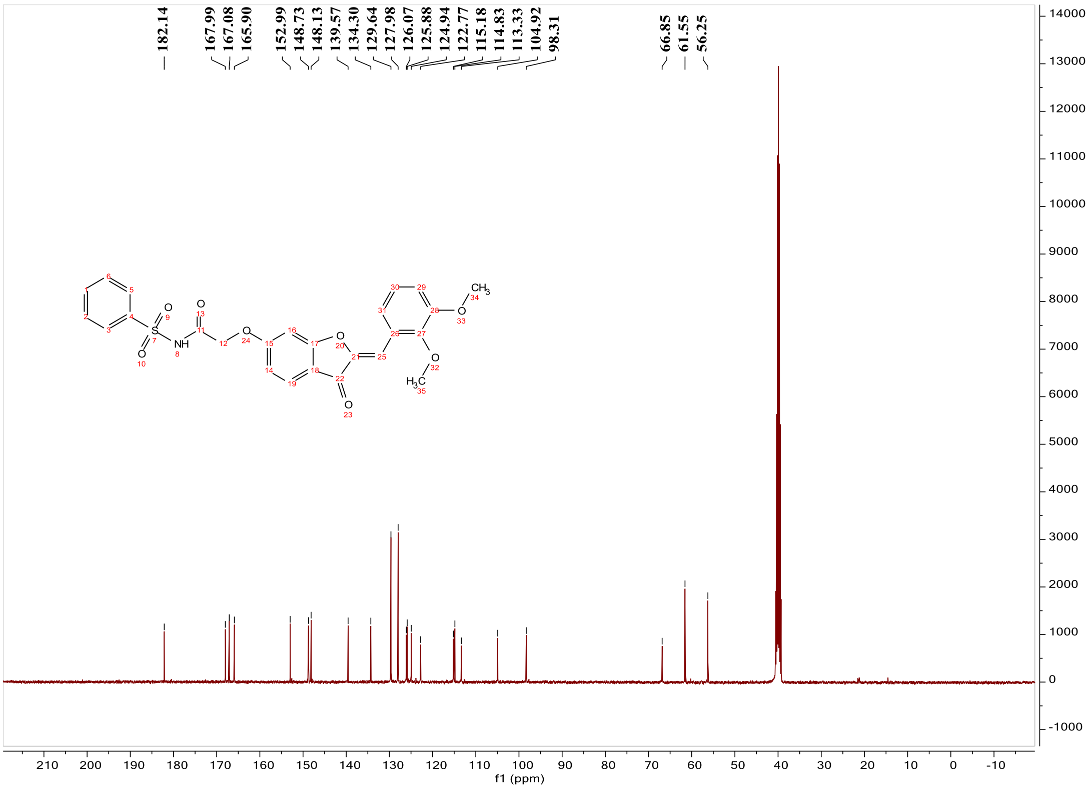

^{13}C NMR (101 MHz, DMSO-d_6) spectrum of compound **D20**

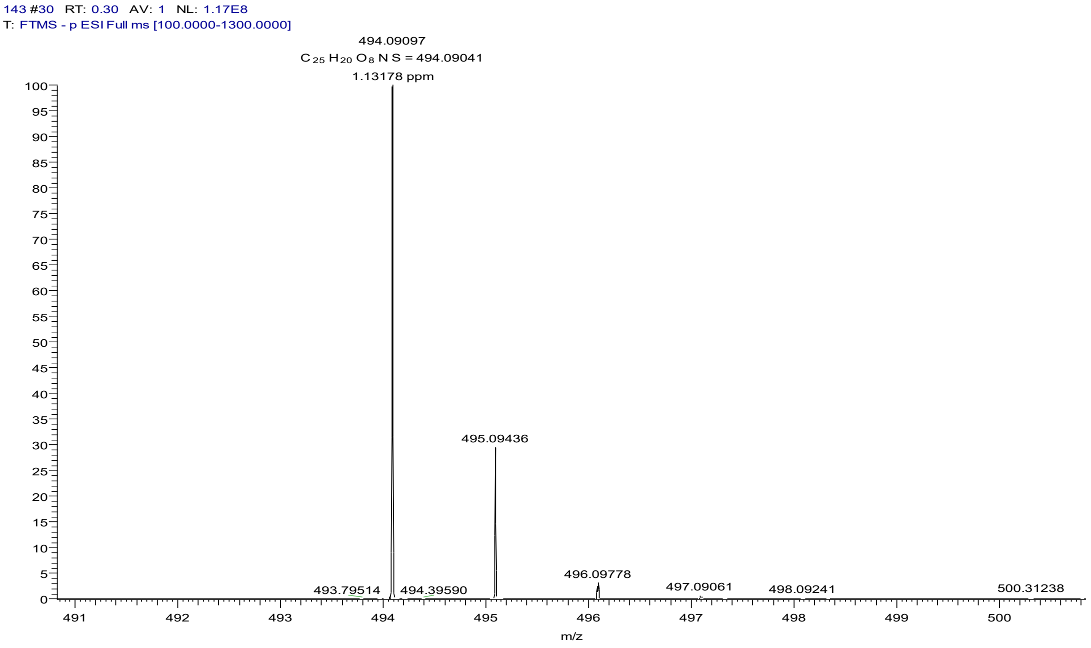

HRMS spectrum of compound **D20**

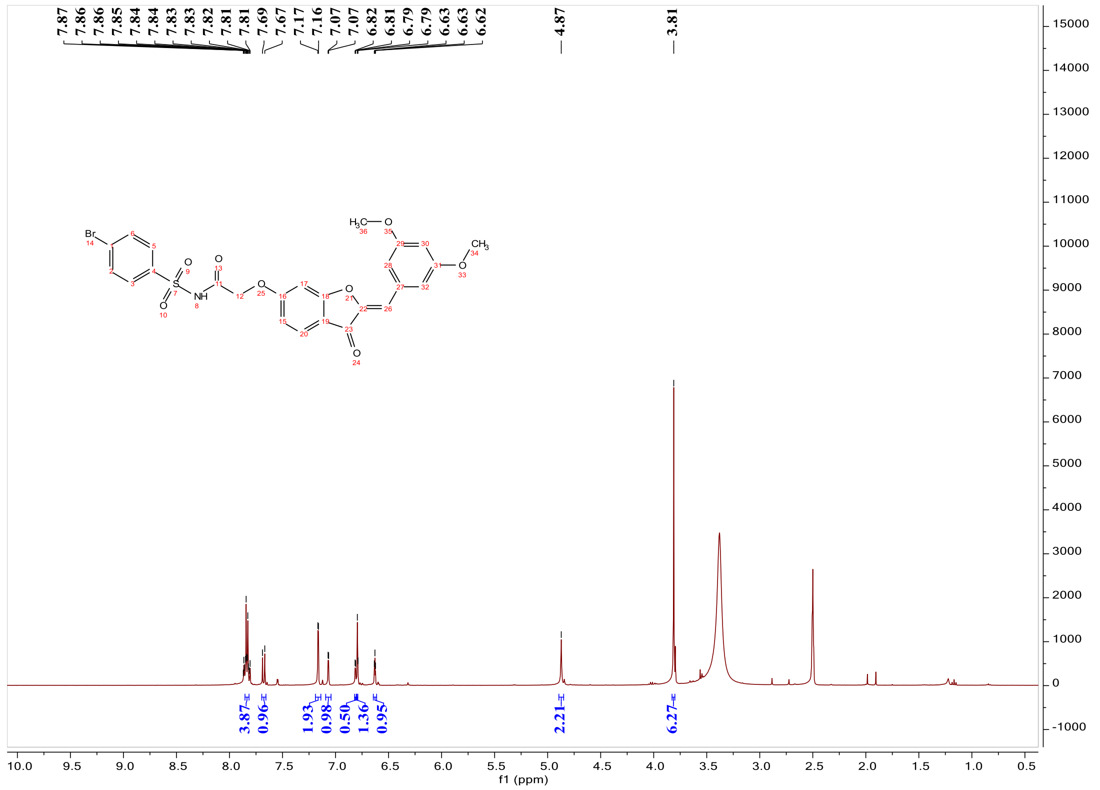

^{1}H NMR (400 MHz, DMSO-d_6) spectrum of compound **D21**

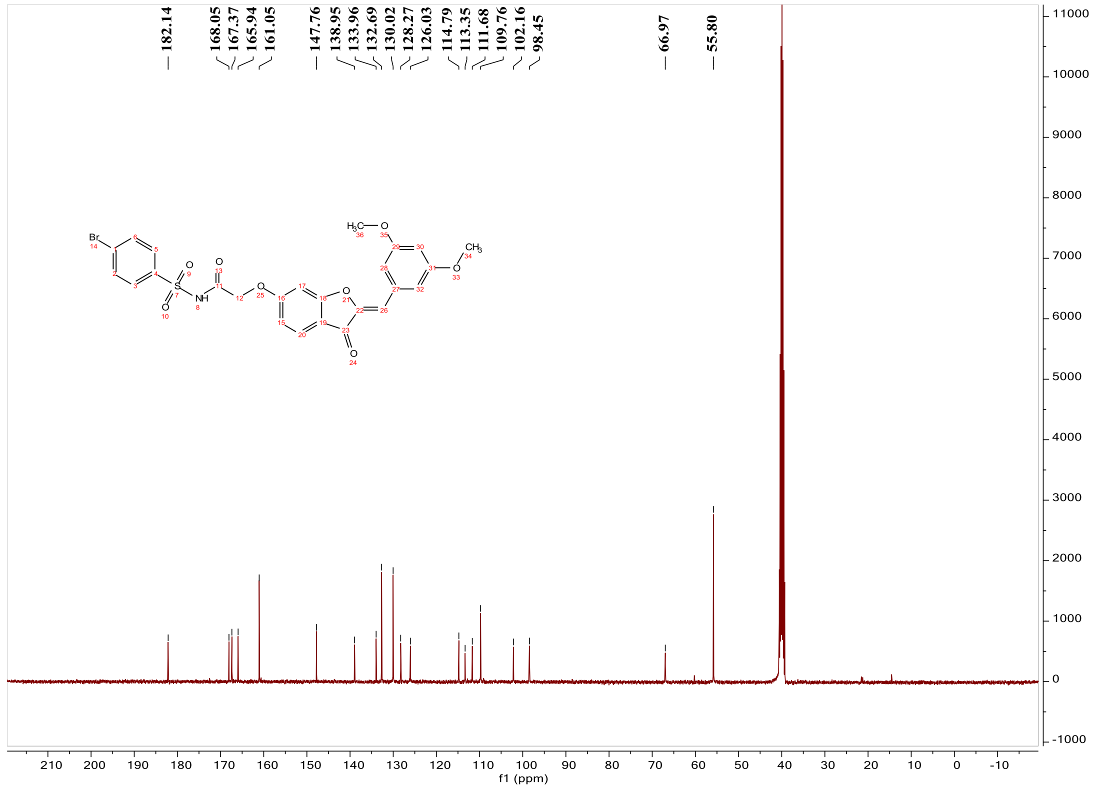

^{13}C NMR (101 MHz, DMSO-d_6) spectrum of compound **D21**

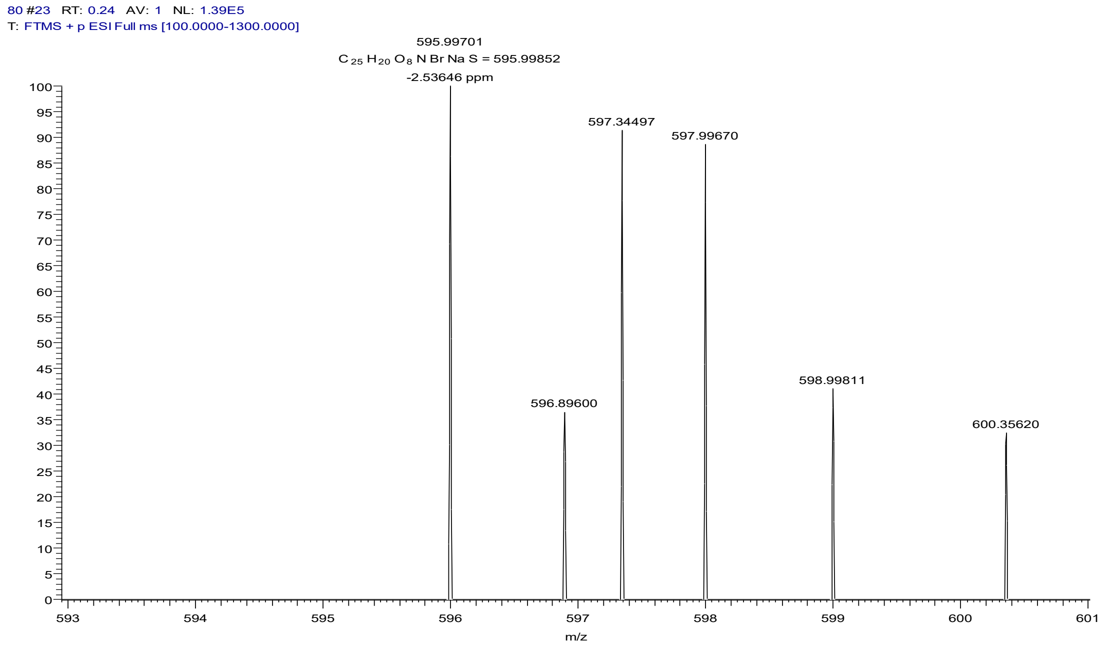

HRMS spectrum of compound **D21**

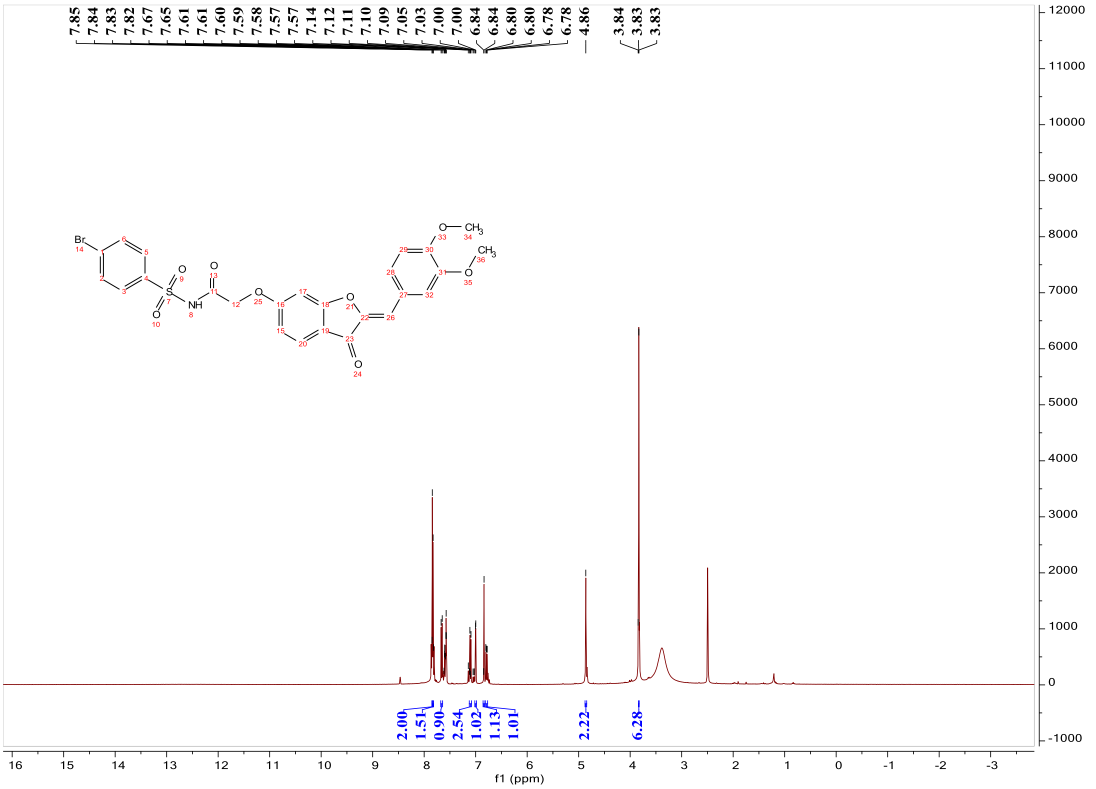

^{1}H NMR (400 MHz, DMSO-d_6) spectrum of compound **D22**

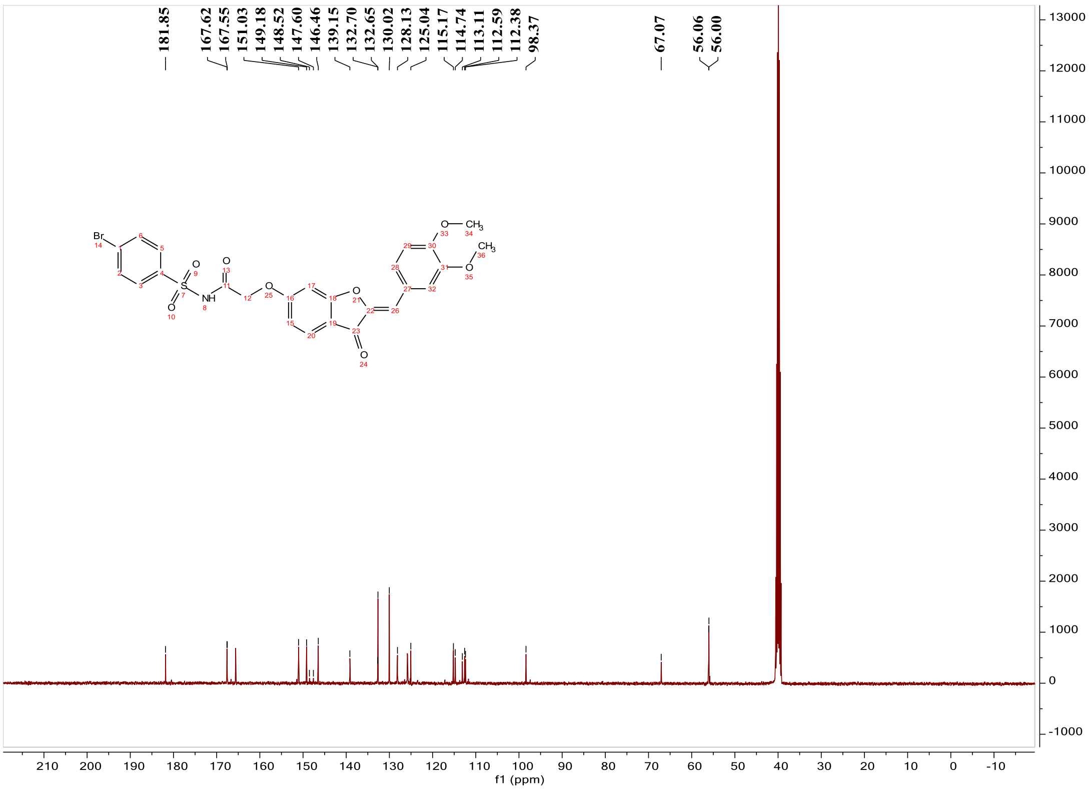

^{13}C NMR (101 MHz, DMSO-d_6) spectrum of compound **D22**

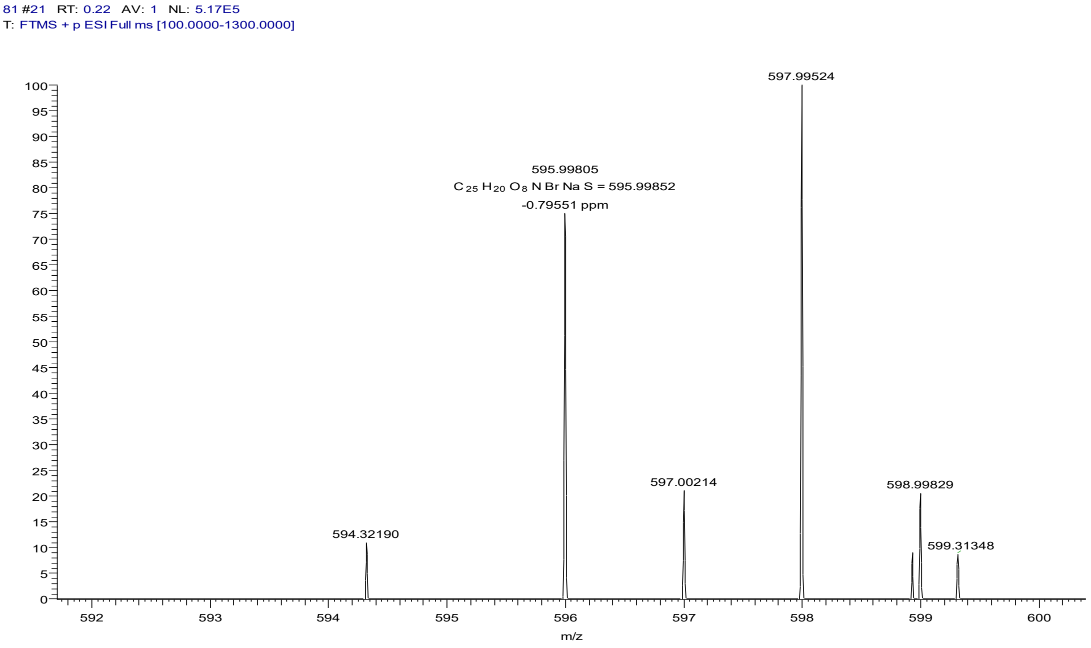

HRMS spectrum of compound **D22**

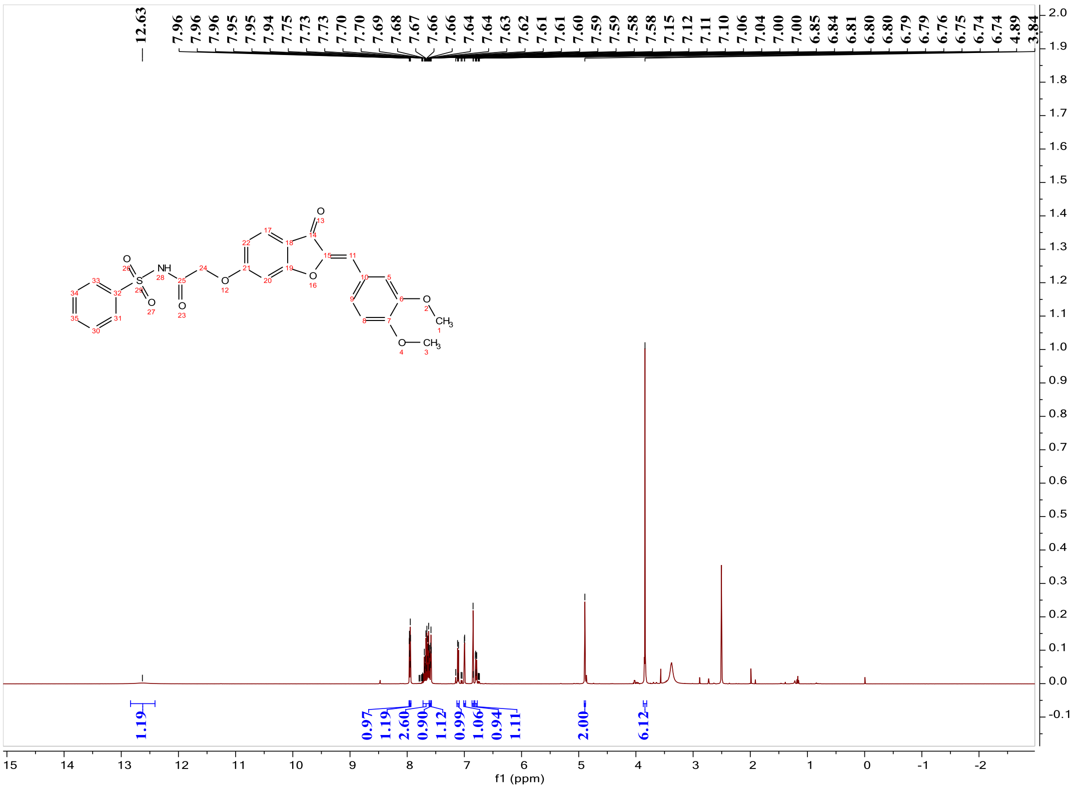

^{1}H NMR (500 MHz, DMSO-d_6) spectrum of compound **D23**

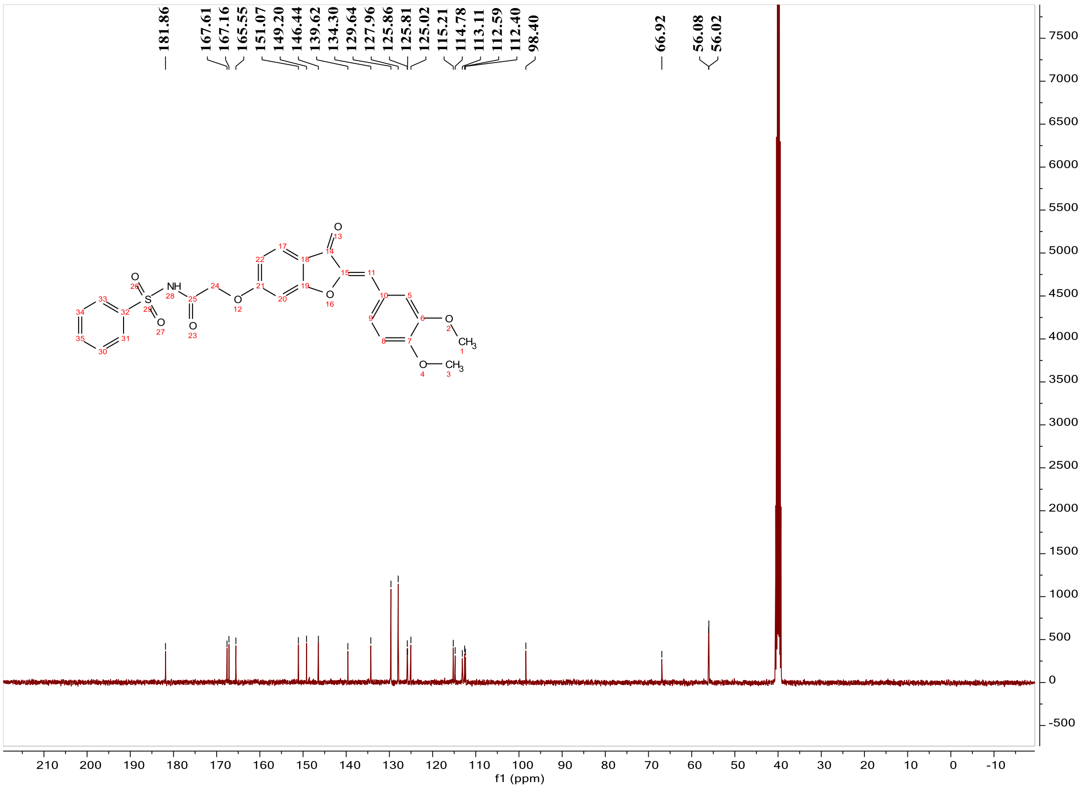

^{13}C NMR (101 MHz, DMSO-d_6) spectrum of compound **D23**

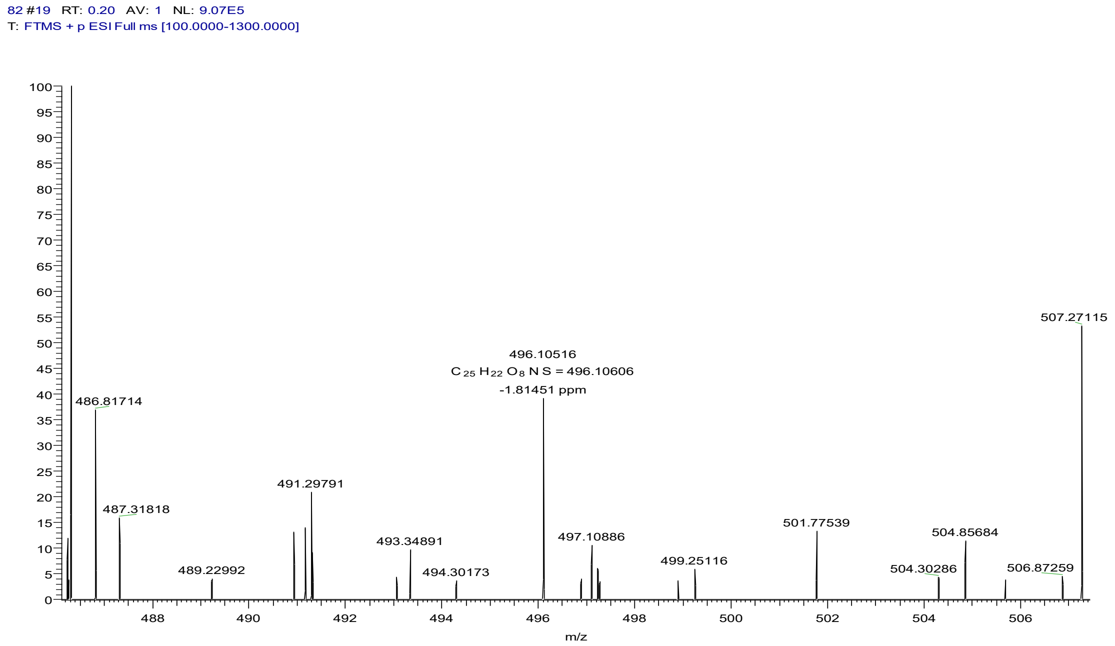

HRMS spectrum of compound **D23**

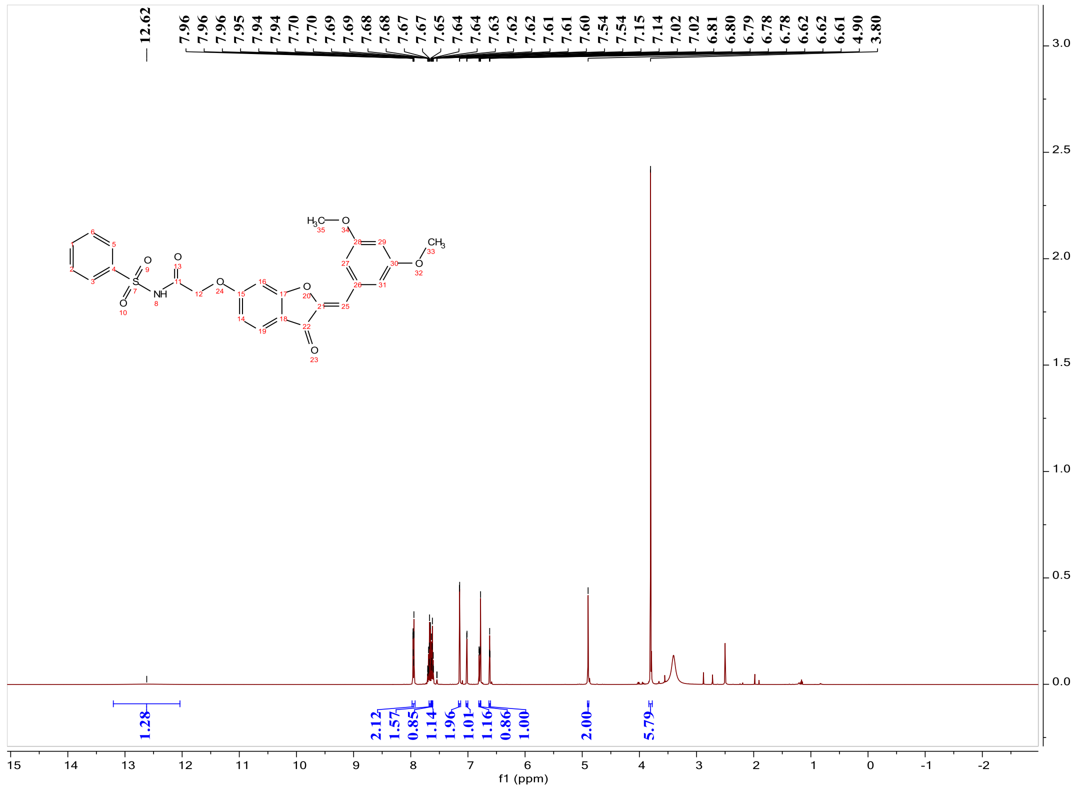

^{1}H NMR (500 MHz, DMSO-d_6) spectrum of compound **D24**

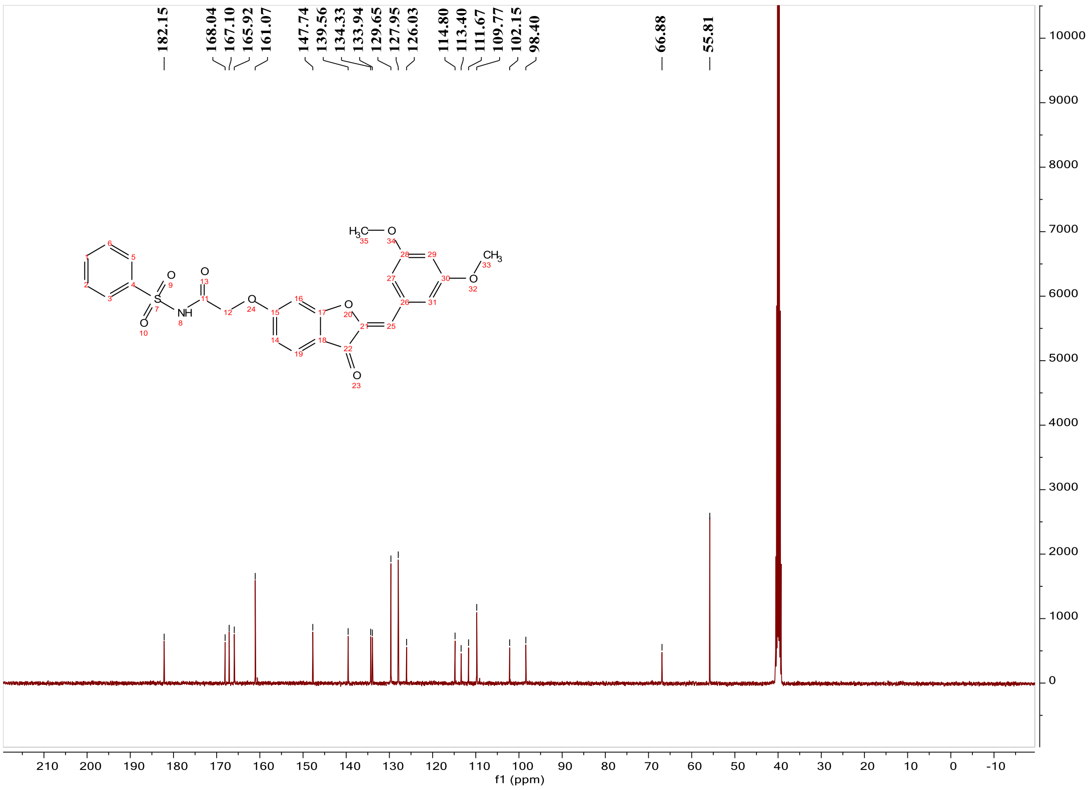

^{13}C NMR (101 MHz, DMSO-d_6) spectrum of compound **D24**

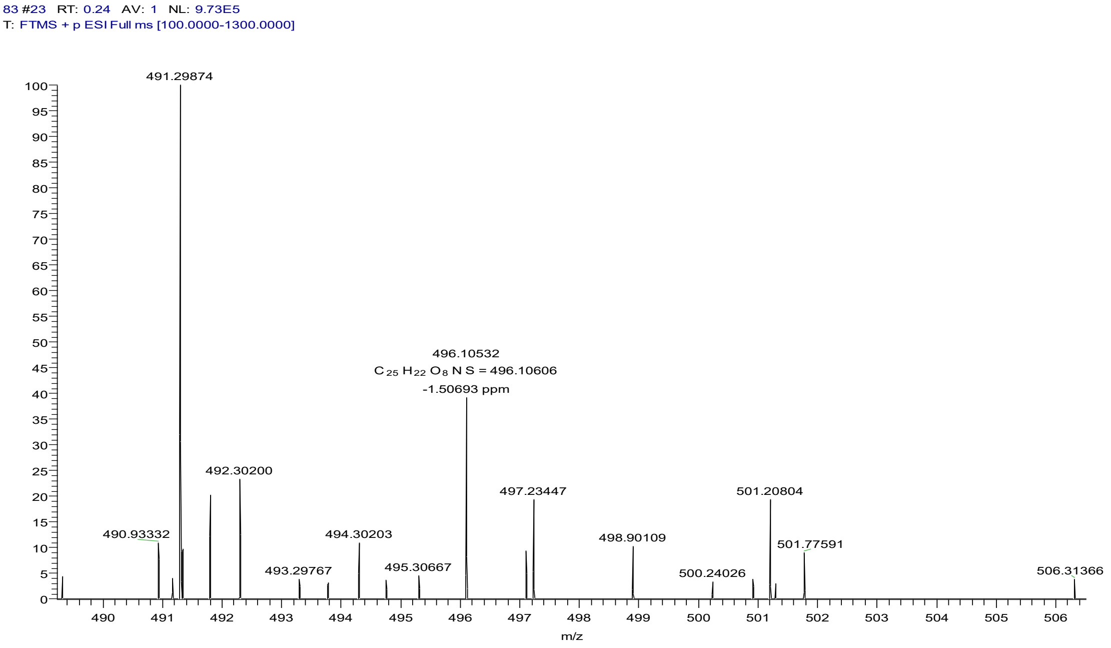

HRMS spectrum of compound **D24**

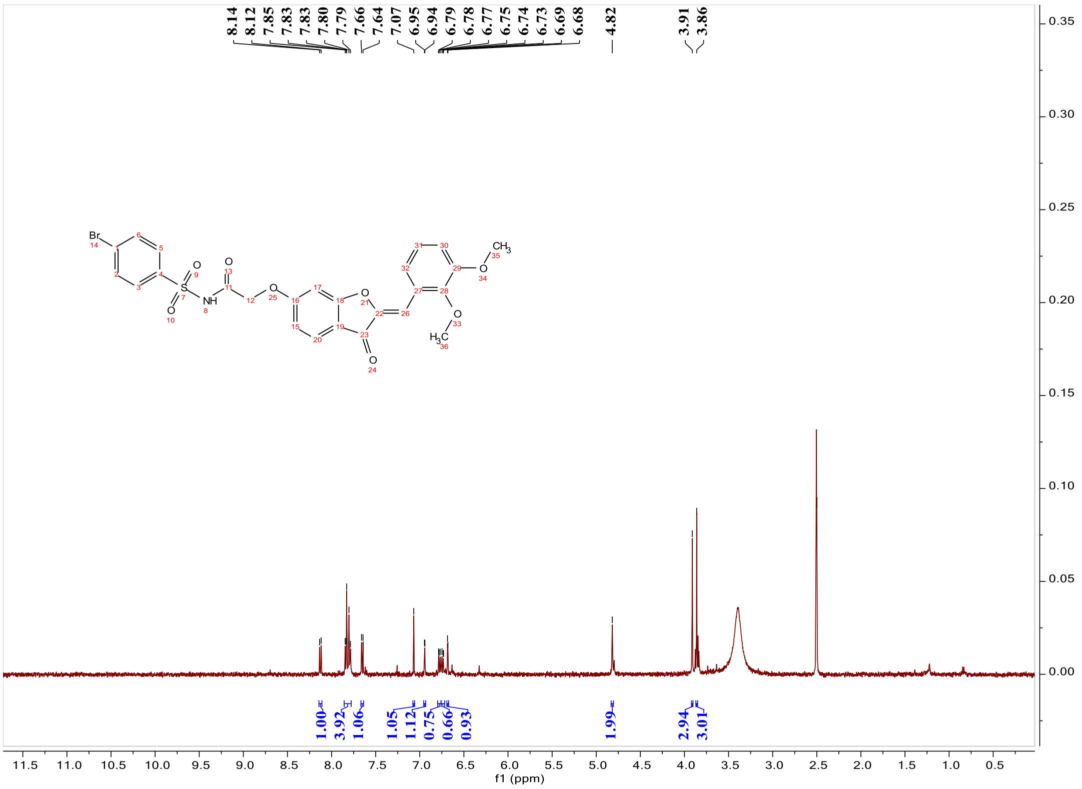

^{1}H NMR (500 MHz, DMSO-d_6) spectrum of compound **D25**

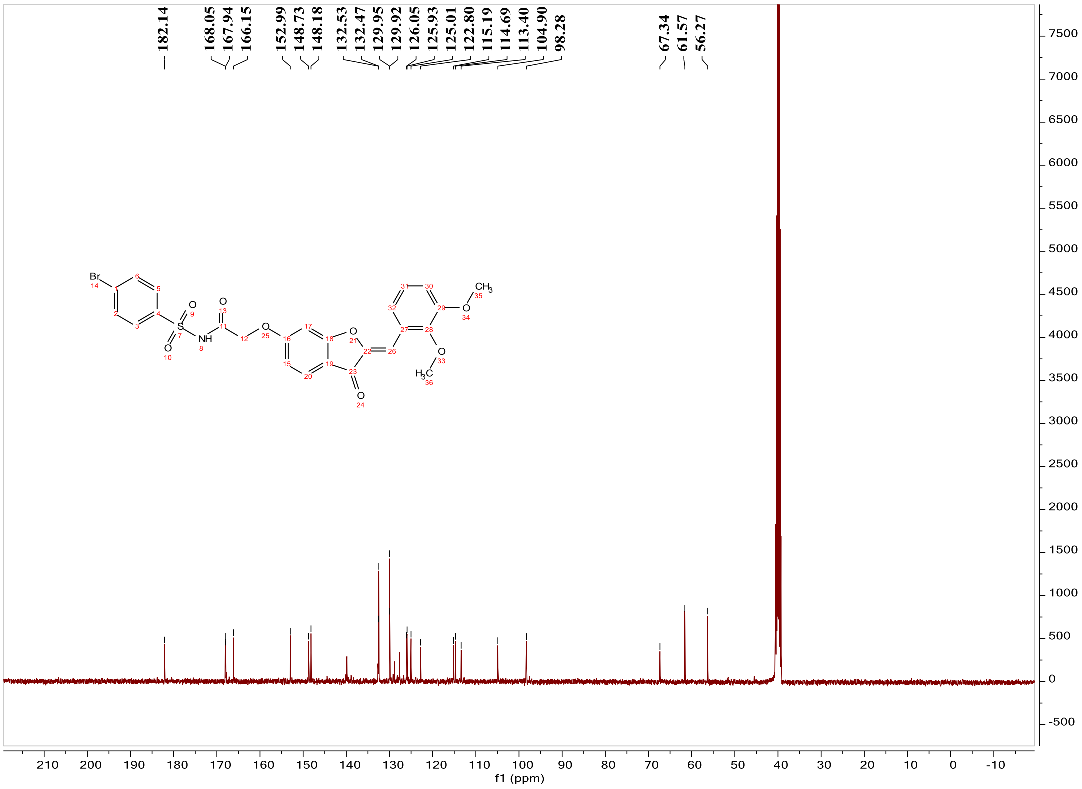

^{13}C NMR (101 MHz, DMSO-d_6) spectrum of compound **D25**

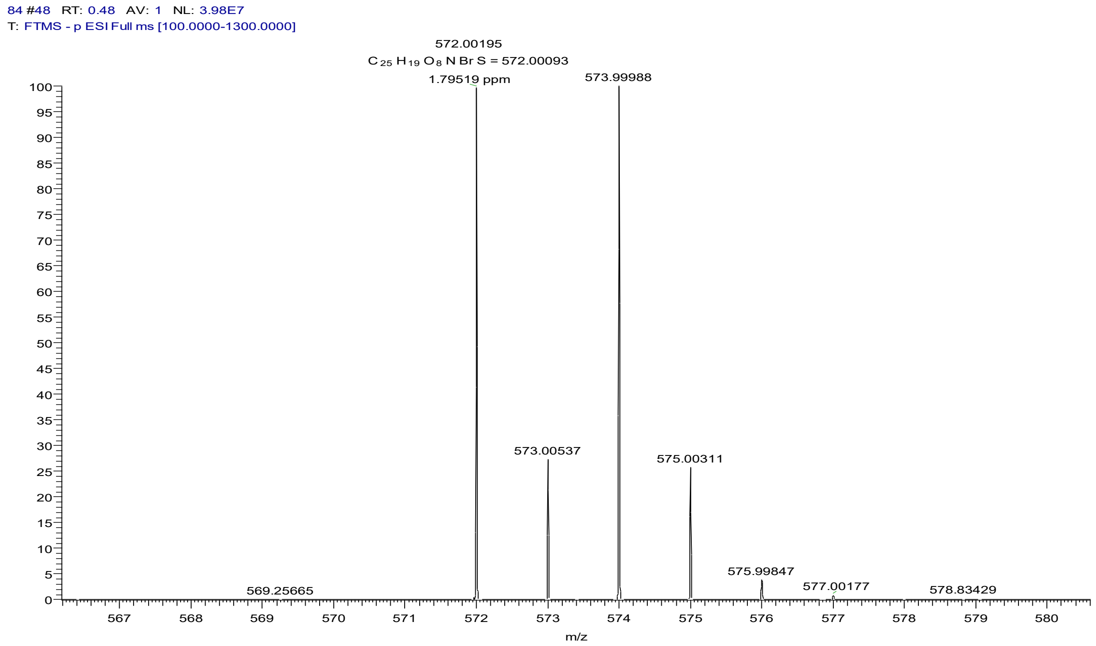

HRMS spectrum of compound **D25**

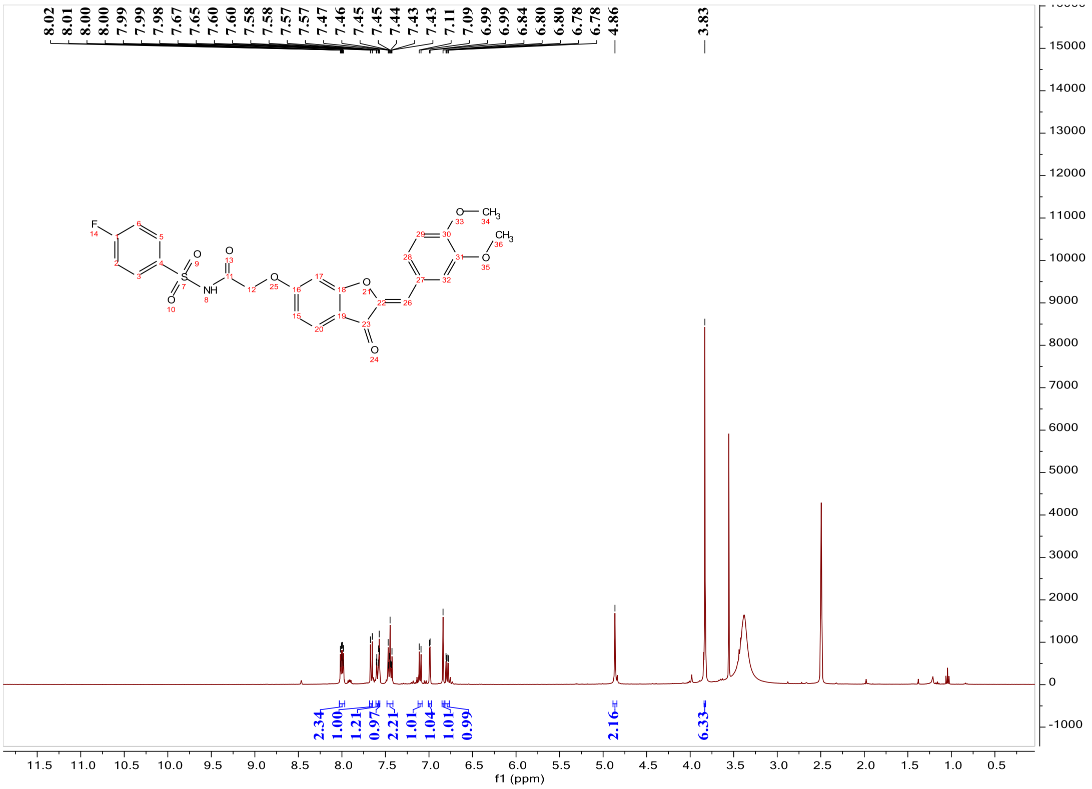

^{1}H NMR (400 MHz, DMSO-d_6) spectrum of compound **D26**

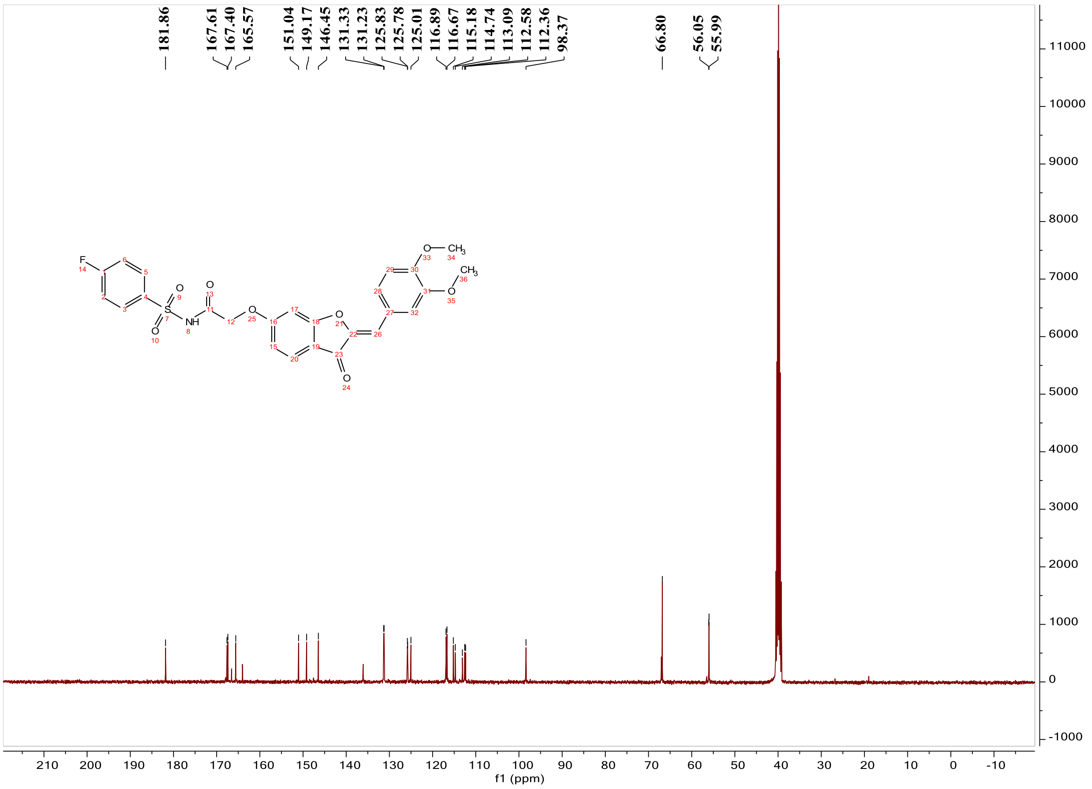

^{13}C NMR (101 MHz, DMSO-d_6) spectrum of compound **D26**

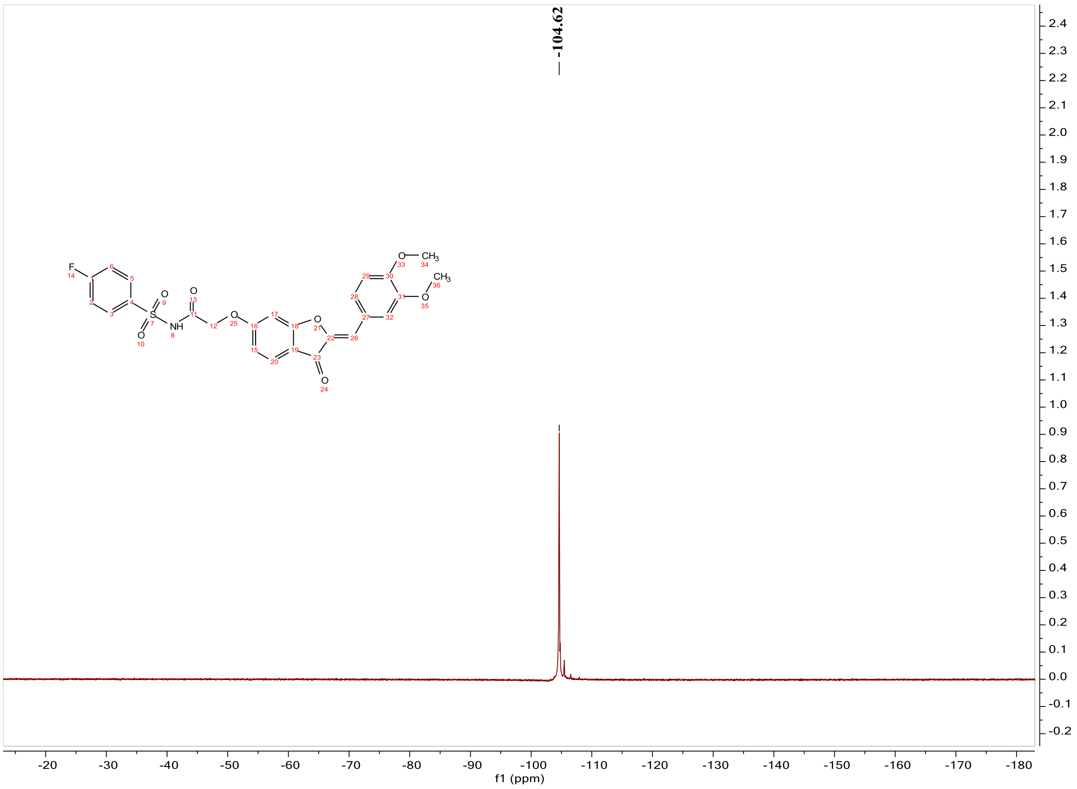

^{19}F NMR (471 MHz, DMSO-d_6) spectrum of compound **D26**

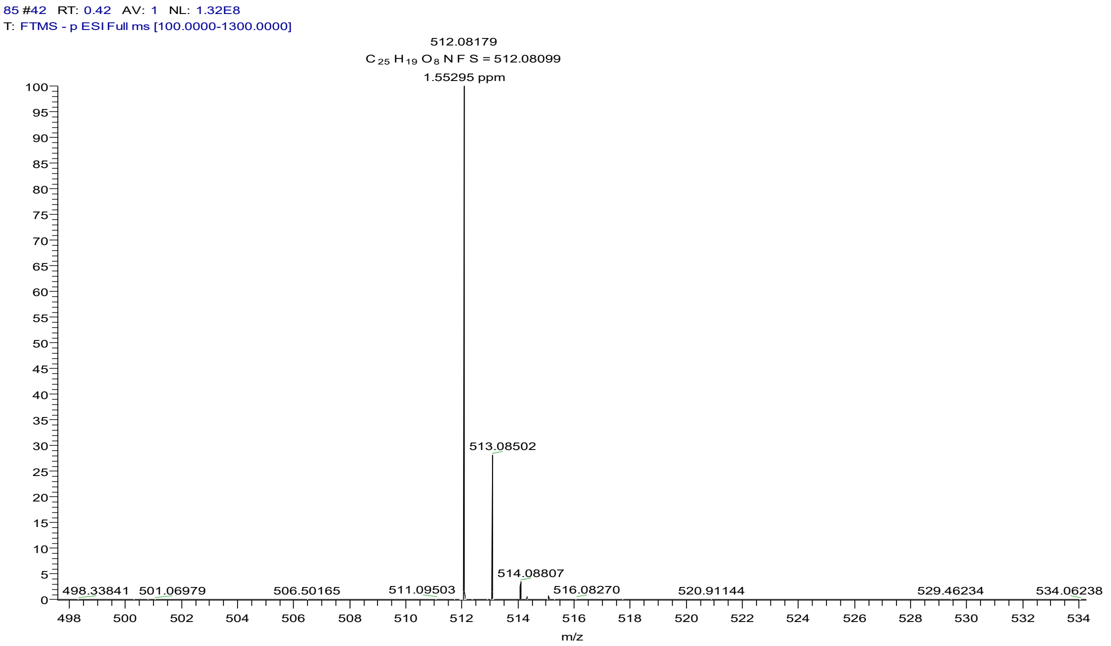

HRMS spectrum of compound **D26**

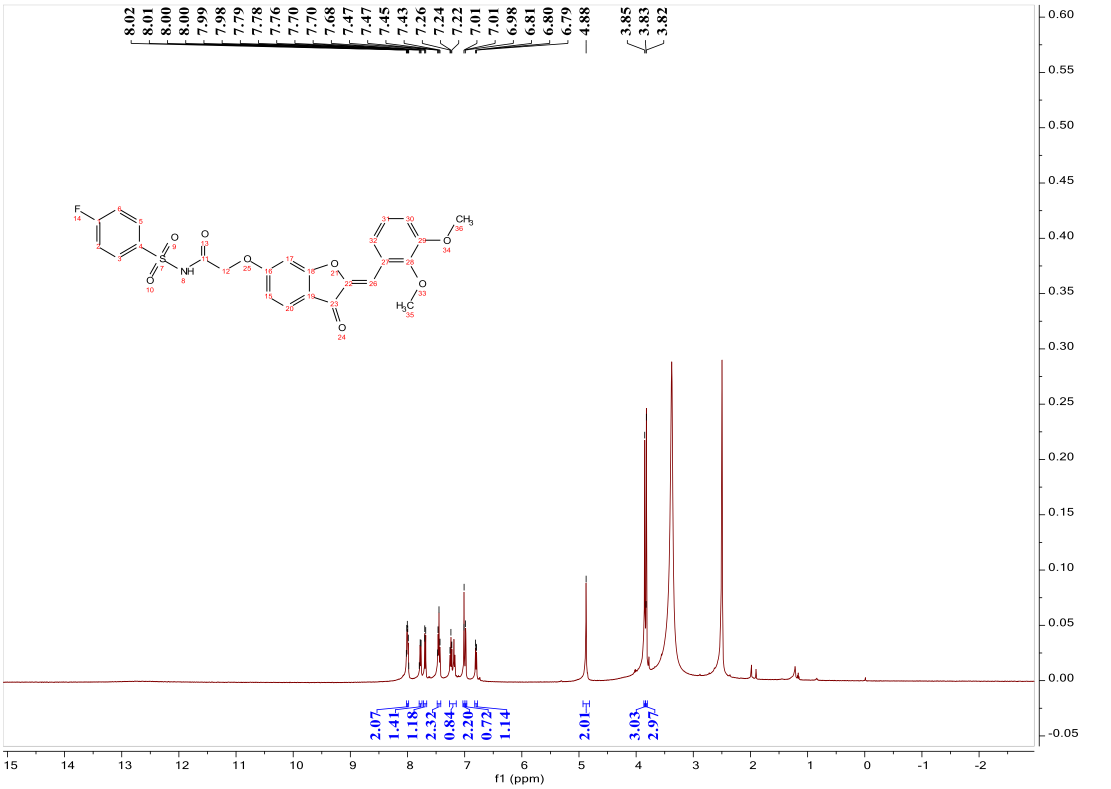

^{1}H NMR (500 MHz, DMSO-d_6) spectrum of compound **D27**

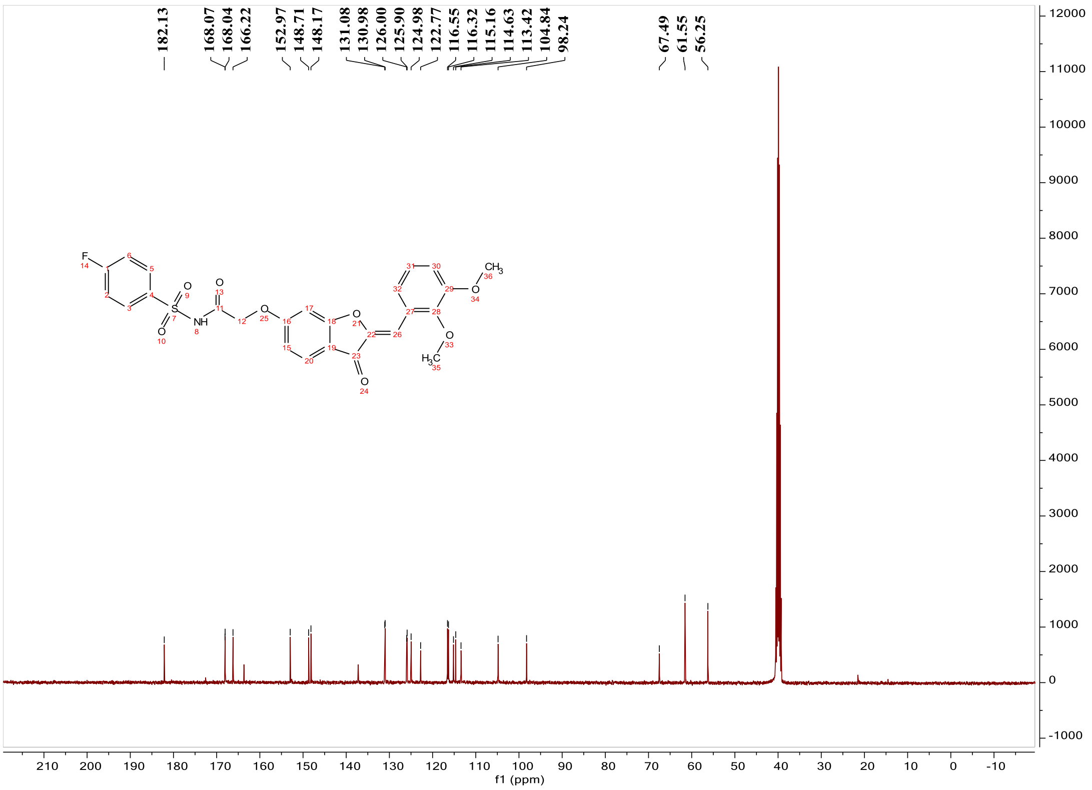

^{13}C NMR (101 MHz, DMSO-d_6) spectrum of compound **D27**

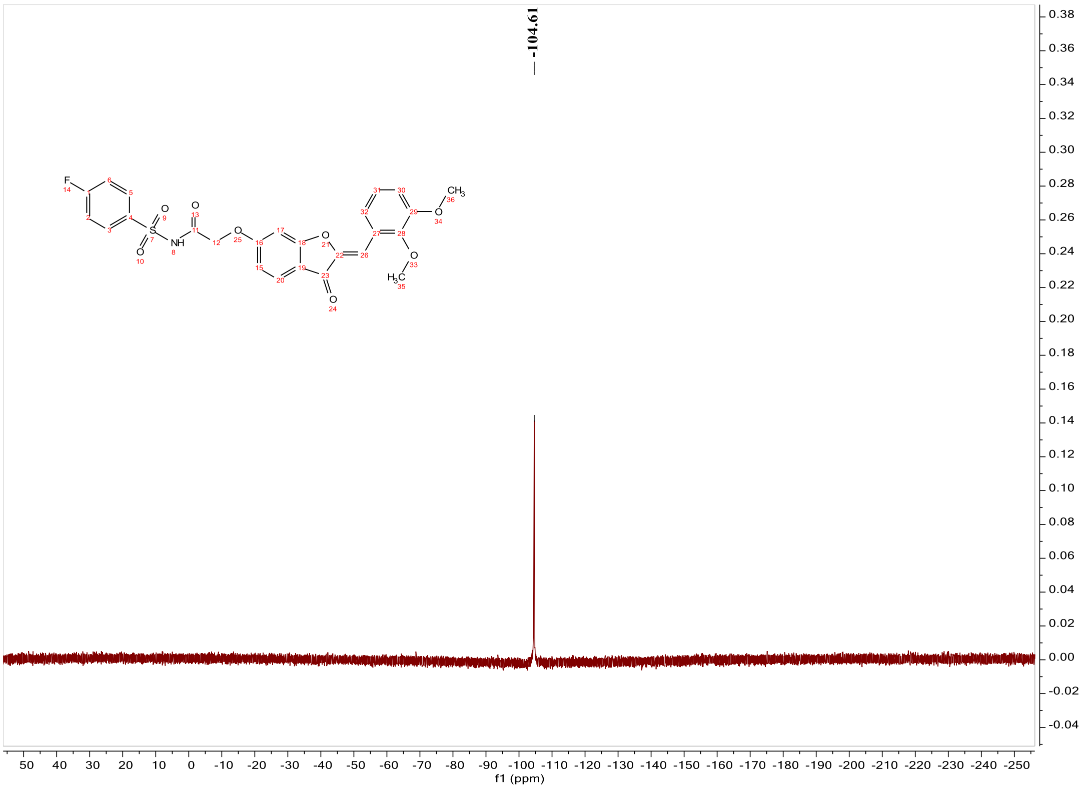

^{19}F NMR (471 MHz, DMSO-d_6) spectrum of compound **D27**

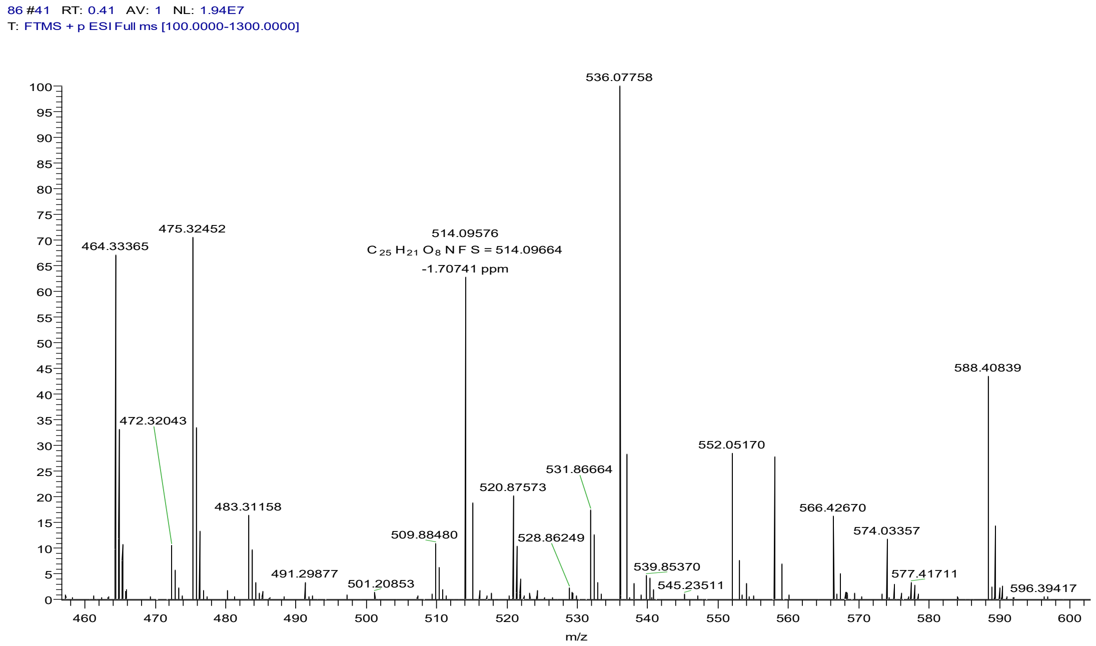

HRMS spectrum of compound **D27**

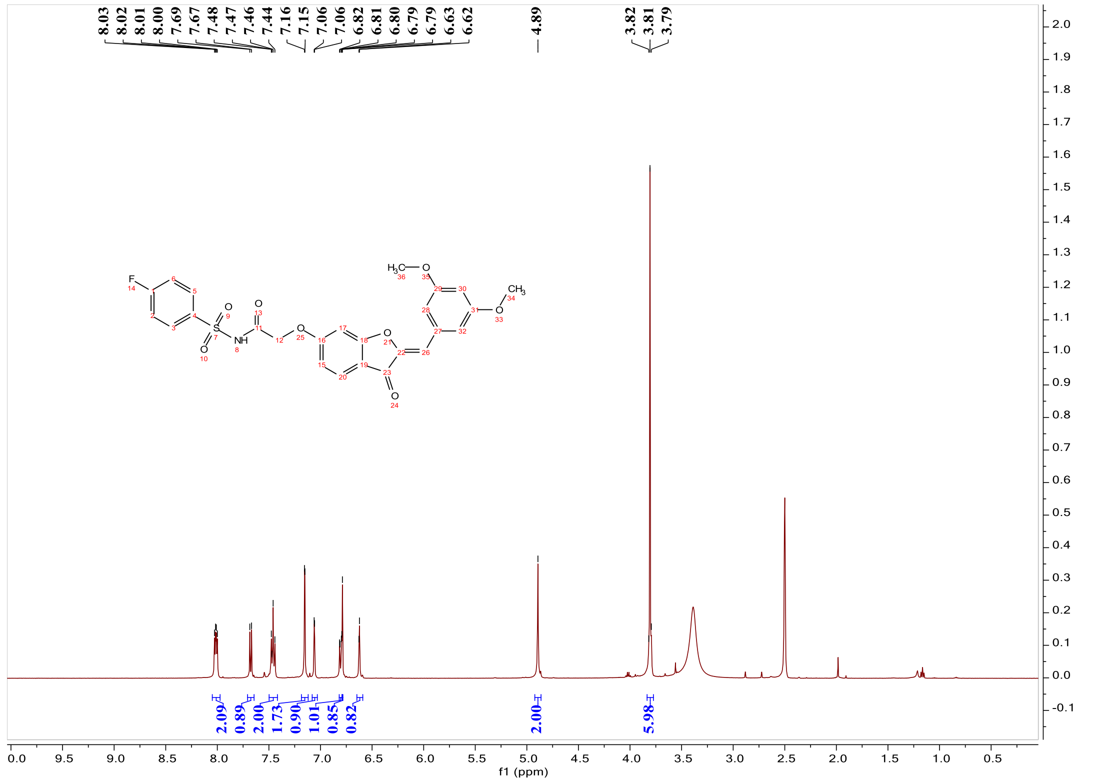

^{1}H NMR (500 MHz, DMSO-d_6) spectrum of compound **D28**

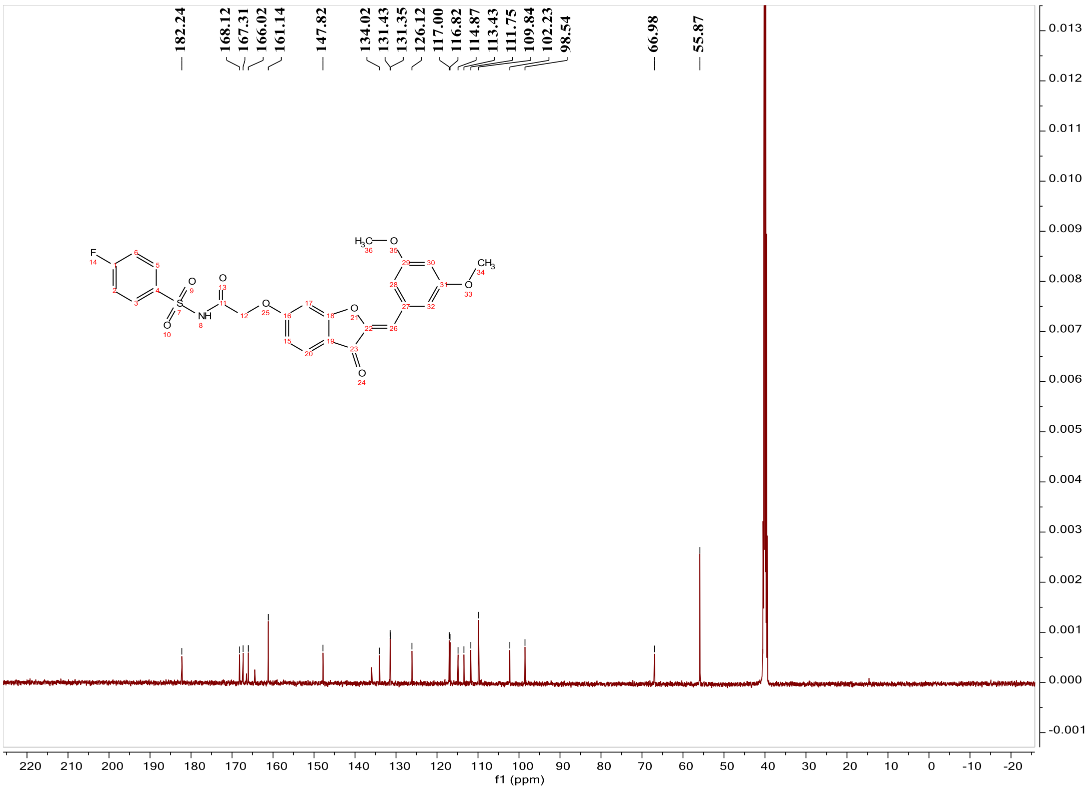

^{13}C NMR (126 MHz, DMSO-d_6) spectrum of compound **D28**

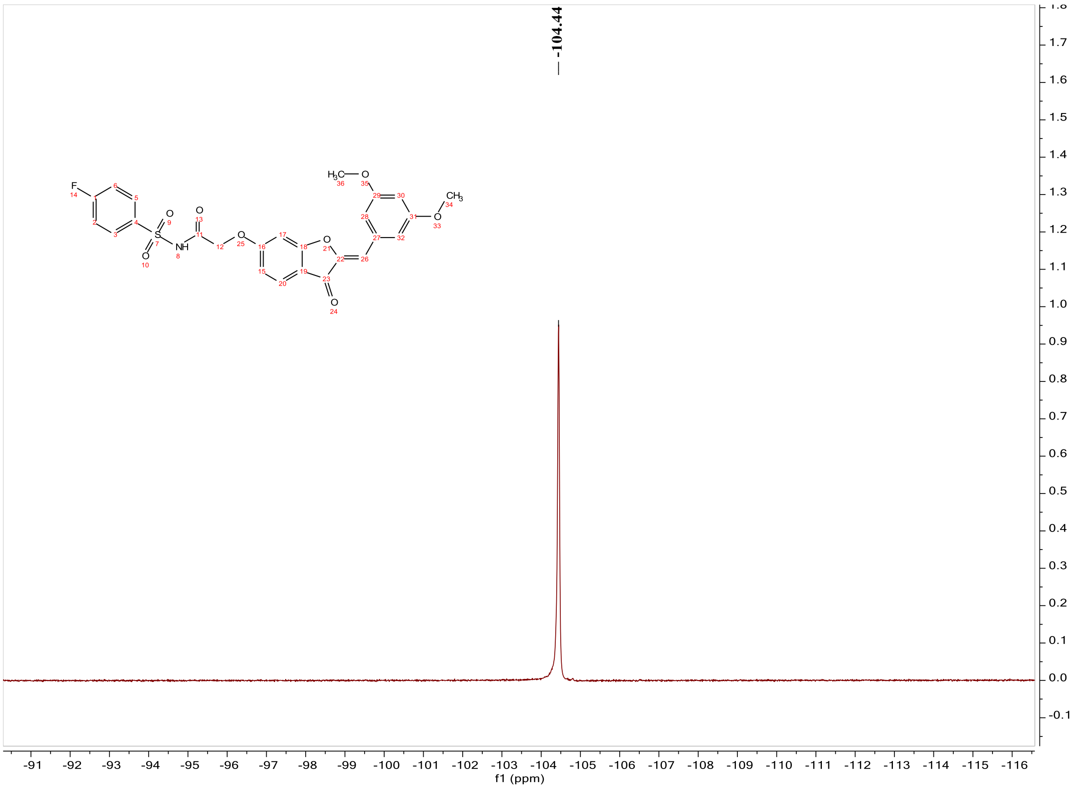

^{19}F NMR (471 MHz, DMSO-d_6) spectrum of compound **D28**

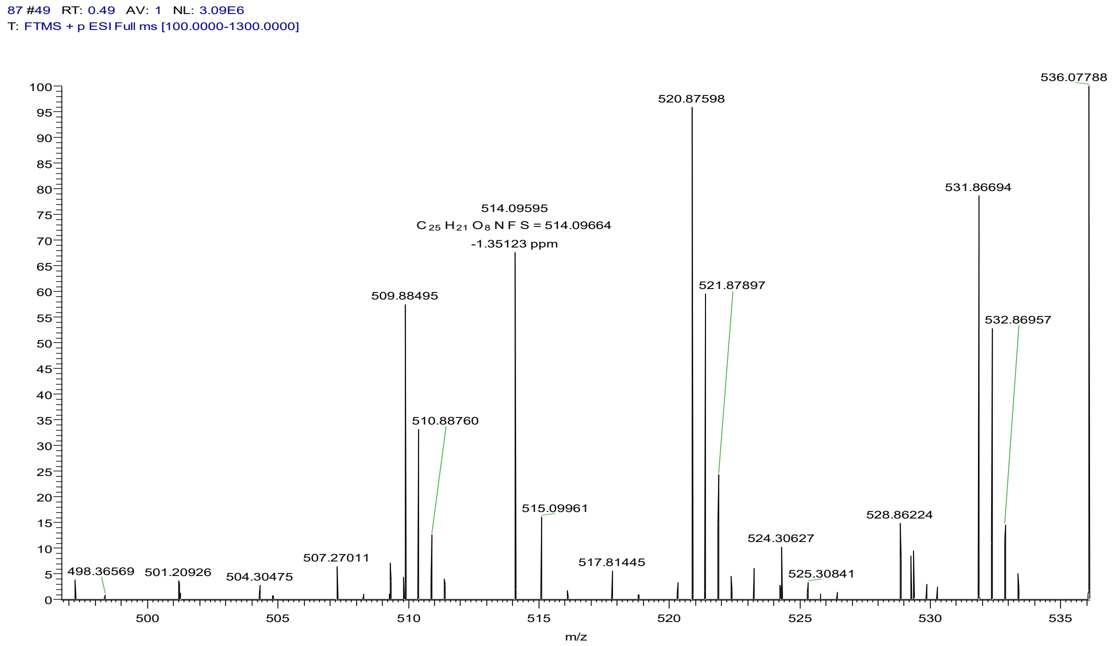

HRMS spectrum of compound **D28**

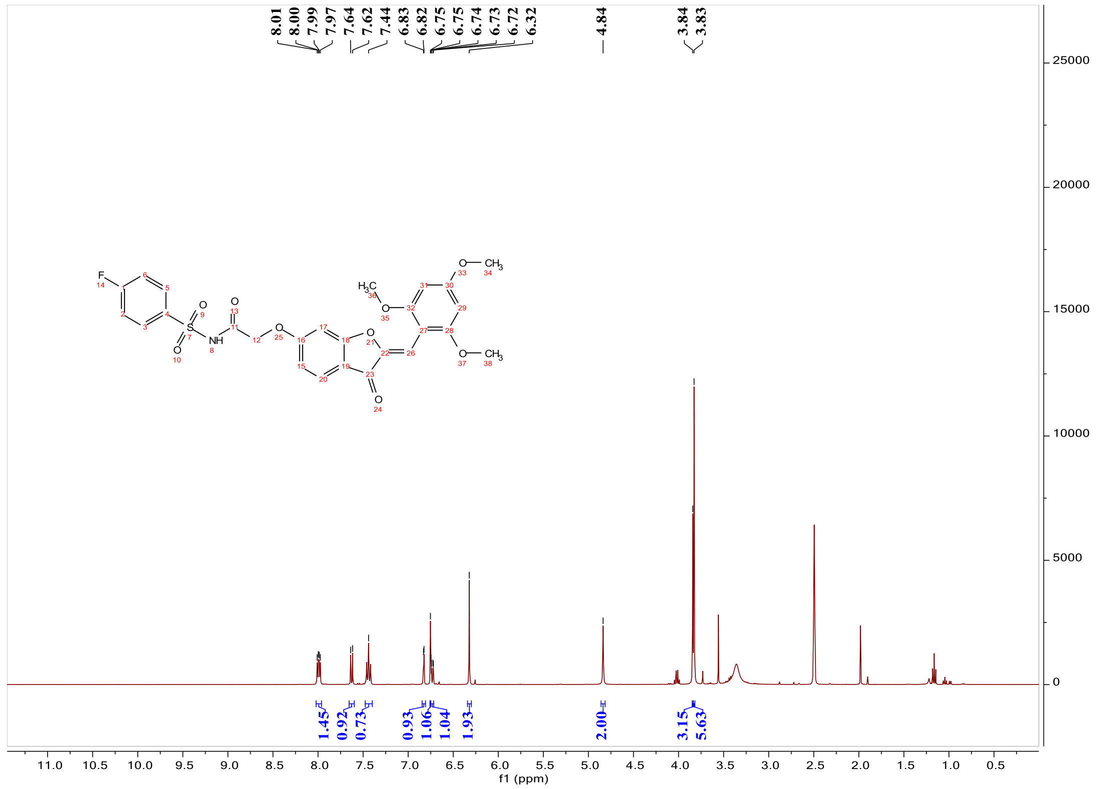

^{1}H NMR (400 MHz, DMSO-d_6) spectrum of compound **D29**

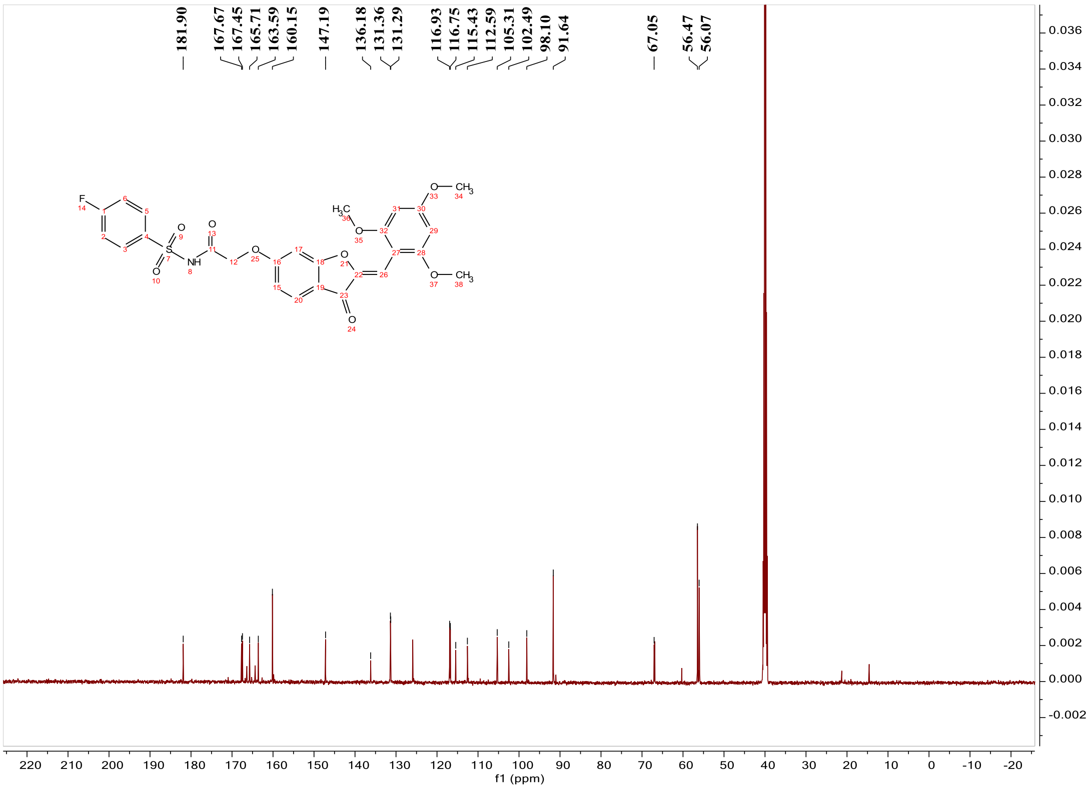

^{13}C NMR (126 MHz, DMSO-d_6) spectrum of compound **D29**

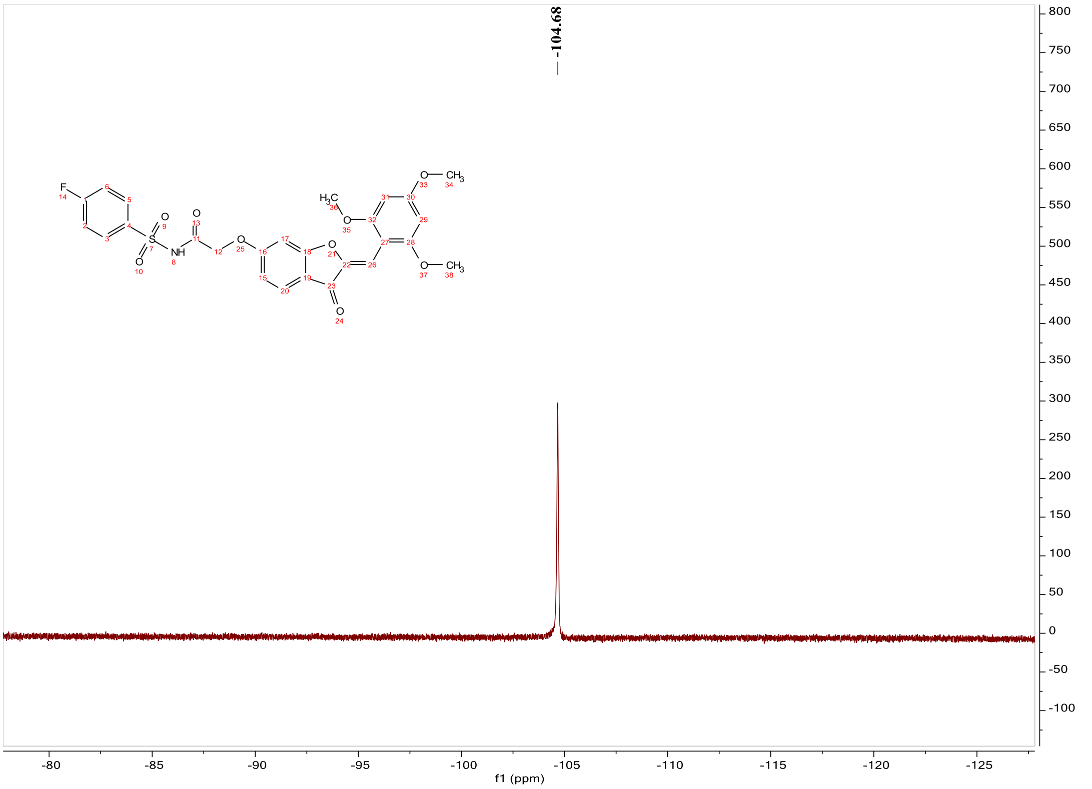

^{19}F NMR (376 MHz, DMSO-d_6) spectrum of compound **D29**

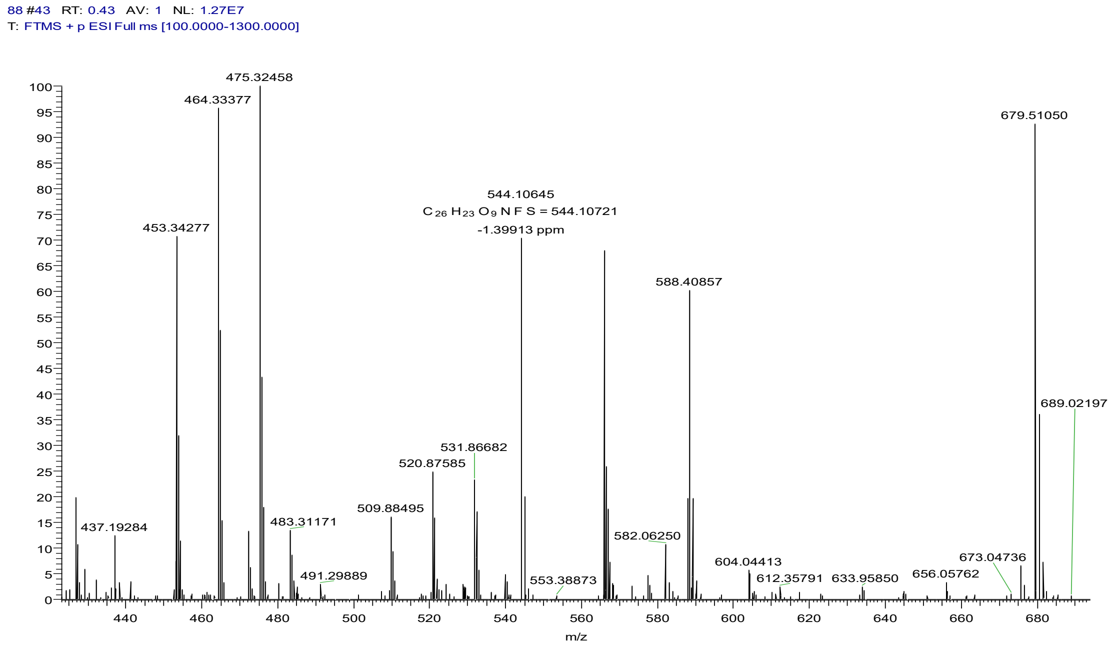

HRMS spectrum of compound **D29**

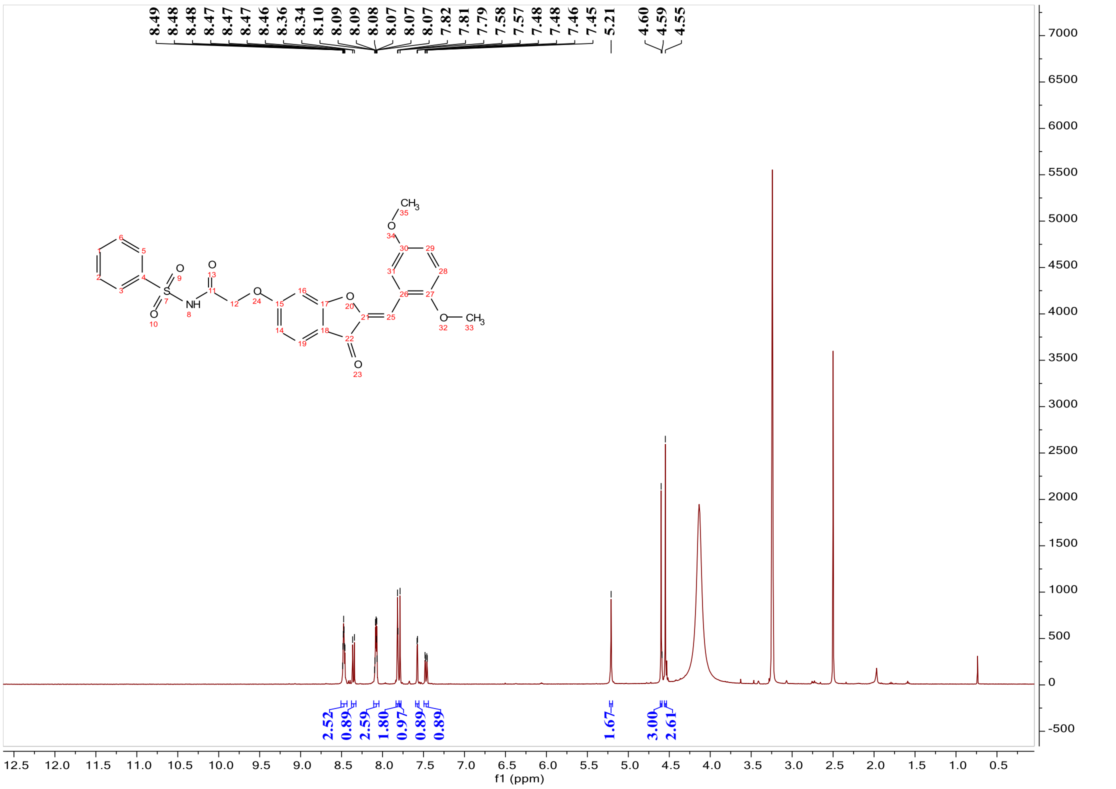

^{1}H NMR (400 MHz, DMSO-d_6) spectrum of compound **D30**

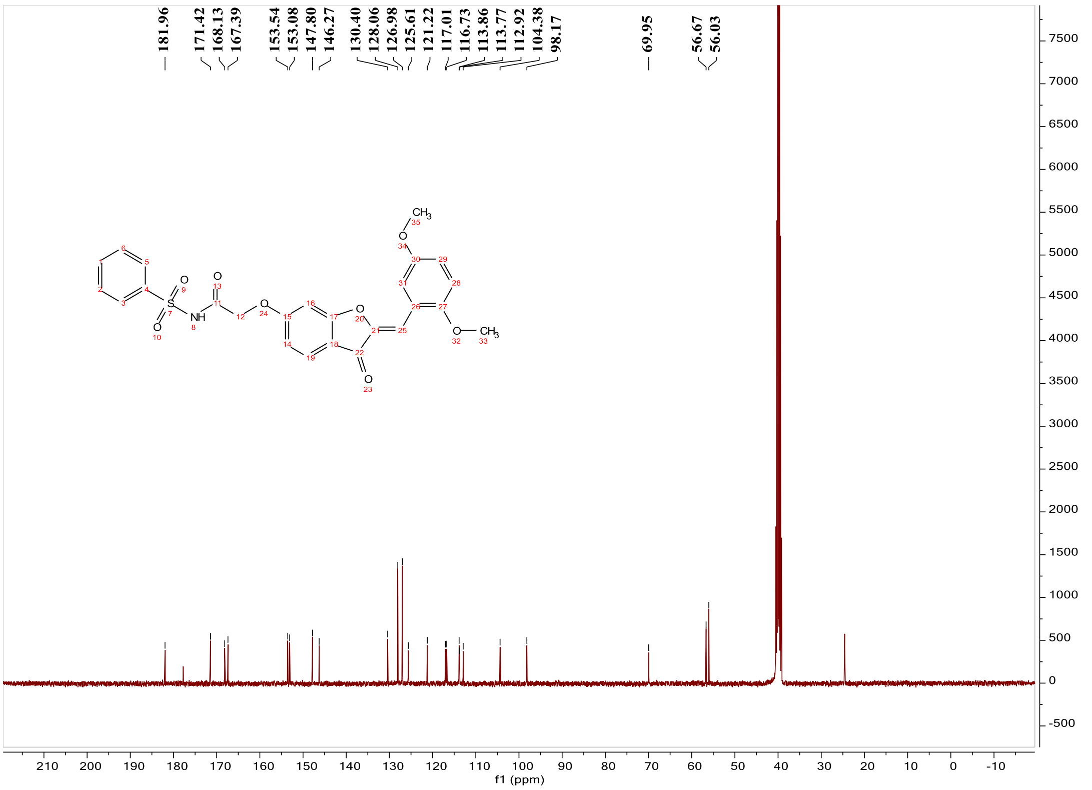

^{13}C NMR (101 MHz, DMSO-d_6) spectrum of compound **D30**

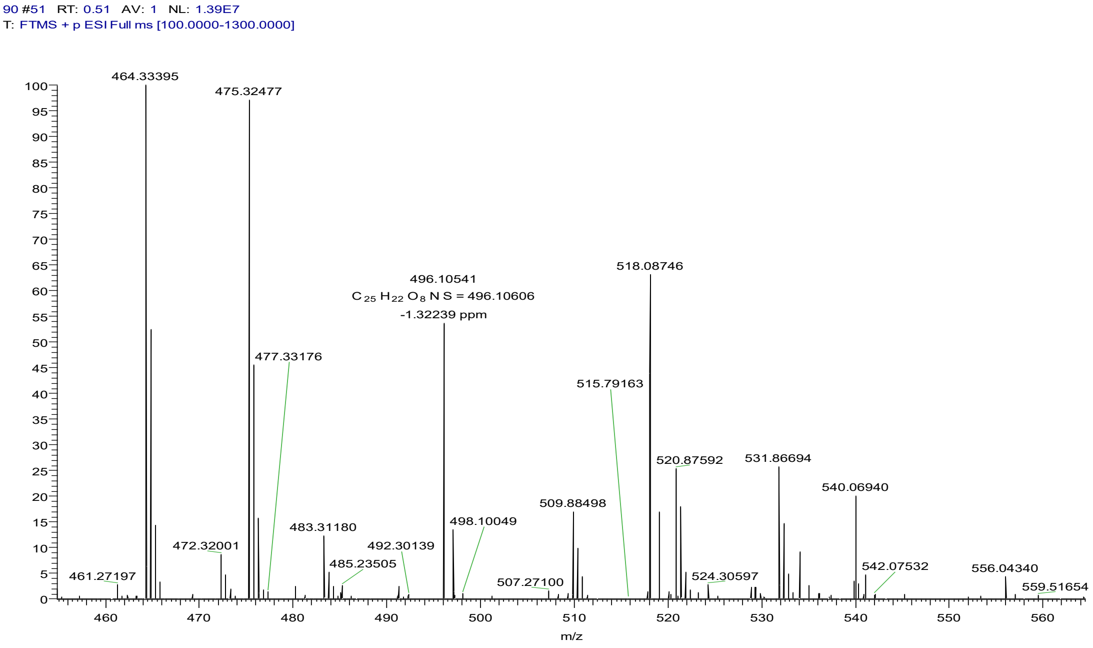

HRMS spectrum of compound **D30**

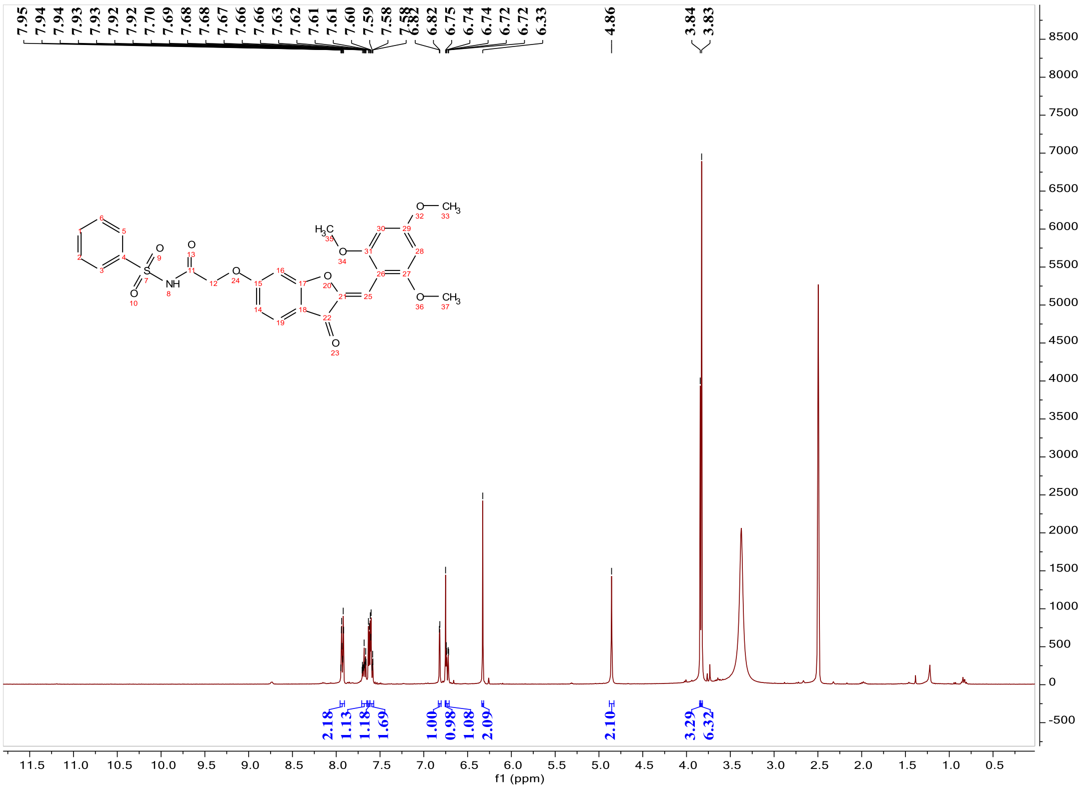

^{1}H NMR (400 MHz, DMSO-d_6) spectrum of compound **D31**

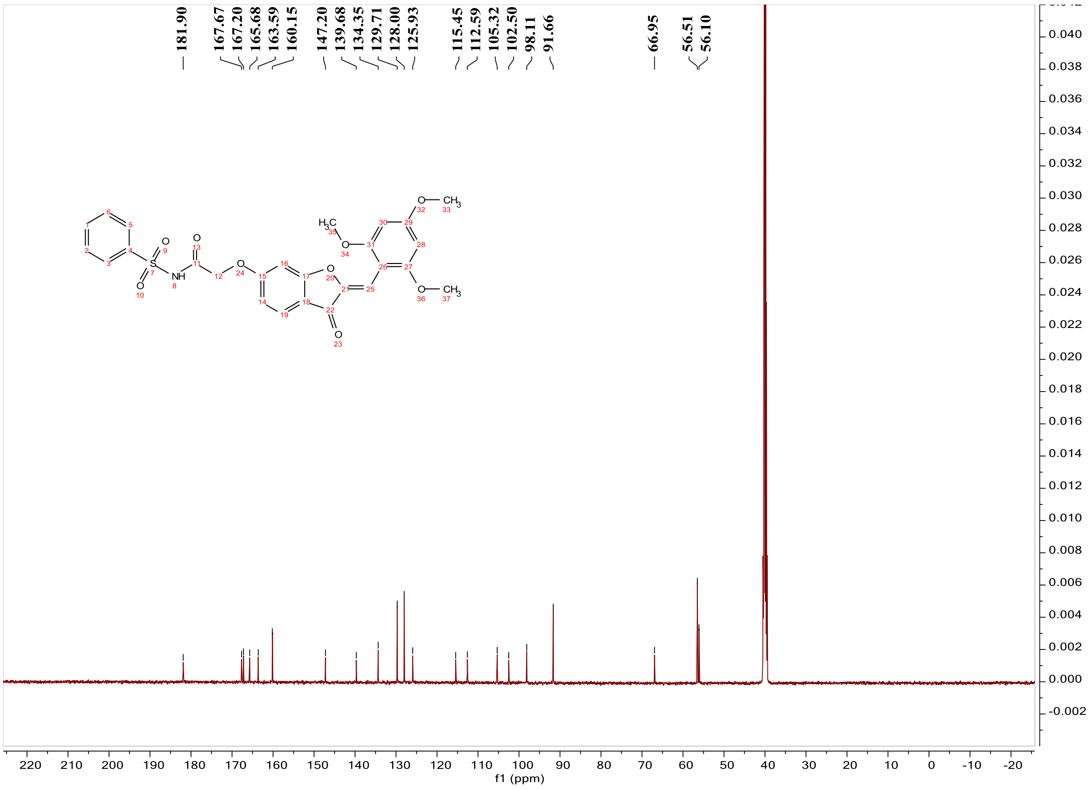

^{13}C NMR (126 MHz, DMSO-d_6) spectrum of compound **D31**

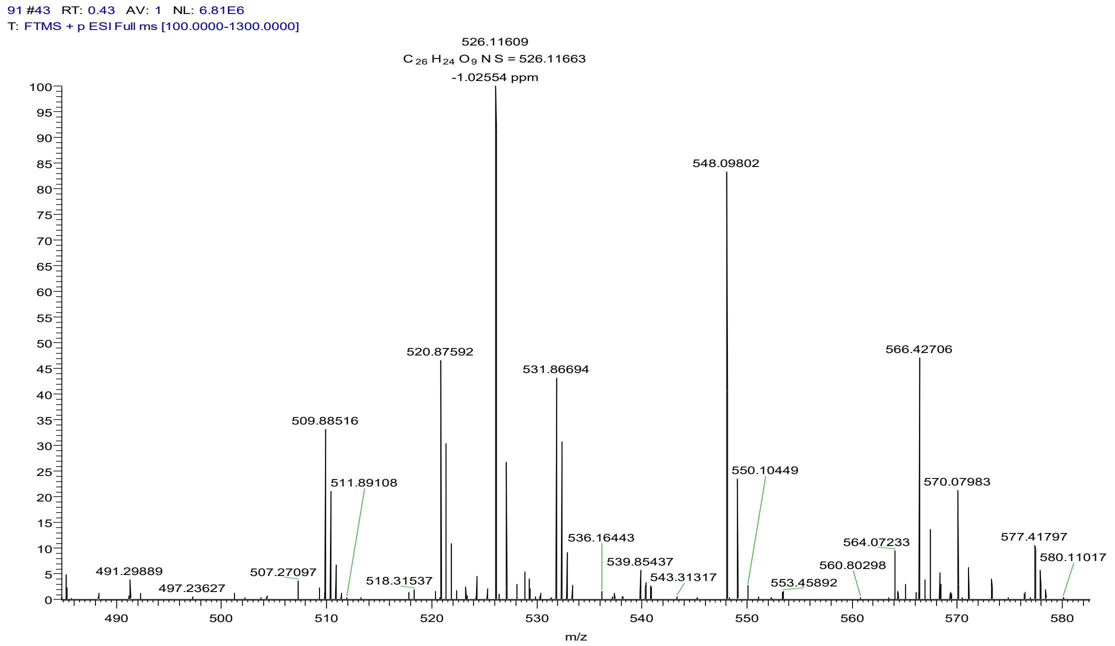

HRMS spectrum of compound **D31**

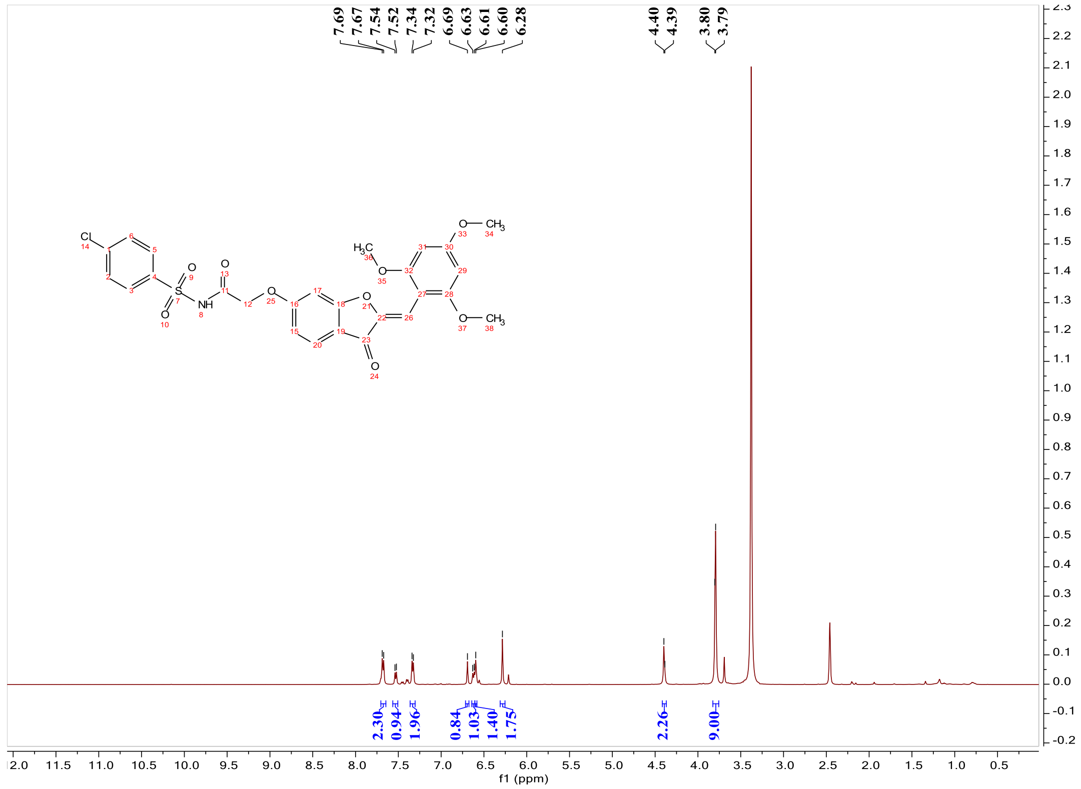

^{1}H NMR (500 MHz, DMSO-d_6) spectrum of compound **D32**

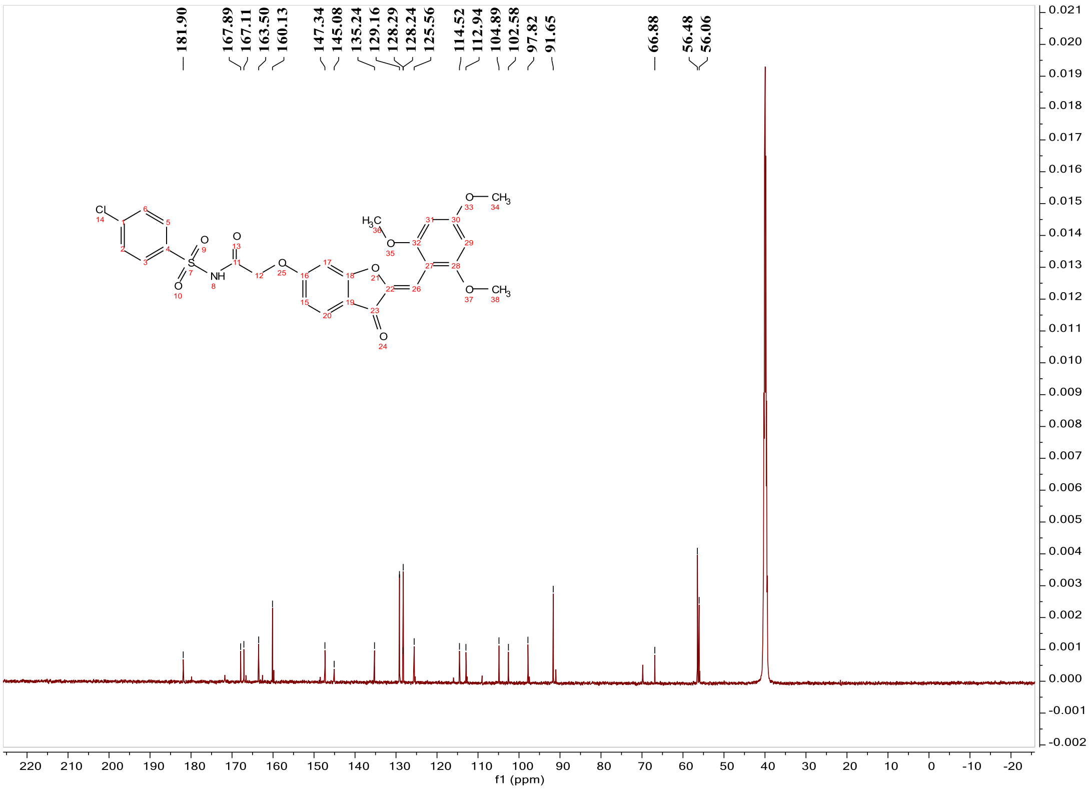

^{13}C NMR (126 MHz, DMSO-d_6) spectrum of compound **D32**

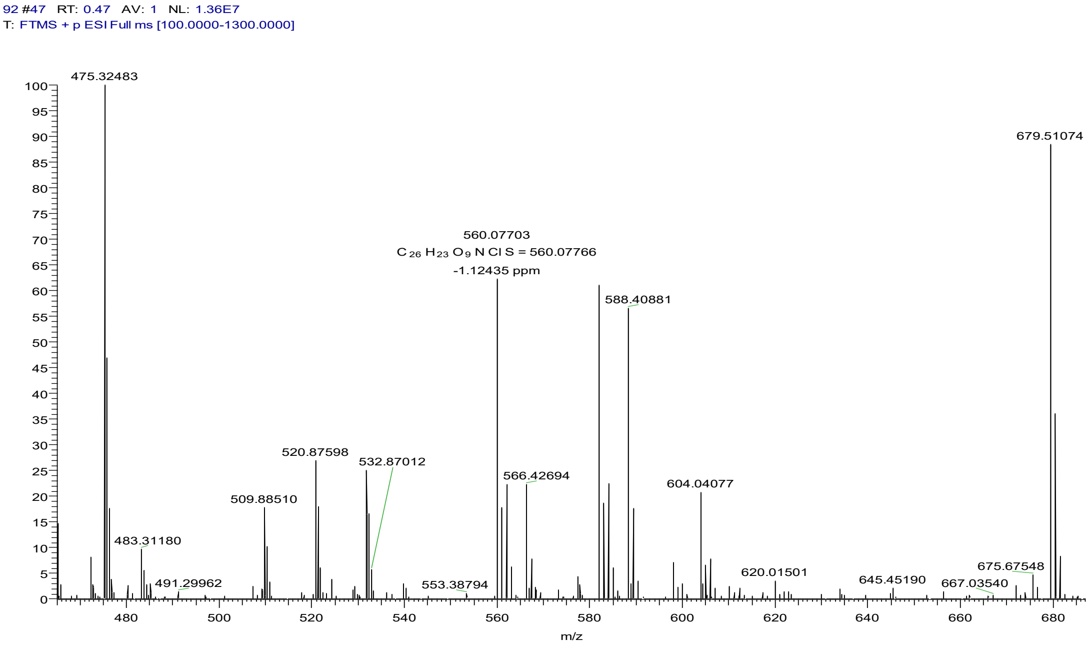

HRMS spectrum of compound **D32**

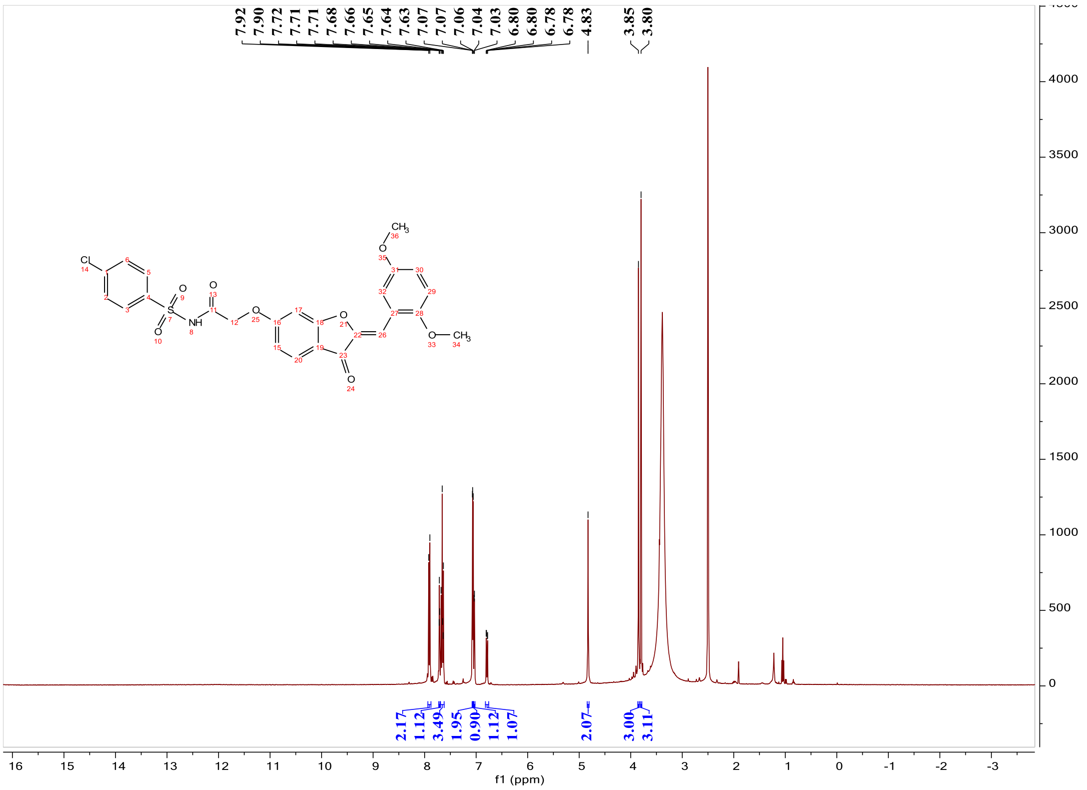

^{1}H NMR (400 MHz, DMSO-d_6) spectrum of compound **D33**

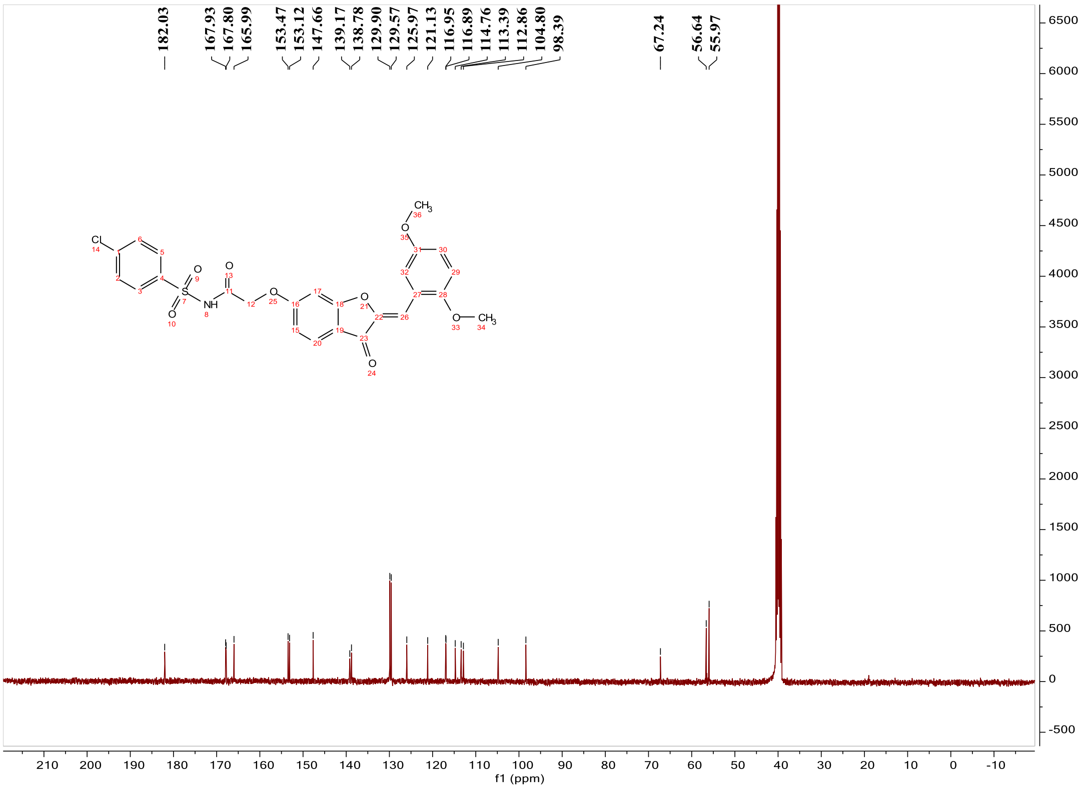

^{13}C NMR (101 MHz, DMSO-d_6) spectrum of compound **D33**

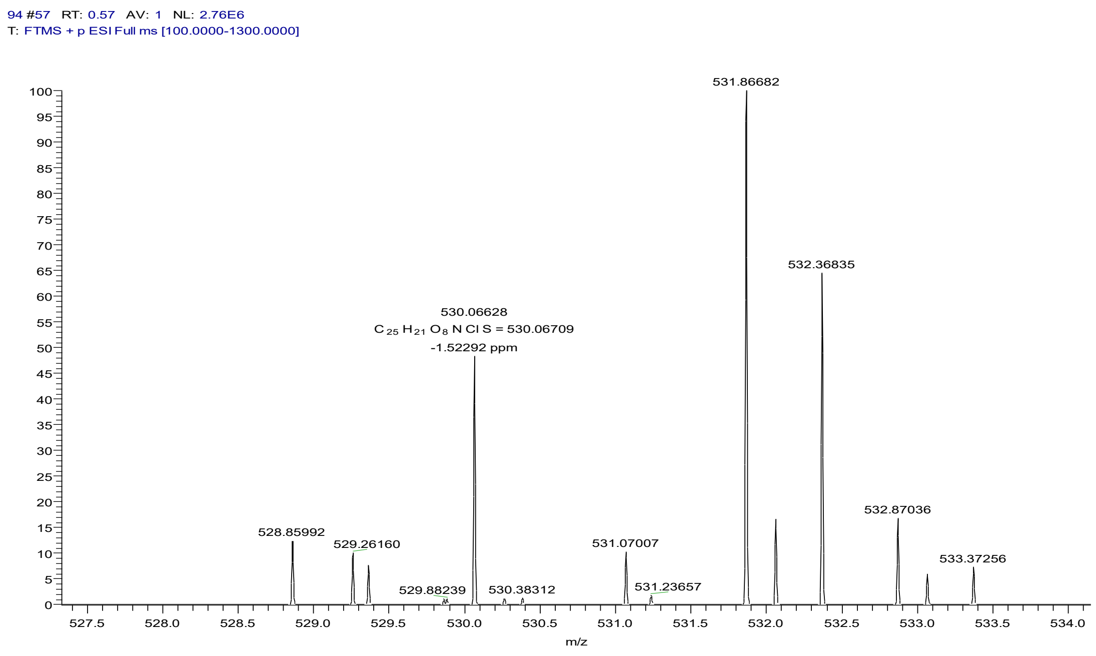

HRMS spectrum of compound **D33**

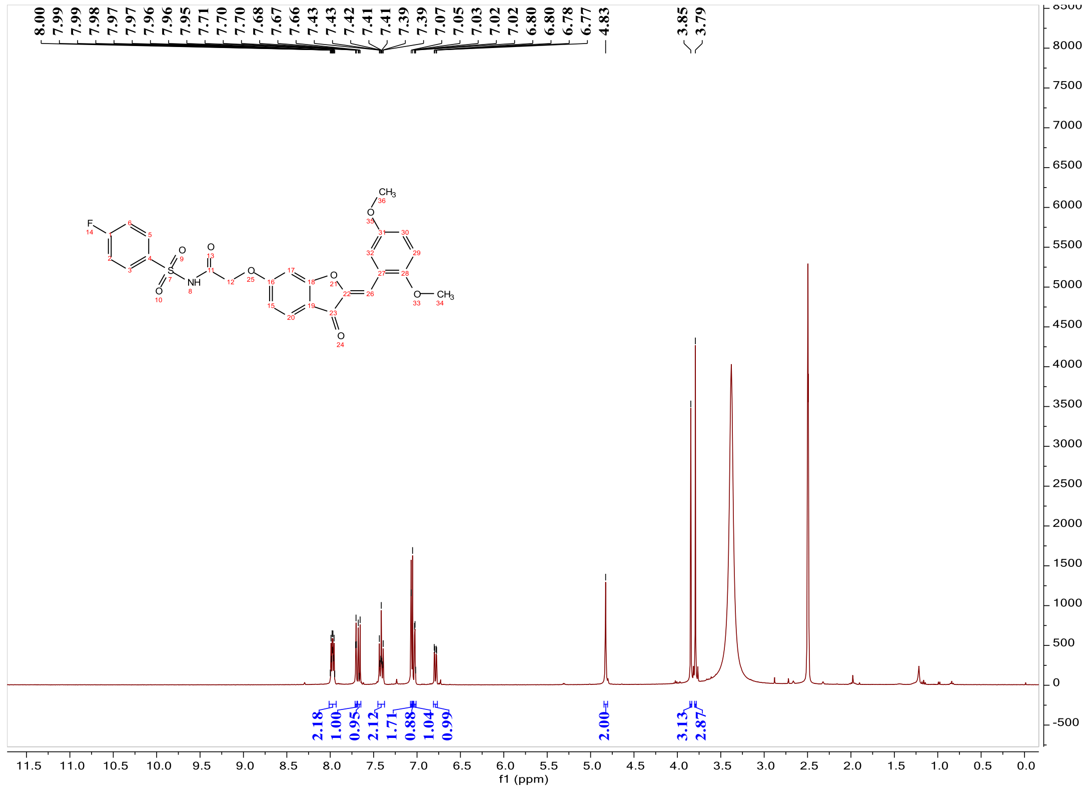

^{1}H NMR (400 MHz, DMSO-d_6) spectrum of compound **D34**

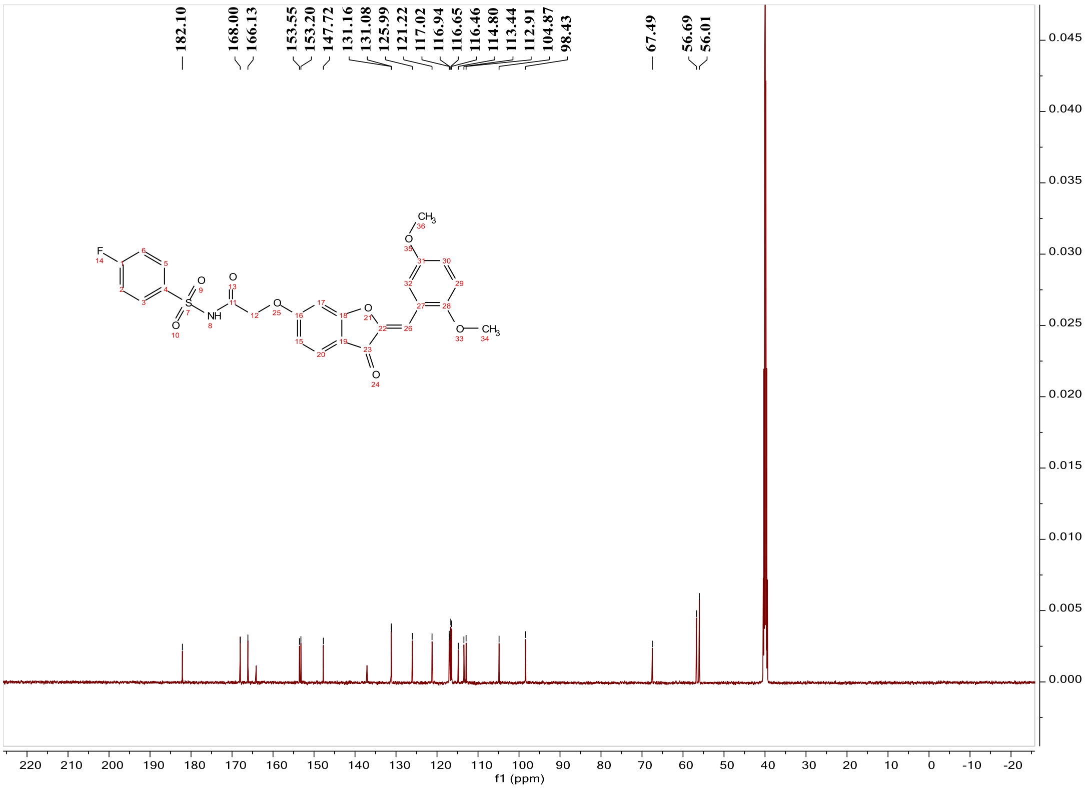

^{13}C NMR (126 MHz, DMSO-d_6) spectrum of compound **D34**

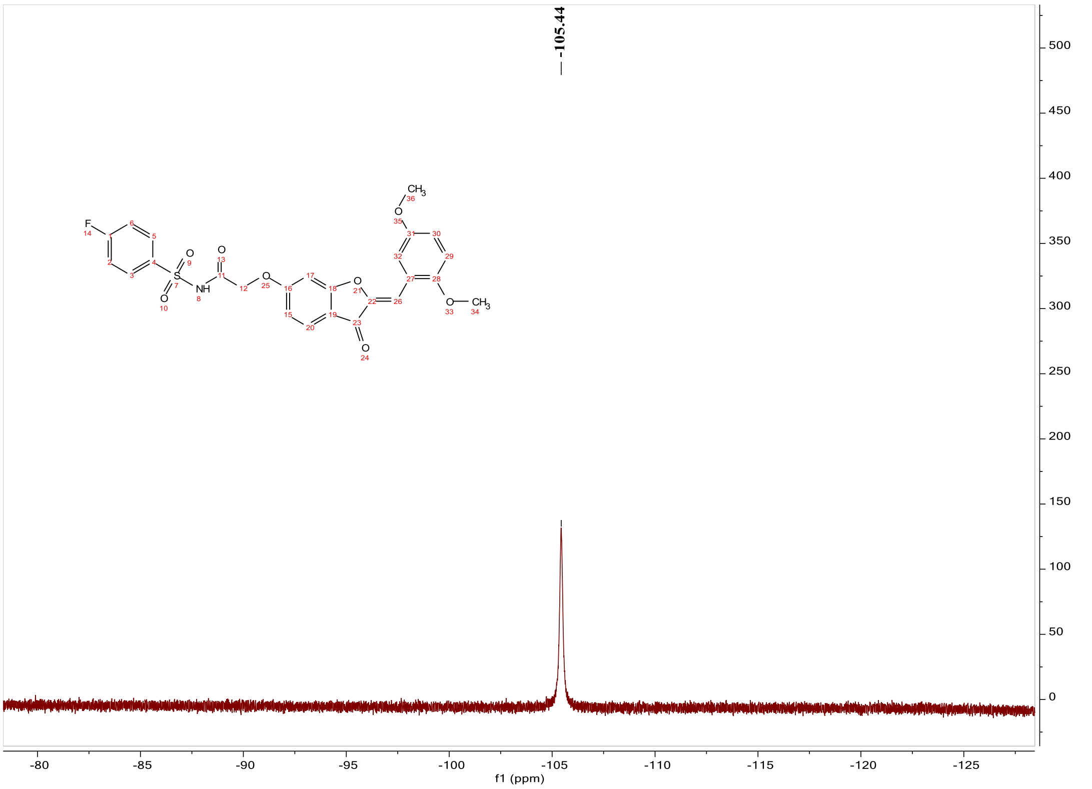

^{19} F NMR (376 MHz, DMSO-d_6) spectrum of compound **D34**

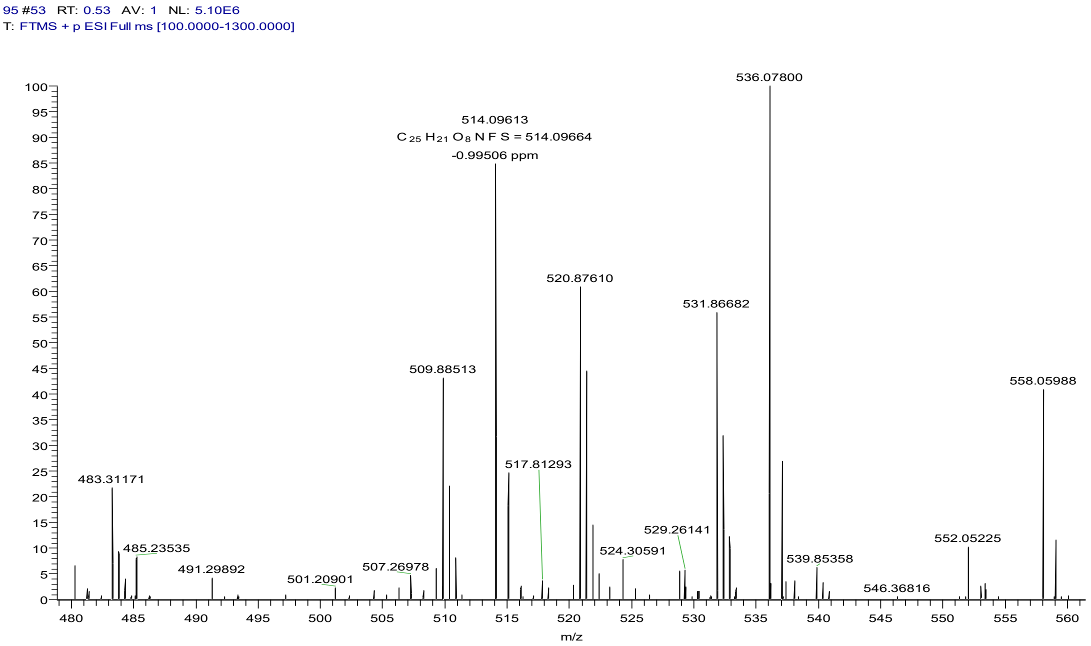

HRMS spectrum of compound **D34**

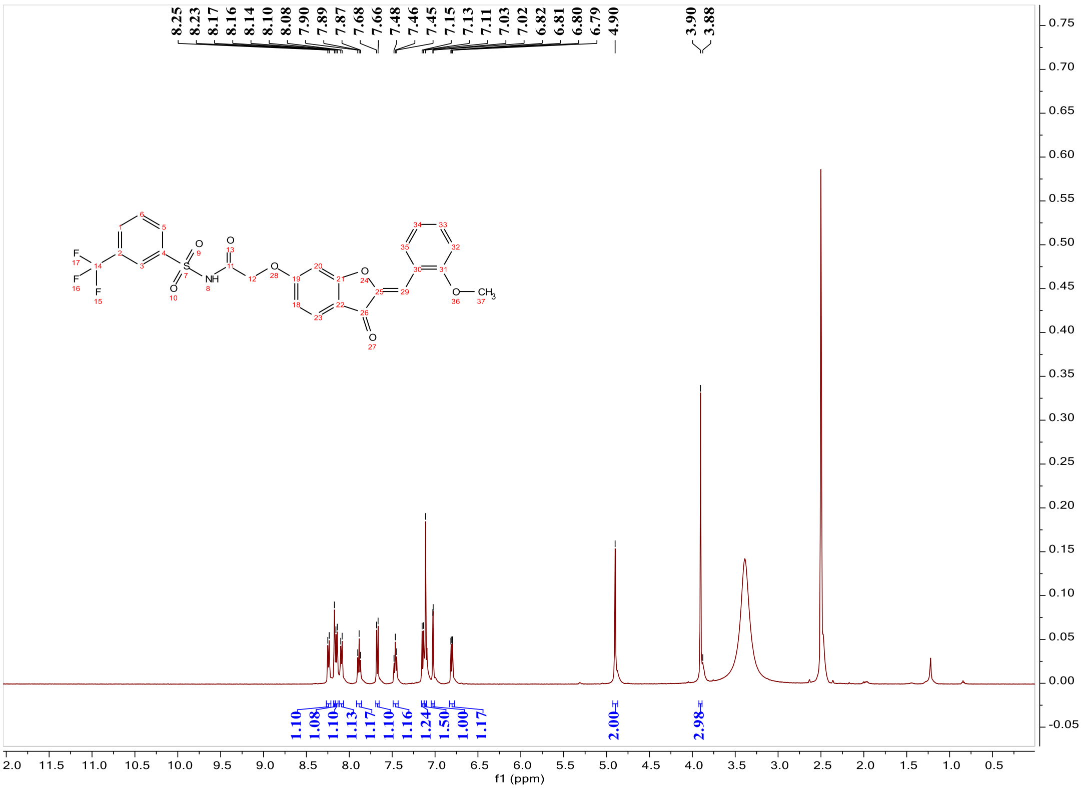

^{1}H NMR (500 MHz, DMSO-d_6) spectrum of compound **D35**

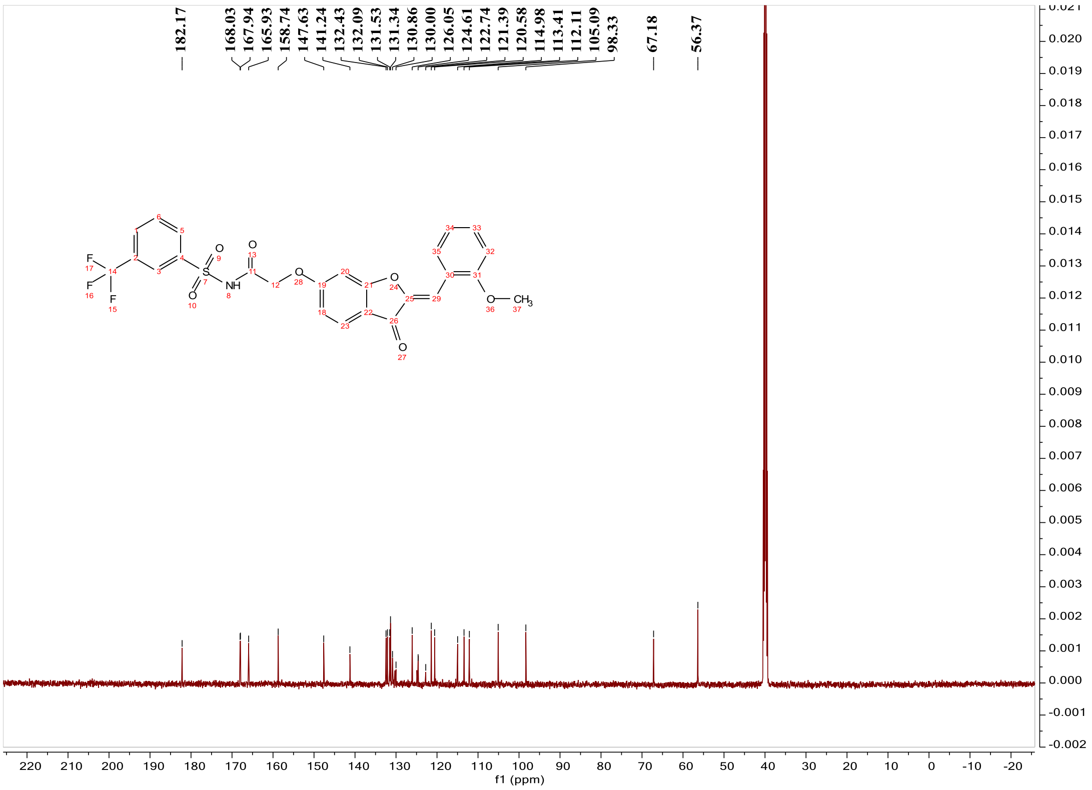

^{13}C NMR (126 MHz, DMSO-d_6) spectrum of compound **D35**

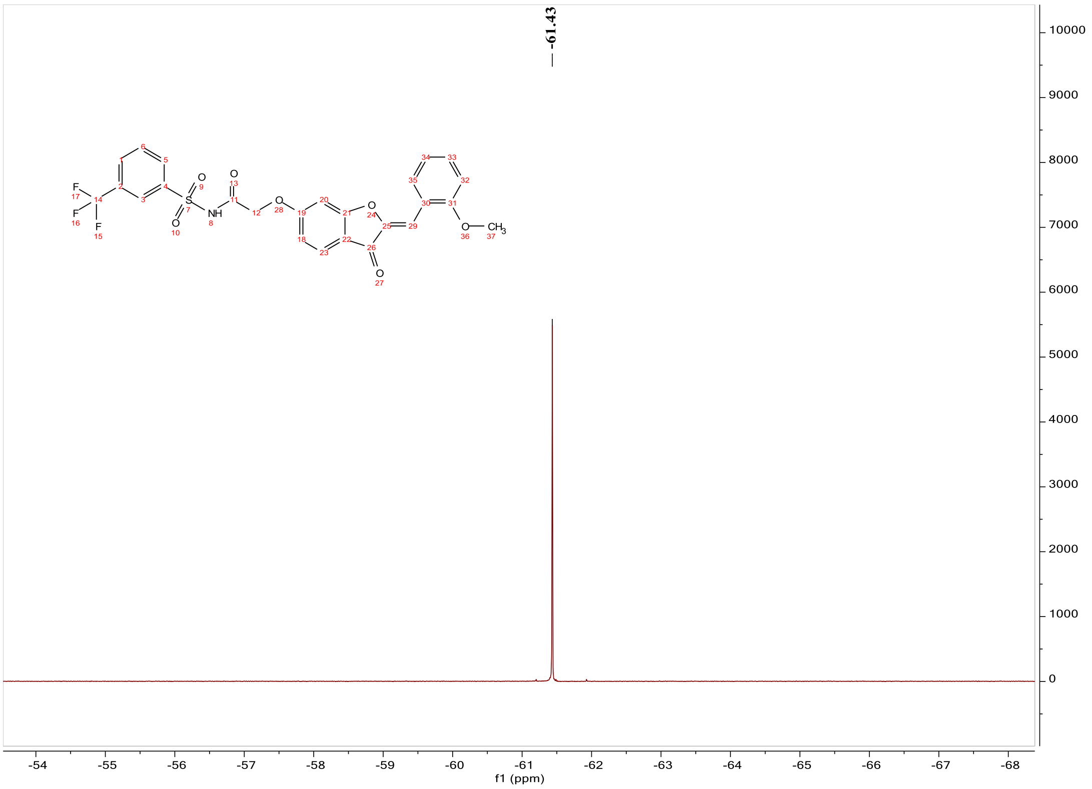

^{19}F NMR (376 MHz, DMSO-d_6) spectrum of compound **D35**

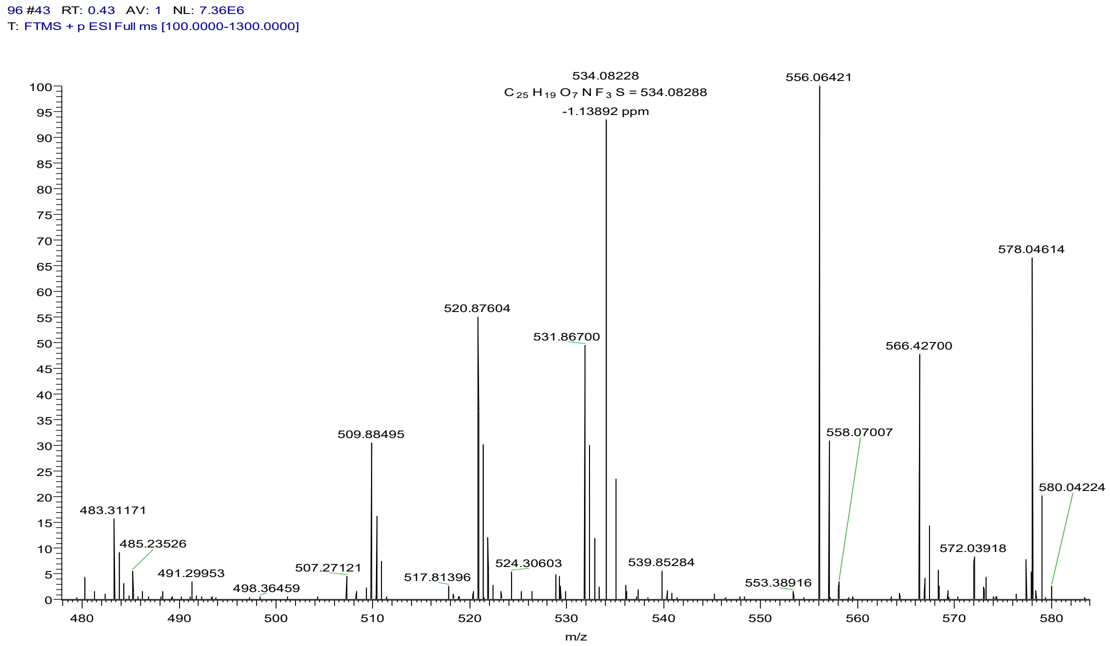

HRMS spectrum of compound **D35**

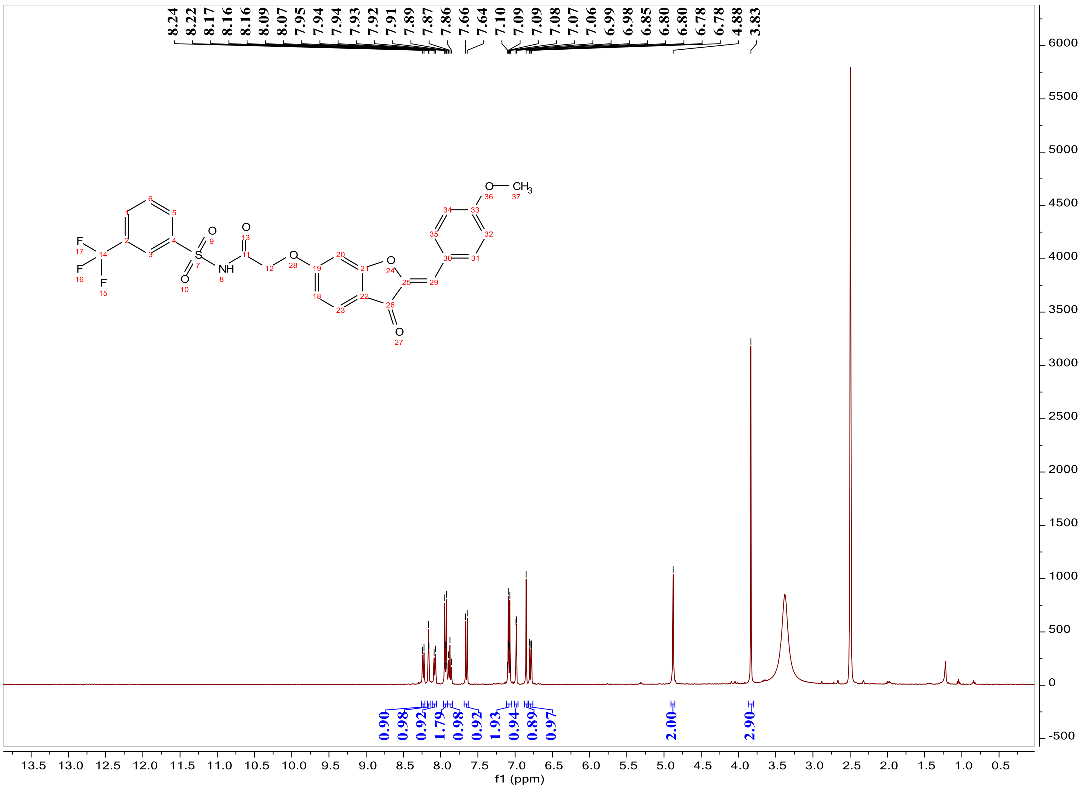

^{1}H NMR (400 MHz, DMSO-d_6) spectrum of compound **D36**

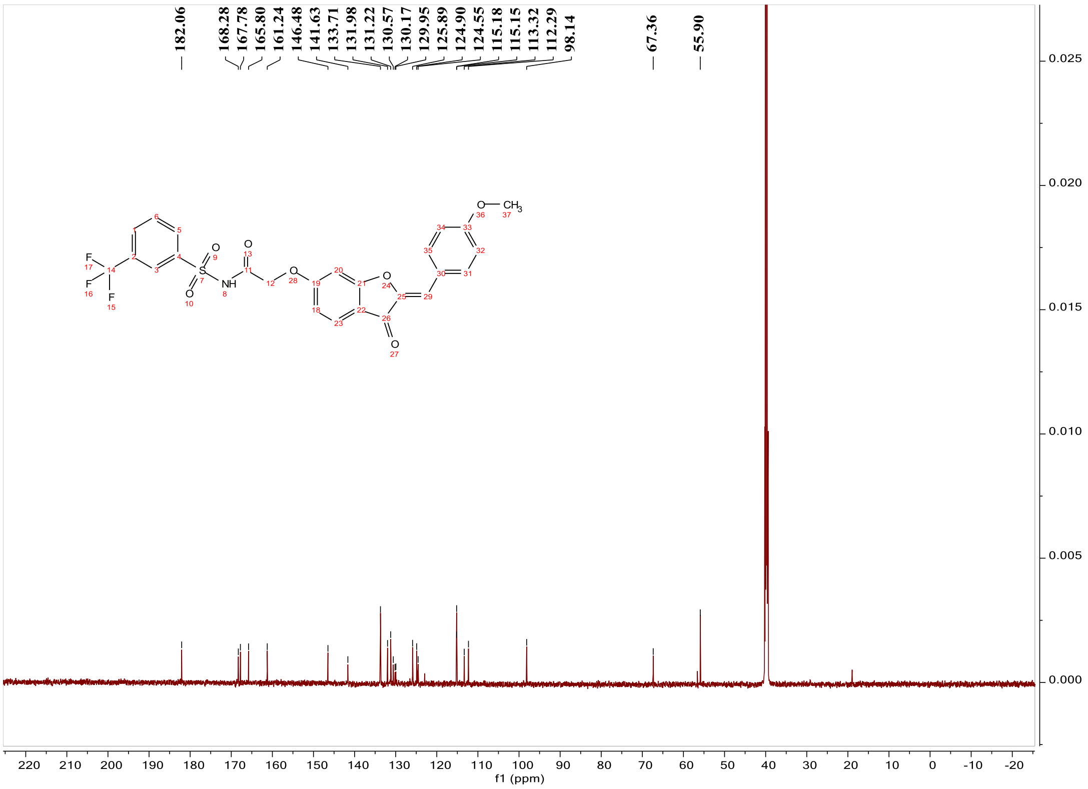

^{13}C NMR (151 MHz, DMSO-d_6) spectrum of compound **D36**

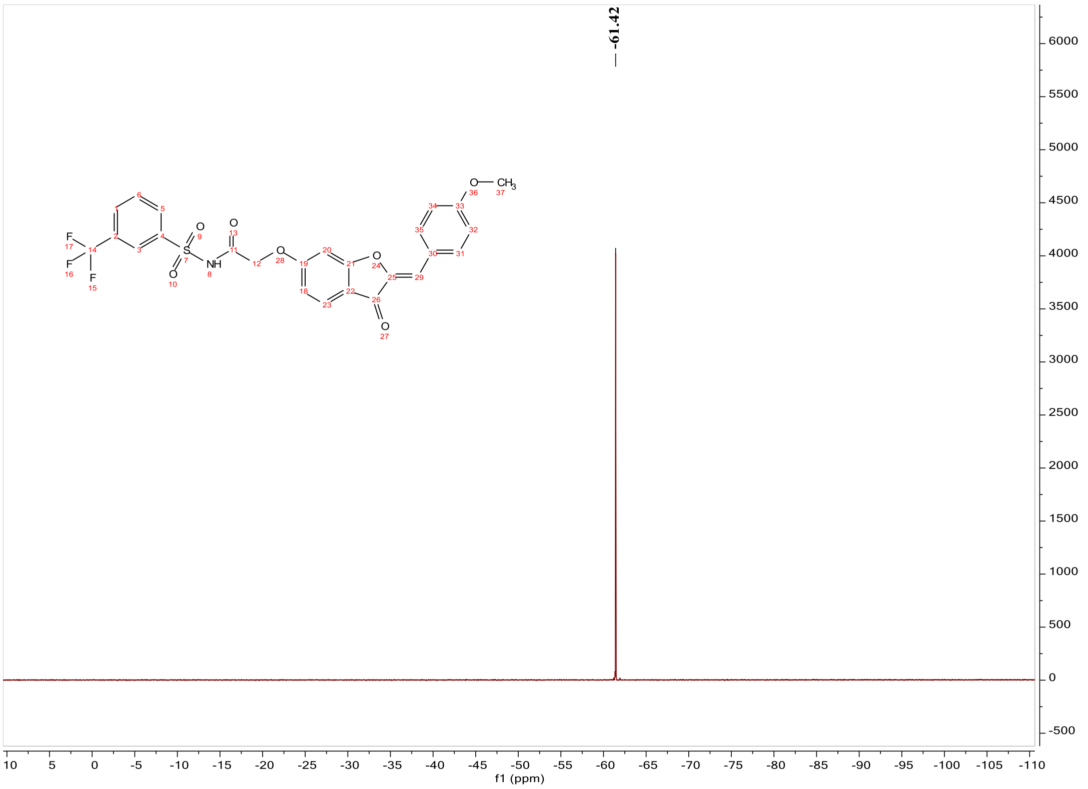

^{19}F NMR (376 MHz, DMSO-d_6) spectrum of compound **D36**

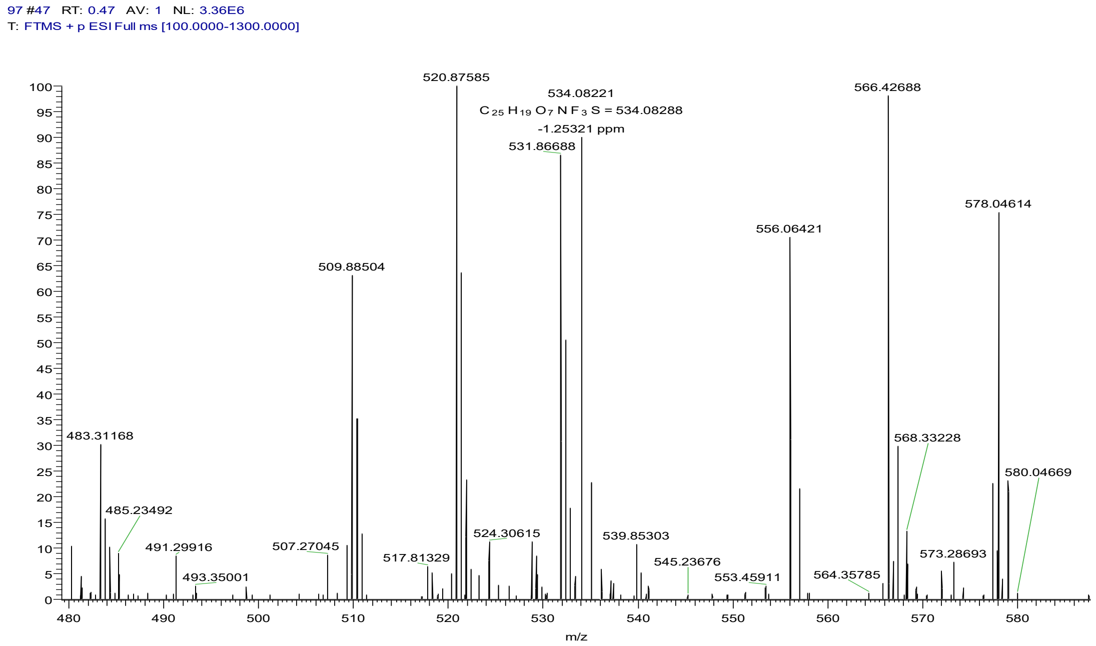

HRMS spectrum of compound **D36**

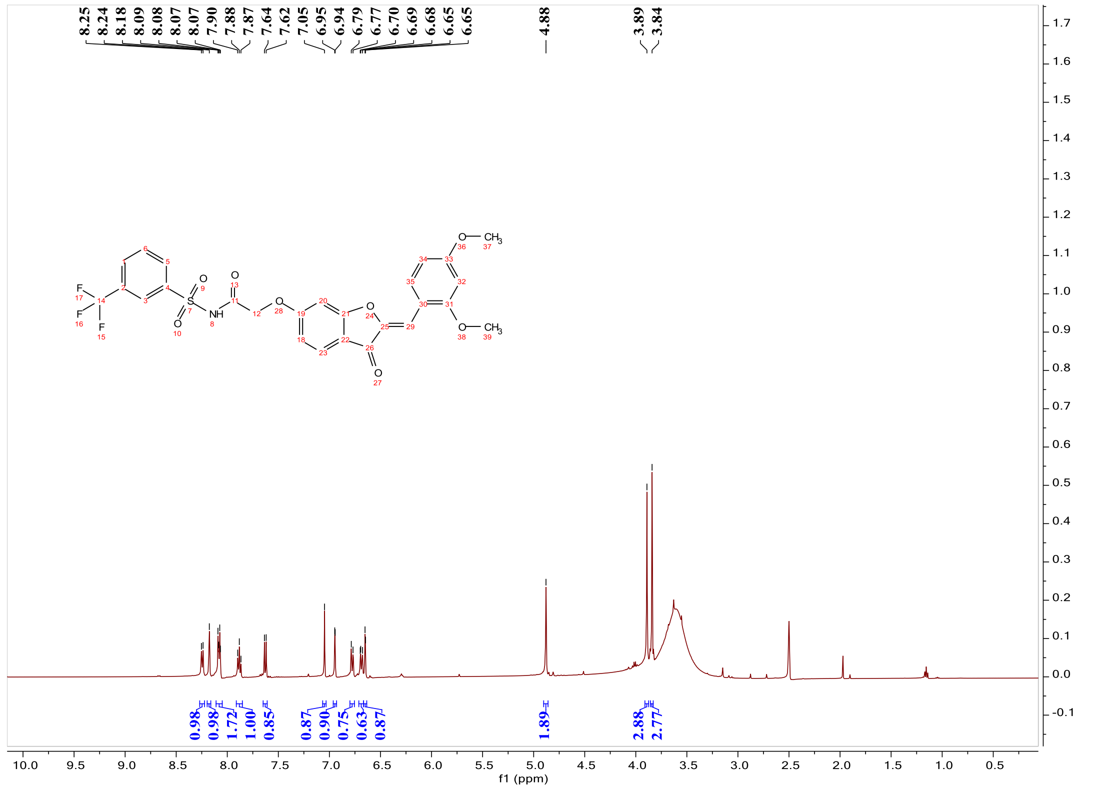

^{1}H NMR (500 MHz, DMSO-d_6) spectrum of compound **D37**

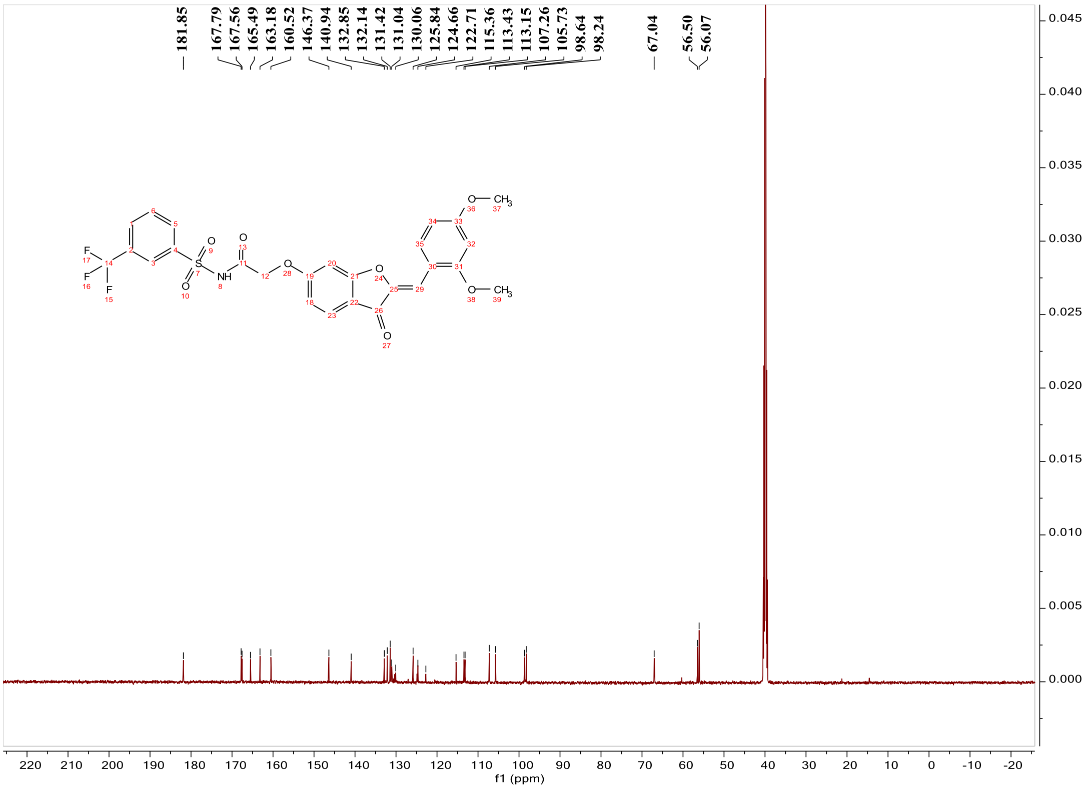

^{13}C NMR (126 MHz, DMSO-d_6) spectrum of compound **D37**

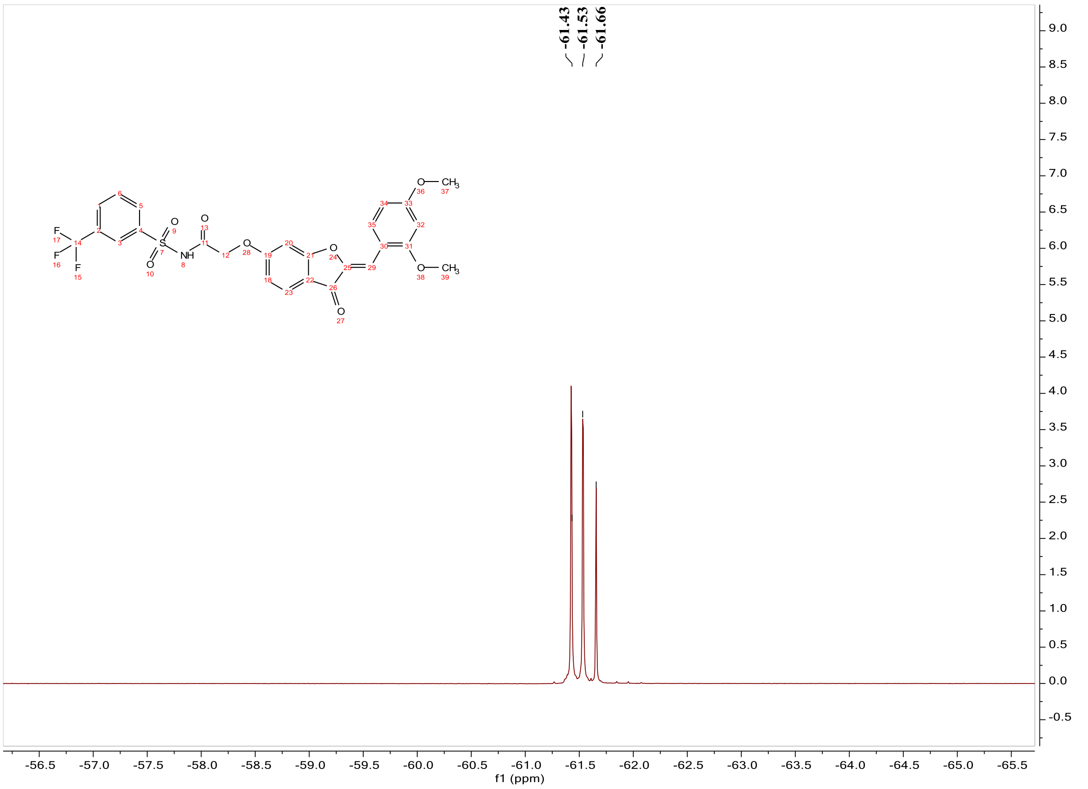

^{19}F NMR (471 MHz, DMSO-d_6) spectrum of compound **D37**

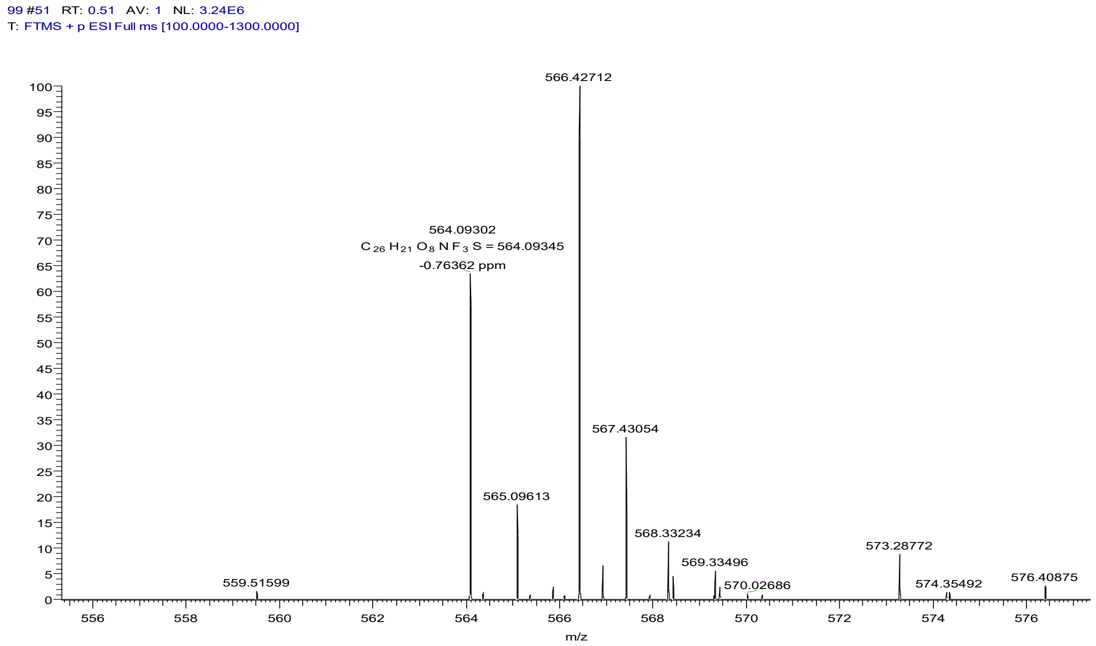

HRMS spectrum of compound **D37**

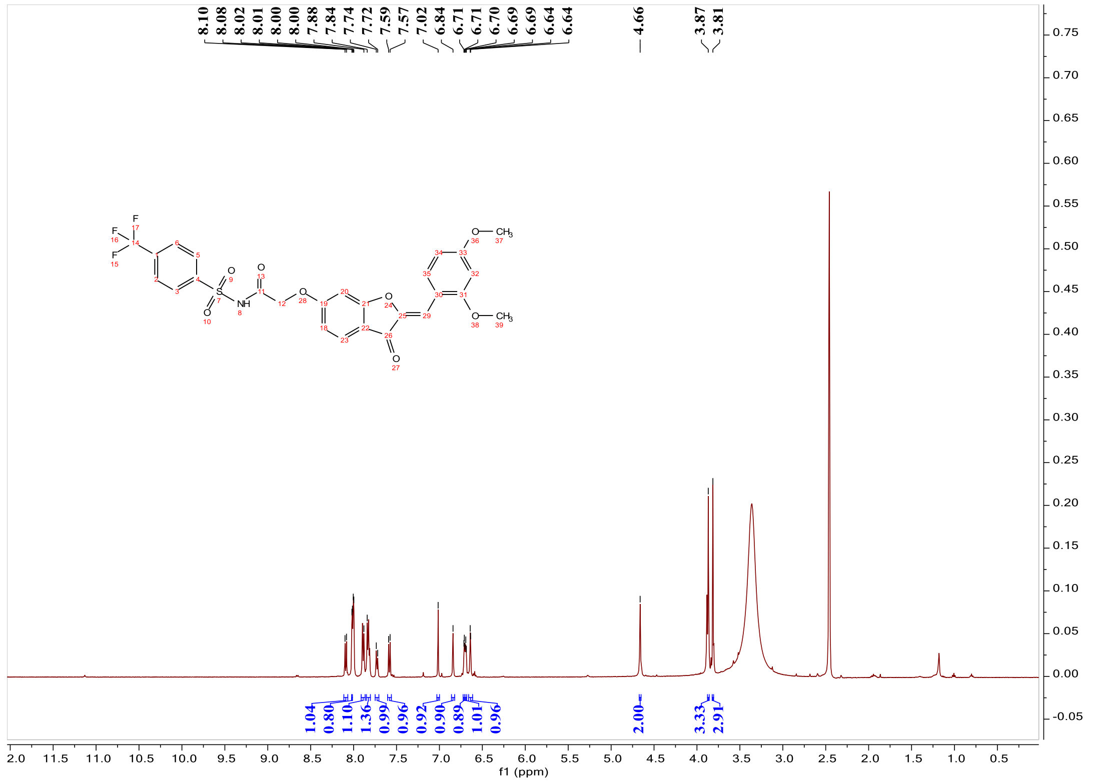

^{1}H NMR (500 MHz, DMSO-d_6) spectrum of compound **D38**

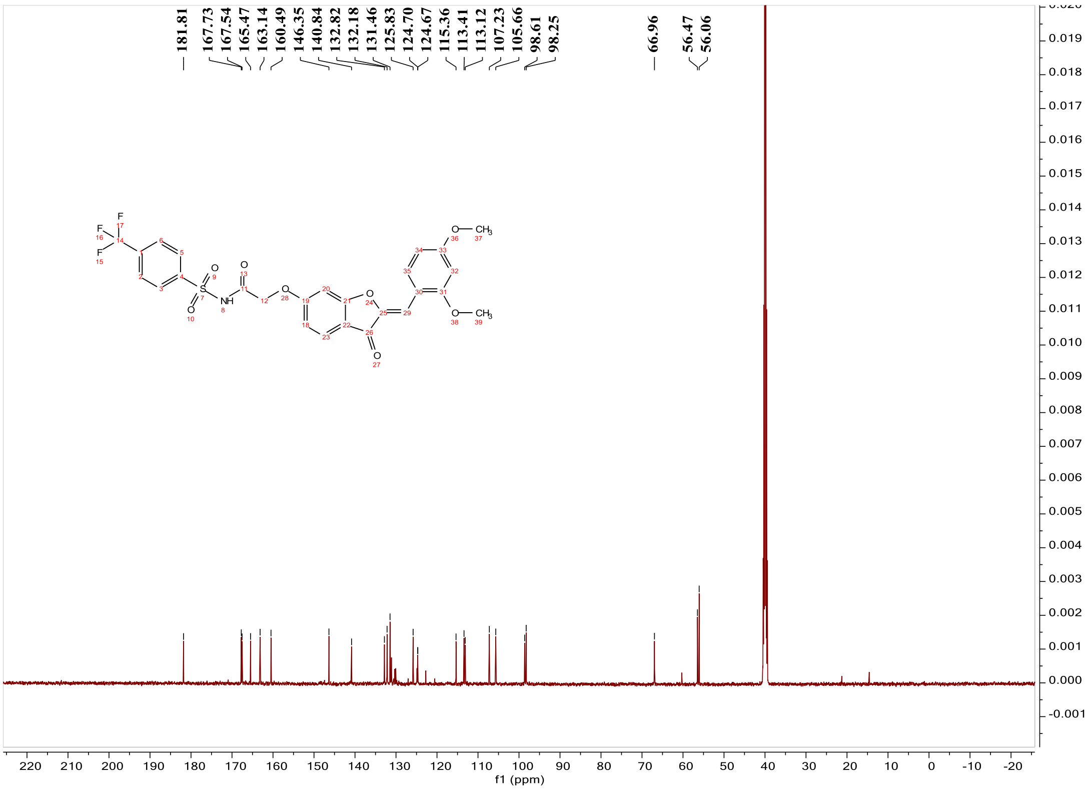

^{13}C NMR (100 MHz, DMSO-d_6) spectrum of compound **D38**

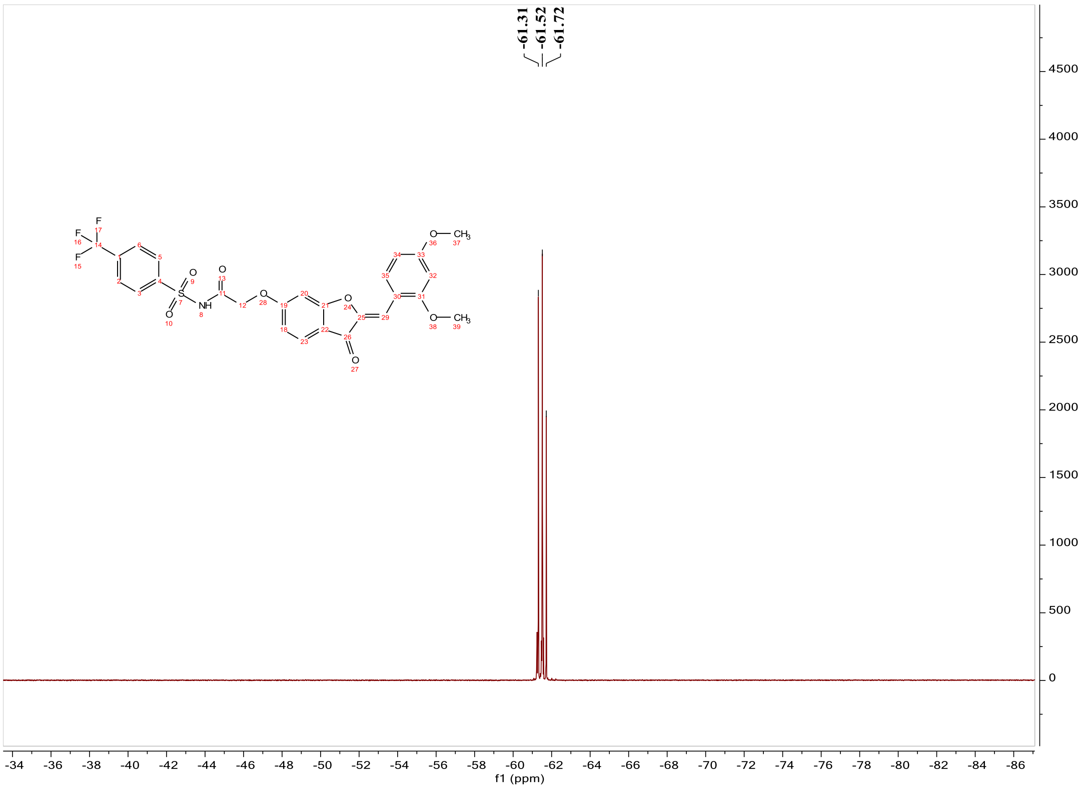

^{19}F NMR (376 MHz, DMSO-d_6) spectrum of compound **D38**

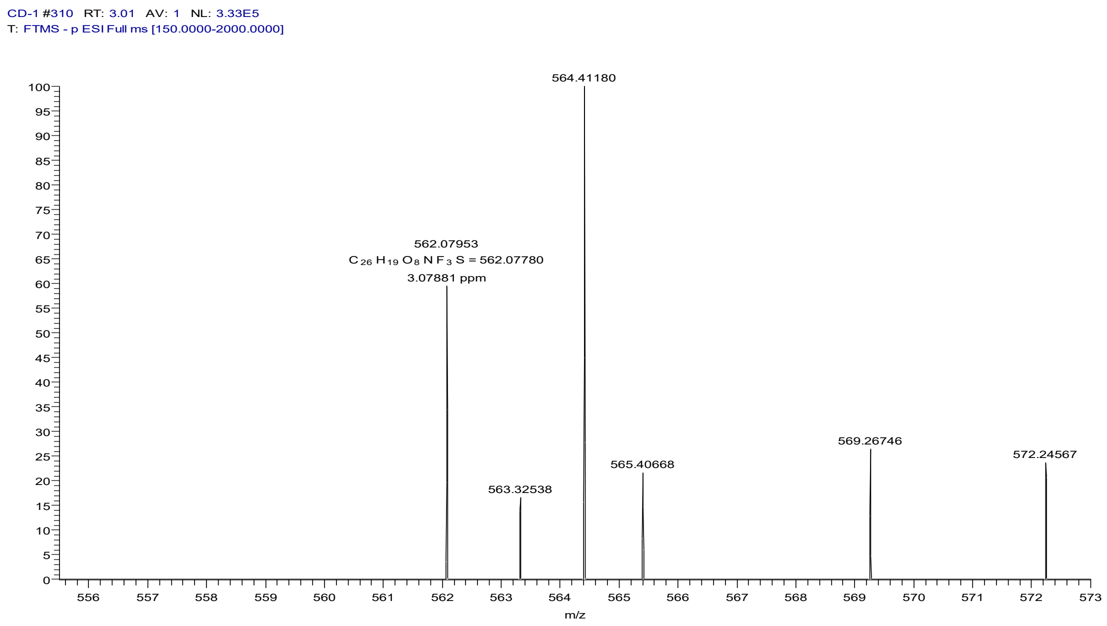

HRMS spectrum of compound **D38**
