# Supplementary figures and images for: Sulfonamine-containing aurone derivatives act as inhibitors of intercellular movement of potato virus Y
Source: iScience. 2025 Oct 30;28(12):113914. doi: 10.1016/j.isci.2025.113914 (PMC12663734; doi:10.1016/j.isci.2025.113914)

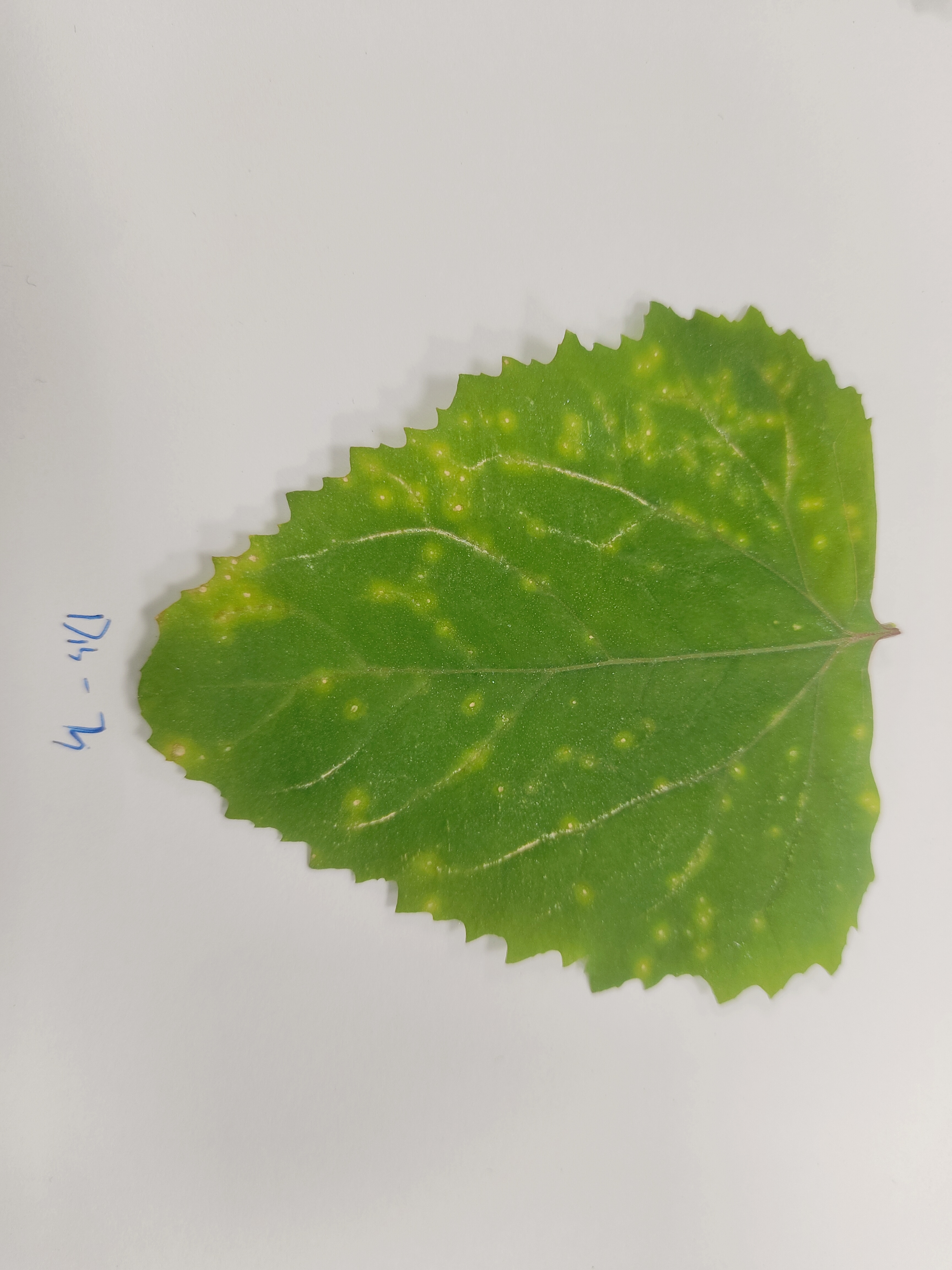

Supplement: Data S1. Unprocessed raw experimental images [file mmc2.zip › Data S1/D13-125.jpg]

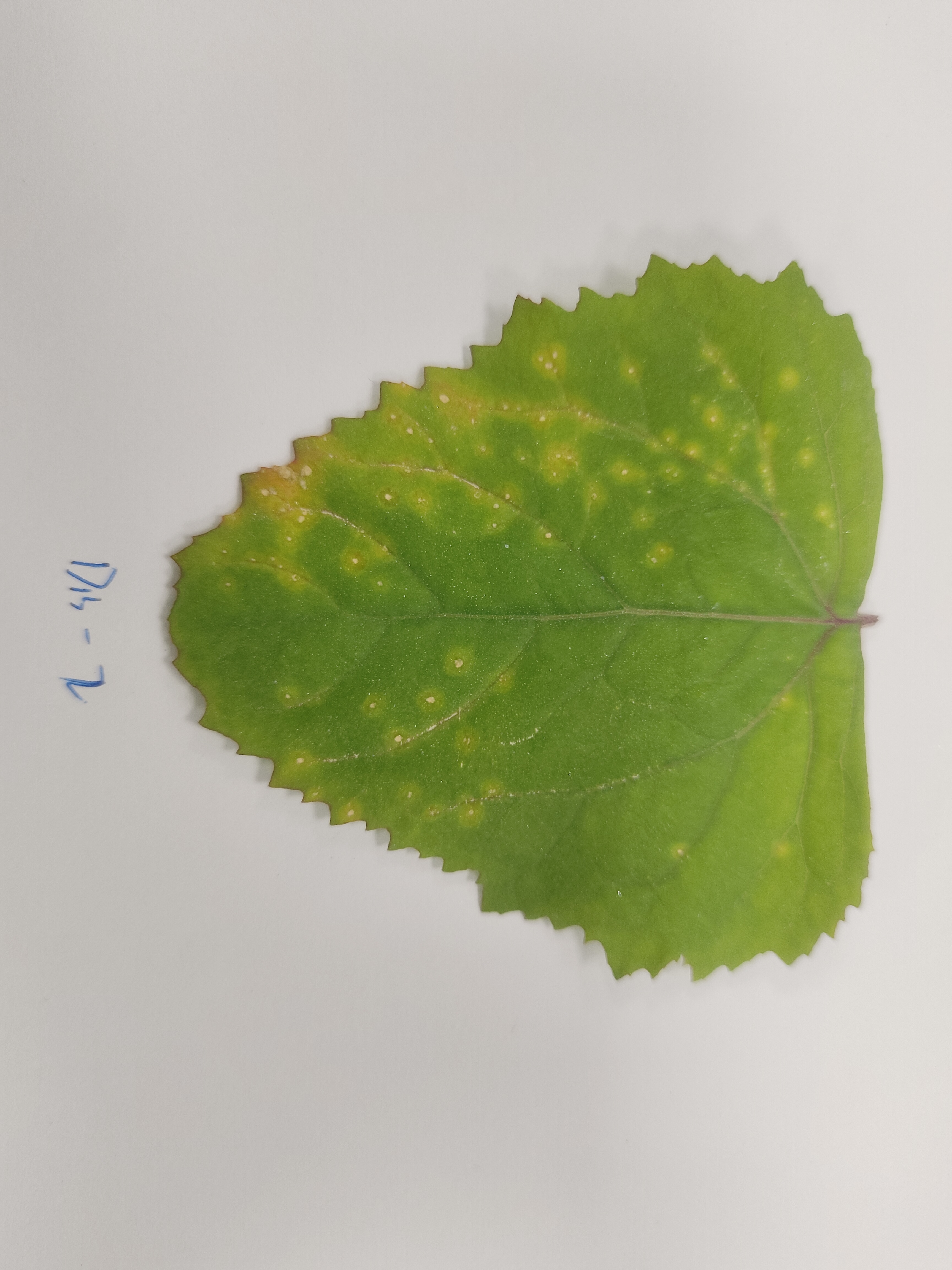

Supplement: Data S1. Unprocessed raw experimental images [file mmc2.zip › Data S1/D13-250.jpg]

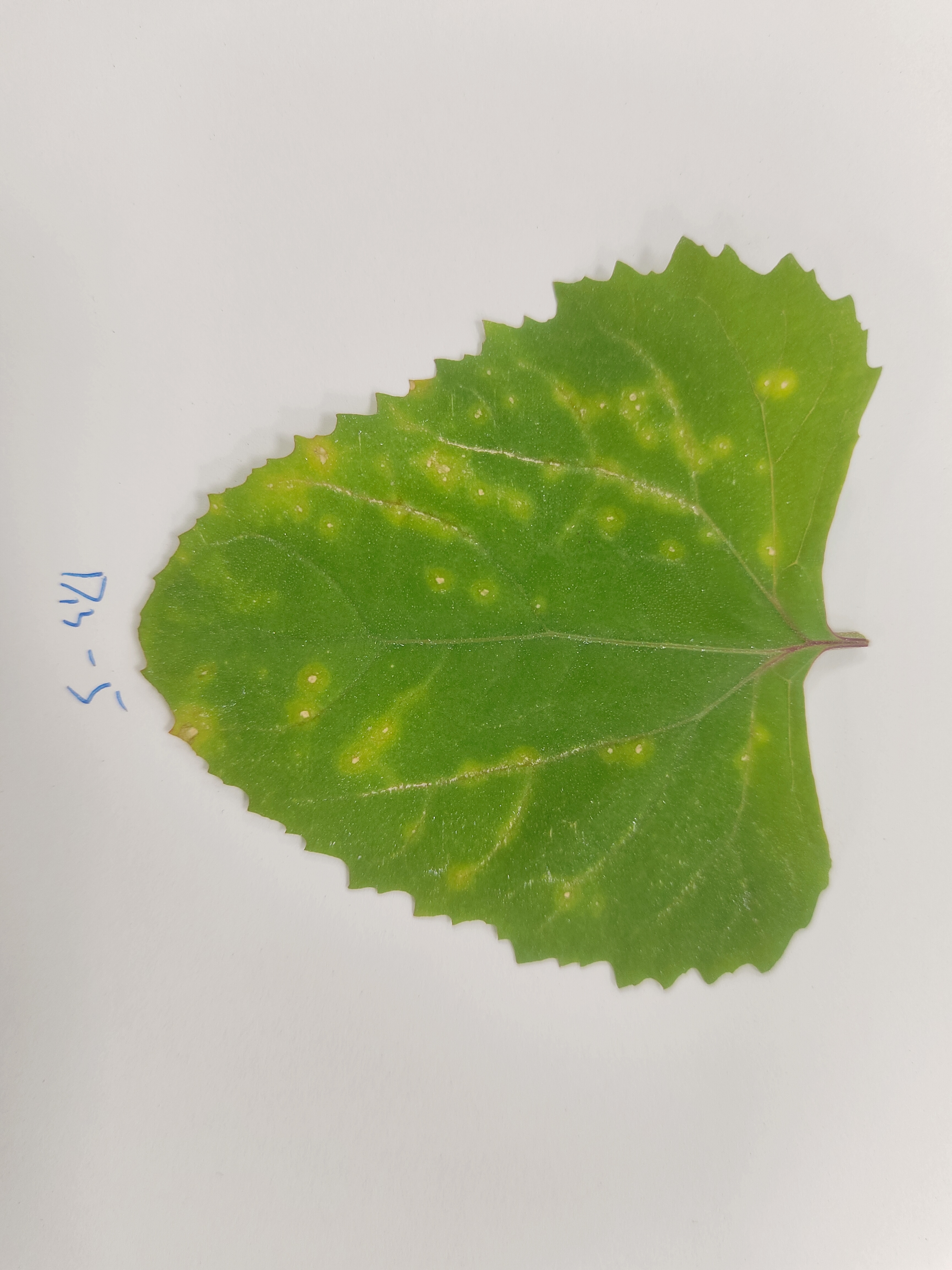

Supplement: Data S1. Unprocessed raw experimental images [file mmc2.zip › Data S1/D13-31.25.jpg]

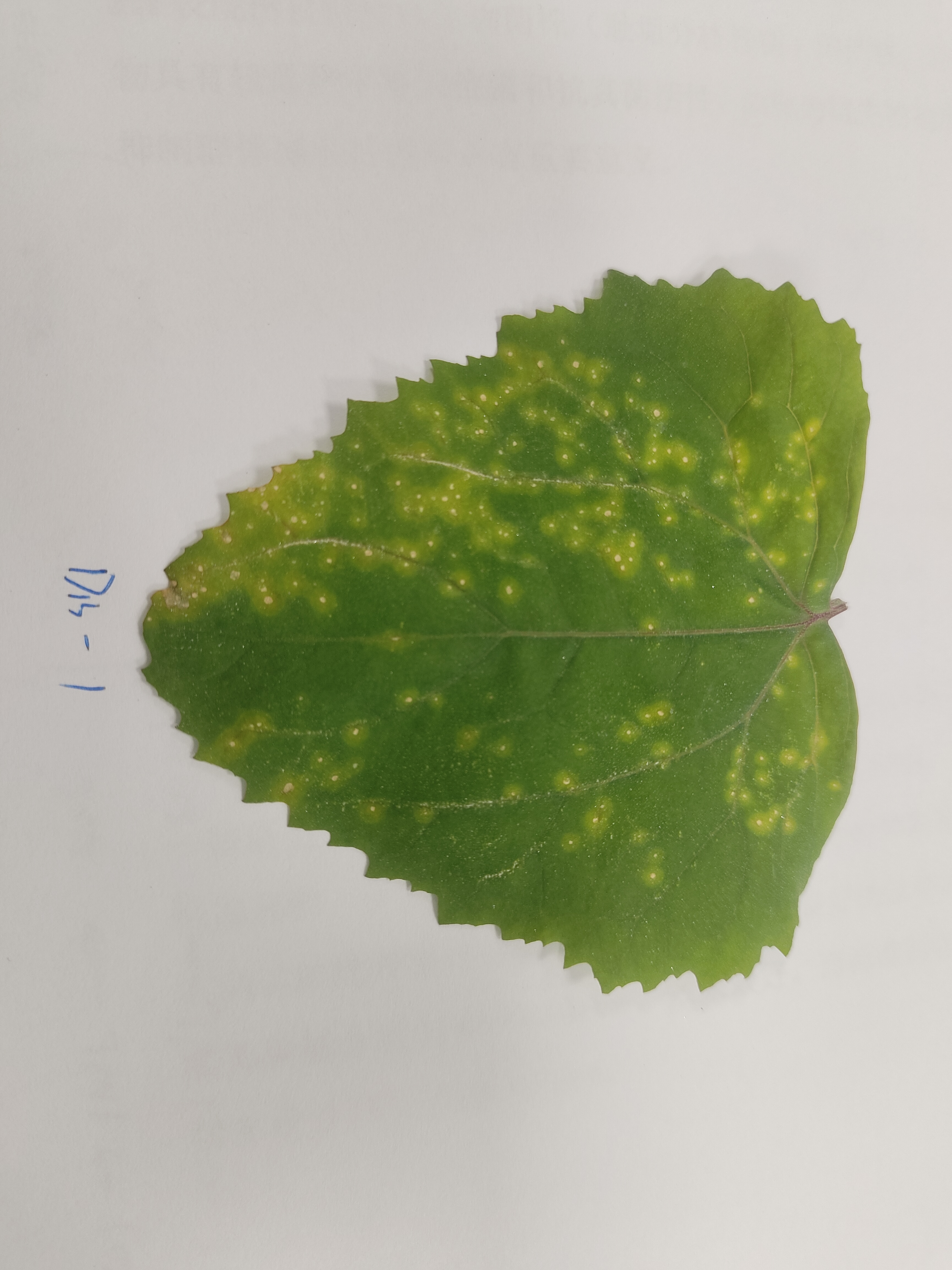

Supplement: Data S1. Unprocessed raw experimental images [file mmc2.zip › Data S1/D13-500.jpg]

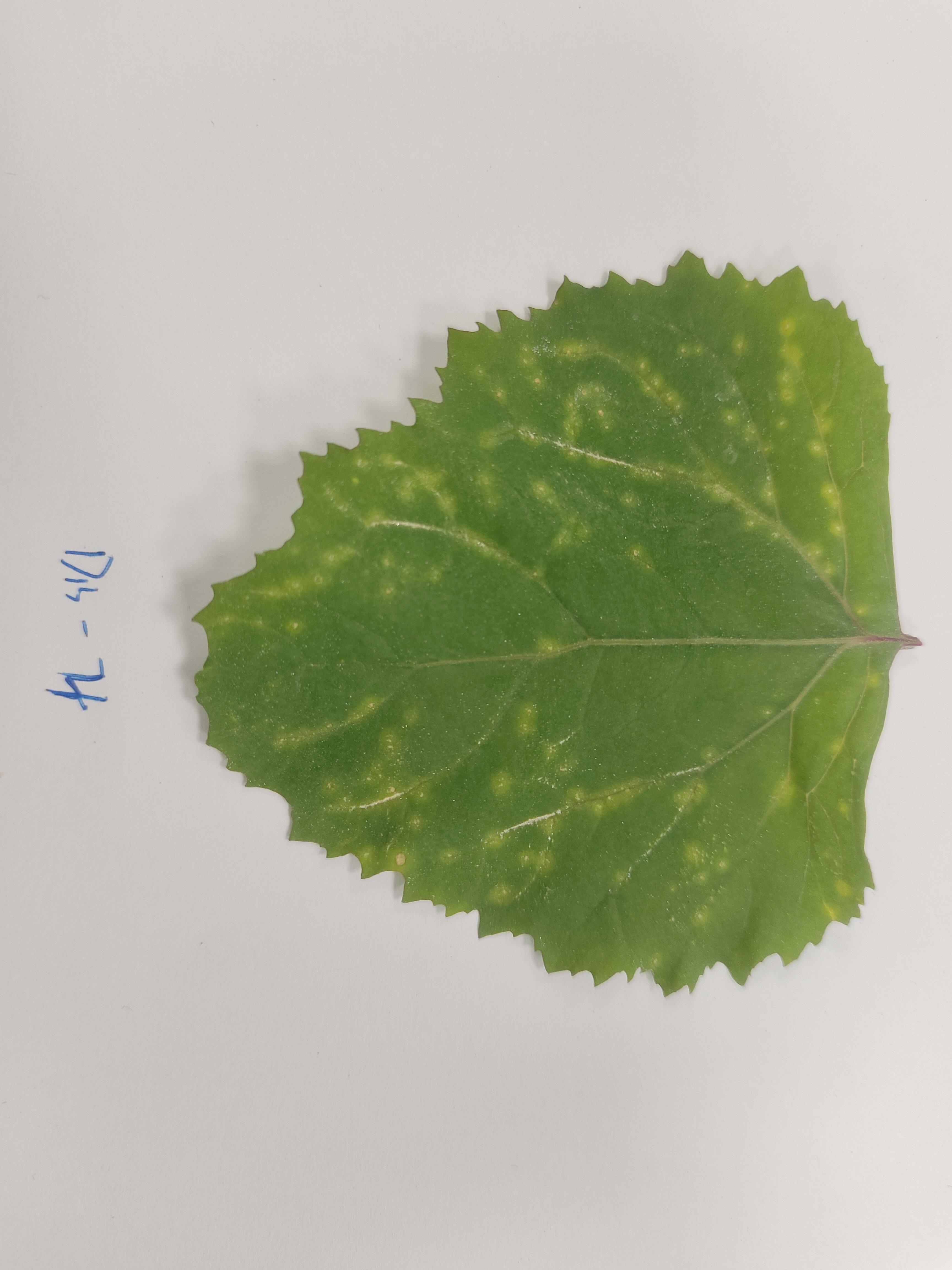

Supplement: Data S1. Unprocessed raw experimental images [file mmc2.zip › Data S1/D13-62.5.jpg]

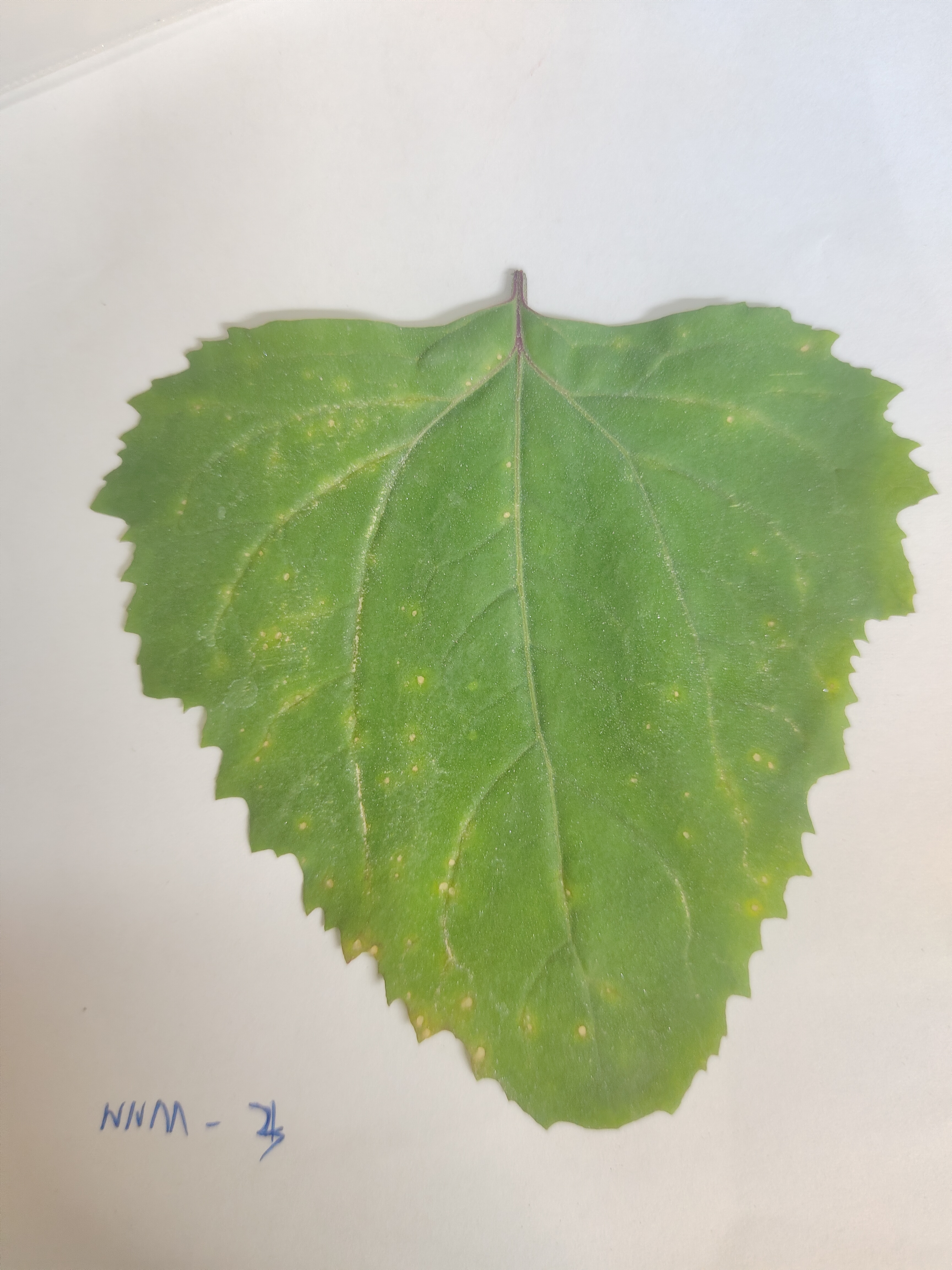

Supplement: Data S1. Unprocessed raw experimental images [file mmc2.zip › Data S1/NNM-125.jpg]

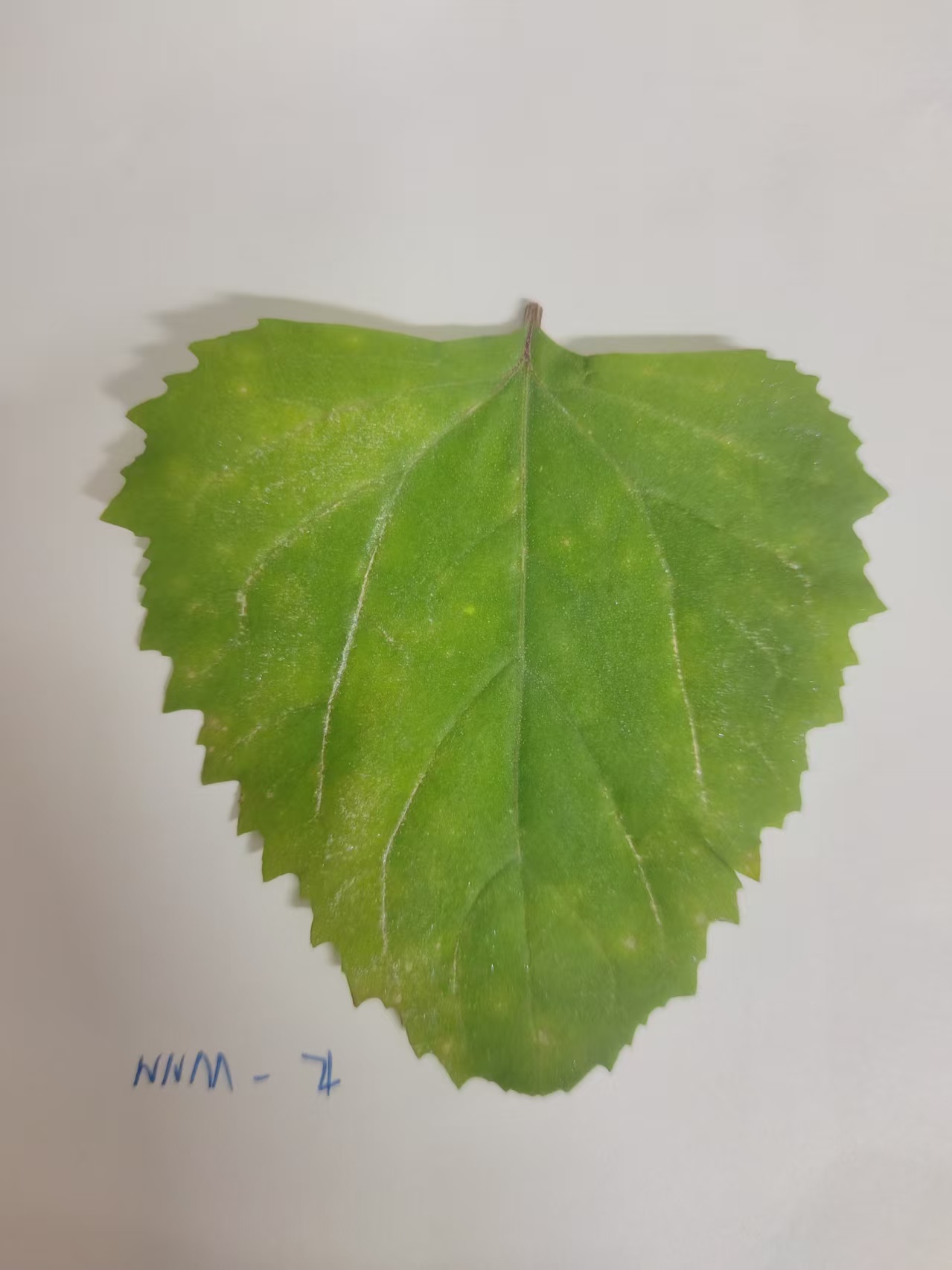

Supplement: Data S1. Unprocessed raw experimental images [file mmc2.zip › Data S1/NNM-250.jpg]

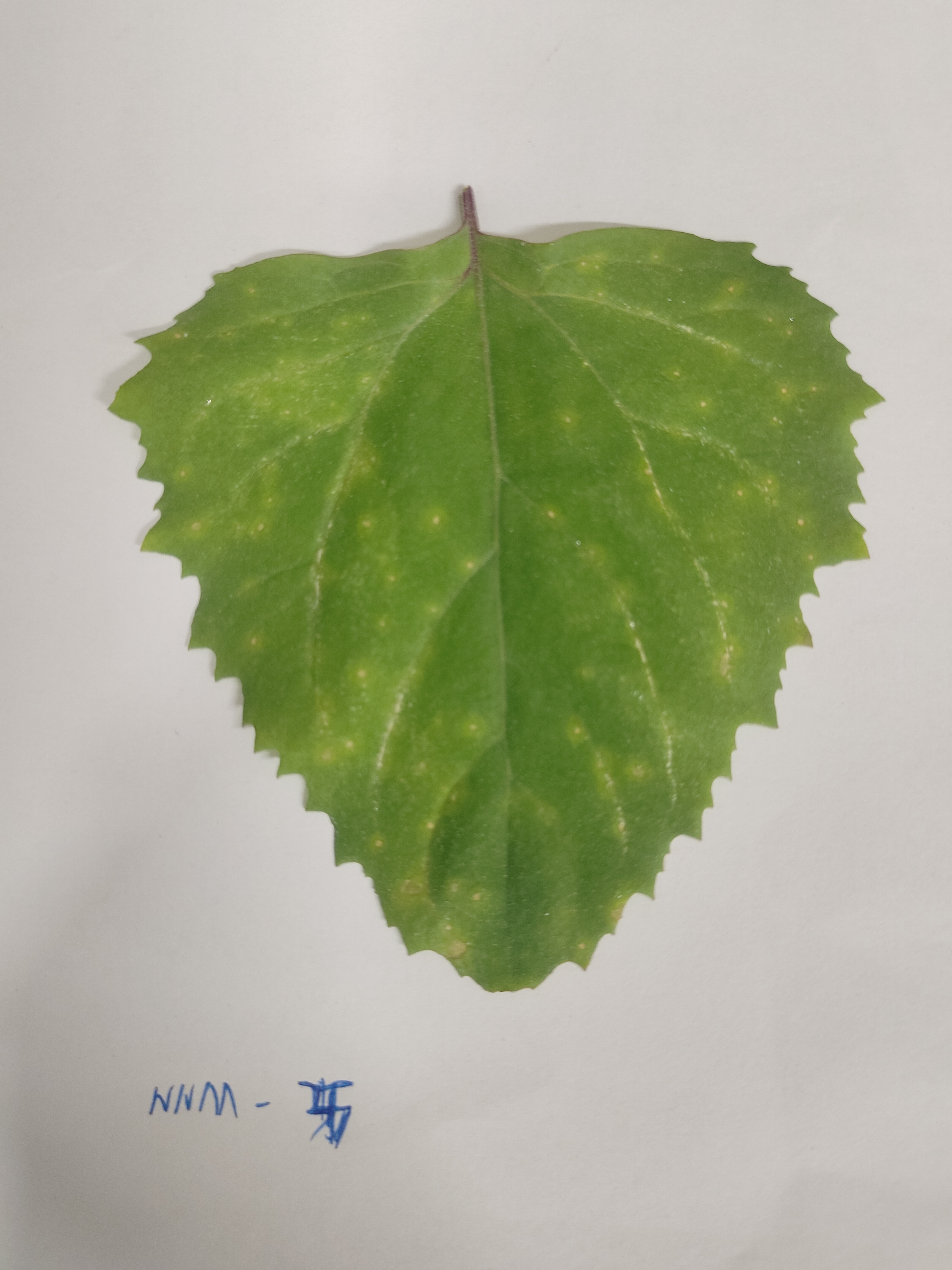

Supplement: Data S1. Unprocessed raw experimental images [file mmc2.zip › Data S1/NNM-31.25.jpg]

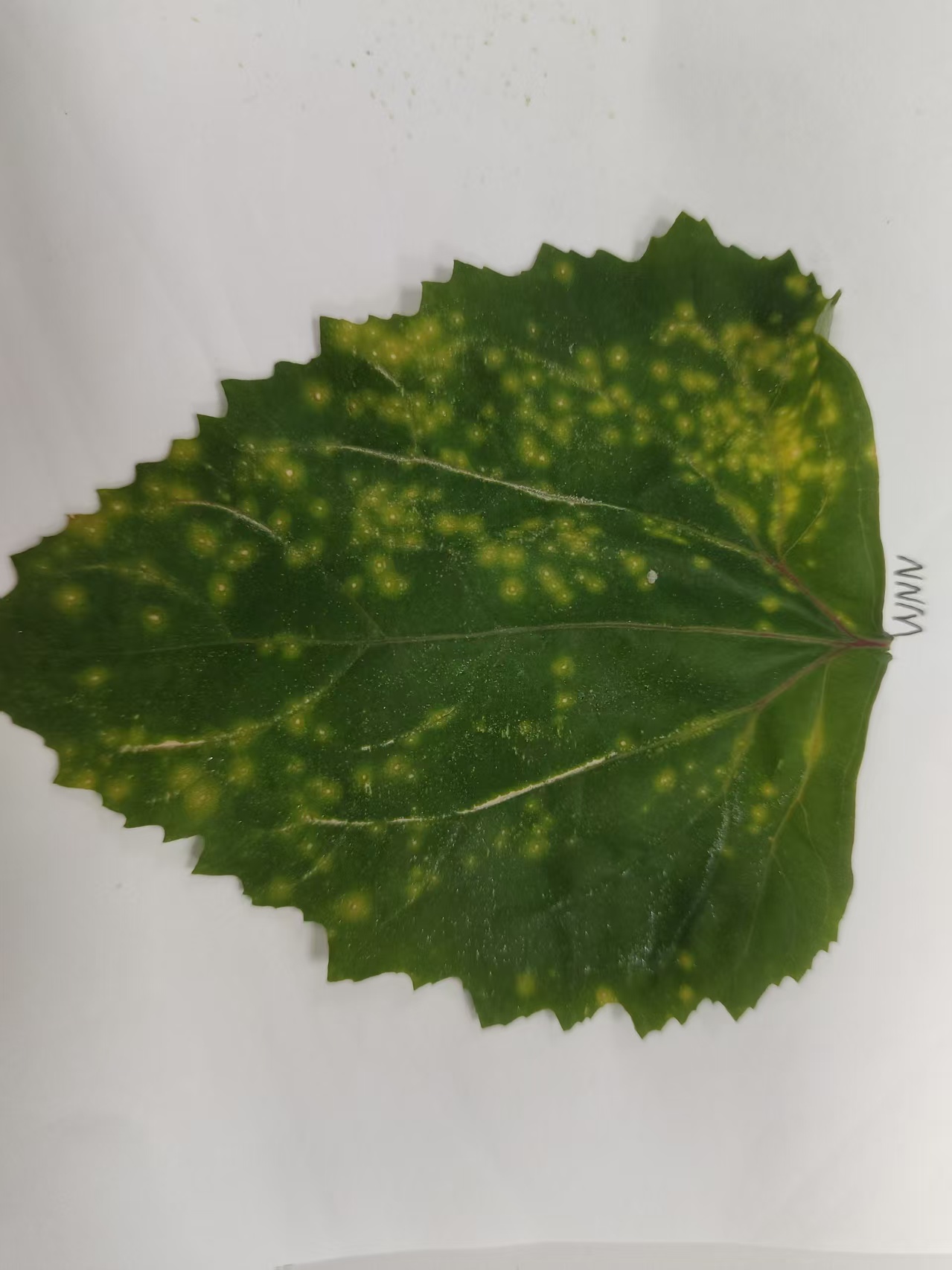

Supplement: Data S1. Unprocessed raw experimental images [file mmc2.zip › Data S1/NNM-500.jpg]

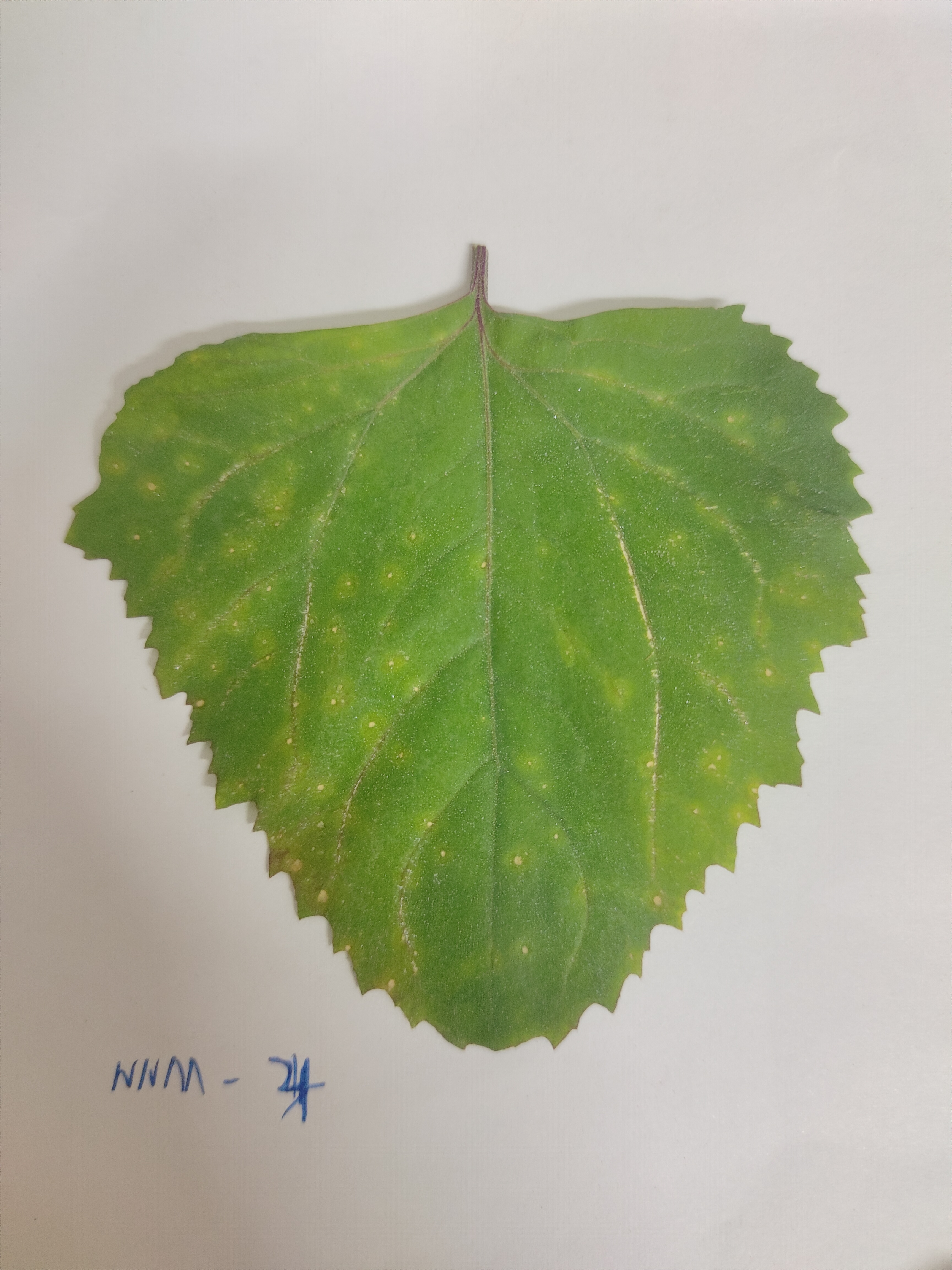

Supplement: Data S1. Unprocessed raw experimental images [file mmc2.zip › Data S1/NNM-62.5.jpg]

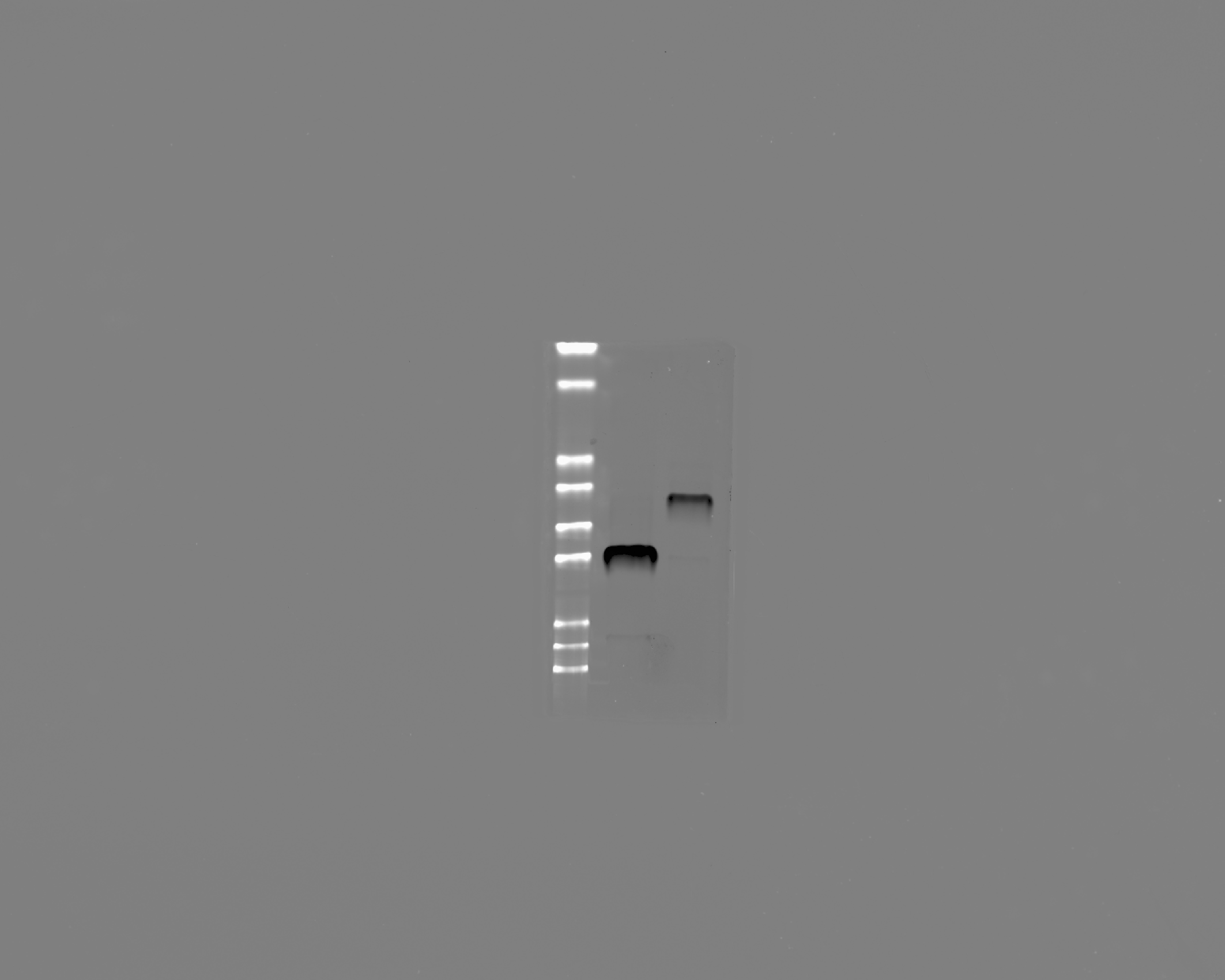

Supplement: Data S1. Unprocessed raw experimental images [file mmc2.zip › Data S1/PVY CPP154A.tif]

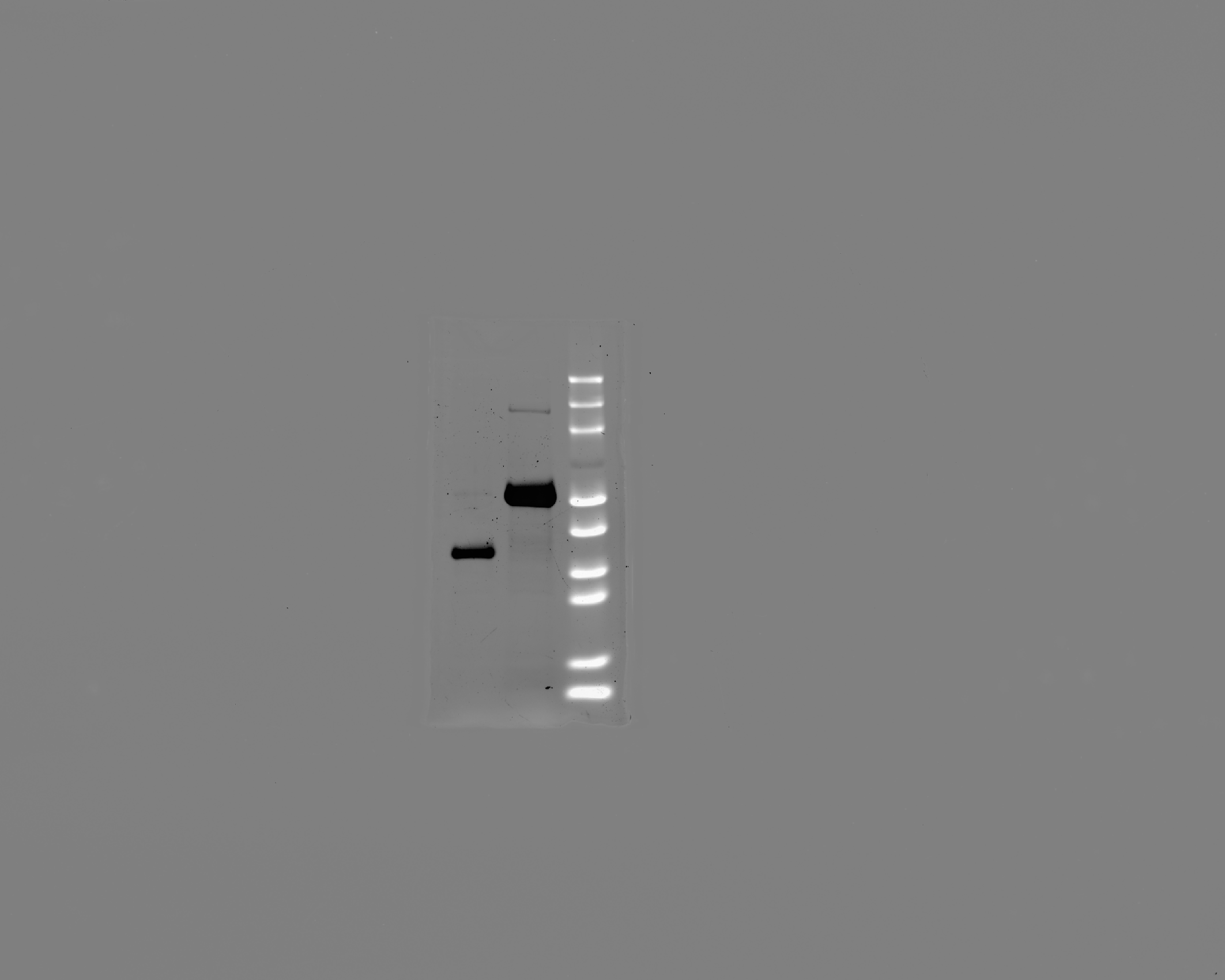

Supplement: Data S1. Unprocessed raw experimental images [file mmc2.zip › Data S1/PVY CPWT.tif]

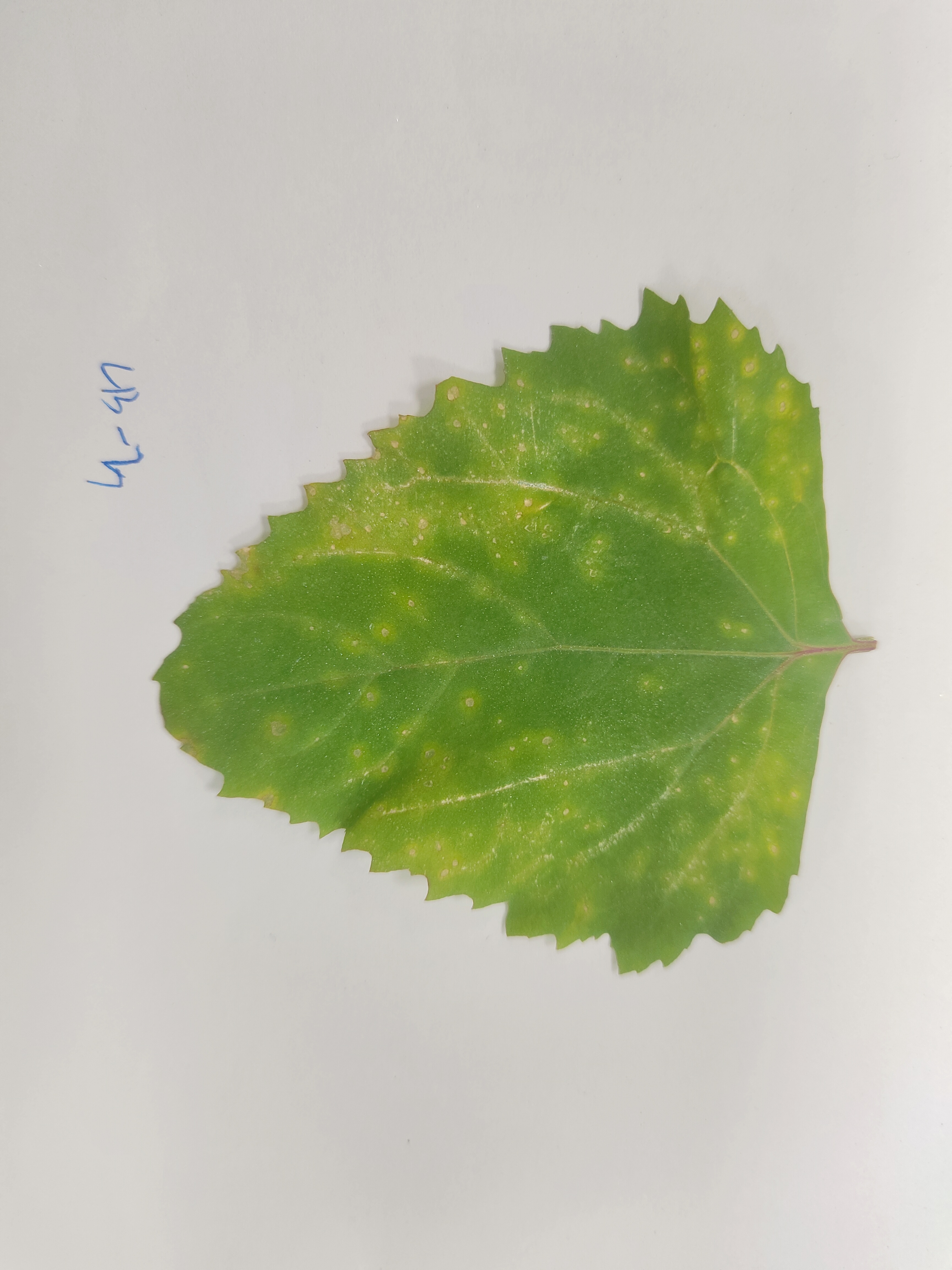

Supplement: Data S1. Unprocessed raw experimental images [file mmc2.zip › Data S1/RBV-125.jpg]

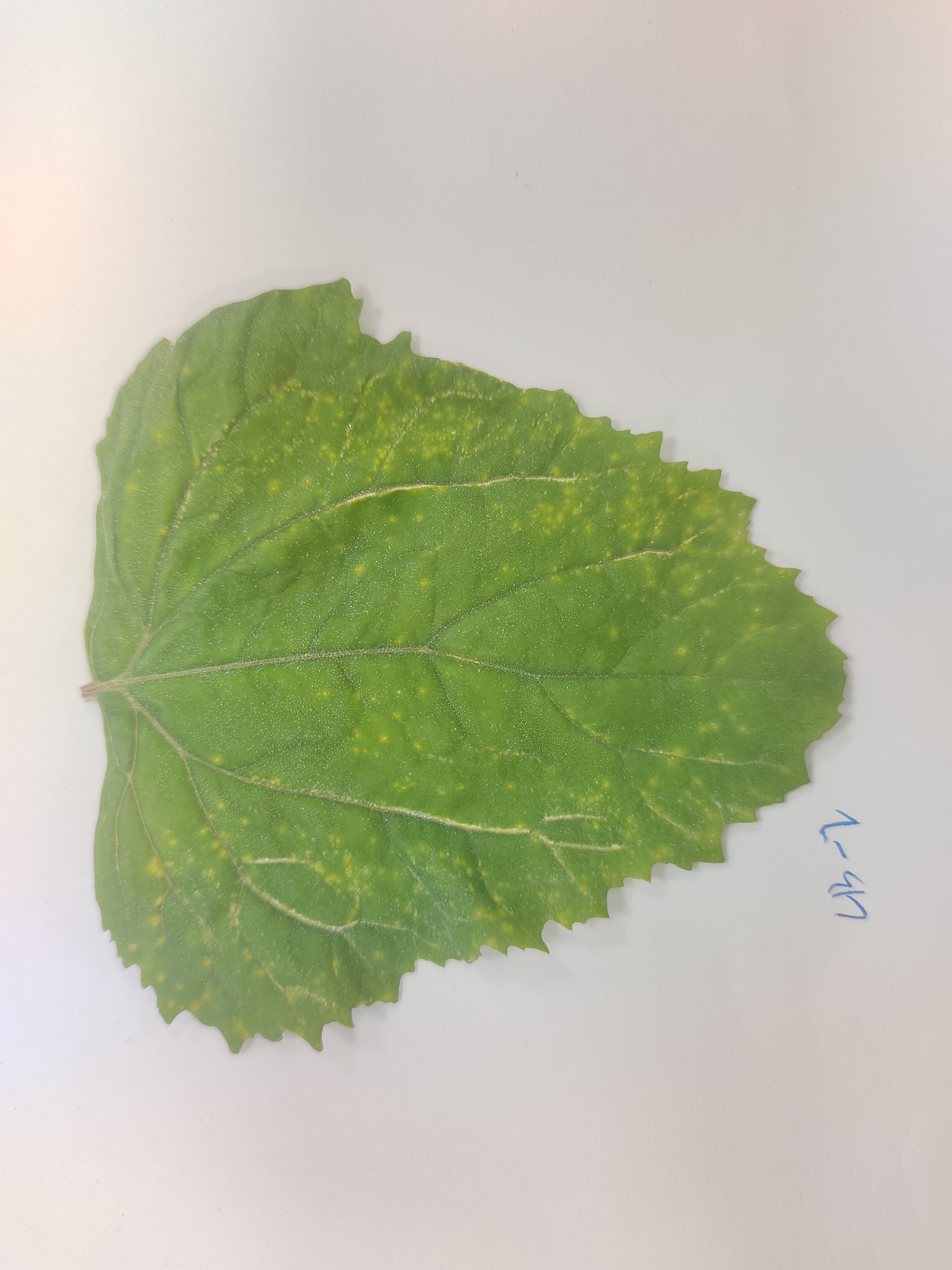

Supplement: Data S1. Unprocessed raw experimental images [file mmc2.zip › Data S1/RBV-250.jpg]

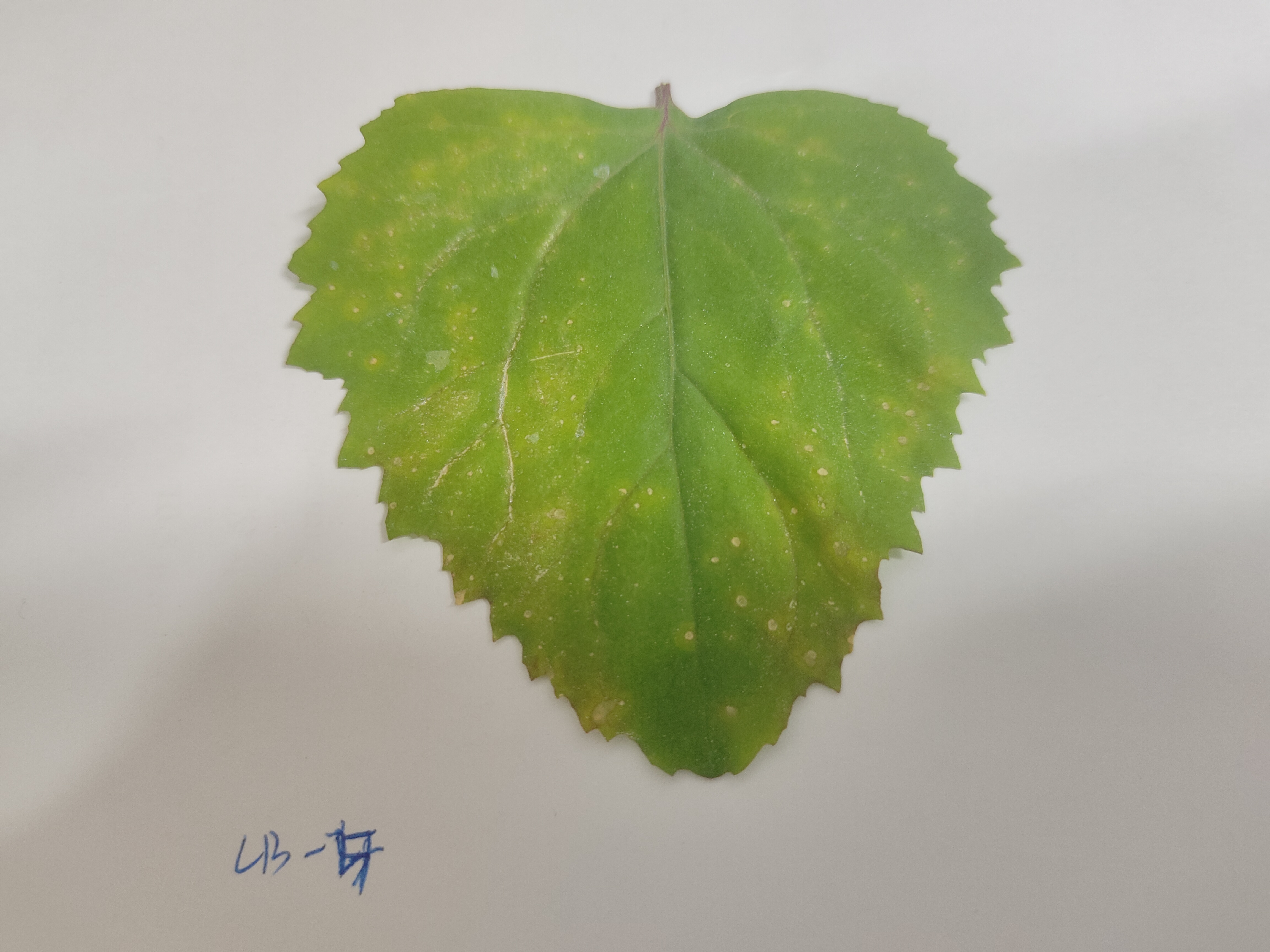

Supplement: Data S1. Unprocessed raw experimental images [file mmc2.zip › Data S1/RBV-31.25.jpg]

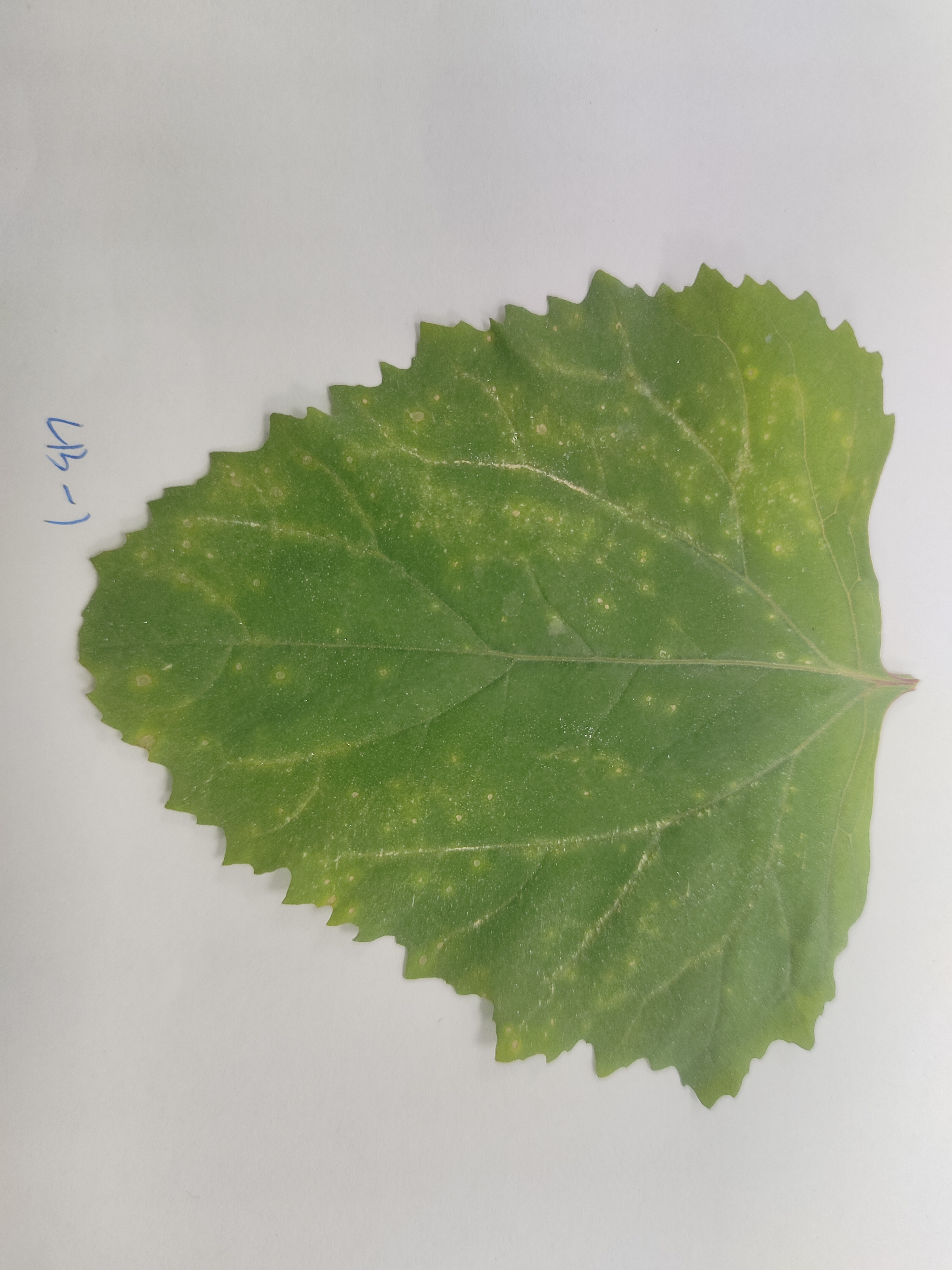

Supplement: Data S1. Unprocessed raw experimental images [file mmc2.zip › Data S1/RBV-500.jpg]

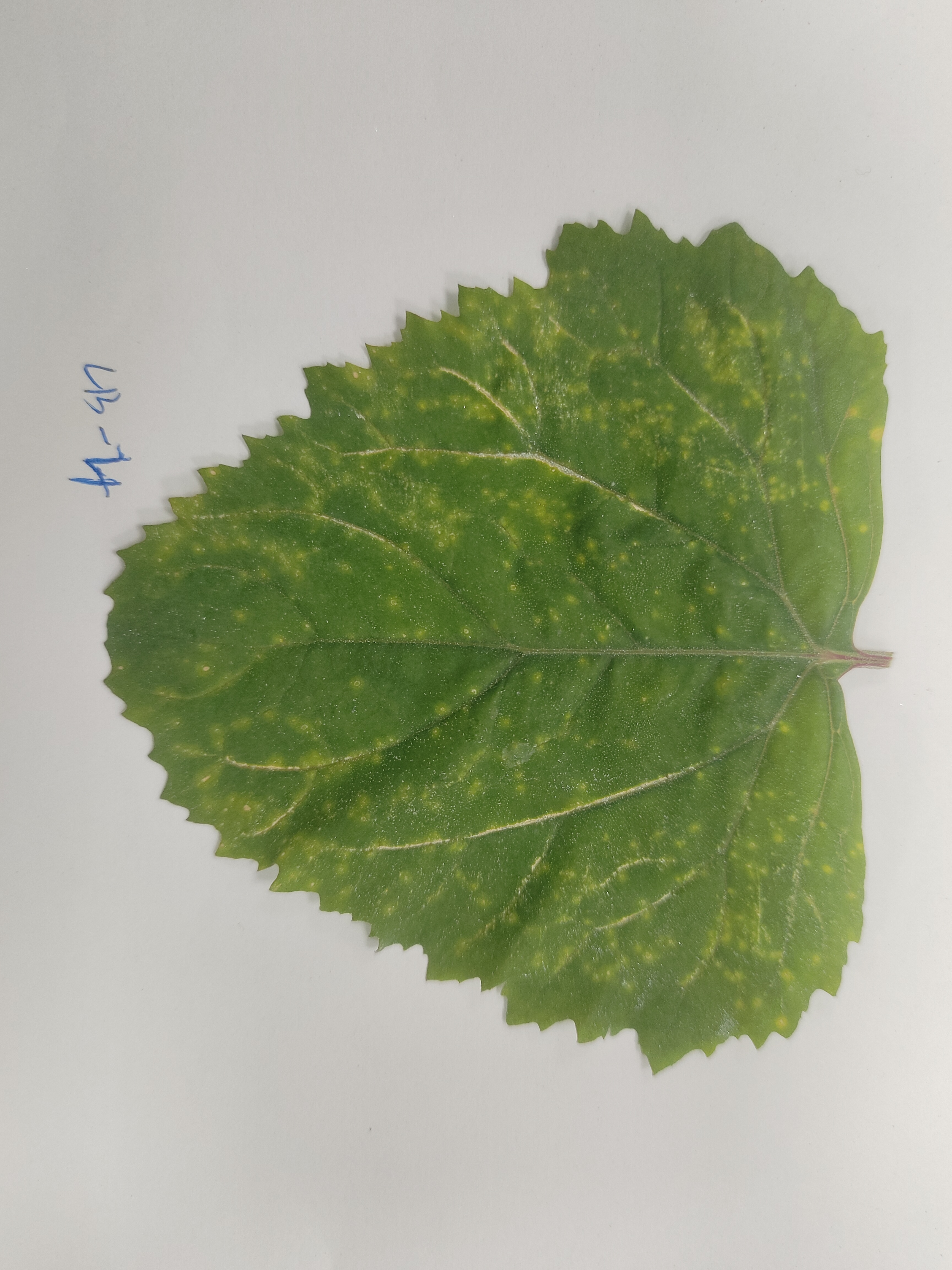

Supplement: Data S1. Unprocessed raw experimental images [file mmc2.zip › Data S1/RBV-62.5.jpg]
